# Bayesian Spatiotemporal Wombling

**DOI:** 10.1080/01621459.2026.2700798

**Published:** 2026-09-08

**Authors:** Aritra Halder, Didong Li, Sudipto Banerjee

**Affiliations:** a Department of Biostatistics & Epidemiology, Drexel University, Philadelphia, PA; b Department of Biostatistics, University of North Carolina, Chapel Hill, NC; c Department of Biostatistics, University of California, Los Angeles, CA

**Keywords:** Gaussian processes, Parametric surfaces, Rates of change, Spatiotemporal boundary analysis, Spatiotemporal processes, Wombling

## Abstract

Stochastic process models for spatiotemporal data underlying random fields find substantial utility in a range of scientific disciplines. Subsequent to predictive inference on the values of the random field (or spatial surface indexed continuously over time) at arbitrary space-time coordinates, scientific interest often turns to gleaning information regarding zones of rapid spatial-temporal change. We develop Bayesian modeling and inference for directional rates of change along a given surface. These surfaces, which demarcate regions of rapid change, are referred to as “*wombling*” *surface boundaries*. Existing methods for studying such changes have often been associated with curves and are not easily extendable to surfaces resulting from curves evolving over time. Our current contribution devises a fully model-based inferential framework for analyzing differential behavior in spatiotemporal responses by formalizing the notion of a “wombling” surface boundary using conventional multi-linear vector analytic frameworks and geometry followed by posterior predictive computations using triangulated surface approximations. We illustrate our methodology with comprehensive simulation experiments followed by multiple applications in neuroimaging; environmental and climate science and pollutant analysis in environmental health. Supplementary materials for this article are available online, including a standardized description of the materials available for reproducing the work.

## 1. Introduction

Statistical inference for rates of change in spatial random fields is instrumental in data-driven scientific discoveries. Such inference is pursued in diverse scientific investigations and can be traced back to the origins of evolutionary biology that have resulted in the discovery of lurking inexplicable differences (see, e.g., Mayr 1944, on the “The Wallace, Weber and Lydekker Lines”). More generally, the study of spatial rates of change in the context of scientific experiments is often referred to as “wombling” (Womble 1951; Gleyze, Bacro, and Allard 2001). “Wombling” broadly refers to delineating zones of rapid change on spatial surfaces usually defined in terms of locations with statistically significant directional gradients (Banerjee, Gelfand, and Sirmans 2003), but also by testing paths or boundaries for rapid change on the spatial field (Banerjee and Gelfand 2006; Fitzpatrick et al. 2010; Qu, Bradley, and Niu 2021).

The methods employed for “wombling” depend on whether the data features a continuous or discrete spatial indexing. For recent approaches to discrete (areal) data, see Wu and Banerjee (2026), and references therein. Here we will treat space and time as continuous when modeling the random field. We consider point-referenced spatiotemporal data, where variables of interest are mapped at locations within an Euclidean coordinate frame (e.g., using a planar map projection) and indexed over time as a realization of a smoothly varying stochastic process. Probabilistic inference on higher order rates of change relies on the smoothness of the parent process (see, e.g., Adler 1981; Greenwood 1984; Kent 1989; Morris, Mitchell, and Ylvisaker 1993; Mardia et al. 1996; Stein 1999; Banerjee and Gelfand 2003; Guindani and Gelfand 2006). Formal inference on rates of change over spatial random fields enjoys a significant literature in diverse scenarios (see, e.g., Banerjee, Gelfand, and Sirmans 2003; Majumdar et al. 2006; Liang, Banerjee, and Carlin 2009; Gabriel, Allard, and Bacro 2011; Heaton 2014; Terres and Gelfand 2015; Wang and Berger 2016; Terres and Gelfand 2016; Wang, Bhattacharya, and Gelfand 2018; Qu, Bradley, and Niu 2021), including recent investigations into optimal plug-in models for gradients (see, e.g., Liu and Li 2026) and inference on spatial curvature processes (see, e.g., Halder, Banerjee, and Dey 2024).

This manuscript develops statistical inference for “wombling” boundaries within a hierarchical spatiotemporal modeling framework (Cressie and Wikle 2015). The literature on rates of change on spatiotemporal random fields is considerably sparser than for spatial random fields. Quick, Banerjee, and Carlin (2013) explored temporal gradients in the context of dynamic Markov random fields, while Quick, Banerjee, and Carlin (2015) developed inference restricted to first-order and mixed spatial-temporal gradients on spatiotemporal random fields. More recently Yu et al. (2023) discussed the utility of temporal rates of change to conduct inference on stationary points in Gaussian process regression models.

Wombling can be contrasted and compared with popular machine learning approaches to detecting clusters (see, e.g., Neal 2000; Birant and Kut 2007), image segmentation (see, e.g., Ronneberger, Fischer, and Brox 2015), hot-spot detection (see, e.g, Bivand and Wong 2018; Di Martino, Pedrycz, and Sessa 2018) and level set methods (see, e.g., Osher and Fedkiw 2001; Lie, Lysaker, and Tai 2006; Boledi et al. 2022). Wombling seeks predictive inference over curves (and surfaces) offering fully model-based uncertainty quantification leveraging rates of change. Clustering aims to find homogeneous regions in the reference domain, unable to offer inference over curves separating such regions. Image segmentation aims to locate boundaries in the interpolated surface images. It often requires careful and expensive training (see, e.g., Akkus et al. 2017) and seldom provides uncertainty quantification. Hot-spot detection techniques for point-referenced data are only able to track regions of rapid change. Level set approaches leverage finite differences to detect boundaries but are unable to offer any uncertainty quantification on them. Wombling yields statistical inference over arbitrary space-time coordinates as well as, on geographic features that may or may not be delineating zones of rapid change (e.g., mountains, rivers, administrative boundaries).

Within a spatiotemporal setting, curves tracking rapid spatial change evolve over time to produce surfaces posing challenges for wombling which has been typically associated to curves. Furthermore, the extent of change may not be captured by first-order derivatives. For example, the rate of change in neural activity may show substantial increments over time—persistently increasing patterns in event related potentials (ERPs) over time, requiring inference on higher-order gradient processes like the spatiotemporal curvature. Here, we construct these processes by jointly modeling the parent Gaussian process and its derivatives to perform inference on higher-order rates of change for spatiotemporal fields. More pertinently for “wombling”, we seek to evaluate average higher-order rates of change not only at arbitrary space-time coordinates in a posterior predictive fashion, but we also extend inference to integrals over surfaces representing temporally evolving zones that are posited to be harboring rapid change, for example, large pockets of smog traversing a geographic space. In this context, spatiotemporal random fields offer significant challenges over purely spatial settings as we develop inference for integrals over surfaces resulting from evolving boundaries in contrast to boundaries that remain static.

The article evolves as follows. Section 2 sets the stage for our methodological framework with an outline of the calculus of spatial-temporal derivative processes and the related distribution theory for legitimate probabilistic inference. Section 3 develops measures of higher-order rates of change and their averages over evolving boundaries (Section 3.3). Section 4 develops a Bayesian inferential framework underscoring computation using surface triangulation to avoid high-dimensional quadrature. Sections 5 and 6 provide simulation experiments and spatiotemporal data analysis to illustrate inference on rates of change at points and over surfaces arising from neuroimaging as ERPs in electroencephalography (EEG) sessions to detect predisposition to alcoholism. Applications featuring analysis of precipitation in Northern California and PM_2.5_ levels during the Canada wildfires are housed in the supplement. Section 7 concludes with a discussion.

## 2. Spatiotemporal Derivative Processes

### 2.1. Calculus for Spatiotemporal Derivatives

Let $\left\{Z(s,t)\;:\;(s,t)\in R^{d}\times R^{+}\right\}$ be a spatial-temporal process, where $s\in R^{d}$ (usually d=2) denotes a spatial location and t is the temporal reference on $R^{+}=[0,\infty )$. We require formal spatiotemporal derivative processes induced by Z(s,t). Such derivatives can be purely spatial, temporal or jointly spatial and temporal underscoring the need for higher-order derivative processes to achieve probabilistic inference. The dimensions for such processes increase with the order of derivatives—the kth-order derivative is a k-fold tensor array—so we use conventional multi-linear algebra in developing higher-order derivatives.

Our subsequent developments rely heavily upon the gradient operator applied to Z(s,t) to produce the (d+1)×1 vector of partial derivatives with respect to each coordinate, that is, $\nabla Z(s,t)≔{\left(\partial_{1}Z(s,t),\partial_{2}Z(s,t),\ldots ,\partial_{d}Z(s,t),\partial_{t}Z(s,t)\right)}^{\text{T}}={\left(\partial_{s}Z(s,t{)}^{\text{T}},\partial_{t}Z(s,t)\right)}^{\text{T}}$, where $\partial_{j}$ denotes the partial derivative with respect to the jth coordinate of $s,\partial_{s}Z(s,t)$ is the d×1 spatial gradient and $\partial_{t}$ is the partial derivative with respect to time. Following Banerjee, Gelfand, and Sirmans (2003), one can obtain spatial-temporal derivatives as limits of finite difference processes. We define the shift operator $D_{h,u,V}Z(s,t)=Z(s+hu,t+hv)$, where $u\in R^{d}$ is a unit vector representing a displacement in spatial location, v∈[0,1] is a scaled temporal shift and h is a scalar denoting the absolute value of the displacements. Then, $\nabla ≔{\text{lim}}_{h\to 0}\;h^{-1}{\left(\left(D_{h,e_{1},0}-1\right),\ldots ,\left(D_{h,e_{d},0}-1\right),\left(D_{h,0,1}-1\right)\right)}^{\text{T}}$, where $\left\{e_{1},\ldots ,e_{d}\right\}$ are the d orthonormal vectors in $R^{d}$. We will specifically use the tensor product $\nabla^{⊗2}≔\nabla ⊗\nabla$, which yields the $(d+1{)}^{2}\times 1$ vector ∇⊗(∇Z(s,t)) by applying ∇ to each element of ∇Z(s,t). Higher-orders are defined recursively as $\nabla^{⊗r}≔\nabla ⊗\left(\nabla^{⊗(r-1)}\right)$. The $(d+1{)}^{r}$ vector $\nabla^{⊗r}Z(s,t)$ contains all partial derivatives of order r in both space and time. Section S1 provides explicit constructions for these tensor products.

The process Z(s,t) is kth order mean-squared differentiable if the elements of $\nabla^{⊗r}Z(s,t)$ are continuously differentiable for $r<k,\;\nabla^{⊗k}Z(s,t)$ are continuous and, for any $h\in R^{+},Z(s+hu,t+hv)=\sum_{r=0}^{k}\;\frac{h^{r}}{r!}{\left(u^{\text{T}},v\right)}^{⊗r}\cdot \nabla^{⊗r}Z(s,t)+o\left(h^{k+1}{\left\|{\left(u^{\text{T}},v\right)}^{\text{T}}\right\|}^{k+1}\right)$, where u is any vector of unit length in $R^{d},v\in [0,1]$ and ${\left(u^{\text{T}},v\right)}^{⊗r}=\left(u^{\text{T}},v\right)⊗\cdots ⊗\left(u^{\text{T}},v\right)$ (Kronecker product with itself r times) is $1\times (d+1{)}^{r}$. We refer to Z(s,t) as the parent process and $\nabla^{⊗r}Z(s,t)$ as the rth order derivative process induced by the parent. *Directional differential processes* are of interest when the change in Z(s,t) is measured along a particular direction. For r=1 and $u\in R^{d},\;{\text{lim}}_{h\to 0}\;h^{-1}{\left(\left(D_{h,u,0}-1\right)Z(s,t),\left(D_{h,0,v}-1\right)Z(s,t)\right)}^{\text{T}}=(u^{\text{T}}⊕v)\nabla Z(s,t)={\left(u^{\text{T}}\partial_{s}Z(s,t),v\partial_{t}Z(s,t)\right)}^{\text{T}}$, where ⊕ is the direct sum operator and $u^{\text{T}}⊕v=\left(\begin{array}{cc} u^{\text{T}} & 0\\ 0_{d}^{\text{T}} & v \end{array}\right)$. More generally, the rth order directional derivative process is ${\left(u^{\text{T}}⊕v\right)}^{⊗r}\nabla^{⊗r}Z(s,t)$.

### 2.2. Distribution Theory for Spatiotemporal Derivative processes

We assume that Z(s,t) is a weakly stationary spatiotemporal process that is zero centered with finite second moment (Gneiting and Guttorp 2010). We write Z(s,t)~GP(0,K(⋅,⋅;θ)) to denote a zero-centered Gaussian process (GP), where $K(𝚫,\delta ;\theta )=\text{cov}\left(Z(s,t),Z\left(s^{\prime },t^{\prime }\right)\right)$ is a stationary spatiotemporal covariance function indexed by process parameters θ with $𝚫=s-s^{\prime }$ and $\delta =t-t^{\prime }$. To ease notation, we suppress the dependence on θ and simply write K(⋅,⋅;θ)=K(⋅,⋅). The covariance function satisfies var $\left\{\sum_{i=1}^{N}\;\;a_{i}Z\left(s_{i},t_{i}\right)\right\}=\sum_{i=1}^{N}\;\sum_{j=1}^{N}\;a_{i}a_{j}K\left({𝚫}_{ij},\delta_{ij}\right)>0$ for any finite collection of spatiotemporal coordinates $\left\{\left(s_{i},t_{i}\right):\right.\;i=1,\ldots ,N\}$, where ${𝚫}_{ij}=s_{i}-s_{j},\delta_{ij}=t_{i}-t_{j}$ and $a_{i},a_{j}\in R,\;i,j=1,2,\ldots ,N$ are not all zero. The process Z(s,t) is mean-squared continuous at (s,t) if $E[Z(s+𝚫,t+\delta )-Z(s,t){]}^{2}\to 0$ as $(𝚫,\delta )\to 0_{d+1}$. Under isotropy, we have $K=K(𝚫,\delta )=\tilde{K}(\|𝚫\|,|\delta |)=\tilde{K}$.

Our subsequent developments rely upon joint processes consisting of the parent and derivatives of different orders. To that end, let $L_{r}\;:\;R\to R^{m_{r}}$ be the *differential operator* of order r, where $m_{r}=\sum_{i=1}^{r}\;(d+1{)}^{i}$, which maps Z(s,t) to the derivative processes up to order r. Thus, $L_{r}Z(s,t)={\left(\nabla^{⊗1}Z(s,t{)}^{\text{T}},\ldots ,\nabla^{⊗r}Z(s,t{)}^{\text{T}}\right)}^{\text{T}}$ is $m_{r}\times 1$. For $L_{r}Z(s,t)$ to be a valid process we require unique pure and partial derivative processes. In general, $L_{r}Z(s,t)$ includes redundancies. For example, $L_{2}Z(s,t)$ includes $\frac{\partial^{2}}{\partial s\partial t}$ and $\frac{\partial^{2}}{\partial t\partial s}$, while $L_{4}Z(s,t)$ includes $\frac{\partial^{4}}{\partial s^{3}\partial t}$ which is not required. We use elimination and permutation matrices to transform $L_{r}Z(s,t)$ to 𝓛rZ(s,t):R→Rmr*,mr*=∑i=0ri+di, comprised of unique partial and pure derivatives.

Let $\partial_{s}^{r}=\partial^{r}/\partial_{1}^{i_{1}}\ldots \partial_{d}^{i_{d}}$ with $\sum_{k=1}^{d}\;i_{k}=r$ and $i_{k}\geq 0$. If $\partial_{t}^{j_{1}+j_{2}}\partial_{s}^{r_{1}+r_{2}-j_{1}-j_{2}}K(𝚫,\delta )$ exists, then $\text{cov}\left(\partial_{t}^{j_{1}}\partial_{s}^{r_{1}-j_{1}}Z(s,t),\partial_{t}^{j_{2}}\partial_{s}^{r_{2}-j_{2}}Z(s+𝚫,t+\delta )\right)=(-1{)}^{r_{1}}\partial_{t}^{j_{1}+j_{2}}\partial_{s}^{r_{1}+r_{2}-j_{1}-j_{2}}K(𝚫,\delta )$ is the covariance between the processes, $\partial_{t}^{j_{1}}\partial_{s}^{r_{1}-j_{1}}Z(s,t)$ and $\partial_{t}^{j_{2}}\partial_{s}^{r_{2}-j_{2}}Z(s+𝚫,t+\delta )$ (see Section S2). The partial derivatives operate element-wise on K(⋅,⋅). Also, ${𝓛}_{r}Z(s,t)$ is a valid GP if $\partial_{t}^{2j}\partial_{s}^{2(r-j)}K\left(0_{d},0\right)$ exists for every j=0,1,…,r. In particular, the case j=r-j=2 requires $\partial_{t}^{4}\partial_{s}^{4}K\left(0_{d},0\right)$ to exist. A GP assumption on Z(s,t) has immediate consequences. If $Z_{1}(s,t)\sim GP\left(0,K_{1}(\cdot ,\cdot )\right)$ and $Z_{2}(s,t)\sim GP\left(0,K_{2}(\cdot ,\cdot )\right)$ independently, then ${𝓛}_{r_{1}}Z_{1}(s,t)$ and ${𝓛}_{r_{2}}Z_{2}(s,t)$ are independent. For $a_{1},a_{2}\in R,\;{𝓛}_{r}\left(a_{1}Z_{1}(s,t)+a_{2}Z_{2}(s,t)\right)=a_{1}{𝓛}_{r}Z_{1}(s,t)+a_{2}{𝓛}_{r}Z_{2}(s,t)$ is a GP. Any sub-vector of ${𝓛}_{r}Z(s,t)$ is a GP. This is useful if selected derivative processes are required for inference as is seen in the next paragraph. In addition, inference extends to differential geometric operators like the divergence and the Laplacian (see Section S3).

We estimate ${𝓛}^{*}Z(s,t)=\left({𝓛}_{s}Z(s,t{)}^{\text{T}},\partial_{t}Z(s,t),\partial_{t}{𝓛}_{s}Z(s,t{)}^{\text{T}}\right.,{\left.\partial_{t}^{2}Z(s,t),\partial_{t}^{2}{𝓛}_{s}Z(s,t{)}^{\text{T}}\right)}^{\text{T}},\;$ where $\;{𝓛}_{s}Z(s,t)\;={\left(\partial_{s}Z(s,t{)}^{\text{T}},{\tilde{\partial_{s}}}^{2}Z(s,t{)}^{\text{T}}\right)}^{\text{T}}$ and ${\tilde{\partial_{s}}}^{2}$ is the operator producing the d2+d vector of unique second order spatial derivatives. The elements of ${𝓛}^{*}Z(s,t)$ capture spatial and temporal rates of change in Z(s,t). The process ${𝓛}_{s}Z(s,t)$ captures spatial gradients and curvature in Z(s,t) while, $\partial_{t}{𝓛}_{s}Z(s,t)$ and $\partial_{t}^{2}{𝓛}_{s}Z(s,t)$ capture temporal gradients and curvature in ${𝓛}_{s}Z(s,t)$ respectively. Casting ${𝓛}^{*}$ as a spatiotemporal differential operator, 𝓛*:R→Rm*,m*=31+d+d(d+1)2-1.Z(s,t)𝓛*Z(s,t) is a zero-centered stationary process. Its cross-covariance function VZ,𝓛*Z(𝚫,δ)=covZ(s,t)𝓛*Z(s,t),Zs′,t′𝓛*Zs′,t′ is a matrix-valued function from $R^{d}\times R$ to $R^{\left(m^{*}+1\right)\times \left(m^{*}+1\right)}$ with $\left(l,l^{\prime }\right)$th element being the covariance between the lth element of Z(s,t)𝓛*Z(s,t) and $l^{\prime }$th element of Zs′,t′𝓛*Zs′,t′. The cross-covariance $V_{Z,{𝓛}^{*}Z}(𝚫,\delta )$ is expressible as a block matrix in terms of K(𝚫,δ) and its derivatives with blocks $\left(\begin{array}{ccc} \partial_{t}^{i-1}\partial_{t}^{j-1}K(𝚫,\delta ) & \partial_{t}^{i-1}\partial_{t}^{j-1}\partial_{s}K(𝚫,\delta {)}^{\text{T}} & \partial_{t}^{i-1}\partial_{t}^{j-1}{\tilde{\partial }}_{s}^{2}K(𝚫,\delta {)}^{\text{T}}\\ -\partial_{t}^{i-1}\partial_{t}^{j-1}\partial_{s}K(𝚫,\delta ) & -\partial_{t}^{i-1}\partial_{t}^{j-1}H_{K}^{11}(𝚫,\delta ) & -\partial_{t}^{i-1}\partial_{t}^{j-1}H_{K}^{12}(𝚫,\delta )\\ \partial_{t}^{i-1}\partial_{t}^{j-1}{\tilde{\partial }}_{s}^{2}K(𝚫,\delta ) & \partial_{t}^{i-1}\partial_{t}^{j-1}H_{K}^{21}(𝚫,\delta ) & \partial_{t}^{i-1}\partial_{t}^{j-1}H_{K}^{22}(𝚫,\delta ) \end{array}\right),\;i,j=1,2,3$. where $\partial_{t}$ acts element-wise on matrices. The entries $H_{K}^{11}(𝚫,\delta )=\text{var}\left(\partial_{s}Z(s,t)\right),H_{K}^{12}(𝚫,\delta )=\text{cov}\left(\partial_{s}Z(s,t),{\tilde{\partial_{s}}}^{2}Z\left(s^{\prime },t^{\prime }\right)\right)=H_{K}^{21}(𝚫,\delta {)}^{\text{T}}$ and $H_{K}^{22}(𝚫,\delta )=\text{var}\left({\tilde{\partial_{s}}}^{2}Z(s,t)\right)$ (see Section S4).

Under isotropy, we have some simplifications when obtaining the entries of $V_{Z,{𝓛}^{*}Z}(𝚫,\delta )$. We write the selected entries as follows: $\partial_{s}K=\left(\frac{d\tilde{K}}{d\|𝚫\|}\right)\frac{𝚫}{\left\|𝚫\right\|},\partial_{t}K=\left(\frac{d\tilde{K}}{d|\delta |}\right)\frac{\delta }{\left|\delta \right|}$ and $\partial_{t}^{2}{\tilde{\partial }}_{s}^{2}K=\left(\frac{d^{2}}{d|\delta {|}^{2}}\frac{d}{d\|𝚫\|}\tilde{K}\right)\frac{P_{2,1}}{\left\|𝚫\right\|}+\left(\frac{d^{2}}{d|\delta {|}^{2}}\frac{d^{2}}{d\|𝚫{\|}^{2}}\tilde{K}-\frac{1}{\left\|𝚫\right\|}\frac{d^{2}}{d|\delta {|}^{2}}\frac{d}{d\|𝚫\|}\tilde{K}\right)\frac{P_{2,2}}{\|𝚫{\|}^{2}}$ where P⋅,⋅ are permutation matrices (see Section S5.1 for details). Simplifications owing to isotropy also arise in the expressions for the covariance of directional spatiotemporal derivative processes. For instance, the variance for the directional spatiotemporal curvature process has the following expression: $\frac{3}{\|𝚫{\|}^{2}}\left(1-5\frac{{\left(u^{\text{T}}𝚫\right)}^{2}}{\|𝚫{\|}^{2}}\right)\left(1-\frac{{\left(u^{\text{T}}𝚫\right)}^{2}}{\|𝚫{\|}^{2}}\right)\left(\frac{d^{4}}{d|\delta {|}^{4}}\frac{d^{2}}{{\left.d\|𝚫\|\right|}^{2}}\tilde{K}-\frac{1}{\left\|𝚫\right\|}\frac{d^{4}}{d|\delta {|}^{4}}\frac{d}{d\|𝚫\|}\tilde{K}\right)+\frac{6}{\left\|𝚫\right\|}\frac{{\left(u^{\text{T}}𝚫\right)}^{2}}{\|𝚫{\|}^{2}}\left(1-\frac{{\left(u^{\text{T}}𝚫\right)}^{2}}{\|𝚫{\|}^{2}}\right)\left(\frac{d^{4}}{d|\delta {|}^{4}}\frac{d^{3}}{d\|𝚫{\|}^{3}}\tilde{K}\right)+{\left(\frac{u^{\text{T}}𝚫}{\left\|𝚫\right\|}\right)}^{4}\left(\frac{d^{4}}{d|\delta {|}^{4}}\frac{d^{4}}{d\|𝚫{\|}^{4}}\tilde{K}\right)$. Similar results can be obtained for all spatiotemporal directional derivatives (see Section S5.2). We observe that ${𝓛}^{*}Z(s,t)$ is no longer isotropic.

We use spectral theory to characterize covariance functions that admit such processes. The existence of fourth-order spectral moments for space and time ensures admittance of the differential processes within ${𝓛}^{*}Z(s,t)$. We adopt non-separable spatiotemporal covariance functions of the form, $K(𝚫,\delta )=\tilde{K}(\|𝚫\|,|\delta |)=\frac{\sigma^{2}}{\psi {\left(|\delta {|}^{2}\right)}^{d/2}}\varphi \left(\frac{\|𝚫{\|}^{2}}{\psi \left(|\delta {|}^{2}\right)}\right)$, where φ(x) and ψ(x) are complete monotone, and positive with complete monotone derivative on [0,∞), respectively (see, e.g., Gneiting 2002). Specifically, we use the Matérn kernel, $(x)={\left(2^{\nu -1}\Gamma (\nu )\right)}^{-1}{\left(\phi_{s}x^{1/2}\right)}^{\nu }K_{\nu }\left(\phi_{s}x^{1/2}\right),\phi_{s}>0,\nu >0$, where $K_{\nu }(\cdot )$ is the modified Bessel function of the second kind, and $\psi (x)={\left(\phi_{t}^{2}x^{\alpha }+1\right)}^{\beta },a>0,0<\alpha \leq 1,0\leq \beta \leq 1$. We fix α=β=1, producing the covariance kernel,

(1) $$\tilde{K}(\|𝚫\|,|\delta |;\theta )=\frac{\sigma^{2}}{2^{v-1}\Gamma (v)A_{t}^{d/2}}{\left(\frac{\phi_{s}\|𝚫\|}{A_{t}^{1/2}}\right)}^{v}\times K_{v}\left(\frac{\phi_{s}\|𝚫\|}{A_{t}^{1/2}}\right),\;A_{t}=\phi_{t}^{2}|\delta {|}^{2}+1.$$

Existence of the fourth spectral moment requires the fractal parameter, v>2. Although irregularities exist in smoothness for separable covariance functions (see, e.g., Stein 2005), characterizing smoothness for such functions is relatively simpler. Section S6 provides further details. Establishing the validity of ${𝓛}^{*}Z(s,t)$, requires no further assumptions.

Inference on ${𝓛}^{*}Z\left(s_{0},t_{0}\right)$ at an arbitrary space-time location $\left(s_{0},t_{0}\right)$ is sought through the joint distribution, $P\left[𝓩,{ℒ}^{*}Z\left(s_{0},t_{0}\right)\right]$, where $𝓩={\left(Z\left(s_{1},t_{1}\right),\ldots ,Z\left(s_{N},t_{N}\right)\right)}^{\text{T}}$ is an N×1 vector of space-time measurements. Let $\sum_{𝓩}=\left[\text{cov}\left\{Z\left(s_{i},t_{i}\right),Z\left(s_{i^{\prime }},t_{i^{\prime }}\right)\right\}\right]=\left[K\left({𝚫}_{ii^{\prime }},\delta_{ii^{\prime }}\right)\right]$ be the N×N covariance matrix associated with our measurements where, ${𝚫}_{ii^{\prime }}=s_{i}-s_{i^{\prime }},\delta_{ii^{\prime }}=t_{i}-t_{i^{\prime }},i,i^{\prime }=1,\ldots ,N$. Let ${𝓚}_{0}=\text{cov}\left(𝓩,{𝓛}^{*}Z\left(s_{0},t_{0}\right)\right)=\left(\begin{array}{c} {𝓚}_{10}{\left({𝚫}_{10},\delta_{10}\right)}^{\text{T}}\\ \vdots \\ {𝓚}_{N0}{\left({𝚫}_{N0},\delta_{N0}\right)}^{\text{T}} \end{array}\right)$ be a $N\times m^{*}$ matrix with columns ${𝓚}_{i0}\left({𝚫}_{i0},\delta_{i0}\right)=\text{cov}\left\{Z\left(s_{i},t_{i}\right),{𝓛}^{*}Z\left(s_{0},t_{0}\right)\right\}$ for i=1,…,N. The joint distribution is

(2) (𝓩𝓛*Zs0,t0)~𝒩N+m*0N+m*,∑𝓩𝓚0𝓚0TV𝓛*Z0d,0,

where $V_{{𝓛}^{*}Z}\left(0_{d},0\right)$ is the cross-covariance matrix of ${𝓛}^{*}Z(s,t)$ evaluated at $\left(0_{d},0\right)$. Section 4 develops posterior predictive inference for ${𝓛}^{*}Z\left(s_{0},t_{0}\right)$.

## 3. Wombling on Spatiotemporal Surfaces

Bayesian spatiotemporal wombling requires inference for integrals over surfaces that enclose regions of rapid change in space-time coordinates. Such surfaces are defined from planar curves tracking rapid change in space and across time. In traditional wombling, which hitherto has been purely spatial, the curves remain fixed over time (e.g., geographic features like rivers, mountain ranges or administrative boundaries) and we seek to measure change along these curves. Alternatively, the regions of interest can vary over time, for example, scalp regions with ERPs over a certain threshold or, tracking smog arising from wildfires (see the supplement, S15.2) in the interest of public health, regions evolve with changing wind patterns. This will be the focus of our discussion.

We focus on real valued processes, Z(s,t), over $H^{3}=R^{2}\times R^{+}\subset R^{3}$ using familiar concepts in differential geometry (see, e.g., Spivak 1979) for the ensuing developments. We explore spatiotemporal derivatives and curvatures over smooth and regular surfaces with boundaries. This extends the development of derivatives and curvatures over planar curves required for curvilinear wombling within a purely spatial context (see, e.g., Banerjee and Gelfand 2006; Halder, Banerjee, and Dey 2024) to surfaces in space-time.

### 3.1. Wombling Measures for Surfaces

In general, we assume that 𝒞 is a surface in $H^{3}$. Our aim is to statistically estimate the average directional gradient of Z(s,t) along 𝒞 from the realized data. We define such measures of interest on parametric surfaces. Let $𝒞=\left\{\left(s_{x}(\omega ,v),s_{y}(\omega ,v),t(v)\right)\;:\;(\omega ,v)\in {𝒟}_{\omega }\times {𝒟}_{v}\right\}$ be a smooth and regular surface with domain, ${𝒟}_{\omega }\times {𝒟}_{v}\subset H^{2}=R\times R^{+}$ and let t(v)=v for convenience. The *outward* pointing normal and unit normal to 𝒞 at (ω,v) are

(3) $$\bar{n}(s(\omega ,v),v)\;={\left(\frac{\partial s_{y}(\omega ,v)}{\partial \omega },-\frac{\partial s_{x}(\omega ,v)}{\partial \omega },\left|\frac{\partial \left(s_{x},s_{y}\right)}{\partial (\omega ,v)}\right|\right)}^{\text{T}},\;n(s(\omega ,v),v)\;=\frac{\bar{n}(s(\omega ,v),v)}{\left\|\bar{n}(s(\omega ,v),v)\right\|},$$

respectively, where $\left|\frac{\partial \left(s_{x},s_{y}\right)}{\partial (\omega ,v)}\right|=\frac{\partial s_{x}(\omega ,v)}{\partial \omega }\frac{\partial s_{y}(\omega ,v)}{\partial v}-\frac{\partial s_{y}(\omega ,v)}{\partial \omega }\frac{\partial s_{x}(\omega ,v)}{\partial v}$. Let ${\bar{n}}_{s}(\omega ,v)={\left(\frac{\partial s_{y}(\omega ,v)}{\partial \omega },-\frac{\partial s_{x}(\omega ,v)}{\partial \omega }\right)}^{\text{T}}$ and ${\overset{‾}{n}}_{t}(\omega ,v)=\left|\frac{\partial \left(s_{x},s_{y}\right)}{\partial (\omega ,v)}\right|$. If $s_{x},s_{y}$ are injective functions and all partial derivatives in (3) exist, then the surface 𝒞 is smooth. Also, 𝒞 is regular if $\left\|{\bar{n}}_{s}(\omega ,v)\right\|\neq 0$ and the unit normal n(s(ω,v),v) in (3) is well-defined. We denote the surface area of 𝒞, as 𝒜(𝒞) given by

(4) $$\iint_{{𝒟}_{v}\times {𝒟}_{\omega }}\;\;\|\bar{n}(s(\omega ,v),v)\|d\omega \;dv=\iint_{{𝒟}_{v}\times {𝒟}_{\omega }}\;\;\left\{{\left(\frac{\partial s_{y}(\omega ,v)}{\partial \omega }\right)}^{2}\right.{\left.\;+{\left(\frac{\partial s_{x}(\omega ,v)}{\partial \omega }\right)}^{2}+{\left|\frac{\partial \left(s_{x},s_{y}\right)}{\partial (\omega ,v)}\right|}^{2}\right\}}^{\frac{1}{2}}d\omega \;dv.$$

If ${𝒟}_{v}=\left[v_{0},v_{1}\right]$ and ${𝒟}_{\omega }=\left[\omega_{0},\omega_{1}\right]$, then $𝒜(𝒞)={𝒜}_{\omega_{0},v_{0}}\left(\omega_{1},v_{1}\right)=\int_{v_{0}}^{v_{1}}\;\int_{\omega_{0}}^{\omega_{1}}\;\|\bar{n}(s(\omega ,v),v)\|d\omega \;dv$ and, hence, $\text{d}{𝒜}_{v_{0},\omega_{0}}(\omega ,v)=\|\bar{n}(s(\omega ,v),v)\|d\omega \;dv$. We denote, $n_{t}(\omega ,v)=n_{t}$ and $n_{s}(\omega ,v)=n_{s}$ for brevity of ensuing expressions. For any point (s(ω,v),v) on 𝒞, let ${𝓛}_{n_{t},n_{s}}^{*}Z(s(\omega ,v),v)=N_{st}{𝓛}^{*}Z(s(\omega ,v),v)$, be the 8 × 1 directional differential process along the *normal direction matrix*, $N_{st}=\left({\tilde{n}}_{s,-1}⊕n_{t}{\tilde{n}}_{s}⊕n_{t}^{2}{\tilde{n}}_{s}\right)$, where ${\tilde{n}}_{s}=1⊕{\tilde{n}}_{s,-1},{\tilde{n}}_{s,-1}=n_{s}^{\text{T}}⊕{\left\{{\tilde{n}}_{s}^{⊗2}\right\}}^{\text{T}}$ and ${\tilde{n}}_{s}^{⊗2}=\left(\begin{array}{llll} 1 & 0 & 0 & 0\\ 0 & 1 & 1 & 0\\ 0 & 0 & 0 & 1 \end{array}\right)n_{s}^{⊗2}$. We define *wombling measures* as the total and average differential over 𝒞,

(5) $$𝚪(𝒞)=\;\iint_{𝒞}\;\;{𝓛}_{n_{t},n_{s}}^{*}Z(s(\omega ,v),v)\|\bar{n}(s(\omega ,v),v)\|d\omega \;dv\;\text{and}\;\bar{𝚪}(𝒞)=\frac{𝚪(𝒞)}{𝒜(𝒞)},$$

respectively, where the double integral acts element-wise. Therefore, 𝚪 and $\bar{𝚪}$ are 8 × 1 vectors. A large value for any entry of 𝚪(𝒞) (or $\bar{𝚪}(𝒞)$) indicates that 𝒞 tracks a zone of rapid spatiotemporal change. More generally, ${𝓛}_{n_{t},n_{s}}^{*}Z(s,t)$ can be replaced with, $g\left\{{𝓛}^{*}Z(s,t)\right\}$, where g(⋅) is any linear functional. Section S7 offers more details on parameterized surfaces.

Specifically, we are interested in surfaces with a boundary (in the topological sense). In the subsequent examples, the boundary for 𝒞 are open or closed parametric planar curves, $C_{v_{i}}=\left\{\left(s_{x}\left(\omega ,v_{i}\right),s_{y}\left(\omega ,v_{i}\right)\right)\;:\;\omega \in \left[\omega_{0},\omega_{1}\right]\right\},i=0,1$. If, in particular, $C_{v_{0}}=C_{v_{1}}=C_{v}=C$, for every $v\in \left[v_{0},v_{1}\right]$, the curve stays fixed over time so $s_{x}$ and $s_{y}$ are functions of ω only. From (4), $𝒜(𝒞)=\left(v_{1}-v_{0}\right)\int_{\omega_{0}}^{\omega_{1}}\;{\left\{{\left(\frac{\partial s_{y}(\omega )}{\partial \omega }\right)}^{2}+{\left(\frac{\partial s_{x}(\omega )}{\partial \omega }\right)}^{2}\right\}}^{\frac{1}{2}}d\omega =\left(v_{1}-v_{0}\right)\ell (C)$, where ℓ(C) is the arc-length of the parametric curve $C=\left\{\left(s_{x}(\omega ),s_{y}(\omega )\right)\;:\;\omega \in \left[\omega_{0},\omega_{1}\right]\right\}$.

### 3.2. Curves on Surfaces

Curves of interest on 𝒞 are *planar curves*. They mark spatial regions of rapid change at time, $v=v^{\prime }$. Let $C_{v^{\prime }}=\left\{\left(s_{x}\left(\omega ,v^{\prime }\right),s_{y}\left(\omega ,v^{\prime }\right)\right)\;:\;\omega \in \left[\omega_{0},\omega_{1}\right],\omega_{0}\neq \omega_{1}\right\}$ denote an open parametric planar curve. The wombling measures in (5) are defined with respect to the normal, $n_{s}\left(\omega ,v^{\prime }\right)$, to $C_{v^{\prime }}$. One could, in principle, consider defining wombling measures with respect to the tangential direction to $C_{v^{\prime }}$. The tangent is $\bar{u}\left(\omega ,v^{\prime }\right)={\left(\frac{\partial s_{x}\left(\omega ,v^{\prime }\right)}{\partial \omega },\frac{\partial s_{y}\left(\omega ,v^{\prime }\right)}{\partial \omega }\right)}^{\text{T}}$, with corresponding unit tangent, $u\left(\omega ,v^{\prime }\right)=\frac{\bar{u}\left(\omega ,v^{\prime }\right)}{\left\|\bar{u}\left(\omega ,v^{\prime }\right)\right\|}$. However, wombling boundaries are, in general, representative of geographic features that delineate zones of rapid change. This suggests that such boundaries should be identified based upon rates of change orthogonal, and not tangential, to the curve. Furthermore, replacing normals with tangents in (5) for closed curves yields the value of 0 in each element of 𝚪(𝒞). For example, if $C_{v^{\prime }}$ is an administrative or natural boundary of a closed region (e.g., zip-code, county, boundary of water body), then the corresponding wombling measures along the tangential direction will be zero and, hence, lack interpretation. On the other hand, the wombling measures defined with normals, as in (5), for closed curves correspond to the notion of *spatiotemporal flux* associated with the enclosed region (see, e.g., Banerjee and Gelfand 2006, for spatial flux).

In particular, if for every $v\in \left[v_{0},v_{1}\right],C_{v}$ is a closed curve, then the surface 𝒞 encloses a topologically compact region W with a *piece-wise smooth boundary* ∂W. We denote the integral over the boundary by $\int_{\partial W}\;$. The total wombling measure is $𝚪(𝒞)=\int_{\partial W}\;{𝓛}_{n_{t},n_{s}}^{*}Z(s,t)\;\text{d}𝒜$. For such volumes the spatiotemporal flux and rate of change in spatiotemporal flux represent rates of change in the spatiotemporal process enclosed by W. If Z(s,t) is three times differentiable, then the closed surface integral can be expressed as a volume integral over W without requiring explicit parametric representation. Let F1(s,t)=∂sZ(s,t)∂tZ(s,t) and $F_{2}(s,t)=\left(\begin{array}{c} F_{21}\\ \vdots \\ F_{26} \end{array}\right)=\nabla^{⊗2}Z(s,t)$. The spatiotemporal flux and the rate of change in spatiotemporal flux satisfy the identities, $\int_{𝒞=\partial W}\;n(s,t{)}^{\text{T}}F_{1}(s,t)\;\text{d}𝒜={∭}_{W}\;\text{div}\;F_{1}\;\text{d}W$ and $\int_{𝒞=\partial W}\;n(s,t{)}^{⊗2^{\text{T}}}F_{2}(s,t)\;\text{d}𝒜\;={∭}_{W}\;\left\{\text{div}\;n_{s_{x}}\left(\begin{array}{l} F_{21}\\ F_{22}\\ F_{23} \end{array}\right)+\text{div}\;n_{s_{y}}\left(\begin{array}{l} F_{22}\\ F_{24}\\ F_{25} \end{array}\right)+\text{div}\;n_{t}\left(\begin{array}{l} F_{23}\\ F_{25}\\ F_{26} \end{array}\right)\right\}\;\text{d}W$, where $n(s,t)=\left(\begin{array}{c} n_{s_{x}}\\ n_{sy}\\ n_{t} \end{array}\right)$ and $\text{d}W=ds_{x}\;ds_{y\;}dt$ is the volume element. The integrand on the right consists of elements from $\nabla^{⊗3}Z(s,t)$. The identities are obtained using the Divergence Theorem due to Gauss (see, e.g., Spivak 1979, vol. 4, chap. 7, p. 192). The first identity concerning spatiotemporal flux requires Z(s,t) to be twice differentiable (see Section S8).

Turning to practical computation of the spatiotemporal flux (or its rate of change) ${∭}_{W}\;$ can be evaluated using a customary Riemann approximation (with cubes inside the region W), which, while simple in concept, can be demanding on resources and requires smoother process specifications. On the other hand, evaluating $\int_{𝒞=\partial W}\;$ is conceptually harder and benefits from the triangulation procedure discussed in Section 4.2. Finally, we remark that one could possibly use Stokes’ theorem on surfaces $𝒞=\left\{\left(s_{x}(\omega (v)),s_{y}(\omega (v)),v\right)\;:\;v\in {𝒟}_{v}\right\}$. This is a parameterization from $R^{+}\to R^{3}$. Wombling measures would then be line integrals defined on curves bounding a surface ${\oint \;}_{C}n(s,t{)}^{\text{T}}F_{1}(s,t)\;\text{d}\ell =\iint_{𝒞}\;$ curl $F_{1}\;\text{d}𝒜$. These would then be defined on curves and be incongruous with our aim of associating them with surfaces. We do not pursue such developments here.

### 3.3. Spatiotemporal Wombling

Our scientific applications include, for example, temporally evolving wombling measures for neural activity over regions of the human scalp. We consider temporally indexed curves, say $C_{t}$, which generate surfaces. For example, a line segment is described as $C_{t}=\left\{s_{t}+\omega u_{t}\;:\;\omega \in [0,1]\right\}$ on $t\in \left[t_{0},t_{1}\right]$. We write $C_{0}=\left\{s_{0}+\omega u\;:\;\omega \in [0,1]\right\}$ and $C_{1}=\left\{s_{0}+v+\omega (w-v)\;:\;\omega \in \right.[0,1]\}$ for $t=t_{0}$ and $t=t_{1}$, respectively, where $s_{t_{0}}=s_{0},u_{t_{0}}=u,\;v=s_{t_{1}}-s_{0}$ is the displacement vector for $s_{0}$ and $w=u_{t_{1}}+v$ is the corresponding displacement of $u_{t_{1}}$. Hence, $C_{t_{0}}$ evolves continuously to $C_{t_{1}}$ generating planes from two sets of non-collinear points, s0t0,s0+ut0,s0+vt1 and s0+ut0,s0+vt1,s0+wt1. The resulting surface, 𝒞, is a union of two triangular planes given by 𝒞0=s0t0+ωu0+vv1:(ω,v)∈T and 𝒞1=s0+wt1+ωv-w0+vu-w-1:(ω,v)∈T, where T={(ω,v):ω,v∈[0,1],ω+v≤1} is a triangular region in $R^{2}$ (left panel of Figure 1). The unit normals (independent of (ω,v)) are $n_{i}=\frac{{\bar{n}}_{i}}{\left\|{\bar{n}}_{i}\right\|}$ for i=0,1, where n¯0=v1×u0=n¯s,0n‾t,0 and n¯1=v-w0×w-u1=n‾s,1n‾t,1, respectively, and × is the vector product. The areas are $𝒜\left({𝒞}_{i}\right)=\iint_{T}\;\left\|{\bar{n}}_{i}\right\|d\omega \;dv=\frac{\left\|{\bar{n}}_{i}\right\|}{2}$, and wombling measures in (5) are $𝚪\left({𝒞}_{i}\right)=\left\|{\bar{n}}_{i}\right\|\iint_{T}\;{ℒ}_{n_{t,i},n_{s,i}}^{*}Z(s(\omega ,v),v)\;d\omega \;dv$, for i=0,1. The total and average wombling measures over the plane $𝒞={𝒞}_{0}\cup {𝒞}_{1}$ with area $𝒜(𝒞)=𝒜\left({𝒞}_{0}\right)+𝒜\left({𝒞}_{1}\right)$ are $𝚪(𝒞)=𝚪\left({𝒞}_{0}\right)+𝚪\left({𝒞}_{1}\right)$ and $\bar{𝚪}(𝒞)=\frac{𝚪(𝒞)}{𝒜(𝒞)}$, respectively.

The right panel of Figure 1 displays another example: a surface generated from arcs of parametric semi-circles, $𝒞=\left\{(rv\;\text{cos}\;\omega ,rv\;\text{sin}\;\omega ,v):\omega \in [0,\pi ],v\in \left[t_{0},t_{0}+1\right],r>0\right\}$. The radii of the arcs increase linearly with time. The normal to 𝒞 is $\bar{n}(s(\omega ,v),v)={\left(rv\;\text{cos}\;\omega ,rv\;\text{sin}\;\omega ,-r^{2}v\right)}^{\text{T}}$. The unit normal is $n(s(\omega ,v),v)=\frac{1}{\sqrt{1+r^{2}}}(\text{cos}\;\omega ,\text{sin}\;\omega ,-r{)}^{\text{T}}$ and the area is $(𝒞)=\int_{t_{0}}^{t_{0}+1}\;\int_{0}^{\pi }\;\|\bar{n}(s(\omega ,v),v)\|d\omega \;dv=\left(t_{0}+\frac{1}{2}\right)\pi r\sqrt{1+r^{2}}$. The average wombling measure is $\bar{𝚪}(𝒞)=\frac{1}{\left(t_{0}+\frac{1}{2}\right)\pi }\int_{t_{0}}^{t_{0}+1}\;\int_{0}^{\pi }\;{𝓛}_{n_{t}(v),n_{s}(\omega ,v)}^{*}Z(s(\omega ,v),v)\;v\;d\omega \;dv$. Section S14 discusses static wombling, where the curve stays fixed over time.

### 3.4. Distribution Theory for Wombling Measures

We extend the distribution theory in Section 2 to derive the joint distribution for the N×1 process 𝓩 and the wombling measures 𝚪(𝒞) in (5) for conducting predictive inference. Suppose 𝒞 is generated over intervals, ${𝒟}_{v_{0}}=\left[0,v_{0}\right]$ and ${𝒟}_{\omega_{0}}=\left[0,\omega_{0}\right]$. For any $u^{*}\in {𝒟}_{v_{0}}$ and $\omega^{*}\in {𝒟}_{\omega_{0}}$, let ${𝒞}_{*}$ denote the surface restricted to ${𝒟}_{*}={𝒟}_{v^{*}}\times {𝒟}_{\omega^{*}}=\left[0,v^{*}\right]\times \left[0,\omega^{*}\right]$. The wombling measures in (5) are $𝚪\left({𝒞}_{*}\right)=\iint_{{𝒟}_{*}}\;{𝓛}_{n_{t},n_{s}}^{*}Z(s(\omega ,v),v)\|\bar{n}(s(\omega ,v),v)\|d\omega \;dv$. The directional differential processes, ${𝓛}_{n_{t},n_{s}}^{*}Z(s,t)$, are valid zero-mean Gaussian processes (GPs). Hence, $𝚪\left({𝒞}_{*}\right)\sim {𝒩}_{8}\left(0_{8},K_{𝚪}\left({𝒞}_{*},{𝒞}_{*}\right)\right)$, where

(6) $$K_{\Gamma }\left({𝒞}_{*},{𝒞}_{*}\right)=\underset{{𝒟}_{*}\times {𝒟}_{*}}{\int \cdots \int }N_{st}V_{{𝓛}^{*}Z}\left(𝚫\left(\omega_{1},\omega_{2},v_{1},v_{2}\right),\delta \left(v_{1},v_{2}\right)\right)N_{st}^{\text{T}}.\;\left\|\bar{n}\left(s\left(\omega_{1},v_{1}\right),v_{1}\right)\right\|\cdot \left\|\bar{n}\left(s\left(\omega_{2},v_{2}\right),v_{2}\right)\right\|\;d\omega_{1}dv_{1}d\omega_{2}dv_{2},$$

$𝚫\left(\omega_{1},\omega_{2},v_{1},v_{2}\right)=s\left(\omega_{2},v_{2}\right)-s\left(\omega_{1},v_{1}\right)$ and $\left(v_{1},v_{2}\right)=v_{2}-v_{1}$. The repeated integral acts element-wise on matrices. $𝚪\left({𝒞}_{*}\right)$ is a *dependent GP* on ${𝒟}_{v_{0}}\times {𝒟}_{\omega_{0}}$—for any two points, $\left(\omega_{1}^{*},v_{1}^{*}\right)$ and $\left(\omega_{2}^{*},v_{2}^{*}\right)$, the dependence is specified through, 𝚪𝒞1*𝚪𝒞2*~𝒩16016,K𝚪𝒞1*,𝒞1*K𝚪𝒞1*,𝒞2*K𝚪𝒞2*,𝒞1*K𝚪𝒞2*,𝒞2*, where $𝚪\left({𝒞}_{i*}\right)$ is the wombling measure computed over $\left[0,\omega_{i}^{*}\right]\times \left[0,v_{i}^{*}\right]$ and each $K_{𝚪}\left({𝒞}_{i*},{𝒞}_{j*}\right)$ is 8 × 8 obtained from (6) for $i,j=1,2.\;𝚪\left({𝒞}_{*}\right)$ is mean-square continuous. Although Z(s,t) is stationary, $𝚪\left({𝒞}_{*}\right)$ need not be. Let $G\left({𝒞}_{*}\right)=\text{cov}\left\{𝓩,𝚪\left({𝒞}_{*}\right)\right\}=\left(\begin{array}{c} \gamma_{1}{\left({𝒞}_{*}\right)}^{\text{T}}\\ \vdots \\ \gamma_{N}{\left({𝒞}_{*}\right)}^{\text{T}} \end{array}\right)$ as the N×8 matrix, where $\gamma_{i}\left({𝒞}_{*}\right)=\text{cov}\left(Z\left(s_{i},t_{i}\right),𝚪\left({𝒞}_{*}\right)\right),\;{𝚫}_{i}(\omega ,v)=s_{i}-s(\omega ,v),\delta_{i}(v)=t_{i}-v$,

(7) $$\gamma_{i}\left({𝒞}_{*}\right)=\;\iint_{{𝒟}_{*}}\;\;N_{st}{𝓚}_{i}\left({𝚫}_{i}(\omega ,v),\delta_{i}(v)\right)\times \|\bar{n}(s(\omega ,v),v)\|d\omega \;dv,\;i=1,\ldots ,N,$$

and the integrals act element-wise on the vector. The desired distribution $P\left[𝓩,𝚪\left({𝒞}_{*}\right)\right]$ is

(8) 𝓩𝚪𝒞*~𝒩N+80N+8,∑𝓩G𝒞*GT𝒞*K𝚪𝒞*,𝒞*.

## 4. Bayesian Inference and Computation

### 4.1. Spatiotemporal Hierarchical Model

Let $𝓨={\left(y\left(s_{1},t_{1}\right),\ldots ,y\left(s_{N},t_{N}\right)\right)}^{\text{T}}$ be the N×1 vector of observations over spatiotemporal coordinates $\left\{\left(s_{1},t_{1}\right),\ldots ,\left(s_{N},t_{N}\right)\right\}$. We construct the spatiotemporal hierarchical model,

(9) $$y\left(s_{i},t_{i}\right)=\mu \left(s_{i},t_{i}\right)+Z\left(s_{i},t_{i}\right)+\epsilon \left(s_{i},t_{i}\right),$$

where $\mu \left(s_{i},t_{i}\right)=x{\left(s_{i},t_{i}\right)}^{\text{T}}\beta$, each $x\left(s_{i},t_{i}\right)$ is a p×1 vector of spatiotemporal covariates, $Z\left(s_{i},t_{i}\right)\text{s}$ are realizations of the process Z(s,t)~GP(0,K(⋅,⋅,θ)) and $\epsilon \left(s_{i},t_{i}\right)\overset{\text{iid}}{\sim }N\left(0,\tau^{2}\right)$ accounts for white noise. The process parameters are $\Theta =\left\{\theta ,\tau^{2},\beta \right\}$, where $\theta =\left\{\sigma^{2},\phi_{s},\phi_{t}\right\}$. With customary prior specifications we obtain the posterior,

(10) $$P(\Theta |𝓨)\propto \pi \left(\theta ,\tau^{2}\right)\times 𝒩\left(\beta |\mu_{\beta },\sum_{\beta }\right)\times {𝒩}_{N}\left(𝓨|X(s,t)\beta ,\sum_{𝓩}+\tau^{2}I_{N}\right),$$

where $\pi \left(\theta ,\tau^{2}\right)=U\left(\phi_{s}|\;a_{\phi_{s}},b_{\phi_{s}}\right)\times U\left(\phi_{t}|\;a_{\phi_{t}},b_{\phi_{t}}\right)\times IG\left(\sigma^{2}|a_{\sigma },b_{\sigma }\right)\times IG\left(\tau^{2}|a_{\tau },b_{\tau }\right),\sum_{𝓩}=\sigma^{2}R\left(\phi_{s},\phi_{t}\right)$ is the N×N spatiotemporal covariance matrix constructed from the Matérn covariance kernel in (1), IG denotes the inverse-gamma distribution, 𝒩 denotes univariate Gaussian and ${𝒩}_{N}$ denotes N-variate Gaussian distribution.

We compute P(Θ∣𝓨) by marginalizing out 𝓩, thereby producing a “collapsed”, more efficient alternative. In a more general setting, if learning smoothness of the process from available data is of interest, then $v\sim U\left(a_{v},b_{v}\right)$. Depending on the posterior estimate for ν, the spatiotemporal differential processes are sampled using closed-form Matérn kernels with half-integer values for ν as follows, $\nu =1+\frac{1}{2}$ or, $2+\frac{1}{2}$. For instance, if 2≤ν≤3, then a Matérn with $\nu =\frac{5}{2}$ is used to generate posterior samples for the differential processes. This features in our implementation. Alternatively, models can be fit simultaneously with $v=\frac{1}{2},\frac{3}{2}$ and $\frac{5}{2}$ and a model selection criteria can be used to select v for the best fit. The posterior distribution in (10) is sampled using a combination of Gibbs and Metropolis-Hastings (MH) steps. We first sample θ and $\tau^{2}$ using MH steps, followed by Gibbs samples for β and 𝓩, one-for-one corresponding to samples of θ and $\tau^{2}$.

Predictive inference on spatiotemporal differential processes and wombling measures for the latent surface is achieved by sampling from $P\left({𝓛}^{*}Z\left(s_{0},t_{0}\right)|𝓨\right)$ and P(𝚪(𝒞)∣𝓨), respectively, where 𝒞 is a surface of interest. The posterior predictive distribution of the derivative processes is $\left({𝓛}^{*}Z\left(s_{0},t_{0}\right)|𝓨\right)=\int \;P\left({𝓛}^{*}Z\left(s_{0},t_{0}\right)|𝓩,\Theta \right)\;P(𝓩|\;𝓨,\Theta )\;P(\Theta |\;𝓨)\;d\Theta$. Posterior inference for any sub-vector, say, $\partial_{t}^{2}{\tilde{\partial_{s}}}^{2}Z\left(s_{0},t_{0}\right)$ is obtained as $\left(\partial_{t}^{2}{\tilde{\partial_{s}}}^{2}Z\left(s_{0},t_{0}\right)|𝓨\right)\;=\;\int \cdots \int P\left(\partial_{t}^{2}{\tilde{\partial }}_{s}^{2}Z\left(s_{0},t_{0}\right)|{\tilde{𝓛}}^{*}Z\left(s_{0},t_{0}\right),𝓩,\Theta \right)P\left({\tilde{𝓛}}^{*}Z\left(s_{0},t_{0}\right)|𝓩,\Theta \right)P(𝓩|𝓨,\Theta )P(\Theta |𝒴)d\Theta \;d{\tilde{𝓛}}^{*}Z$, where ${\tilde{𝓛}}^{*}Z$ is the vector of spatiotemporal derivative processes excluding $\partial_{t}^{2}{\tilde{\partial }}_{s}^{2}Z\left(s_{0},t_{0}\right)$. Posterior sampling from the above conditionals is done one-for-one using (2). One instance of ${𝓛}^{*}Z\left(s_{0},t_{0}\right)$ is sampled for each posterior sample of θ obtained from the posterior, P(θ∣𝓩). The conditional predictive distribution of the spatiotemporal differential process, ${𝓛}^{*}Z\left(s_{0},t_{0}\right)|𝒵,\theta \sim {𝒩}_{m-1}\left(\mu_{𝓛},\sum_{𝓛}\right)$, where $\mu_{𝓛}=-{𝓚}_{0}\sum_{𝓩}^{-1}𝓩$ and $\sum_{𝓛}=V_{{𝓛}^{*}Z}\left(0_{d},0\right)-{𝓚}_{0}\sum_{𝓩}^{-1}{{𝓚}_{0}}^{\text{T}}$. Similar arguments follow for any element or sub-vector of ${𝓛}^{*}Z\left(s_{0},t_{0}\right)$.

Posterior samples for the wombling measures are obtained similarly. The posterior, P(𝚪(𝒞)∣𝓨)=∫P(𝚪(𝒞)∣𝓩,Θ)P(𝓩∣𝓨,Θ)P(Θ∣𝓨)dΘd𝓩 is used to sample wombling measures. For each posterior sample of 𝓩 and θ, we draw $𝚪(𝒞)|𝓩,\theta \sim {𝒩}_{8}\left(-G(𝒞{)}^{\text{T}}\sum_{𝓩}^{-1}𝓩,K_{𝚪}(𝒞,𝒞)-\right.\left.G(𝒞{)}^{\text{T}}\sum_{𝓩}^{-1}G(𝒞)\right)$, where, $K_{𝚪}(𝒞,𝒞),G(𝒞)$ are computed using (6) and (7) using an appropriate parameterization (see Section 4.2). Posterior samples for $𝓩,{𝓛}^{*}Z$ and 𝚪(𝒞) are generated together to avoid repeated computation of $\sum_{𝓩}^{-1}$. We leverage parallel computation to improve algorithmic complexity.

### 4.2. Triangulation of Surfaces

For a surface (compact and connected in the topological sense) to be identified as a wombling boundary, posterior sampling of wombling measures requires a parametric form. We choose to perform spatiotemporal wombling using simple parametric regions, specifically triangular planes as shown in Figure 1 (left). The spatiotemporal wombling measures in (5) are computed on each region followed by an aggregation to produce the wombling measure for the surface. The surface 𝒞 is triangulated using a partition. We partition ${𝒟}_{\omega }=\left[\omega_{0},\omega_{1}\right]$ using $\omega_{0}=\omega_{1}^{\prime }<\cdots <\omega_{n_{\omega }}^{\prime }=\omega_{1}$ and ${𝒟}_{v}=\left[t_{j},t_{j+1}\right]$ using $t_{j}=v_{0}=v_{1}^{\prime }<\cdots <v_{n_{v}}^{\prime }=v_{1}=t_{j+1}$. Denoting the partition as 𝒫, its norm is $|𝒫{|}_{A}=\underset{\begin{array}{c} 1\leq i\leq n_{\omega }-1\\ 1\leq j\leq n_{v}-1 \end{array}}{\text{max}}\;\left(\omega_{i+1}^{\prime }-\omega_{i}^{\prime }\right)\left(v_{j+1}^{\prime }-v_{j}^{\prime }\right)$, which is a function of the area of the largest parallelogram. Let $C_{t_{j}}$ be a planar curve on 𝒞. The curves have a polygonal approximation ${\tilde{C}}_{t_{j}}=\overset{n_{\omega }-1}{\underset{i=1}{⋃}}\;{\tilde{C}}_{ij}$ with ${\tilde{C}}_{ij}=\left\{s\left(\omega_{i}^{\prime },t_{j}\right)+\omega u_{ij}\right.\;:\;\left.u_{ij}=s\left(\omega_{i+1}^{\prime },t_{j}\right)-s\left(\omega_{i}^{\prime },t_{j}\right),\omega \in [0,1]\right\}$. Considering ${\tilde{C}}_{ij}$ and ${\tilde{C}}_{ij+1}$, which are rectilinear segments at adjacent time points $t_{j}$ and $t_{j+1}$, respectively. The triangulation of 𝒞 restricted to the rectangle $\left[\omega_{i}^{\prime },\omega_{i+1}^{\prime }\right]\times \left[t_{j},t_{j+1}\right]$ follows the first example in Section 3.3. The triangular planes are generated using the set, sωi′,tjtj,sωi+1′,tjtj,sωi′,tj+1tj+1,sωi+1′,tj+1tj+1, of four points which are not co-planar. They are 𝒞ij1=sωi′,tjtj+ωuij0+vvij+1tj+1-tj:(ω,v)∈T and 𝒞ij2=sωi+1′,tj+1tj+1+ωuij+10+vvi+1jtj-tj+1:(ω,v)∈T, where, $v_{ij+1}=s\left(\omega_{i}^{\prime },t_{j+1}\right)-s\left(\omega_{i}^{\prime },t_{j}\right),v_{i+1j}=s\left(\omega_{i+1}^{\prime },t_{j}\right)-s\left(\omega_{i+1}^{\prime },t_{j+1}\right)$ and T is a parametric triangle in $R^{2}$ (see Section 3.3).

The normals to the planes are n¯ij1=vij+1tj+1-tj×uij0 and n¯ij2=-uij+10×-vi+1jtj+1-tj, respectively, where × is the vector product. These quantities are free of (ω,v). The corresponding unit normals are nijk=n¯ijkn¯ijk=nijk,snijk,t,k=1,2. Iteration over successive intervals triangulates 𝒞 as $\tilde{𝒞}=\overset{n_{\omega }-1}{\underset{i=1}{⋃}}\;\overset{n_{v}-1}{\underset{j=1}{⋃}}\;\overset{2}{\underset{k=1}{⋃}}\;{𝒞}_{ijk}$. Figure 2 illustrates this procedure. The wombling measure in (5) is computed on ${𝒞}_{ijk}$ as, $\left({𝒞}_{ijk}\right)=\iint_{T}\;N_{ijk,st}{𝓛}^{*}Z(s(\omega ,v),v)\;\left\|{\bar{n}}_{ijk}\right\|\;d\omega \;dv\;=\iint_{T}\;{𝓛}_{n_{ijk,t},n_{ijk,s}}^{*}Z(s(\omega ,v),v)\left\|{\bar{n}}_{ijk}\right\|\;d\omega \;dv$, for k=1,2. Aggregating, we obtain, $𝚪(\tilde{𝒞})=\sum_{i=1}^{n_{\omega }-1}\;\sum_{j=1}^{n_{v}-1}\;\sum_{k=1}^{2}\;𝚪\left({𝒞}_{ijk}\right)$. As $\left|𝒫{|}_{A}\to 0,\;𝚪(\tilde{𝒞})\overset{\text{a.s.}}{\to }𝚪(𝒞\right)$. Hence, $𝚪(\tilde{𝒞})$ is a strongly consistent estimator for 𝚪(𝒞). In general, if we consider $g\left({𝓛}_{n_{t},n_{s}}^{*}Z(s,t)\right)$, where g(⋅) is uniformly continuous over $\left[\omega_{0},\omega_{1}\right]\times \left[t_{1},t_{N}\right]$, then the corresponding estimator, ${𝚪}_{g}(\tilde{𝒞})$, converges almost surely to ${𝚪}_{g}(𝒞)$ (see Theorem 2 in Section S9).

Posterior samples of $𝚪\left({𝒞}_{ijk}\right)$ are obtained from (8). From (6), $K_{𝚪}\left({𝒞}_{ijk},{𝒞}_{ijk}\right){=\;}_{T\times T}^{\int \;\cdots \int \;}N_{ijk,st}\;V_{{𝓛}^{*}Z}\left({𝚫}_{k}\left(\omega_{1},\omega_{2},v_{1},v_{2}\right)\right.,\left.\delta_{k}\left(v_{1},v_{2}\right)\right)\;N_{ijk,st}^{\text{T}}\;{\left\|{\bar{n}}_{ijk}\right\|}^{2}\;d\omega_{1}\;dv_{1}\;d\omega_{2}\;dv_{2}$, with $\delta_{1}\left(v_{2},v_{1}\right)=\left(v_{2}-v_{1}\right)\left(v_{j+1}^{\prime }-v_{j}^{\prime }\right),\delta_{2}\left(v_{2},v_{1}\right)=-\delta_{1}\left(v_{2},v_{1}\right),{𝚫}_{1}\left(\omega_{1},\omega_{2},v_{1},v_{2}\right)=\left(\omega_{2}-\omega_{1}\right)u_{ij}+\left(v_{2}-v_{1}\right)v_{ij+1}$ and ${𝚫}_{2}\left(\omega_{1},\omega_{2},v_{1},v_{2}\right)=\left(\omega_{2}-\omega_{1}\right)u_{ij+1}+\left(v_{2}-v_{1}\right)v_{ij}$. From (7), $G\left({𝒞}_{ijk}\right)=\left(\begin{array}{c} \gamma_{1}{\left({𝒞}_{ijk}\right)}^{\text{T}}\\ \vdots \\ \gamma_{N}{\left({𝒞}_{ijk}\right)}^{\text{T}} \end{array}\right)$, where for any $\left(s_{i^{\prime }},t_{i^{\prime }}\right),\;\gamma_{i^{\prime }}\left({𝒞}_{ijk}\right)=\iint_{T}\;N_{ijk,st}{𝓚}_{i^{\prime }}\left({𝚫}_{i^{\prime }k}(\omega ,v),\delta_{i^{\prime }k}(v)\right)\;\left\|{\bar{n}}_{ijk}\right\|\;d\omega \;dv$, where ${𝚫}_{i^{\prime }1}(\omega ,v)={𝚫}_{i^{\prime }ij}-\omega u_{ij}-vv_{ij+1},{𝚫}_{i^{\prime }2}(\omega ,v)={𝚫}_{i^{\prime }i+1j+1}-\omega u_{ij+1}-vv_{ij},\delta_{i^{\prime }1}(v)=\delta_{i^{\prime }j}-v\delta_{j+1j}$ and $\delta_{i^{\prime }2}(v)=\delta_{i^{\prime }j+1}+v\delta_{j+1j}$ for $i^{\prime }=1,\ldots ,N$, where ${𝚫}_{i^{\prime }ij}=s_{i^{\prime }}-s\left(\omega_{i}^{\prime },t_{j}\right)$ and $\delta_{i^{\prime }j}=t_{i^{\prime }}-t_{j}$. Analytically evaluating $K_{𝚪}\left({𝒞}_{ijk},{𝒞}_{ijk}\right)$ using high-dimensional quadrature is tedious.

### 4.3. Cross-covariance of Wombling Measures

Our choice of simple parametric planes allows us to avoid high dimensional quadrature in (6) required for posterior sampling of wombling measures. In the integrand for $K_{𝚪}\left({𝒞}_{ijk},{𝒞}_{ijk}\right),{𝚫}_{k}\left(\omega_{1},\omega_{2},v_{1},v_{2}\right)={𝚫}_{k}\left(\omega_{2}-\omega_{1},v_{2}-v_{1}\right),\;\delta_{k}\left(v_{1},v_{2}\right)=\delta_{k}\left(v_{2}-v_{1}\right),k=1,2$ are functions of $\omega_{2}-\omega_{1}$ and $v_{2}-v_{1}$. A simple change of variable results in $K_{𝚪}\left({𝒞}_{ijk},{𝒞}_{ijk}\right)=\frac{{\left\|{\bar{n}}_{ijk}\right\|}^{2}}{2}\int_{-1}^{1}\;\int_{-1-x_{v}}^{1-x_{v}}\;N_{ijk,st}V_{{𝓛}^{*}Z}\left({𝚫}_{k}\left(x_{\omega },x_{v}\right),\delta_{k}\left(x_{v}\right)\right)N_{ijk,st}^{\text{T}}dx_{\omega }dx_{v}$, where $x_{\omega }=\omega_{2}-\omega_{1}$ and $x_{v}=v_{2}-v_{1}$. This reduces a four-dimensional integral to a double integral. Bivariate quadrature is performed to obtain the cross-covariance. Similar changes can be effected in the purely spatial case—for the Gaussian kernel we have closed forms available (see Section S10).

## 5. Simulation Experiments

We devise simulation experiments to calibrate inference for spatiotemporal differential processes and wombling measures. Algorithms for posterior sampling of the differential processes and the wombling measures are available in Section S11. The required sub-routines are developed for implementation in the R-statistical environment (R Core Team 2021). The experiments are performed on a Linux OS powered by a 12th Generation Intel^®^ Core^™^ i9–12.9K CPU with 24 cores and 64GB of RAM. Vignettes are available in the GitHub repository https://github.com/arh926/sptwombling.

### Data generation.

Gradients and curvatures in realizations are not observed, but are induced by a parent process. We set up simulation experiments with known true values of differential processes and wombling measures. We consider locations $\left(s_{x},s_{y},t\right)\in [0,1]\times [0,1]\times 𝒯\subseteq R^{2}\times R^{+}$ over the unit square across nine time points, 𝒯={1,2,…,9}. We generate synthetic data from four distributions, $y(s,t)\sim 𝒩\left(\mu_{i}(s,t),\tau^{2}\right),i=1,\ldots ,4$ and $\tau^{2}=1$ using the following specifications: (a) Pattern 1: $\mu_{1}=10\left\{\text{sin}\;\left(3\pi s_{x}\right)+\text{cos}\;\left(3\pi s_{y}\right)\;\text{cos}\;\left(\frac{\pi t}{7}\right)\right\}$; (b) Pattern 2: $\mu_{2}=10\;\text{sin}\;\left(3\pi s_{x}\right)\;\text{cos}\;\left(3\pi s_{y}\right)\;\text{cos}\;\left(\frac{\pi t}{7}\right)$. Figure 3(a) and (b) present interpolated plots for the spatiotemporal data generated from these patterns, respectively. These data generating patterns are motivated by smooth spatiotemporal data that do not arise from (10), where the true spatiotemporal differential processes and wombling measures are computed in closed form. This allows us to calibrate the proposed inferential framework. The patterns also represent scenarios that manifest in real-time data. The pattern in Figure 3(a) displays regions enclosing peaks and troughs that relocate spatially over time. The pattern in Figure 3(b) displays regions enclosing peaks (troughs) that change to troughs (peaks) over time. Pattern 1 has a zero mixed spatial derivative—changes in space along the x-axis does not affect changes along y-axis, while Pattern 2 has all nonzero derivatives.

The true values for the spatiotemporal differential processes induced by the patterns are as follows. We list the spatial gradients and curvature followed by their temporal gradients and curvature. $\partial_{s}\mu_{1}(s,t)=30\pi {\left(\text{cos}\;\left(3\pi s_{x}\right),-\text{sin}\;\left(3\pi s_{y}\right)\;\text{cos}\;\left(\frac{\pi t}{7}\right)\right)}^{\text{T}}$ and $\partial_{s}^{2}\mu_{1}(s,t)=-90\pi^{2}\text{diag}\left\{\text{sin}\;\left(3\pi s_{x}\right),\text{cos}\;\left(3\pi s_{y}\right)\;\text{cos}\;\left(\frac{\pi t}{7}\right)\right\}$. The true temporal derivatives are $\partial_{t}\mu_{1}(s,t)=-\frac{10\pi }{7}\text{cos}\;\left(3\pi s_{y}\right)\;\text{sin}\;\left(\frac{\pi t}{7}\right),\partial_{t}\partial_{s_{x}}\mu_{1}(s,t)=0,\partial_{t}\partial_{s_{y}}\mu_{1}(s,t)=\frac{30\pi^{2}}{7}\text{sin}\;\left(3\pi s_{y}\right)\;\text{sin}\;\left(\frac{\pi t}{7}\right),\partial_{t}\partial_{s_{x}}^{2}\mu_{1}(s,t)=\partial_{t}\partial_{s_{x}s_{y}}^{2}\mu_{1}(s,t)=0$ and $\partial_{t}\partial_{s_{y}}^{2}\mu_{1}(s,t)=\frac{90\pi^{3}}{7}\text{cos}\;\left(3\pi s_{y}\right)\;\text{sin}\;\left(\frac{\pi t}{7}\right)$. The true temporal curvatures are $\partial_{t}^{2}\mu_{1}(s,t)=-\frac{10\pi^{2}}{49}\text{cos}\;\left(3\pi s_{y}\right)\;\text{cos}\;\left(\frac{\pi t}{7}\right),\partial_{t}^{2}\partial_{s_{x}}\mu_{1}(s,t)=0,\partial_{t}^{2}\partial_{s_{y}}\mu_{1}(s,t)=\frac{30\pi^{3}}{49}\text{sin}\;\left(3\pi s_{y}\right)\;\text{cos}\;\left(\frac{\pi t}{7}\right),\partial_{t}^{2}\partial_{s_{x}}^{2}\mu_{1}(s,t)=\partial_{t}^{2}\partial_{s_{x}s_{y}}^{2}\mu_{1}(s,t)=0,\partial_{t}^{2}\partial_{s_{y}}^{2}\mu_{1}(s,t)=\frac{90\pi^{4}}{49}\text{cos}\;\left(3\pi s_{y}\right)\;\text{cos}\;\left(\frac{\pi t}{7}\right)$. Similarly, for pattern 2, $\partial_{s}\mu_{2}(s,t)=30\pi \;\text{cos}\;\left(\frac{\pi t}{7}\right)A_{1}(s),\;\partial_{s}^{2}\mu_{2}(s,t)\;=\;-90\pi^{2}\;\text{cos}\;\left(\frac{\pi t}{7}\right)A_{2}(s)$. The true temporal derivatives are $\partial_{t}\mu_{2}(s,t)=-\frac{10\pi }{7}\text{sin}\;\left(3\pi s_{x}\right)\;\text{cos}\;\left(3\pi s_{y}\right)\;\text{sin}\;\left(\frac{\pi t}{7}\right),\;\partial_{t}\partial_{s}\mu_{2}(s,t)\;=-\frac{30\pi^{2}}{7}\text{sin}\;\left(\frac{\pi t}{7}\right)A_{1}(s),\;\partial_{t}\partial_{s}^{2}\mu_{2}(s,t)\;=\;\frac{90\pi^{3}}{7}\text{sin}\;\left(\frac{\pi t}{7}\right)A_{2}(s)$. The true temporal curvatures are as follows, $\partial_{t}^{2}\mu_{2}(s,t)=-\frac{10\pi^{2}}{49}\text{sin}\;\left(3\pi s_{x}\right)\;\text{cos}\;\left(3\pi s_{y}\right)\;\text{cos}\;\left(\frac{\pi t}{7}\right),\;\partial_{t}^{2}\partial_{s}\mu_{2}(s,t)\;=-\frac{30\pi^{3}}{49}\text{cos}\;\left(\frac{\pi t}{7}\right)A_{1}(s)$ and $\partial_{t}^{2}\partial_{s}^{2}\mu_{2}(s,t)=\frac{90\pi^{4}}{49}\text{cos}\;\left(\frac{\pi t}{7}\right)A_{2}(s)$, where the terms A1(s)=cos3πsxcos3πsy-sin3πsxsin3πsy and A2(s)=sin3πsxcos3πsycos3πsxsin3πsycos3πsxsin3πsysin3πsxcos3πsy. Setting up experiments, we vary $N_{s}=\{30,50,100\}$ and $N_{t}=\{3,6,9\}$ with 10 replications for each setting. These settings are chosen to record the effect of varying spatial and temporal samples on the quality of inference.

### Bayesian model fitting.

We fit the model in (9) with an intercept only to learn the functional spatiotemporal patterns in the synthetic response. The following hyper-parameters are used: $a_{\phi_{s}}=a_{\phi_{t}}=0.01,b_{\phi_{s}}=b_{\phi_{t}}=30,a_{\sigma }=a_{\tau }=2,b_{\sigma }=1,b_{\tau }=0.1,\mu_{\beta }=0_{p},\sum_{\beta }=10^{6}I_{p}$ and $a_{v}=0.01,b_{v}=10$. These choices produce weakly informative priors. Generally, a Uniform(2, 3) prior can be placed on ν and this features in our implementation. However, the scales for our data generating patterns are chosen using model fit criteria. Patterns 1 and 2 are favorable to a Matérn with $v=\frac{5}{2}$ or a Gaussian kernel. Figures S10 and S11 compare model fit for the differential processes for Pattern 2 at t=3, a time point of considerable change in y(s,t). Tables S5–S8 present numerical results.

A grid spanning the unit square is overlaid for every t. Posterior predictive inference on the spatiotemporal differential processes is performed at each grid location following developments in Section 2. Medians and 95% highest posterior density (HPD) intervals (Plummer et al. 2006) are used to summarize inference. For each replicate in the simulation setting, the quality of inference on local geometry of the space-time surface is evaluated using coverage probabilities (CP) and root mean squared errors (RMSE).

### Spatiotemporal Surface Wombling.

We perform wombling on spatiotemporal surfaces by specifying planar curves in advance that serve the triangulation process. We aim to locate the curves that track rapid spatial change at each time point on the simulated surfaces. For instance, consider the space-time surfaces generated using Pattern 1. We begin with curves as they allow us to triangulate the wombling surface. Subsequently, we proceed to evaluate the posterior distribution of total or average wombling measure for the surface and assess their significance. In the absence of pre-specified curves they can be selected using *level curves*: $C_{y_{0}^{\left(t\right)}}=\left\{(s,t)\;:\;Y(s,t)=y_{0}^{\left(t\right)}\right\}$, for t=1,…,N. Figure 4 demonstrates this on surfaces generated from Pattern 1. Alternatively, working with *annotated curves*, points selected by the investigator are used to generate a smooth curve, for example, a Beźier spline, specifying regions that possibly contain wombling boundaries within the space-time domain. We consider three level curves for our purposes: closed curves enclosing (A) troughs, (B) peaks and (C) an open curve; see Figure 4(a).

Spatiotemporal surface wombling is pursued following the methods described in Section 3.3. We use a partition with $n_{\omega }=n_{v}=10$ to triangulate the wombling surface. For each pair of triangular regions, we compute areas and outward pointing normals (see Section 4.2). The collection of all pairs represents the wombling surface, $\tilde{𝒞}$. Algorithm 4 in the supplement formulates efficient computation for $𝚪(\tilde{𝒞})$ using two-dimensional quadrature (see, (7) and Section 4.3). Posterior samples of $𝚪(\tilde{𝒞})$ are obtained using (8). For surfaces A,B and C the chosen partitions yield sufficiently small norms (1.5 × 10^−3^, 1.4 × 10^−3^ and 2.2 × 10^−3^ respectively) thereby guaranteeing the accuracy of wombling measures (see Theorem 2 in the supplement). The median (HPD interval) of posterior samples for $𝚪(\tilde{𝒞})$ serves as our point (interval) estimate. The design of our experiment allows us to compute, ${𝚪}_{\text{true}}(𝒞)=\iint_{𝒞}\;{𝓛}_{n_{t},n_{s}}^{*}\mu_{1}(s,t)\;\text{d}𝒜$. Coverage of HPD intervals and significance is assessed at triangular region, time-interval and overall levels. Surface A(B) encloses evolving troughs (peaks) and local minimums (maximums) while surface C delineates a separating region between troughs and peaks (see Figure 4(a)). For surfaces A and C a positive spatial directional gradient is expected along the normal, while B is expected to house negative gradients. They yield significant wombling measures.

Surfaces A and B lie in formative regions (troughs and peaks) associated with evolving curvature, thereby expected to produce significant wombling measures associated with curvature, while surface C lies in a comparatively flat region. With regard to temporal gradients—surface A encloses troughs which flatten by t=4, deepen over t=6 before flattening again. A trough flattening over time implies positive temporal gradients; surface B shows a similar trend with peaks flattening and reforming; surface C encloses regions where peaks mostly flatten yielding negative temporal gradients. For surfaces A and B there is an overall decrease in spatial curvature over time, while for surface C, specifically referring to t=6,7, and 8, the spatial curvature increases over time. These interpretations align with findings presented in Table 1, which assesses the quality of estimation for spatiotemporal wombling measures. Analysis for the overall temporal curvature is similar. A more detailed analysis follows from time-intervals or triangular-region specific considerations (see Section S15). Figure S14 shows 3-D plots displaying significance at a triangular-region level for the wombling surfaces we consider. The magnitude and algebraic sign of the wombling measure helps in discerning the nature and differentiating between boundaries. For example, the rate of curvature decreases at a slower rate on surface A compared to B. Similar conclusions follow from Tables S9 and S10.

### Additional Experiments.

The online supplement contains auxiliary experiments and results. Tables S5–S8 present goodness of fit measures for assessing spatiotemporal differential processes using RMSE, standard deviations and CPs for 95% HPD intervals addressing Patterns 1 and 2. Tables S5 and S6 use *non-separable* kernels while S7 and S8 use *separable* kernels. Increased spatial (temporal) locations result in decreased RMSE for spatial (temporal) derivatives. For instance, comparing results for $N_{s}\times N_{t}=50\times 6$ with $N_{s}\times N_{t}=100\times 3$, we find lower (higher) RMSEs for spatial (temporal) differential processes in the latter. Separable covariance kernels provide fast inference on rates of change and wombling, albeit their several shortcomings (Stein 2005). Figures S10 and S11 compare true and estimated differential processes for Patterns 2 using a Matérn (ν=5/2) kernel at t=3. We are able to learn the induced spatiotemporal differential processes under varying smoothness in process specification. Figures S12 and S13 demonstrate inference for differential geometric quantities–Gaussian curvature, divergence and Laplacian, that capture change.

## 6. Application: Neuroimaging

We study neural activity from EEG sessions designed to test genetic predisposition to alcoholism (Begleiter 1999). Subjects are classified into two groups: alcoholic and control. A standard set of pictures serve as stimulus generating neural activity recorded by the EEG cap through ERPs. Figure 5 shows interpolated spatiotemporal surfaces for average ERPs over six time points. Heavy alcohol users show reduced activity in the occipital and temporal regions (see, e.g., Lew et al. 2020). These regions are demarcated by curves on the scalp (see Figure S15). We are interested in comparing neural activity in these regions between the two groups. The entirety of these regions are not constantly active throughout the measurement period (see Figure 5). Therefore, a space-time surface displaying regions that exhibit significant changes in neural activity for the two groups is of scientific interest (see, e.g., Lenartowicz et al. 2014). Spatiotemporal wombling can answer these questions.

*Projected locations* of 57 electrodes on the EEG cap are used as coordinates (marked with dots in Figure 5) to model the ERP using (9). We use a Matérn kernel with fractal parameter, ν=5/2 that ensures the existence and validity of all spatiotemporal processes within ${𝓛}^{*}Z(s,t)$. Weakly informative priors with hyper-parameter settings outlined in Section 4 are used to conduct Bayesian inference. The resulting posterior estimates are shown in Table 2. A larger value of the variance ratio, $\frac{\sigma^{2}}{\sigma^{2}+\tau^{2}}$ indicates a larger proportion of the total variance being attributed to spatiotemporal change. This implies the presence of strong *spatiotemporal variation* in the data. It is stronger in the control subjects compared to the alcoholic subjects, given $\frac{\hat{\sigma^{2}}}{\hat{\sigma^{2}}+\tau^{2}}=\frac{14.55}{14.55+0.07}=99.52\%$ and $\frac{\hat{\sigma^{2}}}{\sigma^{2}+\tau^{2}}=\frac{5.55}{5.55+0.10}=98.23\%$ of the variation being spatiotemporal respectively. Figure 6 compares the surfaces generated from spatial point estimates of mixed spatial-temporal gradient along the y-axis for the two groups and their difference. We see significant differences at times 1, 2, 5, and 6. For a location $s,\partial_{t}\partial_{s_{y}}Z(s)<0$ implies increased spatial change over time toward the occipital region. This is seen in the control group (second row, Figure 6) for time points t=1,2. The third row tests the difference of ERPs between alcoholic and control groups. A significant positive value implies a higher and quicker rate of change in neural activation of the occipital lobe for the control subjects. Evidently, spatiotemporal gradient analysis aids in discerning differences between groups.

Spatiotemporal wombling is executed on the residual surfaces, Z(s,t), which is not a requirement, but a better choice as it displays variation that is free from the effects explained by covariates, if present. To generate the surface we select the 0 contour. It acts as a separator between regions of high and low activity. The regions enclosed by the curves overlap the occipital and temporal regions. The resulting wombling surface, generated by rotating the topographic head-plots anti-clockwise, enclose spatiotemporally active locations. Triangulation (see Section 4.2) yields the wombling surface approximation. The top and bottom right quadrant of Figure 7 shows the triangulated approximation of the wombling surface enclosing active regions generated for each group. It also shows the triangular region level significance for wombling measures. Comparing the top and bottom right quadrants of Figure 7 reveals a larger proportion of triangular regions with statistically significant wombling measures for the control group than the alcoholic group for all eight measures, except perhaps for $\partial_{s}$. Comparing average wombling measures in Table 3, the alcoholic subjects show significant wombling measures associated only with $\partial_{s}^{2}$ and $\partial_{t}\partial_{s}$. The control group reveal significant measures, with the exception of $\partial_{t}\partial_{s}^{2}$ and $\partial_{t}^{2}\partial_{s}^{2}$. This implies there were significant spatial and temporal changes in the neural activity, however, the overall temporal rates of spatial change, measured by wombling measures corresponding to $\partial_{t}\partial_{s}^{2}$ and $\partial_{t}^{2}\partial_{s}^{2}$ (see Table 3), stayed relatively constant over time. This is a scientific insight of interest in EEG and alcoholism (see, e.g., Durazzo, Stephens, and Meyerhoff 2024). A topographic shift occurs after t=2 in both groups, as seen from the time-interval level analysis in Tables S12 and S13, in the supplement. The spatiotemporal wombling exercise provides concrete statistical evidence of higher neural activity in the occipital and temporal regions for the control group.

We also compared spatiotemporal wombling with purely spatial wombling. Owing to higher neural activity we consider the control group for our comparison. Estimates and HPD intervals for average wombling measures for spatial gradient $\left(\Gamma_{1}\left(C_{t}\right)\right)$ and curvature $\left(\Gamma_{2}\left(C_{t}\right)\right)$ are shown in Table 4. Wombling is performed on the same set of planar curves as seen in the bottom left quadrant of Figure 7. Statistically significant curve segments are shown in Figure 8. Purely spatial wombling does not yield a significant spatial curvature which is in contrast with the findings from its spatiotemporal counterpart. The proportion of significant regions on the wombling surface for spatial gradients is also larger compared to the same for curve segments. The temporal rates of change are unaccounted for in purely spatial wombling, for example, the increase in $\Gamma_{1}\left(C_{t}\right)$, from t=1 to t=2. Thus, in the presence of temporal variation in the data, purely spatial wombling fails to account for the extent of variability, thereby undermining the quality of inference.

## 7. Discussion

We have developed a fully model-based Bayesian framework for inference on differential processes that quantify rates of change in spatiotemporal data. These processes are used to formalize wombling surfaces—a landmark in the existing literature for wombling. The resulting difference surface boundaries and measures aid in discerning zones of rapid spatiotemporal change. GPs prove crucial for calibrating inference for differential processes and wombling. Section S13 in the supplement presents detailed comparisons of wombling with related approaches such as clustering, image segmentation, hot-spot detection and level set modeling. Wombling surfaces are two-dimensional compact Riemannian manifolds which can be triangulated in multiple nonunique ways (see Radó’s theorem). As a result, they can also be constructed using space curves that are interpreted as temporal wombling boundaries which evolve in space.

Future work can proceed along several directions. Extending Bayesian wombling beyond Euclidean domains using GPs on manifolds (see, e.g., Li, Tang, and Banerjee 2023; Li, Halder, and Banerjee 2026) is especially relevant for modern neuroimaging studies. Spatiotemporal energetics is an emerging area related to streaming data from wearable devices that holds significant promise (Alaimo Di Loro et al. 2023). Exploring differential activity can provide scientific insights into human movement and energy patterns. Open-source statistical software development can expand on Halder and Banerjee (2025). Finally, embedding wombling within the predictive stacking inferential framework (see, e.g., Zhang, Tang, and Banerjee 2025) has several theoretical and computational advantages.

## Supplementary Material

Supp 1

The online supplement includes additional theoretical results ranging from tensor reshaping to detailed results on the validity of the spatial processes, derivations of cross-covariance matrices, and some results on spectral densities (Sections S1 through S6), details on the representation of parametric surfaces and curves on them (Sections S7 and S8), some asymptotic properties of the wombling measure (Section S9), some mathematical results leading to computational simplifications (Section S10), Algorithms (Section S11), specific details surrounding the spatiotemporal covariance functions used in the manuscript (Section S12), comparisons with a selection of other methods including clustering and level set detection (Section S13), static wombling (Section S14), additional applications to scientific data analysis and further simulation experiments (Section S15 through S18). A consolidated version that includes the main article and the online supplement in a single manuscript is also available as an arXiv preprint at https://doi.org/10.48550/arXiv.2407.17804.

Supplementary materials for this article are available online. Please go to www.tandfonline.com/r/JASA.

## Figures and Tables

**Figure 1.**
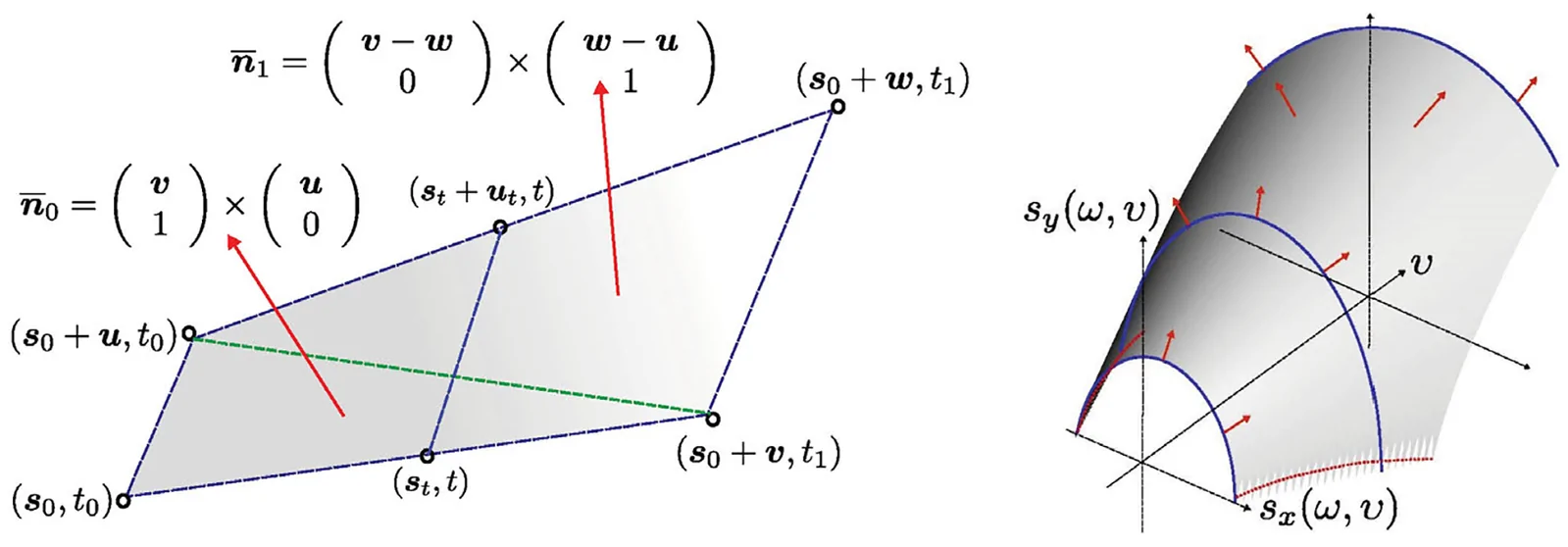
Illustrative examples for wombling: (left) parametric planes (right) parametric circles. Normals to the surface are indicated using red arrows and × denotes the vector product.

**Figure 2.**
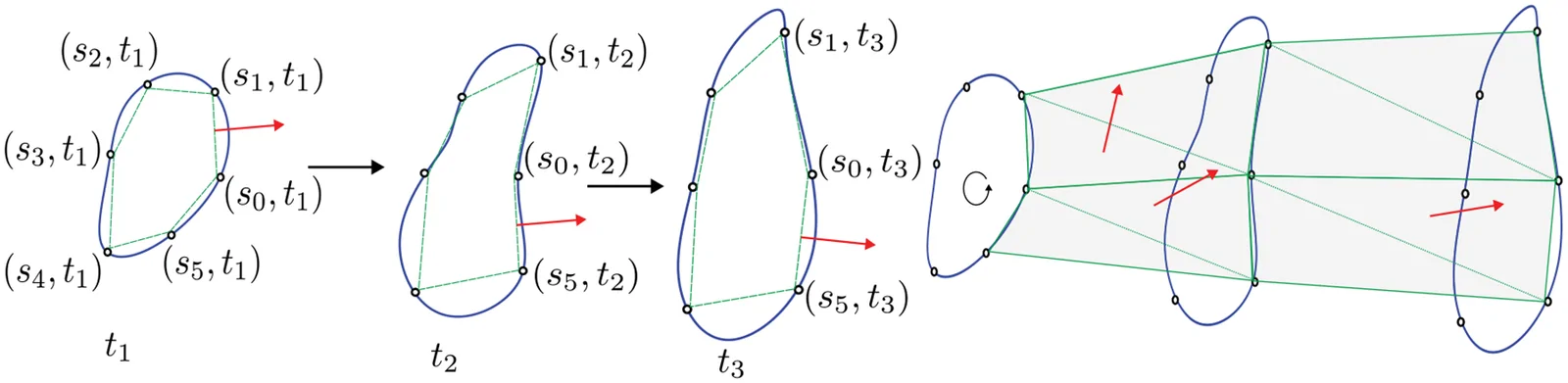
Illustration of spatiotemporal wombling using three time points. At each time point, for a closed planar curve, a partition consisting of six points is chosen to triangulate the surface. An anti-clockwise orientation is used for outward pointing normals which are marked with red arrows.

**Figure 3.**
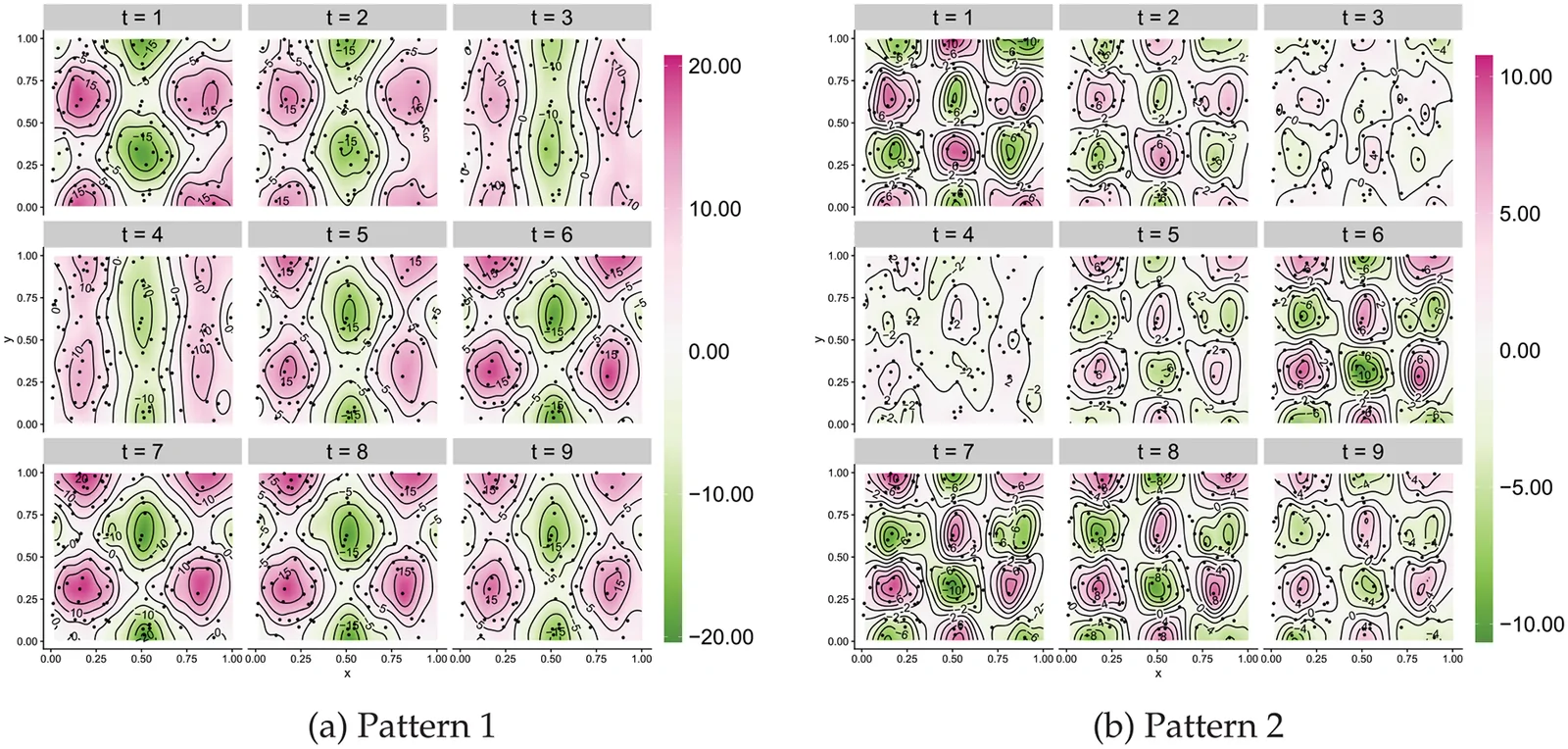
Spatiotemporal surfaces of patterned data used for experiments.

**Figure 4.**
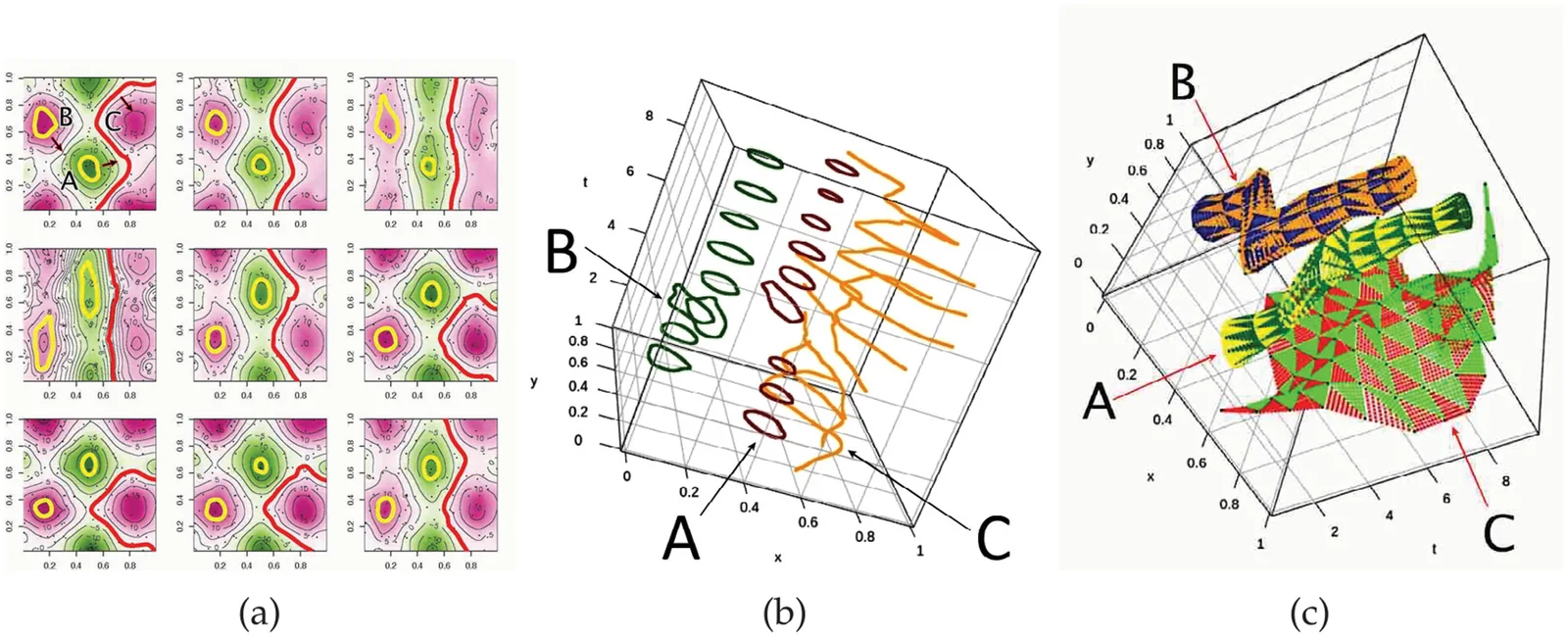
(a) Contours selected—“A” and “B”, marked in yellow, are closed curves and “C”, marked in red, is an open curve. (b) 3-D plot of the planar curves collected over time. (c) Triangulation of the *wombling surfaces* associated with the curves using $n_{\omega }=n_{v}=10$.

**Figure 5.**
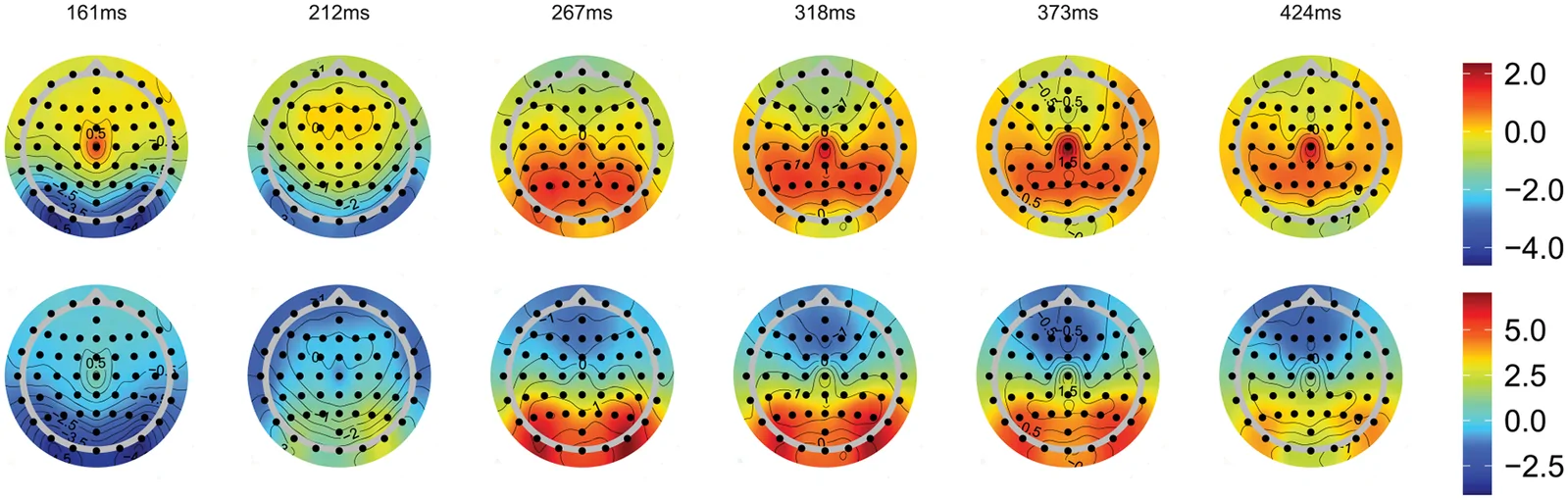
ERP (measured in μV) surfaces for alcoholic (top row) and control (bottom row) subjects.

**Figure 6.**
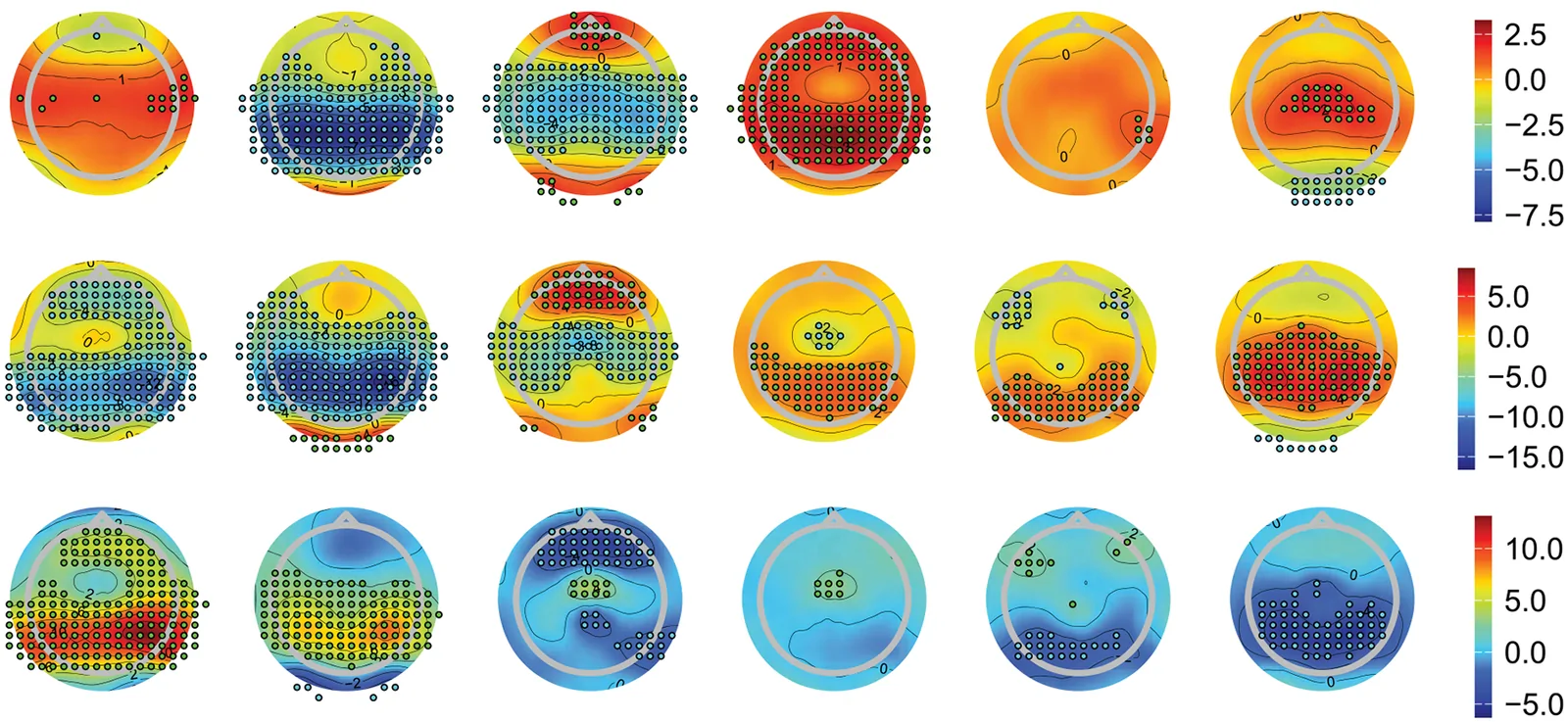
Comparing surfaces generated from spatial point estimates of $\partial_{t}\partial_{s_{y}}$ in ERPs for alcoholic (first row) versus control (second row) and their difference (third row). Significant locations are color-coded: positive/negative (green/cyan).

**Figure 7.**
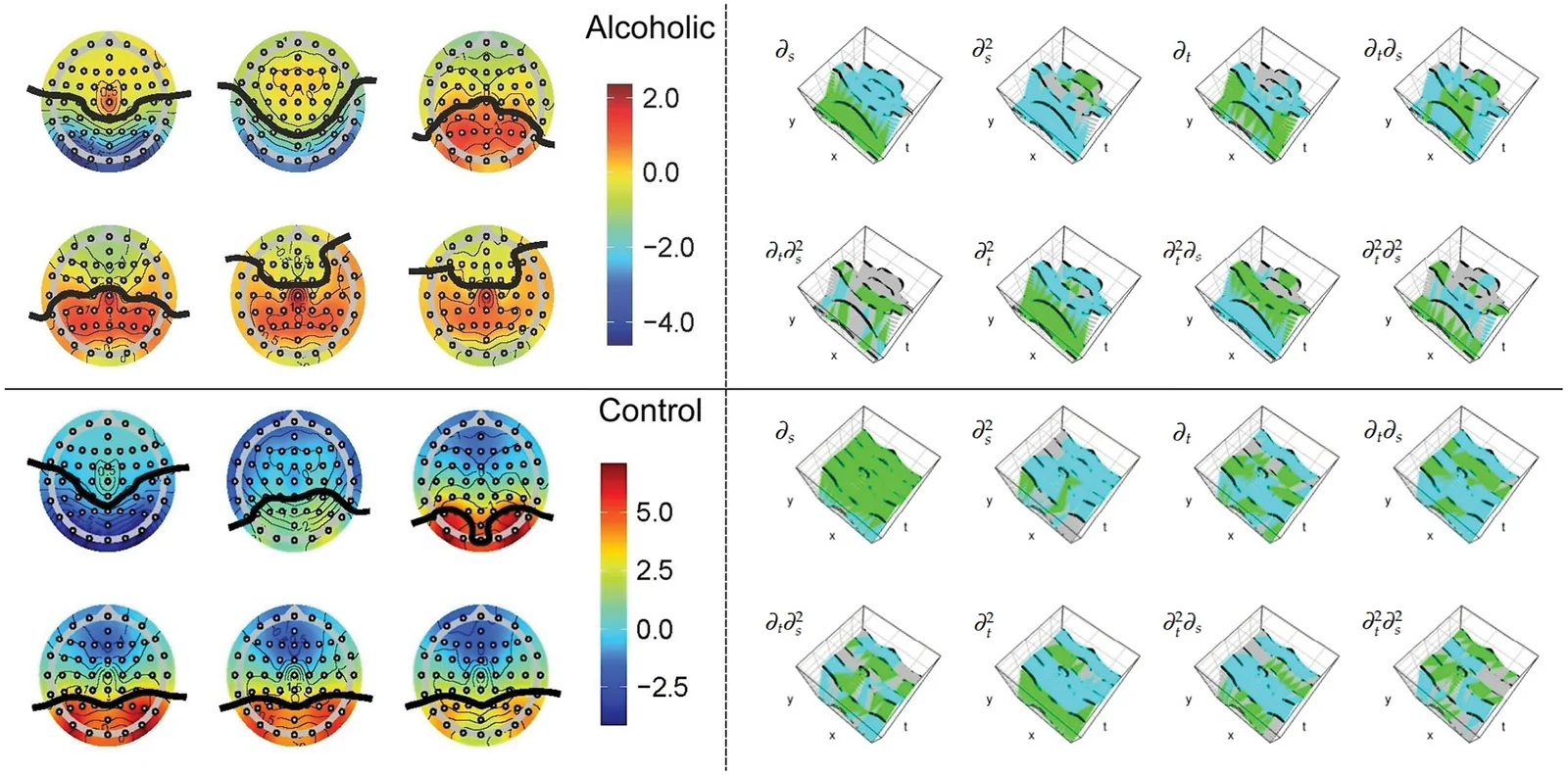
Curves and resulting wombling surfaces for ERPs from alcoholic and control subjects. Significant triangular regions are color-coded: positive/negative (green/cyan), not significant (gray).

**Figure 8.**
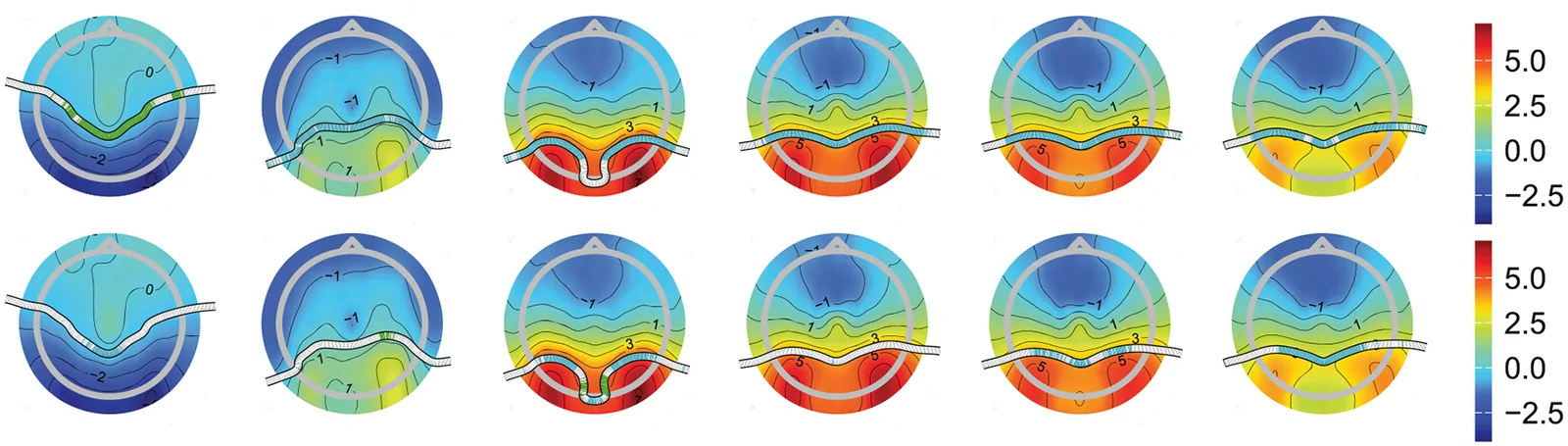
Significance assessments for (top row) spatial gradients $\left(\partial_{s}\right)$ (bottom row) spatial curvature $\left(\partial_{s}^{2}\right)$ over curves used for the control group performing purely spatial wombling. Significant regions are color coded, while white regions indicate no significance.

**Table 1.** True and estimated average wombling measures for surfaces A, B, and C (see Figure 4) accompanied by HPD intervals.

$\bar{𝚪}\left({𝒞}^{*}\right)$	A		B		C	
	Estimate	True	Estimate	True	Estimate	True
${𝝏}_{s}$	**2.23** **(1.65, 2.82)**	**2.25**	**−2.26** **(−2.80, −1.73)**	**−2.21**	**0.98** **(0.48, 1.49)**	**1.04**
${𝝏}_{s}^{2}$	**40.58** **(35.19, 46.01)**	**41.09**	**−36.66** **(−42.43, −30.77)**	**−36.26**	−0.14 (−4.48, 4.28)	−1.43
$\partial_{t}$	**0.16** **(0.03, 0.30)**	**0.15**	**0.33** **(0.20, 0.47)**	**0.32**	**−0.19** **(−0.29, −0.08)**	**−0.19**
$\partial_{t}{𝝏}_{s}$	**1.45** **(0.94, 1.94)**	**1.43**	**0.59** **(0.11, 1.04)**	**0.60**	**−0.59** **(−1.03,−0.18)**	**−0.64**
$\partial_{t}{𝝏}_{s}^{2}$	−7.92 (−16.73, 0.95)	−7.00	**−13.56** **(−23.38, −3.88)**	**−12.07**	**11.07** **(3.63, 18.68)**	**10.56**
$\partial_{t}^{2}$	**0.79** **(0.27, 1.34)**	**0.74**	−0.29 (−0.85, 0.29)	−0.70	0.34 (−0.17, 0.85)	0.16
$\partial_{t}^{2}{𝝏}_{s}$	−3.90 (−9.12, 1.35)	−4.23	3.62 (−2.15, 9.28)	4.53	−0.22 (−4.81, 4.51)	−0.82
$\partial_{t}^{2}{𝝏}_{s}^{2}$	−21.61 (−76.30, 36.11)	−23.44	3.60 (−65.64, 72.73)	25.75	−37.27 (−96.01, 19.40)	−6.72

NOTE: Statistically significant estimates are marked in bold.

**Table 2.** Posterior estimates accompanied by HPD intervals for Θ from the ERP analyses.

Parameter	Estimate		Parameter	Estimate	
	Alcoholic	Control		Alcoholic	Control
$\sigma^{2}$	5.55 (1.57, 13.22)	14.55 (5.90, 30.43)	$\phi_{t}$	0.42 (0.34, 0.55)	0.36 (0.30, 0.43)
$\tau^{2}$	0.10 (0.08, 0.11)	0.07 (0.05, 0.08)	$\beta_{0}$	−0.23 (−0.27, −0.20)	0.82 (0.80, 0.85)
$\phi_{s}$	1.25 (0.86, 1.63)	1.53 (1.27, 1.90)			

**Table 3.** Average wombling measures (WMs) for wombling surfaces selected for the two groups.

Group	$\bar{𝚪}(𝓒)\times 10^{2}$							
	$\partial_{s}$	$\partial_{s}^{2}$	$\partial_{t}$	$\partial_{t}\partial_{s}$	$\partial_{t}\partial_{s}^{2}$	$\partial_{t}^{2}$	$\partial_{t}^{2}\partial_{s}$	$\partial_{t}^{2}\partial_{s}^{2}$
Alcoholic	−7.90 (−17.93, 2.64)	**−105.94** **(−170.77, −40.15)**	0.09 (−0.35, 0.52)	**−1.52** **(−2.74, −0.32)**	1.68 (−7.30, 11.07)	−0.01 (−0.15, 0.13)	−0.17 (−0.78, 0.42)	−1.36 (−4.81, 2.45)
Control	**151.42** **(135.52, 167.30)**	**−307.12** **(−419.33, −192.83)**	**−0.86** **(−1.23, −0.47)**	**−7.43** **(−8.51, −6.33)**	−8.57 (−20.32, 2.23)	**0.39** **(0.26, 0.54)**	**−0.83** **(−1.32, −0.34)**	−1.56 (−5.01, 2.13)

NOTE: Statistically significant estimates are marked in bold.

**Table 4.** Average spatial WMs for gradient, $\Gamma_{1}\left(C_{t}\right)$ and curvature, $\Gamma_{2}\left(C_{t}\right)$ for $C_{t}$ in Figure 8.

Wombling Measures	Time					
	t=1	t=2	t=3	t=4	t=5	t=6
$\Gamma_{1}\left(C_{t}\right)$	**2.21** **(0.41, 4.18)**	0.05 (−2.40, 2.49)	−1.81 (−4.82, 1.03)	**−3.74** **(−7.31, −0.83)**	**−3.80** **(−6.67, −0.67)**	**−3.41** **(−5.80, −1.02)**
$\Gamma_{2}\left(C_{t}\right)$	−7.86 (−25.51, 9.79)	−23.30 (−46.73, 2.43)	−23.11 (−48.72, 1.27)	−1.05 (−33.43, 32.20)	1.58 (−28.05, 30.80)	4.16 (−19.94, 27.40)

NOTE: Statistically significant estimates are marked in bold.
