# Supplementary material for: Bayesian Spatiotemporal Wombling
Source: J Am Stat Assoc. Author manuscript; Available in PMC 2026 Oct 15. (PMC13664449; doi:10.1080/01621459.2026.2700798)
Supplement: Supplementary file 1 [file NIHMS2211750-supplement-Supp_1.zip › uasa_a_2700798_sm0138.pdf]

Supplementary Materials for *"Bayesian Spatiotemporal Wombling"*

Aritra Halder[a], Didong Li[b] and Sudipto Banerjee[c]
[a]Department of Biostatistics & Epidemiology,
Drexel University, Philadelphia, PA, USA.
[b]Department of Biostatistics,
University of North Carolina, Chapel Hill, North Carolina, USA.
[c]Department of Biostatistics,
University of California, Los Angeles, California, USA.

S1 Tensor reshaping: Vectorization

We supplement the discussion in Section 2.1 of the manuscript with details concerning reshaping of tensor differential operators. We are interested in the vectorization of tensors–reshaping tensors to vectors. The order of listing for elements in the tensor operator $\boldsymbol{\partial}^{\otimes r}$ operating on $f : \Re^{d+1} \to \Re$ needs specification. We begin with an explicit definition of $\boldsymbol{\partial}^{\otimes r}$ in terms of its coordinates.

$$\boldsymbol{\partial}^{\otimes r} f(\boldsymbol{x}, t) = \begin{pmatrix} \frac{\partial}{\partial x_1} \\ \vdots \\ \frac{\partial}{\partial x_d} \\ \frac{\partial}{\partial t} \end{pmatrix}^{\otimes r} f(\boldsymbol{x}, t), \tag{S1}$$

where $\boldsymbol{x} = (x_1, \ldots, x_d)^\top$. We illustrate the operation using $d = 2$ and $r = 1, 2, 3$ and 4. For $r = 1$, $\boldsymbol{\partial}^{\otimes 1} f(\boldsymbol{x}, t) = \begin{pmatrix} \frac{\partial}{\partial x_1} \\ \frac{\partial}{\partial x_2} \\ \frac{\partial}{\partial t} \end{pmatrix} f(\boldsymbol{x}, t) = \begin{pmatrix} \frac{\partial}{\partial x_1} f(\boldsymbol{x},t) \\ \frac{\partial}{\partial x_2} f(\boldsymbol{x},t) \\ \frac{\partial}{\partial t} f(\boldsymbol{x},t) \end{pmatrix} = \boldsymbol{\partial} f(\boldsymbol{x}, t)$. For $r = 2$,

$$\begin{aligned}
\boldsymbol{\partial}^{\otimes 2} f(\boldsymbol{x}, t) &= \begin{pmatrix} \frac{\partial}{\partial x_1} \\ \frac{\partial}{\partial x_2} \\ \frac{\partial}{\partial t} \end{pmatrix}^{\otimes 2} f(\boldsymbol{x}, t) = \begin{pmatrix} \frac{\partial}{\partial x_1} \\ \frac{\partial}{\partial x_2} \\ \frac{\partial}{\partial t} \end{pmatrix} \otimes \boldsymbol{\partial} f(\boldsymbol{x}, t) \\
&= \left(\frac{\partial^2}{\partial x_1^2}, \frac{\partial^2}{\partial x_1 \partial x_2}, \frac{\partial^2}{\partial x_1 \partial t}, \frac{\partial^2}{\partial x_2 \partial x_1}, \frac{\partial^2}{\partial x_2^2}, \frac{\partial^2}{\partial x_2 \partial t}, \frac{\partial^2}{\partial t \partial x_1}, \frac{\partial^2}{\partial t \partial x_2}, \frac{\partial^2}{\partial t^2} \right)^\top f(\boldsymbol{x}, t) \\
&= \left(\frac{\partial^2}{\partial x_1^2} f(\boldsymbol{x}, t), \frac{\partial^2}{\partial x_1 \partial x_2} f(\boldsymbol{x}, t), \frac{\partial^2}{\partial x_1 \partial t} f(\boldsymbol{x}, t), \frac{\partial^2}{\partial x_2 \partial x_1} f(\boldsymbol{x}, t), \frac{\partial^2}{\partial x_2^2} f(\boldsymbol{x}, t), \right. \\
&\qquad \left. \frac{\partial^2}{\partial x_2 \partial t} f(\boldsymbol{x}, t), \frac{\partial^2}{\partial t \partial x_1} f(\boldsymbol{x}, t), \frac{\partial^2}{\partial t \partial x_2} f(\boldsymbol{x}, t), \frac{\partial^2}{\partial t^2} f(\boldsymbol{x}, t) \right)^\top,
\end{aligned} \tag{S2}$$

This is the 9×1 vectorized Hessian. For $r = 3$, $\boldsymbol{\partial}^{\otimes 3} f(\boldsymbol{x}, t) = \begin{pmatrix} \frac{\partial}{\partial x_1} \\ \frac{\partial}{\partial x_2} \\ \frac{\partial}{\partial t} \end{pmatrix}^{\otimes 3} f(\boldsymbol{x}, t) = \begin{pmatrix} \frac{\partial}{\partial x_1} \\ \frac{\partial}{\partial x_2} \\ \frac{\partial}{\partial t} \end{pmatrix} \otimes \boldsymbol{\partial}^{\otimes 2} f(\boldsymbol{x}, t)$ produces the vectorized 3-order tensor containing all pure and partial derivatives. Similar calculations follow for $r = 4$.

S2 Details for calculating the covariance between derivative processes, $\partial_t^{j_1} \boldsymbol{\partial}_{\boldsymbol{s}}^{r_1 - j_1} Z(\boldsymbol{s}, t)$ and $\partial_t^{j_2} \boldsymbol{\partial}_{\boldsymbol{s}}^{r_2 - j_2} Z(\boldsymbol{s}, t)$

For ease of notation, we write $\frac{\partial^r}{\partial_t^j \partial x_1^{i_1} \ldots \partial x_d^{i_d}} Z(\boldsymbol{s}, t)$, $\sum_{l=1}^{d} i_l = r - j$ as $\partial_t^j \boldsymbol{\partial}_{\boldsymbol{s}}^{r-j} Z(\boldsymbol{s}, t)$, for $j = 0, \ldots, r$. We aim to derive the covariance between two spatiotemporal derivative processes of possibly different orders.

Proposition 1. *Let $\partial_t^{j_1} \boldsymbol{\partial}_{\boldsymbol{s}}^{r_1 - j_1} Z(\boldsymbol{s}, t)$ and $\partial_t^{j_2} \boldsymbol{\partial}_{\boldsymbol{s}}^{r_2 - j_2} Z(\boldsymbol{s}, t)$ be two spatiotemporal derivative processes of order r_1 and r_2. Then,*

$$\operatorname{Cov}\left(\partial_t^{j_1} \boldsymbol{\partial}_{\boldsymbol{s}}^{r_1 - j_1} Z(\boldsymbol{s}, t), \partial_t^{j_2} \boldsymbol{\partial}_{\boldsymbol{s}}^{r_2 - j_2} Z(\boldsymbol{s} + \boldsymbol{\Delta}, t + \delta) \right) = (-1)^{r_1} \partial_t^{j_1 + j_2} \boldsymbol{\partial}_{\boldsymbol{s}}^{r_1 + r_2 - (j_1 + j_2)} K(\boldsymbol{\Delta}, \delta),$$

for every $j_1 = 0, \ldots, r_1$ and $j_2 = 0, \ldots, r_2$.

Proof. We express the covariance between the two processes as a limit of the covariance

between its finite difference approximations as,

$$\mathrm{Cov}\left(\partial_t^{j_1}\boldsymbol{\partial}_{\boldsymbol{s}}^{r_1-j_1}Z(\boldsymbol{s},t),\partial_t^{j_2}\boldsymbol{\partial}_{\boldsymbol{s}}^{r_2-j_2}Z(\boldsymbol{s}+\boldsymbol{\Delta},t+\delta)\right)=\lim_{k\to 0}\lim_{h\to 0}\mathrm{Cov}\left(Z_{k,\mathbf{I}_d,1}^{(j_1,r_1)}(\boldsymbol{s},t),\ Z_{h,\mathbf{I}_d,1}^{(j_2,r_2)}(\boldsymbol{s}+\boldsymbol{\Delta},t+\delta)\right),$$

where $Z_{k,\mathbf{I}_d,1}^{(j_1,r_1)}(\boldsymbol{s},t)=k^{-r_1}\prod_{l=1}^{d}(D_{k,\boldsymbol{e}_l,0}-1)^{i_l}(D_{k,0,1}-1)^{j_1}Z(\boldsymbol{s},t)$, $Z_{h,\mathbf{I}_d,1}^{(j_2,r_2)}(\boldsymbol{s}+\boldsymbol{\Delta},t+\delta)=h^{-r_2}\prod_{l'=1}^{d}(D_{h,\boldsymbol{e}_{l'},0}-1)^{l'}(D_{h,0,1}-1)^{j_2}Z(\boldsymbol{s}+\boldsymbol{\Delta},t+\delta)$, $\sum_{l=1}^{d}i_l=r_1-j_1$ and $\sum_{l'=1}^{d}i_{l'}=r_2-j_2$. Using these expressions and performing binomial expansion on the spatiotemporal shift operator we obtain

$$\begin{aligned}
&\lim_{k\to 0}\lim_{h\to 0}\mathrm{Cov}\left(Z_{k,\mathbf{I}_d,1}^{(j_1,r_1)}(\boldsymbol{s},t),\ Z_{h,\mathbf{I}_d,1}^{(j_2,r_2)}(\boldsymbol{s}+\boldsymbol{\Delta},t+\delta)\right)=\\
&\lim_{k\to 0}\lim_{h\to 0}\ k^{-r_1}\ h^{-r_2}\ \mathrm{Cov}\left(\prod_{l=1}^{d}\sum_{i=1}^{i_l}\sum_{j=1}^{j_1}\binom{i_l}{i}\binom{j_1}{j}(-1)^{(i_l+j_1)-(i+j)}\ Z(\boldsymbol{s}+ik\boldsymbol{e}_l,t+jk),\right.\\
&\qquad\qquad\left.\prod_{l'=1}^{d}\sum_{i'=1}^{i_{l'}}\sum_{j'=1}^{j_2}\binom{i_{l'}}{i'}\binom{j_2}{j'}(-1)^{(i_{l'}+j_2)-(i'+j')}\ Z(\boldsymbol{s}+\boldsymbol{\Delta}+i'h\boldsymbol{e}_{l'},t+\delta+j'h)\right),\\
&=(-1)^{r_1}\partial_t^{j_1+j_2}\boldsymbol{\partial}_{\boldsymbol{s}}^{r_1+r_2-(j_1+j_2)}\ K(\boldsymbol{\Delta},\delta),
\end{aligned}$$

for every $j_1=0,\ldots,r_1$ and $j_2=0,\ldots,r_2$. The last equality is obtained after some algebra. □

The algebraic sign depends on the order of the first derivative process. Section S4 provides the cross-covariance matrix using the above result for the process, $\binom{Z(\boldsymbol{s},t)}{\mathcal{L}^*Z(\boldsymbol{s},t)}$. Subsequently, Section S12 provides the required expressions for our kernel choices.

S3 Validity of the process $\mathcal{L}_r$, its consequences and distributions for the divergence and Laplacian

Let $Z(\boldsymbol{s},t)$ be a zero-centered process with $\mathrm{Cov}(Z(\boldsymbol{s},t),Z(\boldsymbol{s}',t'))=K(\boldsymbol{\Delta},\delta)$, $\boldsymbol{\Delta}=\boldsymbol{s}-\boldsymbol{s}'$ and $\delta=t-t'$. Consider the process $(Z(\boldsymbol{s},t),\mathcal{L}_1Z(\boldsymbol{s},t))^{\mathrm{T}}=(Z(\boldsymbol{s},t),\boldsymbol{\partial}_{\boldsymbol{s}}Z(\boldsymbol{s},t)^{\mathrm{T}},\partial_tZ(\boldsymbol{s},t))^{\mathrm{T}}$. Define the lower triangular matrix $\mathcal{G}_h=\begin{pmatrix}1&&\\-\frac{1}{h}\mathbf{1}_d&\frac{1}{h}\mathbf{I}_d&\\-\frac{1}{h}&&\frac{1}{h}\end{pmatrix}$ and the $(d+2)$-vector $\boldsymbol{W}_h=\begin{pmatrix}1\\D_{h,\boldsymbol{e}_1,0}\\\vdots\\D_{h,\boldsymbol{e}_d,0}\\D_{h,0,1}\end{pmatrix}$. The associated

finite difference process is $\mathbf{U}_h^{(1)}(\boldsymbol{s},t) = \mathscr{G}_h \boldsymbol{W}_h Z(\boldsymbol{s},t)$. The process $\mathbf{U}_h^{(1)}(\boldsymbol{s},t)$ is valid. It is zero-centered, since, $\mathbb{E}\mathbf{U}_h^{(1)}(\boldsymbol{s},t) = \mathscr{G}_h \boldsymbol{W}_h \mathbb{E}Z(\boldsymbol{s},t) = \mathbf{0}_{1+\binom{d+1}{1}}$ and $\mathrm{Cov}(\mathbf{U}_h^{(1)}(\boldsymbol{s},t), \mathbf{U}_h^{(1)}(\boldsymbol{s}',t')) = \mathscr{G}_h \boldsymbol{W}_h \mathbf{K}(\boldsymbol{\Delta},\delta)\,(\mathscr{G}_h \boldsymbol{W}_h)^{\mathrm{T}}$ for $h \neq 0$. The next lemma generalizes this for any order $r > 0$.

Lemma 1. *For $h \neq 0$ and any $r > 0$, let $\widetilde{\mathscr{G}_h}^{\otimes r}$ be a matrix containing finite difference coefficients and $\widetilde{\boldsymbol{W}}_h^{\otimes r}$ be a vector of spatiotemporal shift operators corresponding to unique spatiotemporal derivative processes up to order r. The finite difference process, $\mathbf{U}_h^{(r)}(\boldsymbol{s},t) = \widetilde{\mathscr{G}_h}^{\otimes r} \widetilde{\boldsymbol{W}}_h^{\otimes r} Z(\boldsymbol{s},t)$ corresponding to $(Z(\boldsymbol{s},t), \boldsymbol{\mathcal{L}}_r Z(\boldsymbol{s},t)^{\mathrm{T}})^{\mathrm{T}}$ is valid.*

Proof. The process is valid if for every $r > 0$, $\widetilde{\mathscr{G}_h}^{\otimes r}$ is non-singular. We rely on an inductive argument. For $r = 1$, set $\widetilde{\mathscr{G}_h}^{\otimes 1} = \mathscr{G}_h$ and $\widetilde{\boldsymbol{W}}_h^{\otimes 1} = \boldsymbol{W}_h$. Since $\det \mathscr{G}_h = h^{-\left\{0\cdot 1 + 1\cdot\binom{d+1}{1}\right\}} \neq 0$, this results in a non-singular linear transformation of $\boldsymbol{W}_h Z(\boldsymbol{s},t)$ for every $h \neq 0$. For $r = 2$, $(Z(\boldsymbol{s},t), \boldsymbol{\mathcal{L}}_2 Z(\boldsymbol{s},t)^{\mathrm{T}})^{\mathrm{T}} = (Z(\boldsymbol{s},t), \boldsymbol{\partial}_{\boldsymbol{s}} Z(\boldsymbol{s},t)^{\mathrm{T}}, \partial_t Z(\boldsymbol{s},t), \widetilde{\boldsymbol{\partial}_{\boldsymbol{s}}}^{2} Z(\boldsymbol{s},t)^{\mathrm{T}}, \partial_t \boldsymbol{\partial}_{\boldsymbol{s}} Z(\boldsymbol{s},t)^{\mathrm{T}}, \partial_t^2 Z(\boldsymbol{s},t))^{\mathrm{T}}$. The associated finite difference process is $\mathbf{U}_h^{(2)}(\boldsymbol{s},t) = \widetilde{\mathscr{G}_h}^{\otimes 2} \widetilde{\boldsymbol{W}}_h^{\otimes 2} Z(\boldsymbol{s},t)$, where $\widetilde{\boldsymbol{W}}_h^{\otimes 2}$ contains only unique elements from $\boldsymbol{W}_h^{\otimes 2}$ and $\widetilde{\mathscr{G}_h}^{\otimes 2}$ is constructed from $\mathscr{G}_h^{\otimes 2}$ containing finite differences coefficients corresponding to the elements in $\widetilde{\boldsymbol{W}}_h^{\otimes 2}$. The vector $\widetilde{\boldsymbol{W}}_h^{\otimes 2}$ is comprised of $\boldsymbol{W}_h$ and $\left\{ D_{h,0,1}^{i_{d+1}} \prod_{j=1}^{d} D_{h,\boldsymbol{e}_j,0}^{i_j} \right\}$, $\sum_{j=1}^{d+1} i_j = 2$, $i_j \geq 0$. The number of integer solutions to the equation $\sum_{j=1}^{d+1} i_j = 2$, $i_j \geq 0$ is $\binom{d+2}{2}$. The matrix $G_h^{\otimes 2}$ (and $\widetilde{\mathscr{G}_h}^{\otimes 2}$) is lower triangular since $\mathscr{G}_h$ is lower triangular. The second order finite difference processes in $\widetilde{\boldsymbol{W}}_h^{\otimes 2} Z(\boldsymbol{s},t)$ are constructed from first order finite difference processes in $\boldsymbol{W}_h Z(\boldsymbol{s},t)$. Also, $\widetilde{\mathscr{G}_h}^{\otimes 2}$ is non-singular since $\det \widetilde{\mathscr{G}_h}^{\otimes 2} = h^{-\left\{0\cdot 1 + 1\cdot\binom{d+1}{1} + 2\cdot\binom{d+2}{2}\right\}} \neq 0$. Hence, $\mathbf{U}_h^{(2)}(\boldsymbol{s},t)$ is a non-singular linear transformation of $\widetilde{\boldsymbol{W}_h}^{\otimes 2} Z(\boldsymbol{s},t)$. For example, if $d = 2$, then

$$\boldsymbol{W}_h^{\otimes 2} = \Big(\boldsymbol{W}_h^{\mathrm{T}},\ D_{h,\boldsymbol{e}_1,0},\ D_{h,\boldsymbol{e}_1,0}^2,\ D_{h,\boldsymbol{e}_1,0}D_{h,\boldsymbol{e}_2,0},\ D_{h,\boldsymbol{e}_1,0}D_{h,0,1},\ D_{h,\boldsymbol{e}_2,0},\ D_{h,\boldsymbol{e}_2,0}D_{h,\boldsymbol{e}_1,0},$$
$$D_{h,\boldsymbol{e}_2,0}^2,\ D_{h,0,1}D_{h,\boldsymbol{e}_2,0},\ D_{h,0,1},\ D_{h,0,1}D_{h,\boldsymbol{e}_1,0},\ D_{h,0,1}D_{h,\boldsymbol{e}_2,0},\ D_{h,0,1}^2\Big)^{\mathrm{T}},$$

and $\widetilde{\boldsymbol{W}_h}^{\otimes 2} = \left(\boldsymbol{W}_h^{\mathrm{T}}, D_{h,\boldsymbol{e}_1,0}^2, D_{h,\boldsymbol{e}_1,0}D_{h,\boldsymbol{e}_2,0}, D_{h,\boldsymbol{e}_1,0}D_{h,0,1}, D_{h,\boldsymbol{e}_2,0}^2, D_{h,0,1}D_{h,\boldsymbol{e}_2,0}, D_{h,0,1}^2\right)^{\mathrm{T}}$ contains only the unique spatiotemporal shift operators. Next, $\mathscr{G}_h^{\otimes 2} = \begin{pmatrix} \mathscr{G}_h & & & \\ -\frac{1}{h}\mathscr{G}_h & \frac{1}{h}\mathscr{G}_h & & \\ -\frac{1}{h}\mathscr{G}_h & & \frac{1}{h}\mathscr{G}_h & \\ -\frac{1}{h}\mathscr{G}_h & & & \frac{1}{h}\mathscr{G}_h \end{pmatrix}$. Using column operations followed by row-column elimination matrices on $\mathscr{G}_h^{\otimes 2}$ produces

$$\widetilde{\mathscr{G}_h}^{\otimes 2} = \begin{pmatrix} \mathscr{G}_h & & & \\ \mathscr{G}_{21} & \frac{1}{h^2}\mathbf{I}_3 & & \\ \mathscr{G}_{31} & & \frac{1}{h^2}\mathbf{I}_2 & \\ \mathscr{G}_{41} & & & \frac{1}{h^2} \end{pmatrix}, \quad \mathscr{G}_{21} = \begin{pmatrix} \frac{1}{h^2} & -\frac{2}{h^2} & 0 & 0 \\ \frac{1}{h^2} & -\frac{1}{h^2} & -\frac{1}{h^2} & 0 \\ \frac{1}{h^2} & -\frac{1}{h^2} & 0 & -\frac{1}{h^2} \end{pmatrix}, \quad \mathscr{G}_{31} = \begin{pmatrix} \frac{1}{h^2} & 0 & -\frac{2}{h^2} & 0 \\ \frac{1}{h^2} & 0 & -\frac{1}{h^2} & -\frac{1}{h^2} \end{pmatrix} \text{ and } \mathscr{G}_{41} = \begin{pmatrix} \frac{1}{h^2} \\ 0 \\ 0 \\ -\frac{2}{h^2} \end{pmatrix}^{\mathrm{T}},$$

which is lower triangular. The row and column elimination matrices are $\mathbf{E}_{r2} = \mathbf{I}_3 \oplus \mathbf{O}_1 \oplus \mathbf{I}_2 \oplus \mathbf{O}_2 \oplus \mathbf{I}_1$ and $\mathbf{E}_{c2} = \mathbf{E}_{r2}^{\mathrm{T}}$ respectively. For $r = k-1$, let $\mathbf{U}_h^{(k-1)}(\boldsymbol{s},t) = \widetilde{\mathscr{G}_h}^{\otimes k-1}\widetilde{\boldsymbol{W}_h}^{\otimes k-1}Z(\boldsymbol{s},t)$ be the finite difference process associated with $(Z(\boldsymbol{s},t), \boldsymbol{\mathcal{L}}_{k-1}Z(\boldsymbol{s},t)^{\mathrm{T}})^{\mathrm{T}}$, where $\widetilde{\boldsymbol{W}_h}^{\otimes k-1}$ is comprised of shift operators required for finite difference processes up to order $k-1$ and $\widetilde{\mathscr{G}_h}^{\otimes k-1}$ is a lower-triangular non-singular matrix of finite difference coefficients of order $k-1$ with, $\det \widetilde{\mathscr{G}_h}^{\otimes(k-1)} = h^{-\sum_{i=0}^{k-1} i\binom{d+i}{i}}$. Then, for $r = k$, the process $(Z(\boldsymbol{s},t), \boldsymbol{\mathcal{L}}_k Z(\boldsymbol{s},t)^{\mathrm{T}})^{\mathrm{T}}$ has the associated finite difference process $\mathbf{U}_h^{(k)}(\boldsymbol{s},t) = \widetilde{\mathscr{G}_h}^{\otimes k}\widetilde{\boldsymbol{W}_h}^{\otimes k}Z(\boldsymbol{s},t)$, where $\widetilde{\boldsymbol{W}_h}^{\otimes k}$ is composed of $\widetilde{\boldsymbol{W}_h}^{\otimes k-1}$ and $\left\{D_{h,0,1}^{i_{d+1}} \prod_{j=1}^{d} D_{h,\boldsymbol{e}_j,0}^{i_j}\right\}$, $\sum_{j=1}^{d+1} i_j = k$, $i_j \geq 0$. The number of integer solutions to this equation is $\binom{d+k}{k}$. Observe that $\mathscr{G}_h^{\otimes k}$ (and $\widetilde{\mathscr{G}_h}^{\otimes k}$) is lower triangular since $\mathscr{G}_h^{\otimes k-1}$ (and $\widetilde{\mathscr{G}_h}^{\otimes k-1}$) is lower triangular and the k-order finite difference processes are constructed using the $(k-1)$-order processes in $\widetilde{\boldsymbol{W}_h}^{\otimes k-1}Z(\boldsymbol{s},t)$. The matrix $\widetilde{\mathscr{G}_h}^{\otimes k}$ is non-singular since, $\det \widetilde{\mathscr{G}_h}^{\otimes k} = \det \widetilde{\mathscr{G}_h}^{\otimes k-1} h^{-k\binom{d+k}{k}}$ which is non-zero for $h \neq 0$ implying that $\mathbf{U}_h^{(k)}(\boldsymbol{s},t)$ is a non-singular transformation of $\widetilde{\boldsymbol{W}_h}^{\otimes k}Z(\boldsymbol{s},t)$. □

The process $(Z(\boldsymbol{s},t), \boldsymbol{\mathcal{L}}_r Z(\boldsymbol{s},t)^{\mathrm{T}})^{\mathrm{T}}$ is valid if the associated mean vector and cross-covariance matrix are well-defined. The next theorem elucidates the necessary conditions.

Theorem 1. *For $r > 0$, if $\partial_t^{2j}\boldsymbol{\partial}_{\boldsymbol{s}}^{2(r-j)}K(\mathbf{0}_d, 0)$ exists for every $j = 0, \ldots, r$, then $(Z(\boldsymbol{s},t), \boldsymbol{\mathcal{L}}_r Z(\boldsymbol{s},t)^{\mathrm{T}})^{\mathrm{T}}$ is a valid zero-centered process.*

Proof. We use a limiting argument on the finite difference process, $\mathbf{U}_h^{(r)}(\boldsymbol{s},t)$ described in Lemma 1. Observe that $\mathbb{E}\mathbf{U}_h^{(r)}(\boldsymbol{s},t) = \widetilde{\mathscr{G}_h}^{\otimes r}\widetilde{\boldsymbol{W}_h}^{\otimes r}\mathbb{E}Z(\boldsymbol{s},t) = \mathbf{0}_m$ and $\mathrm{Cov}\{\mathbf{U}_h^{(r)}(\boldsymbol{s},t), \mathbf{U}_h^{(r)}(\boldsymbol{s}',t')\} = \mathrm{Cov}\{\widetilde{\mathscr{G}_h}^{\otimes r}\widetilde{\boldsymbol{W}_h}^{\otimes r}Z(\boldsymbol{s},t), \widetilde{\mathscr{G}_h}^{\otimes r}\widetilde{\boldsymbol{W}_h}^{\otimes r}Z(\boldsymbol{s}',t')\} = \widetilde{\mathscr{G}_h}^{\otimes r}\widetilde{\boldsymbol{W}_h}^{\otimes r}K(\boldsymbol{\Delta},\delta)(\widetilde{\mathscr{G}_h}^{\otimes r}\widetilde{\boldsymbol{W}_h}^{\otimes r})^{\mathrm{T}}$, where $m = \sum_{i=0}^{r}\binom{d+i}{i}$, for every $r > 0$ and $h \neq 0$. $\mathbf{U}_h^{(r)}(\boldsymbol{s},t)$ is a well-defined zero-centered process. The elements of $\mathbf{U}_h^{(r)}(\boldsymbol{s},t)$ are $Z(\boldsymbol{s},t)$ and $\left\{\frac{1}{h^k}(D_{h,0,1}-1)^{i_{d+1}}\prod_{j=1}^{d}(D_{h,\boldsymbol{e}_j,0}-1)^{i_j}\right\}Z(\boldsymbol{s},t)$, $\sum_{j=1}^{d+1} i_j = k$, $i_j \geq 0$, for every $k = 1, \ldots, r$. Taking the limit, $\lim_{h\downarrow 0}\frac{1}{h^k}(D_{h,0,1}-1)^{i_{d+1}}\prod_{j=1}^{d}(D_{h,\boldsymbol{e}_j,0}-1)^{i_j}Z(\boldsymbol{s},t) = \partial_t^{i_{d+1}}\boldsymbol{\partial}_{\boldsymbol{s}}^{k-i_{d+1}}Z(\boldsymbol{s},t)$, $0 \leq i_{d+1} \leq k$. Hence, $\lim_{h\downarrow 0}\mathbf{U}_h^{(r)}(\boldsymbol{s},t) = \lim_{h\downarrow 0}\widetilde{\mathscr{G}_h}^{\otimes r}\widetilde{\boldsymbol{W}_h}^{\otimes r}Z(\boldsymbol{s},t) = \binom{Z(\boldsymbol{s},t)}{\boldsymbol{\mathcal{L}}_r Z(\boldsymbol{s},t)}$, where the limit operates

element-wise. Clearly, $\mathbb{E}\left(\begin{smallmatrix} Z(\boldsymbol{s},t) \\ \mathcal{L}_r Z(\boldsymbol{s},t)\end{smallmatrix}\right) = \lim_{h\downarrow 0} \mathbb{E}\mathbf{U}_h^{(r)}(\boldsymbol{s},t) = \lim_{h\downarrow 0} \widetilde{\mathscr{G}}_h^{\otimes r} \widetilde{\boldsymbol{W}}_h^{\otimes r} \mathbb{E}Z(\boldsymbol{s},t) = 0_m$. Similarly, $\mathrm{Cov}\left\{\left(\begin{smallmatrix} Z(\boldsymbol{s},t) \\ \mathcal{L}_r Z(\boldsymbol{s},t)\end{smallmatrix}\right), \left(\begin{smallmatrix} Z(\boldsymbol{s}',t') \\ \mathcal{L}_r Z(\boldsymbol{s}',t')\end{smallmatrix}\right)\right\} = \lim_{h\downarrow 0} \widetilde{\mathscr{G}}_h^{\otimes r} \widetilde{\boldsymbol{W}}_h^{\otimes r} K(\boldsymbol{\Delta},\delta) \left(\widetilde{\mathscr{G}}_h^{\otimes r} \widetilde{\boldsymbol{W}}_h^{\otimes r}\right)^{\mathrm{T}} = \{(-1)^{r_1} \partial_t^{j_1+j_2} \boldsymbol{\partial}_{\boldsymbol{s}}^{r_1+r_2-j_1-j_2} K(\boldsymbol{\Delta},\delta)\}$, where $0 \le r_1, r_2 \le r$, $0 \le j_i \le r_i$, $i = 1,2$. The last equality is obtained using Proposition 1 and the limit operates on matrices. The covariance matrix is well-defined if all the above entries exist including the variances, obtained as $(\boldsymbol{\Delta},\delta) \to (\mathbf{0}_d, 0)$. The existence of $\partial_t^{2j} \boldsymbol{\partial}_{\boldsymbol{s}}^{2(r-j)} K(\mathbf{0}_d, 0)$, for $0 \le j \le r$, is necessary. □

Constructing $\mathcal{L}^*$, we only need $j = r - j = 2$ and hence require the existence of $\partial_t^4 \boldsymbol{\partial}_{\boldsymbol{s}}^4 K(\mathbf{0}_d, 0)$. Here onwards, we assume $Z(\boldsymbol{s},t) \sim GP(0,K)$. The next corollary provides some immediate consequences of this assumption.

Corollary 1. *If $Z_1(\boldsymbol{s},t) \sim GP(0,K_1(\cdot,\cdot))$ and $Z_2(\boldsymbol{s},t) \sim GP(0,K_2(\cdot,\cdot))$ independently, then (i) $\mathcal{L}_{r_1} Z_1(\boldsymbol{s},t)$ and $\mathcal{L}_{r_2} Z_2(\boldsymbol{s},t)$ are independent GPs, (ii) for $a_1, a_2 \in \Re$, $\mathcal{L}_r(a_1 Z_1(\boldsymbol{s},t) + a_2 Z_2(\boldsymbol{s},t)) = a_1 \mathcal{L}_r Z_1(\boldsymbol{s},t) + a_2 \mathcal{L}_r Z_2(\boldsymbol{s},t)$ is a GP, and (iii) any sub-vector of $\mathcal{L}_r Z(\boldsymbol{s},t)$ is a GP.*

Proof. Observe that from Theorem 1, $\mathcal{L}_{r_i} Z_i(\boldsymbol{s},t)$ is a zero-centered GP with cross-covariance matrix $\mathbf{V}_{\mathcal{L}_{r_i} Z}(\boldsymbol{\Delta},\delta) = \{(-1)^{k_1} \partial_t^{j_1+j_2} \boldsymbol{\partial}_{\boldsymbol{s}}^{k_1+k_2-j_1-j_2} K(\boldsymbol{\Delta},\delta)\}$, $0 < k_1, k_2 \le r_i$, $0 \le j_i \le k_i$, for $i = 1,2$ and $\mathrm{Cov}(\mathcal{L}_{r_1} Z_1(\boldsymbol{s},t), \mathcal{L}_{r_2} Z_2(\boldsymbol{s},t)) = \lim_{h\downarrow 0} \mathrm{Cov}\left(\widetilde{\mathscr{G}}_h^{\otimes r_1} \widetilde{\boldsymbol{W}}_h^{\otimes r_1} Z_1(\boldsymbol{s},t), \widetilde{\mathscr{G}}_h^{\otimes r_2} \widetilde{\boldsymbol{W}}_h^{\otimes r_2} Z_2(\boldsymbol{s},t)\right) = \lim_{h\downarrow 0} \widetilde{\mathscr{G}}_h^{\otimes r_1} \widetilde{\boldsymbol{W}}_h^{\otimes r_1} \cdot 0 \cdot \left(\widetilde{\mathscr{G}}_h^{\otimes r_2} \widetilde{\boldsymbol{W}}_h^{\otimes r_2}\right)^{\mathrm{T}} = 0$ for $r_1, r_2 > 0$. Consider $\mathcal{L}_r(a_1 Z_1(\boldsymbol{s},t) + a_2 Z_2(\boldsymbol{s},t)) = \lim_{h\downarrow 0} \widetilde{\mathscr{G}}_h^{\otimes r} \widetilde{\boldsymbol{W}}_h^{\otimes r} (a_1 Z_1(\boldsymbol{s},t) + a_2 Z_2(\boldsymbol{s},t)) = a_1 \lim_{h\downarrow 0} \widetilde{\mathscr{G}}_h^{\otimes r} \widetilde{\boldsymbol{W}}_h^{\otimes r} Z_1(\boldsymbol{s},t) + a_2 \lim_{h\downarrow 0} \widetilde{\mathscr{G}}_h^{\otimes r} \widetilde{\boldsymbol{W}}_h^{\otimes r} Z_2(\boldsymbol{s},t) = a_1 \mathcal{L}_r Z_1(\boldsymbol{s},t) + a_2 \mathcal{L}_r Z_2(\boldsymbol{s},t)$. Finally, since $Z_1(\boldsymbol{s},t)$ and $Z_2(\boldsymbol{s},t)$ are independent GPs, $a_1 \mathcal{L}_r Z_1(\boldsymbol{s},t) + a_2 \mathcal{L}_r Z_2(\boldsymbol{s},t)$ is also a GP with zero mean and cross-covariance $a_1 \mathbf{V}_{\mathcal{L}_{r_1}}(\boldsymbol{\Delta},\delta) + a_2 \mathbf{V}_{\mathcal{L}_{r_2}}(\boldsymbol{\Delta},\delta)$. Lastly, since $\mathcal{L}_r Z(\boldsymbol{s},t)$ is a GP, any sub-vector is also a zero-centered GP with the cross-covariance being a sub-matrix of $\mathbf{V}_{\mathcal{L}_r}(\boldsymbol{\Delta},\delta)$ (see, e.g., Rasmussen & Williams 2005). □

S4 Cross-Covariance Matrix for $(Z, \boldsymbol{\mathcal{L}}^{*}Z^{\mathrm{T}})^{\mathrm{T}}$

Assuming $d = 2$ and suppressing the dependence on $\boldsymbol{\Delta} = \boldsymbol{s} - \boldsymbol{s}'$ and $\delta = t - t'$, the resulting cross-covariance matrix for the process, $(Z(\boldsymbol{s},t), \boldsymbol{\mathcal{L}}^{*}Z(\boldsymbol{s},t)^{\mathrm{T}})^{\mathrm{T}}$ denoted by $\mathbf{V}_{Z,\boldsymbol{\mathcal{L}}^{*}Z}$, is

$$
\left[\begin{array}{ccc|ccc|ccc}
\mathbf{K} & \boldsymbol{\partial}_s\mathbf{K}^{\mathrm{T}} & \widetilde{\boldsymbol{\partial}_s}^{2}\mathbf{K}^{\mathrm{T}} & \partial_t\mathbf{K} & \partial_t\boldsymbol{\partial}_s\mathbf{K}^{\mathrm{T}} & \partial_t\widetilde{\boldsymbol{\partial}_s}^{2}\mathbf{K}^{\mathrm{T}} & \partial_t^2\mathbf{K} & \partial_t^2\boldsymbol{\partial}_s\mathbf{K}^{\mathrm{T}} & \partial_t^2\widetilde{\boldsymbol{\partial}_s}^{2}\mathbf{K}^{\mathrm{T}} \\
-\boldsymbol{\partial}_s\mathbf{K} & {\color{blue}-\boldsymbol{\partial}_s^2\mathbf{K}_{2\times2}} & -\widetilde{\boldsymbol{\partial}_s}^{3}\mathbf{K}_{2\times3} & -\partial_t\boldsymbol{\partial}_s\mathbf{K}^{\mathrm{T}}_{2\times1} & -\partial_t\boldsymbol{\partial}_s^2\mathbf{K}^{\mathrm{T}}_{2\times2} & -\partial_t\widetilde{\boldsymbol{\partial}_s}^{3}\mathbf{K}^{\mathrm{T}}_{2\times3} & -\partial_t^2\boldsymbol{\partial}_s\mathbf{K}^{\mathrm{T}}_{2\times1} & {\color{blue}-\partial_t^2\boldsymbol{\partial}_s^2\mathbf{K}^{\mathrm{T}}_{2\times2}} & -\partial_t^2\widetilde{\boldsymbol{\partial}_s}^{3}\mathbf{K}^{\mathrm{T}}_{2\times3} \\
\widetilde{\boldsymbol{\partial}_s}^{2}\mathbf{K} & \widetilde{\boldsymbol{\partial}_s}^{3}\mathbf{K}_{3\times2} & {\color{blue}\widetilde{\boldsymbol{\partial}_s}^{4}\mathbf{K}_{3\times3}} & \partial_t\widetilde{\boldsymbol{\partial}_s}^{2}\mathbf{K}_{3\times1} & \partial_t\widetilde{\boldsymbol{\partial}_s}^{3}\mathbf{K}^{\mathrm{T}}_{3\times2} & \partial_t\widetilde{\boldsymbol{\partial}_s}^{4}\mathbf{K}^{\mathrm{T}}_{3\times3} & {\color{blue}\partial_t^2\widetilde{\boldsymbol{\partial}_s}^{2}\mathbf{K}^{\mathrm{T}}_{3\times1}} & \partial_t^2\widetilde{\boldsymbol{\partial}_s}^{3}\mathbf{K}^{\mathrm{T}}_{3\times2} & {\color{blue}\partial_t^2\widetilde{\boldsymbol{\partial}_s}^{4}\mathbf{K}^{\mathrm{T}}_{3\times3}} \\
\hline
-\partial_t K & -\partial_t\boldsymbol{\partial}_s\mathbf{K}_{1\times2} & -\partial_t\widetilde{\boldsymbol{\partial}_s}^{2}\mathbf{K}^{\mathrm{T}}_{1\times3} & {\color{blue}-\partial_t^2\mathbf{K}_{1\times1}} & -\partial_t^2\boldsymbol{\partial}_s\mathbf{K}^{\mathrm{T}}_{1\times2} & {\color{blue}-\partial_t^2\widetilde{\boldsymbol{\partial}_s}^{2}\mathbf{K}^{\mathrm{T}}_{1\times3}} & -\partial_t^3\mathbf{K}_{1\times1} & -\partial_t^3\boldsymbol{\partial}_s\mathbf{K}^{\mathrm{T}}_{1\times2} & -\partial_t^3\widetilde{\boldsymbol{\partial}_s}^{2}\mathbf{K}^{\mathrm{T}}_{1\times3} \\
\partial_t\boldsymbol{\partial}_s\mathbf{K} & \partial_t\boldsymbol{\partial}_s^2\mathbf{K}_{2\times2} & \partial_t\widetilde{\boldsymbol{\partial}_s}^{3}\mathbf{K}_{2\times3} & \partial_t^2\boldsymbol{\partial}_s\mathbf{K}_{2\times1} & {\color{blue}\partial_t^2\boldsymbol{\partial}_s^2\mathbf{K}_{2\times2}} & \partial_t^2\widetilde{\boldsymbol{\partial}_s}^{3}\mathbf{K}^{\mathrm{T}}_{2\times3} & \partial_t^3\boldsymbol{\partial}_s\mathbf{K}^{\mathrm{T}}_{2\times1} & \partial_t^3\boldsymbol{\partial}_s^2\mathbf{K}^{\mathrm{T}}_{2\times2} & \partial_t^3\widetilde{\boldsymbol{\partial}_s}^{3}\mathbf{K}^{\mathrm{T}}_{2\times3} \\
-\partial_t\widetilde{\boldsymbol{\partial}_s}^{2}\mathbf{K} & -\partial_t\widetilde{\boldsymbol{\partial}_s}^{3}\mathbf{K}_{3\times2} & -\partial_t\widetilde{\boldsymbol{\partial}_s}^{4}\mathbf{K}_{3\times3} & {\color{blue}-\partial_t^2\widetilde{\boldsymbol{\partial}_s}^{2}\mathbf{K}_{3\times1}} & -\partial_t^2\widetilde{\boldsymbol{\partial}_s}^{3}\mathbf{K}_{3\times2} & {\color{blue}-\partial_t^2\widetilde{\boldsymbol{\partial}_s}^{4}\mathbf{K}_{3\times3}} & -\partial_t^3\widetilde{\boldsymbol{\partial}_s}^{2}\mathbf{K}_{3\times1} & -\partial_t^3\widetilde{\boldsymbol{\partial}_s}^{3}\mathbf{K}^{\mathrm{T}}_{3\times2} & -\partial_t^3\widetilde{\boldsymbol{\partial}_s}^{4}\mathbf{K}^{\mathrm{T}}_{3\times3} \\
\hline
\partial_t^2\mathbf{K} & \partial_t^2\boldsymbol{\partial}_s\mathbf{K}_{1\times2} & {\color{blue}\partial_t^2\widetilde{\boldsymbol{\partial}_s}^{2}\mathbf{K}_{1\times3}} & \partial_t^3\mathbf{K}_{1\times1} & \partial_t^3\boldsymbol{\partial}_s\mathbf{K}_{1\times2} & \partial_t^3\widetilde{\boldsymbol{\partial}_s}^{2}\mathbf{K}^{\mathrm{T}}_{1\times3} & {\color{blue}\partial_t^4\mathbf{K}_{1\times1}} & \partial_t^4\boldsymbol{\partial}_s\mathbf{K}^{\mathrm{T}}_{1\times2} & {\color{blue}\partial_t^4\widetilde{\boldsymbol{\partial}_s}^{2}\mathbf{K}^{\mathrm{T}}_{1\times3}} \\
-\partial_t^2\boldsymbol{\partial}_s\mathbf{K} & {\color{blue}-\partial_t^2\boldsymbol{\partial}_s^2\mathbf{K}_{2\times2}} & -\partial_t^2\widetilde{\boldsymbol{\partial}_s}^{3}\mathbf{K}_{2\times3} & -\partial_t^3\boldsymbol{\partial}_s\mathbf{K}_{2\times1} & -\partial_t^3\boldsymbol{\partial}_s^2\mathbf{K}_{2\times2} & -\partial_t^2\widetilde{\boldsymbol{\partial}_s}^{3}\mathbf{K}_{2\times3} & -\partial_t^4\boldsymbol{\partial}_s\mathbf{K}_{2\times1} & {\color{blue}-\partial_t^4\boldsymbol{\partial}_s^2\mathbf{K}_{2\times2}} & -\partial_t^4\widetilde{\boldsymbol{\partial}_s}^{3}\mathbf{K}^{\mathrm{T}}_{2\times3} \\
\partial_t^2\widetilde{\boldsymbol{\partial}_s}^{2}\mathbf{K} & \partial_t^2\widetilde{\boldsymbol{\partial}_s}^{3}\mathbf{K}_{3\times2} & {\color{blue}\partial_t^2\widetilde{\boldsymbol{\partial}_s}^{4}\mathbf{K}_{3\times3}} & \partial_t^3\widetilde{\boldsymbol{\partial}_s}^{2}\mathbf{K}_{3\times1} & \partial_t^3\widetilde{\boldsymbol{\partial}_s}^{3}\mathbf{K}_{3\times2} & \partial_t^3\widetilde{\boldsymbol{\partial}_s}^{4}\mathbf{K}_{3\times3} & {\color{blue}\partial_t^4\widetilde{\boldsymbol{\partial}_s}^{2}\mathbf{K}_{3\times1}} & \partial_t^4\widetilde{\boldsymbol{\partial}_s}^{3}\mathbf{K}_{3\times2} & {\color{blue}\partial_t^4\widetilde{\boldsymbol{\partial}_s}^{4}\mathbf{K}_{3\times3}}
\end{array}\right], \quad \text{(S3)}
$$

where $\mathrm{Var}\boldsymbol{\partial}_s Z(\boldsymbol{s},t) = \mathrm{Cov}\{\boldsymbol{\partial}_s Z(\boldsymbol{s},t), \boldsymbol{\partial}_s Z(\boldsymbol{s}',t')\} = \boldsymbol{\partial}_s^2\mathbf{K}(\boldsymbol{\Delta},\delta) = \mathbf{H}_K^{11}(\boldsymbol{\Delta},\delta)$ is the variance of $\boldsymbol{\partial}_s Z(\boldsymbol{s},t)$ of order $d\times d$, $\mathrm{Cov}\{\boldsymbol{\partial}_s Z(\boldsymbol{s},t), \widetilde{\boldsymbol{\partial}_s}^{2} Z(\boldsymbol{s}',t')\} = \widetilde{\boldsymbol{\partial}_s}^{3}\mathbf{K}(\boldsymbol{\Delta},\delta) = \mathbf{H}_K^{12}(\boldsymbol{\Delta},\delta) = \mathbf{H}_K^{21^{\mathrm{T}}}(\boldsymbol{\Delta},\delta)$ is of order $d\times\frac{d(d+1)}{2}$ and $\mathrm{Var}\widetilde{\boldsymbol{\partial}_s}^{2}Z(\boldsymbol{s},t) = \mathrm{Cov}\{\widetilde{\boldsymbol{\partial}_s}^{2}Z(\boldsymbol{s},t), \widetilde{\boldsymbol{\partial}_s}^{2}Z(\boldsymbol{s}',t')\} = \widetilde{\boldsymbol{\partial}_s}^{4}\mathbf{K}(\boldsymbol{\Delta},\delta) = \mathbf{H}_K^{22}(\boldsymbol{\Delta},\delta)$ of order, $\frac{d(d+1)}{2}\times\frac{d(d+1)}{2}$. The entries that remain non-zero as $(\boldsymbol{\Delta},\delta)\to(\mathbf{0}_d,0)$ in the $\left(d+\frac{d(d+1)}{2}\right)\times\left(d+\frac{d(d+1)}{2}\right)$ sub-matrix corresponding to $\mathrm{Cov}\{\boldsymbol{\mathcal{L}}_4^{-Z}Z(\boldsymbol{s},t), \boldsymbol{\mathcal{L}}_4^{-Z}Z(\boldsymbol{s}',t')\}$ are marked in blue. In comparison to the spatiotemporal gradient theory (see, Quick et al. 2015, p. 577), as $(\boldsymbol{\Delta},\delta)\to(\mathbf{0}_d,0)$, the resulting cross-covariance matrix is no longer diagonal.

Spatiotemporal Divergence & Laplacian Using Corollary 1 we are able to devise inference for common differential geometric operators that capture change in geometry for spatiotemporal surfaces. We consider two quantities–divergence and Laplacian. We define *spatiotemporal divergence* as $\mathrm{div}Z(\boldsymbol{s},t) = \left(\mathbf{1}^{\mathrm{T}}\boldsymbol{\partial}_s Z(\boldsymbol{s},t), \mathbf{1}^{\mathrm{T}}\partial_t\boldsymbol{\partial}_s Z(\boldsymbol{s},t), \mathbf{1}^{\mathrm{T}}\partial_t^2\boldsymbol{\partial}_s Z(\boldsymbol{s},t)\right)^{\mathrm{T}}$. Assume $d=2$. First, observe that $\begin{pmatrix}\boldsymbol{\partial}_s Z(\boldsymbol{s},t)\\ \partial_t\boldsymbol{\partial}_s Z(\boldsymbol{s},t)\\ \partial_t^2\boldsymbol{\partial}_s Z(\boldsymbol{s},t)\end{pmatrix} = (\mathbf{I}_2\oplus\mathbf{O}_4\oplus\mathbf{I}_2\oplus\mathbf{O}_4\oplus\mathbf{I}_2\oplus\mathbf{O}_3)\boldsymbol{\mathcal{L}}^{*}Z(\boldsymbol{s},t) = \mathbf{E}_{\mathrm{div}}\boldsymbol{\mathcal{L}}^{*}Z(\boldsymbol{s},t)$ forms a subset of $\boldsymbol{\mathcal{L}}^{*}Z(\boldsymbol{s},t)$ and is therefore is a zero-centered GP with cross-covariance, $\mathbf{E}_{\mathrm{div}}\mathbf{V}_{\boldsymbol{\mathcal{L}}^{*}}(\boldsymbol{\Delta},\delta)\mathbf{E}^{\mathrm{T}}_{\mathrm{div}}$. This is obtained using Corollary 1, part (iii). Hence,

$(\mathbf{1}^\top \oplus \mathbf{1}^\top \oplus \mathbf{1}^\top)\mathbf{E}_{\text{div}}\boldsymbol{\mathcal{L}}_* Z(\boldsymbol{s},t)$ is also a zero-centered GP with cross-covariance, $(\mathbf{1}^\top \oplus \mathbf{1}^\top \oplus \mathbf{1}^\top)\mathbf{E}_{\text{div}}\mathbf{V}_{\boldsymbol{\mathcal{L}}_*}(\boldsymbol{\Delta},\delta)\left((\mathbf{1}^\top \oplus \mathbf{1}^\top \oplus \mathbf{1}^\top)\mathbf{E}_{\text{div}}\right)^\top$. We define the *spatiotemporal Laplacian* as, $\text{Lap}Z(\boldsymbol{s},t) = \left(\sum_{i=1}^d \frac{\partial^2}{\partial s_i^2} Z(\boldsymbol{s},t), \sum_{i=1}^d \frac{\partial}{\partial t}\frac{\partial^2}{\partial s_i^2} Z(\boldsymbol{s},t), \sum_{i=1}^d \frac{\partial^2}{\partial t^2}\frac{\partial^2}{\partial s_i^2} Z(\boldsymbol{s},t)\right)^\top$. Similar arguments relying on Corollary 1 (part (iii)) allows $\text{Lap}Z(\boldsymbol{s},t)$ to be a GP. We show the resulting figures in Section S18.2. Next, we show details for $\mathbf{V}_{Z,\boldsymbol{\mathcal{L}}^* Z}(\boldsymbol{\Delta},\delta)$.

S5 Simplifications under isotropy

As is evident from eq. (S3) inference for spatiotemporal gradients (and directional spatiotemporal gradients) requires the derivatives of $K(\boldsymbol{\Delta},\delta)$. Commonly used covariance kernels are isotropic (see Section S12), i.e. $K(\boldsymbol{\Delta},\delta) = \widetilde{K}(\|\boldsymbol{\Delta}\|, |\delta|)$. We derive simplifications assuming isotropy—first considering the resulting expressions for spatiotemporal gradients followed by the same for directional spatiotemporal gradients.

S5.1 Spatiotemporal Gradients

Proposition 2. *Under isotropy, $K = K(\boldsymbol{\Delta},\delta) = \widetilde{K}(\|\boldsymbol{\Delta}\|, |\delta|) = \widetilde{K}$, simplifications arise in the resulting expressions for derivatives of the covariance. They are as follows,*

$$\boldsymbol{\partial}_{\boldsymbol{s}} K = d_{\|\boldsymbol{\Delta}\|}\widetilde{K}\frac{\mathbf{P}_{1,1}}{\|\boldsymbol{\Delta}\|}, \partial_t K = d_{|\delta|}\widetilde{K}\frac{\delta}{|\delta|}, \boldsymbol{\partial}_{\boldsymbol{s}}^2 K = d_{\|\boldsymbol{\Delta}\|}\widetilde{K}\frac{\mathbf{P}_{2,1}}{\|\boldsymbol{\Delta}\|} + \left(d_{\|\boldsymbol{\Delta}\|}^2\widetilde{K} - \frac{d_{\|\boldsymbol{\Delta}\|}\widetilde{K}}{\|\boldsymbol{\Delta}\|}\right)\frac{\mathbf{P}_{2,2}}{\|\boldsymbol{\Delta}\|^2},$$

$$\partial_t\boldsymbol{\partial}_{\boldsymbol{s}} K = d_{|\delta|}d_{\|\boldsymbol{\Delta}\|}\widetilde{K}\frac{\mathbf{P}_{1,1}}{\|\boldsymbol{\Delta}\|}\frac{\delta}{|\delta|}, \partial_t^2 K = d_{|\delta|}^2\widetilde{K},$$

$$\partial_t\boldsymbol{\partial}_{\boldsymbol{s}}^2 K = d_{|\delta|}d_{\|\boldsymbol{\Delta}\|}\widetilde{K}\frac{\mathbf{P}_{2,1}}{\|\boldsymbol{\Delta}\|}\frac{\delta}{|\delta|} + \left(d_{|\delta|}d_{\|\boldsymbol{\Delta}\|}^2\widetilde{K} - \frac{d_{|\delta|}d_{\|\boldsymbol{\Delta}\|}\widetilde{K}}{\|\boldsymbol{\Delta}\|}\right)\frac{\mathbf{P}_{2,2}}{\|\boldsymbol{\Delta}\|^2}\frac{\delta}{|\delta|}, \partial_t^2\boldsymbol{\partial}_{\boldsymbol{s}} K = d_{|\delta|}^2 d_{\|\boldsymbol{\Delta}\|}\widetilde{K}\frac{\mathbf{P}_{1,1}}{\|\boldsymbol{\Delta}\|},$$

$$\boldsymbol{\partial}_{\boldsymbol{s}}^4 K = \left(d_{\|\boldsymbol{\Delta}\|}^2\widetilde{K} - \frac{d_{\|\boldsymbol{\Delta}\|}\widetilde{K}}{\|\boldsymbol{\Delta}\|}\right)\left(-3\frac{\mathbf{P}_{4,1}}{\|\boldsymbol{\Delta}\|^4} + \frac{\mathbf{P}_{4,2}}{\|\boldsymbol{\Delta}\|^2} + 15\frac{\mathbf{P}_{4,3}}{\|\boldsymbol{\Delta}\|^6} - 3\frac{\mathbf{P}_{4,4}}{\|\boldsymbol{\Delta}\|^4}\right) + d_{\|\boldsymbol{\Delta}\|}^3\widetilde{K}\left(\frac{\mathbf{P}_{4,1}}{\|\boldsymbol{\Delta}\|^3} - 6\frac{\mathbf{P}_{4,3}}{\|\boldsymbol{\Delta}\|^5} + \frac{\mathbf{P}_{4,4}}{\|\boldsymbol{\Delta}\|^3}\right) + d_{\|\boldsymbol{\Delta}\|}^4\widetilde{K}\frac{\mathbf{P}_{4,3}}{\|\boldsymbol{\Delta}\|^4},$$

$$\partial_t^2\boldsymbol{\partial}_{\boldsymbol{s}}^2 K = d_{|\delta|}^2 d_{\|\boldsymbol{\Delta}\|}\widetilde{K}\frac{\mathbf{P}_{2,1}}{\|\boldsymbol{\Delta}\|} + \left(d_{|\delta|}^2 d_{\|\boldsymbol{\Delta}\|}^2\widetilde{K} - \frac{d_{|\delta|}^2 d_{\|\boldsymbol{\Delta}\|}\widetilde{K}}{\|\boldsymbol{\Delta}\|}\right)\frac{\mathbf{P}_{2,2}}{\|\boldsymbol{\Delta}\|^2},\ \partial_t^4 K = d_{|\delta|}^4\widetilde{K},$$

$$\partial_t^2\boldsymbol{\partial}_{\boldsymbol{s}}^4 K = \left(d_{|\delta|}^2 d_{\|\boldsymbol{\Delta}\|}^2\widetilde{K} - \frac{d_{|\delta|}^2 d_{\|\boldsymbol{\Delta}\|}\widetilde{K}}{\|\boldsymbol{\Delta}\|}\right)\left(-3\frac{\mathbf{P}_{4,1}}{\|\boldsymbol{\Delta}\|^4} + \frac{\mathbf{P}_{4,2}}{\|\boldsymbol{\Delta}\|^2} + 15\frac{\mathbf{P}_{4,3}}{\|\boldsymbol{\Delta}\|^6} - 3\frac{\mathbf{P}_{4,4}}{\|\boldsymbol{\Delta}\|^4}\right) + d_{|\delta|}^2 d_{\|\boldsymbol{\Delta}\|}^3\widetilde{K}\left(\frac{\mathbf{P}_{4,1}}{\|\boldsymbol{\Delta}\|^3} - 6\frac{\mathbf{P}_{4,3}}{\|\boldsymbol{\Delta}\|^5} + \frac{\mathbf{P}_{4,4}}{\|\boldsymbol{\Delta}\|^3}\right) + d_{|\delta|}^2 d_{\|\boldsymbol{\Delta}\|}^4\widetilde{K}\frac{\mathbf{P}_{4,3}}{\|\boldsymbol{\Delta}\|^4},$$

$$\partial_t^4\boldsymbol{\partial}_{\boldsymbol{s}}^2 K = \left\{d_{|\delta|}^4 d_{\|\boldsymbol{\Delta}\|}\widetilde{K}\frac{\mathbf{P}_{2,1}}{\|\boldsymbol{\Delta}\|} + \left(d_{|\delta|}^4 d_{\|\boldsymbol{\Delta}\|}^2\widetilde{K} - \frac{d_{|\delta|}^4 d_{\|\boldsymbol{\Delta}\|}\widetilde{K}}{\|\boldsymbol{\Delta}\|}\right)\frac{\mathbf{P}_{2,2}}{\|\boldsymbol{\Delta}\|^2}\right\},$$

$$\partial_t^4\boldsymbol{\partial}_{\boldsymbol{s}}^4 K = \left(d_{|\delta|}^4 d_{\|\boldsymbol{\Delta}\|}^2\widetilde{K} - \frac{d_{|\delta|}^4 d_{\|\boldsymbol{\Delta}\|}\widetilde{K}}{\|\boldsymbol{\Delta}\|}\right)\left(-3\frac{\mathbf{P}_{4,1}}{\|\boldsymbol{\Delta}\|^4} + \frac{\mathbf{P}_{4,2}}{\|\boldsymbol{\Delta}\|^2} + 15\frac{\mathbf{P}_{4,3}}{\|\boldsymbol{\Delta}\|^6} - 3\frac{\mathbf{P}_{4,4}}{\|\boldsymbol{\Delta}\|^4}\right) + d_{|\delta|}^4 d_{\|\boldsymbol{\Delta}\|}^3\widetilde{K}\left(\frac{\mathbf{P}_{4,1}}{\|\boldsymbol{\Delta}\|^3} - 6\frac{\mathbf{P}_{4,3}}{\|\boldsymbol{\Delta}\|^5} + \frac{\mathbf{P}_{4,4}}{\|\boldsymbol{\Delta}\|^3}\right) + d_{|\delta|}^4 d_{\|\boldsymbol{\Delta}\|}^4\widetilde{K}\frac{\mathbf{P}_{4,3}}{\|\boldsymbol{\Delta}\|^4},$$

where $\mathbf{P}_{1,1} = \boldsymbol{\Delta}$, $\mathbf{P}_{2,1} = \mathrm{vec}\mathbf{I}_d$, $\mathbf{P}_{2,2} = (\mathbf{I}_d \otimes \boldsymbol{\Delta})\boldsymbol{\Delta}$, $\mathbf{P}_{3,1} = \mathbf{P}_{3,11} + \mathbf{P}_{3,12} + \mathbf{P}_{3,13}$, $\mathbf{P}_{3,11} = \boldsymbol{\Delta} \otimes \mathrm{vec}\mathbf{I}_d$, $\mathbf{P}_{3,12} = (\mathbf{I}_d \otimes \mathbf{U}_2)(\mathrm{vec}\mathbf{I}_d \otimes \mathbf{I}_d)\mathbf{U}_1\boldsymbol{\Delta}$, $\mathbf{P}_{3,13} = \mathbf{I}_d \otimes (\mathbf{I}_d \otimes \boldsymbol{\Delta})\mathrm{vec}\mathbf{I}_d$, $\mathbf{P}_{3,2} = \boldsymbol{\Delta} \otimes (\mathbf{I}_d \otimes \boldsymbol{\Delta})\boldsymbol{\Delta}$, $\mathbf{P}_{4,1} = \boldsymbol{\Delta} \otimes \mathbf{P}_{3,1}$, $\mathbf{P}_{4,2} = \mathbf{P}_{4,21} + \mathbf{P}_{4,22} + \mathbf{P}_{4,23}$, $\mathbf{P}_{4,21} = \mathrm{vec}\mathbf{I}_d \otimes \mathrm{vec}\mathbf{I}_d$, $\mathbf{P}_{4,22} = (\mathbf{I}_d \otimes (\mathbf{I}_d \otimes \mathbf{U}_2)(\mathrm{vec}\mathbf{I}_d \otimes \mathbf{I}_d)\mathbf{U}_1)\,\mathrm{vec}\mathbf{I}_d$, $\mathbf{P}_{4,23} = (\mathbf{I}_d \otimes \mathbf{U}_3)\left\{((\mathbf{I}_d \otimes \mathbf{U}_2)(\mathrm{vec}\mathbf{I}_d \otimes \mathbf{I}_d)\mathbf{U}_1) \otimes \mathbf{I}_d\right\}\mathbf{U}_2\mathrm{vec}\mathbf{I}_d$, $\mathbf{P}_{4,3} = \boldsymbol{\Delta} \otimes \mathbf{P}_{3,2}$ *and* $\mathbf{P}_{4,4} = [\mathrm{vec}\mathbf{I}_d \otimes (\mathbf{I}_d \otimes \boldsymbol{\Delta}) + (\mathbf{I}_d \otimes \mathbf{U}_3)\left\{((\mathbf{I}_d \otimes \mathbf{U}_2)(\mathrm{vec}\mathbf{I}_d \otimes \mathbf{I}_d)\mathbf{U}_1) \otimes \boldsymbol{\Delta}\right\}\mathbf{U}_1]\boldsymbol{\Delta} + \{\mathbf{I}_d \otimes (\boldsymbol{\Delta} \otimes (\mathbf{I}_d \otimes \boldsymbol{\Delta}))\}\,\mathrm{vec}\mathbf{I}_d$, $\mathbf{U}_i$ *is a permutation matrix of order* $d^i \times d^i$, $i = 1,2,3$ *and* $\mathbf{P}_{i,\cdot}$ *is a* $d^i \times 1$ *vector for* $i = 1,2,3,4$. *The operators,* $d_{\|\boldsymbol{\Delta}\|}^r = \frac{d^r}{d\|\boldsymbol{\Delta}\|^r}$ *and* $d_{|\delta|}^r = \frac{d^r}{d|\delta|^r}$.

Proof. Note that $\frac{\partial}{\partial\delta}\frac{\delta}{|\delta|} = \left(\frac{1}{|\delta|} - \frac{\delta}{|\delta|^2}\frac{\delta}{|\delta|}\right) = 0$, $\frac{\partial}{\partial\boldsymbol{\Delta}}\frac{d_{\|\boldsymbol{\Delta}\|}^r\widetilde{K}}{\|\boldsymbol{\Delta}\|^r} = \left(d_{\|\boldsymbol{\Delta}\|}^{r+1}\widetilde{K} - \frac{d_{\|\boldsymbol{\Delta}\|}^r\widetilde{K}}{\|\boldsymbol{\Delta}\|}\right)\frac{\boldsymbol{\Delta}}{\|\boldsymbol{\Delta}\|^{r+1}} - (r-1)\frac{d_{\|\boldsymbol{\Delta}\|}^r\widetilde{K}}{\|\boldsymbol{\Delta}\|^r}\frac{\boldsymbol{\Delta}}{\|\boldsymbol{\Delta}\|^2}$, and for $r, r' > 0$, $\frac{\partial}{\partial\boldsymbol{\Delta}}\left(d_{\|\boldsymbol{\Delta}\|}^{r+1}\widetilde{K} - \frac{d_{\|\boldsymbol{\Delta}\|}^r\widetilde{K}}{\|\boldsymbol{\Delta}\|}\right)\frac{1}{\|\boldsymbol{\Delta}\|^{r'}} = \left\{\frac{d_{\|\boldsymbol{\Delta}\|}^{r+2}\widetilde{K}}{\|\boldsymbol{\Delta}\|^{r'+1}} - \left(d_{\|\boldsymbol{\Delta}\|}^{r+1}\widetilde{K} - \frac{d_{\|\boldsymbol{\Delta}\|}^r\widetilde{K}}{\|\boldsymbol{\Delta}\|}\right)\frac{r'+1}{\|\boldsymbol{\Delta}\|^{r'+2}}\right\}\boldsymbol{\Delta}$. The resulting expressions under isotropy are obtained via successive differentiation.

$\square$

Deriving the expressions above requires computing the derivatives of matrices with respect to vectors. In the traditional sense, this results in tensors, for example, $\frac{\partial}{\partial \boldsymbol{\Delta}}\boldsymbol{\partial}_{\boldsymbol{s}}^2 K(\boldsymbol{\Delta},\delta)$ produces a 3-order tensor. We use notation in Vetter (1970) (also, see, e.g., Graham 2018, Chapter 6) to obtain vector representations for tensor-valued derivatives. The order of listing conforms to the reshaping of tensors discussed in Section S1. We rely on the following, $\frac{\partial \boldsymbol{\Delta}}{\partial \boldsymbol{\Delta}} = (\boldsymbol{e}_1^{\mathrm{T}}, \boldsymbol{e}_2^{\mathrm{T}}, \ldots, \boldsymbol{e}_d^{\mathrm{T}})^{\mathrm{T}} = \mathrm{vec}(\mathbf{I}_d)$, $\frac{\partial \mathbf{AB}}{\partial \boldsymbol{\Delta}} = \frac{\partial \mathbf{A}}{\partial \boldsymbol{\Delta}}(\mathbf{1}\otimes\mathbf{B}) + (\mathbf{I}_d \otimes \mathbf{A})\frac{\partial \mathbf{B}}{\partial \boldsymbol{\Delta}}$ and $\frac{\partial \mathbf{A}\otimes\mathbf{B}}{\partial \boldsymbol{\Delta}} = \frac{\partial \mathbf{A}}{\partial \boldsymbol{\Delta}} \otimes \mathbf{B} + (\mathbf{I}_d \otimes \mathbf{U}_1)\left(\frac{\partial \mathbf{B}}{\partial \boldsymbol{\Delta}}\otimes \mathbf{A}\right)(\mathbf{1}\otimes \mathbf{U}_2)$. In particular, when deriving $\boldsymbol{\partial}_{\boldsymbol{s}}^3 K$, we use $\frac{\partial}{\partial \boldsymbol{\Delta}} \frac{d_{\|\boldsymbol{\Delta}\|}\widetilde{K}}{\|\boldsymbol{\Delta}\|}\mathrm{vec}\mathbf{I}_d = \left(d^2_{\|\boldsymbol{\Delta}\|}\widetilde{K} - \frac{d_{\|\boldsymbol{\Delta}\|}\widetilde{K}}{\|\boldsymbol{\Delta}\|}\right)\frac{\boldsymbol{\Delta}}{\|\boldsymbol{\Delta}\|^2}\otimes \mathrm{vec}\mathbf{I}_d$. The vectors $\boldsymbol{e}_i$, $i = 1,\ldots,d$ constitute the orthonormal basis for $\Re^d$.

S5.2 Directional Spatiotemporal Gradients

Directional spatiotemporal gradients and curvatures involve a direction vector of interest (see Section 2.1 of the manuscript), $(\boldsymbol{u}^{\mathrm{T}}, v)^{\mathrm{T}} \in \mathfrak{H}^{d+1} = \Re^d \times \Re^+$ along which the gradients and curvatures are evaluated. For convenience, we fix $v = 1$. We require the covariance between two directional differential processes of possibly different orders. Let $\boldsymbol{\Delta}(h_s) = \boldsymbol{\Delta} + h_s\boldsymbol{u}$ and $\delta(h_t) = \delta + h_t$. Suppressing dependence on $(\boldsymbol{\Delta},\delta)$, we write $g(h_s,h_t) = K(\boldsymbol{\Delta}(h_s),\delta(h_t)) = K(\boldsymbol{\Delta} + h_s\boldsymbol{u}, \delta + h_t)$. The following result derives the required covariance.

Proposition 3. *Let $\boldsymbol{u}^{\otimes(r_1-j_1)^{\mathrm{T}}}\partial_t^{j_1}\boldsymbol{\partial}_{\boldsymbol{s}}^{r_1-j_1}Z(\boldsymbol{s},t)$ and $\boldsymbol{u}^{\otimes(r_2-j_2)^{\mathrm{T}}}\partial_t^{j_2}\boldsymbol{\partial}_{\boldsymbol{s}}^{r_2-j_2}Z(\boldsymbol{s}',t')$ be two directional differential processes of orders r_1 and r_2 respectively. The covariance,*

$$\mathrm{Cov}\left(\boldsymbol{u}^{\otimes(r_1-j_1)^{\mathrm{T}}}\partial_t^{j_1}\boldsymbol{\partial}_{\boldsymbol{s}}^{r_1-j_1}Z(\boldsymbol{s},t), \boldsymbol{u}^{\otimes(r_2-j_2)^{\mathrm{T}}}\partial_t^{j_2}\boldsymbol{\partial}_{\boldsymbol{s}}^{r_2-j_2}Z(\boldsymbol{s}',t')\right) = (-1)^{r_1}\partial_t^{j_1+j_2}\boldsymbol{\partial}_{\boldsymbol{s}}^{(r_1+r_2-j_1-j_2)}g(\mathbf{0}_d,0),$$

for every $j_i = 0,\ldots,r_i$ and $i = 1,2$.

Proof. Following Proposition 1, we express the covariance between the directional differential processes as a limit of the covariance between the finite difference process,

$$\mathrm{Cov}\left(\boldsymbol{u}^{\otimes(r_1-j_1)^{\mathrm{T}}}\partial_t^{j_1}\boldsymbol{\partial}_{\boldsymbol{s}}^{r_1-j_1}Z(\boldsymbol{s},t),\boldsymbol{u}^{\otimes(r_2-j_2)^{\mathrm{T}}}\partial_t^{j_2}\boldsymbol{\partial}_{\boldsymbol{s}}^{r_2-j_2}Z(\boldsymbol{s}',t')\right)=\lim_{\substack{k\to0\\h\to0}}\mathrm{Cov}\left(Z_{k,\boldsymbol{u},1}^{(j_1,r_1)}(\boldsymbol{s},t),\ Z_{h,\boldsymbol{u},1}^{(j_2,r_2)}(\boldsymbol{s}',t')\right),$$

where $Z_{k,\boldsymbol{u},1}^{(j_1,r_1)}(\boldsymbol{s},t)=k^{-r_1}(D_{k,\boldsymbol{u},0}-1)^{r_1-j_1}(D_{k,0,1}-1)^{j_1}Z(\boldsymbol{s},t)$, $Z_{h,\boldsymbol{u},1}^{(j_2,r_2)}(\boldsymbol{s}',t')=h^{-r_2}(D_{h,\boldsymbol{u},0}-1)^{r_2-j_2}(D_{h,0,1}-1)^{j_2}Z(\boldsymbol{s}',t')$. Using these expressions and performing binomial expansion

$$\begin{aligned}
&\lim_{\substack{k\to0\\h\to0}}\mathrm{Cov}\left(Z_{k,\boldsymbol{u},1}^{(j_1,r_1)}(\boldsymbol{s},t),\ Z_{h,\boldsymbol{u},1}^{(j_2,r_2)}(\boldsymbol{s}',t')\right)=\\
&\lim_{\substack{k\to0\\h\to0}}\ k^{-r_1}\ h^{-r_2}\ \mathrm{Cov}\left(\sum_{i=1}^{r_1-j_1}\sum_{j=1}^{j_1}\binom{r_1-j_1}{i}\binom{j_1}{j}(-1)^{r_1-(i+j)}\ Z(\boldsymbol{s}+ik\boldsymbol{u},t+jk),\right.\\
&\qquad\left.\sum_{i'=1}^{r_2-j_2}\sum_{j'=1}^{j_2}\binom{r_2-j_2}{i'}\binom{j_2}{j'}(-1)^{r_2-(i'+j')}\ Z(\boldsymbol{s}'+i'h\boldsymbol{u},t'+j'h)\right),\\
&=(-1)^{r_1}\partial_t^{j_1+j_2}\boldsymbol{\partial}_{\boldsymbol{s}}^{r_1+r_2-(j_1+j_2)}\ g(\mathbf{0}_d,0),
\end{aligned}$$

for every $j_i=0,\ldots,r_i$, $i=1,2$. The last equality is obtained after some algebra. □

We are interested in the directional derivative process required for inference,

$$\boldsymbol{\mathcal{L}}_{\boldsymbol{u},1}^{*}Z(\boldsymbol{s},t)=\left(\boldsymbol{\mathcal{L}}_{\boldsymbol{s},\boldsymbol{u}}Z(\boldsymbol{s},t)^{\mathrm{T}},\partial_tZ(\boldsymbol{s},t),\partial_t\boldsymbol{\mathcal{L}}_{\boldsymbol{s},\boldsymbol{u}}Z(\boldsymbol{s},t)^{\mathrm{T}},\partial_t^2Z(\boldsymbol{s},t),\partial_t^2\boldsymbol{\mathcal{L}}_{\boldsymbol{s},\boldsymbol{u}}Z(\boldsymbol{s},t)^{\mathrm{T}}\right)^{\mathrm{T}},$$

where $\boldsymbol{\mathcal{L}}_{\boldsymbol{s},\boldsymbol{u}}Z(\boldsymbol{s},t)=\left(\boldsymbol{u}^{\mathrm{T}}\boldsymbol{\partial}_{\boldsymbol{s}}Z(\boldsymbol{s},t),\boldsymbol{u}^{\otimes2^{\mathrm{T}}}\boldsymbol{\partial}_{\boldsymbol{s}}^2Z(\boldsymbol{s},t)\right)^{\mathrm{T}}$. Evidently, $\partial_t^4\boldsymbol{\partial}_{\boldsymbol{s}}^4g(\mathbf{0}_d,0)$ should exist and be well-defined. The next proposition addresses this.

Proposition 4. *The covariance between* $\boldsymbol{u}^{\otimes2^{\mathrm{T}}}\partial_t^2\boldsymbol{\partial}_{\boldsymbol{s}}^2Z(\boldsymbol{s},t)$ *and* $\boldsymbol{u}^{\otimes2^{\mathrm{T}}}\partial_t^2\boldsymbol{\partial}_{\boldsymbol{s}}^2Z(\boldsymbol{s}',t')$ *is given by*

$$\begin{aligned}
&\mathrm{Cov}\left(\boldsymbol{u}^{\otimes2^{\mathrm{T}}}\partial_t^2\boldsymbol{\partial}_{\boldsymbol{s}}^2Z(\boldsymbol{s},t),\boldsymbol{u}^{\otimes2^{\mathrm{T}}}\partial_t^2\boldsymbol{\partial}_{\boldsymbol{s}}^2Z(\boldsymbol{s}',t')\right)\\
&=\frac{3}{\|\boldsymbol{\Delta}\|^2}\left(1-5\frac{(\boldsymbol{u}^{\mathrm{T}}\boldsymbol{\Delta})^2}{\|\boldsymbol{\Delta}\|^2}\right)\left(1-\frac{(\boldsymbol{u}^{\mathrm{T}}\boldsymbol{\Delta})^2}{\|\boldsymbol{\Delta}\|^2}\right)\left(\partial_t^4\boldsymbol{\partial}_{\boldsymbol{s}}^2\widetilde{K}(\|\boldsymbol{\Delta}\|,|\delta|)-\frac{\partial_t^4\boldsymbol{\partial}_{\boldsymbol{s}}\widetilde{K}(\|\boldsymbol{\Delta}\|,|\delta|)}{\|\boldsymbol{\Delta}\|}\right)+\\
&\qquad\frac{6}{\|\boldsymbol{\Delta}\|}\frac{(\boldsymbol{u}^{\mathrm{T}}\boldsymbol{\Delta})^2}{\|\boldsymbol{\Delta}\|^2}\left(1-\frac{(\boldsymbol{u}^{\mathrm{T}}\boldsymbol{\Delta})^2}{\|\boldsymbol{\Delta}\|^2}\right)\partial_t^4\boldsymbol{\partial}_{\boldsymbol{s}}^3\widetilde{K}(\|\boldsymbol{\Delta}\|,|\delta|)+\left(\frac{\boldsymbol{u}^{\mathrm{T}}\boldsymbol{\Delta}}{\|\boldsymbol{\Delta}\|}\right)^4\partial_t^4\boldsymbol{\partial}_{\boldsymbol{s}}^4\widetilde{K}(\|\boldsymbol{\Delta}\|,|\delta|),
\end{aligned}$$

where $\boldsymbol{\Delta}=\boldsymbol{s}-\boldsymbol{s}'$ *and* $\delta=t-t'$.

Proof. For convenience, let $\boldsymbol{\Delta}(h_s)=\boldsymbol{\Delta}$, $\delta(h_t)=\delta$, $K(\boldsymbol{\Delta}(h_s),\delta(h_t))=K$ and $g(h_s,h_t)=g$. Successive differentiation yields

$$\boldsymbol{\partial}_{\boldsymbol{s}} g = \boldsymbol{\Delta}'^{\mathrm{T}} \boldsymbol{\partial}_{\boldsymbol{s}} K,\ \partial_t g = \partial_t K \delta',\ \boldsymbol{\partial}^2_{\boldsymbol{s}} g = \boldsymbol{\Delta}''^{\mathrm{T}} \boldsymbol{\partial}_{\boldsymbol{s}} K + \boldsymbol{\Delta}'^{\otimes 2^{\mathrm{T}}} \boldsymbol{\partial}^2_{\boldsymbol{s}} K,$$

$$\partial_t \boldsymbol{\partial}_{\boldsymbol{s}} g = \boldsymbol{\Delta}'^{\mathrm{T}} \partial_t \boldsymbol{\partial}_{\boldsymbol{s}} K \delta', \partial^2_t g = \delta'' \partial_t K + \delta'^2 \partial^2_t K,$$

$$\boldsymbol{\partial}^3_{\boldsymbol{s}} g = \boldsymbol{\Delta}'''^{\mathrm{T}} \boldsymbol{\partial}_{\boldsymbol{s}} K + 3 \left(\boldsymbol{\Delta}'' \otimes \boldsymbol{\Delta}'\right)^{\mathrm{T}} \boldsymbol{\partial}^2_{\boldsymbol{s}} K + \boldsymbol{\Delta}'^{\otimes 3^{\mathrm{T}}} \boldsymbol{\partial}^3_{\boldsymbol{s}} K,\ \partial_t \boldsymbol{\partial}^2_{\boldsymbol{s}} g = \boldsymbol{\Delta}''^{\mathrm{T}} \partial_t \boldsymbol{\partial}_{\boldsymbol{s}} K \delta' + \boldsymbol{\Delta}'^{\otimes 2^{\mathrm{T}}} \partial_t \boldsymbol{\partial}^2_{\boldsymbol{s}} K \delta',$$

$$\partial^2_t \boldsymbol{\partial}_{\boldsymbol{s}} g = \boldsymbol{\Delta}'^{\mathrm{T}} \partial_t \boldsymbol{\partial}_{\boldsymbol{s}} K \delta'' + \boldsymbol{\Delta}'^{\mathrm{T}} \partial^2_t \boldsymbol{\partial}_{\boldsymbol{s}} K \delta'^2,\ \partial^3_t g = \delta''' \partial_t K + 3\delta'' \delta' \partial^2_t K + \delta'^3 \partial^3_t K,$$

$$\boldsymbol{\partial}^4_{\boldsymbol{s}} g = \boldsymbol{\Delta}^{(iv)^{\mathrm{T}}} \boldsymbol{\partial}_{\boldsymbol{s}} K + 4 \left(\boldsymbol{\Delta}''' \otimes \boldsymbol{\Delta}'\right)^{\mathrm{T}} \boldsymbol{\partial}^2_{\boldsymbol{s}} K + 3 \boldsymbol{\Delta}''^{\otimes 2^{\mathrm{T}}} \boldsymbol{\partial}^2_{\boldsymbol{s}} K + 6 \left(\boldsymbol{\Delta}'' \otimes \boldsymbol{\Delta}'^{\otimes 2}\right)^{\mathrm{T}} \boldsymbol{\partial}^3_{\boldsymbol{s}} K + \boldsymbol{\Delta}'^{\otimes 4^{\mathrm{T}}} \boldsymbol{\partial}^4_{\boldsymbol{s}} K,$$

$$\begin{aligned}\partial^4_t \boldsymbol{\partial}^4_{\boldsymbol{s}} g = &\Big\{\boldsymbol{\Delta}^{(iv)^{\mathrm{T}}} \partial_t \boldsymbol{\partial}_{\boldsymbol{s}} K + 4 \left(\boldsymbol{\Delta}''' \otimes \boldsymbol{\Delta}'\right)^{\mathrm{T}} \partial_t \boldsymbol{\partial}^2_{\boldsymbol{s}} K + 3 \boldsymbol{\Delta}''^{\otimes 2^{\mathrm{T}}} \partial_t \boldsymbol{\partial}^2_{\boldsymbol{s}} K + 6 \left(\boldsymbol{\Delta}'' \otimes \boldsymbol{\Delta}'^{\otimes 2}\right)^{\mathrm{T}} \partial_t \boldsymbol{\partial}^3_{\boldsymbol{s}} K + \\ &\boldsymbol{\Delta}'^{\otimes 4^{\mathrm{T}}} \partial_t \boldsymbol{\partial}^4_{\boldsymbol{s}} K\Big\} \delta^{(iv)} + \Big\{\boldsymbol{\Delta}^{(iv)^{\mathrm{T}}} \partial^2_t \boldsymbol{\partial}_{\boldsymbol{s}} K + 4 \left(\boldsymbol{\Delta}''' \otimes \boldsymbol{\Delta}'\right)^{\mathrm{T}} \partial^2_t \boldsymbol{\partial}^2_{\boldsymbol{s}} K + \\ &3 \boldsymbol{\Delta}''^{\otimes 2^{\mathrm{T}}} \partial_t \boldsymbol{\partial}^2_{\boldsymbol{s}} K + 6 \left(\boldsymbol{\Delta}'' \otimes \boldsymbol{\Delta}'^{\otimes 2}\right)^{\mathrm{T}} \partial^2_t \boldsymbol{\partial}^3_{\boldsymbol{s}} K + \boldsymbol{\Delta}'^{\otimes 4^{\mathrm{T}}} \partial^2_t \boldsymbol{\partial}^4_{\boldsymbol{s}} K\Big\} \left(4\delta''' \delta' + 3\delta''^2\right) + \\ &6 \Big\{\boldsymbol{\Delta}^{(iv)^{\mathrm{T}}} \partial^3_t \boldsymbol{\partial}_{\boldsymbol{s}} K + 4 \left(\boldsymbol{\Delta}''' \otimes \boldsymbol{\Delta}'\right)^{\mathrm{T}} \partial^3_t \boldsymbol{\partial}^2_{\boldsymbol{s}} K + 3 \boldsymbol{\Delta}''^{\otimes 2^{\mathrm{T}}} \partial^3_t \boldsymbol{\partial}^2_{\boldsymbol{s}} K + 6 \left(\boldsymbol{\Delta}'' \otimes \boldsymbol{\Delta}'^{\otimes 2}\right)^{\mathrm{T}} \partial^3_t \boldsymbol{\partial}^3_{\boldsymbol{s}} K + \\ &\boldsymbol{\Delta}'^{\otimes 4^{\mathrm{T}}} \partial^3_t \boldsymbol{\partial}^4_{\boldsymbol{s}} K\Big\} \delta'^2 \delta'' + \Big\{\boldsymbol{\Delta}^{(iv)^{\mathrm{T}}} \partial^4_t \boldsymbol{\partial}_{\boldsymbol{s}} K + 4 \left(\boldsymbol{\Delta}''' \otimes \boldsymbol{\Delta}'\right)^{\mathrm{T}} \partial^4_t \boldsymbol{\partial}^2_{\boldsymbol{s}} K + 3 \boldsymbol{\Delta}''^{\otimes 2^{\mathrm{T}}} \partial^4_t \boldsymbol{\partial}^2_{\boldsymbol{s}} K + \\ &6 \left(\boldsymbol{\Delta}'' \otimes \boldsymbol{\Delta}'^{\otimes 2}\right)^{\mathrm{T}} \partial^4_t \boldsymbol{\partial}^3_{\boldsymbol{s}} K + \boldsymbol{\Delta}'^{\otimes 4^{\mathrm{T}}} \partial^4_t \boldsymbol{\partial}^4_{\boldsymbol{s}} K\Big\} \delta'^4,\end{aligned}$$

obtained through successive differentiation. We note that $\boldsymbol{\Delta}(0) = \boldsymbol{\Delta}$, $\delta(0) = \delta$, $\boldsymbol{\Delta}'(h_s) = \boldsymbol{u}$, $\boldsymbol{\Delta}''(h_s) = \boldsymbol{\Delta}'''(h_s) = \boldsymbol{\Delta}^{(iv)}(h_s) = 0$, $\delta'(h_t) = 1$ and $\delta''(h_t) = \delta'''(h_t) = \delta^{(iv)}(h_t) = 0$, for all h_s and h_t. Substituting these values in the expressions derived above, we get $\partial^4_t \boldsymbol{\partial}^4_{\boldsymbol{s}} g(0_d, 0) = \boldsymbol{u}^{\otimes 4^{\mathrm{T}}} \partial^4_t \boldsymbol{\partial}^4_{\boldsymbol{s}} K(0_d, 0)$. Under isotropy, $K(\boldsymbol{\Delta}(h_s), \delta(h_t)) = \widetilde{K}(||\boldsymbol{\Delta}(h_s)||, |\delta(h_t)|)$. We define $\rho_s(h_s) = ||\boldsymbol{\Delta}(h_s)||$, $\rho_t(h_t) = |\delta(h_t)|$, and let $g(h_s, h_t) = \widetilde{K}(\rho_s(h_s), \rho_t(h_t))$. Hence, $\rho'_s(0) = \frac{\boldsymbol{u}^{\mathrm{T}} \boldsymbol{\Delta}}{||\boldsymbol{\Delta}||}$, $\rho''_s(0) = \frac{1}{||\boldsymbol{\Delta}||}\left(1 - \frac{(\boldsymbol{u}^{\mathrm{T}} \boldsymbol{\Delta})^2}{||\boldsymbol{\Delta}||^2}\right)$, $\rho'''_s(0) = -3\frac{\boldsymbol{u}^{\mathrm{T}} \boldsymbol{\Delta}}{||\boldsymbol{\Delta}||^3}\left(1 - \frac{(\boldsymbol{u}^{\mathrm{T}} \boldsymbol{\Delta})^2}{||\boldsymbol{\Delta}||^2}\right)$, and $\rho^{(iv)}_s(0) = -\frac{3}{||\boldsymbol{\Delta}||^3}\left(1 - 5\frac{(\boldsymbol{u}^{\mathrm{T}} \boldsymbol{\Delta})^2}{||\boldsymbol{\Delta}||^2}\right)\left(1 - \frac{(\boldsymbol{u}^{\mathrm{T}} \boldsymbol{\Delta})^2}{||\boldsymbol{\Delta}||^2}\right)$. Similarly, $\rho'_t(0) = \frac{\delta}{|\delta|}$, but $\rho''_t(0) = \rho'''_t(0) = \rho^{(iv)}_t(0) = 0$. On noting that $\rho'_t(0)^4 = 1$ we obtain $\partial^4_t \boldsymbol{\partial}^4_{\boldsymbol{s}} g(0, 0) = \rho^{(iv)}_s(0) \partial^4_t \boldsymbol{\partial}_{\boldsymbol{s}} \widetilde{K} + (4\rho'''_s(0)\rho'_s(0) + 3\rho''_s(0)^2)\, \partial^4_t \boldsymbol{\partial}^2_{\boldsymbol{s}} \widetilde{K} + 6\rho''_s(0)\rho'_s(0)^2 \partial^4_t \boldsymbol{\partial}^3_{\boldsymbol{s}} \widetilde{K} + \rho'_s(0)^4 \partial^4_t \boldsymbol{\partial}^4_{\boldsymbol{s}} \widetilde{K}$, which is the required result. □

Expressions for lower order spatiotemporal derivatives in the above equations are obtained via similar substitutions. In the next discussion we examine covariance kernels that admit the spatiotemporal derivative processes of interest, viz., $\mathcal{L}^* Z(\boldsymbol{s}, t)$ or $\mathcal{L}^*_{n_t, \boldsymbol{n}_s} Z(\boldsymbol{s}, t)$. We investigate separable and non-separable spatiotemporal covariance kernels in search of

a necessary criterion involving spectral moments that ensures the existence of $\mathcal{L}^* Z(\mathbf{s}, t)$.

S6 Spectral densities for spatiotemporal covariance kernels

We turn to spectral theory to characterize these covariance functions. Using Bochner's theorem (see, e.g., Bochner 2005, Rasmussen & Williams 2005), K is a positive-definite covariance function on $\Re^d \times \Re$ if and only if it is of the form $K(\boldsymbol{\Delta}, \delta) = \iint_{\Re^{d+1}} \exp(i\boldsymbol{w}_s^{\mathrm{T}}\boldsymbol{\Delta} + iw_t\delta)\; F(d\boldsymbol{w}_s, dw_t)$, where F is the spectral measure for $Z(\mathbf{s}, t)$. Additionally, if $K(\boldsymbol{\Delta}, \delta)$ is integrable then F is absolutely continuous and has a spectral density, f, with respect to the Lebesgue measure which is the Fourier transform of K, thus allowing $K(\boldsymbol{\Delta}, \delta) = \iint_{\Re^{d+1}} \exp(i\boldsymbol{w}_s^{\mathrm{T}}\boldsymbol{\Delta} + iw_t\delta)\; f(\boldsymbol{w}_s, w_t)\; d\boldsymbol{w}_s\; dw_t$, where f is a continuous, non-negative, integrable function. If, $f(\boldsymbol{w}_s, w_t) = f_s(\boldsymbol{w}_s)\; f_t(w_t)$, a separable spatiotemporal covariance arises (see Section S12 for examples). The next proposition provides the necessary conditions from the perspective of spectral theory that ensure the existence of $\mathcal{L}^* Z(\mathbf{s}, t)$.

Proposition 5. *The process $\mathcal{L}^* Z(\mathbf{s}, t)$ is valid if the associated spatiotemporal spectral density possesses fourth moments in space and time.*

Proof. We consider the non-separable case. The proof for the separable case uses $f(\boldsymbol{w}_s, w_t) = f_s(\boldsymbol{w}_s) f_t(w_t)$. Note that f is symmetric around the origin,

$$K(\boldsymbol{\Delta}, \delta) = \iint_{\Re^{d+1}} e^{i\left(\boldsymbol{w}_s^{\mathrm{T}}\boldsymbol{\Delta} + w_t\delta\right)} f(\boldsymbol{w}_s, w_t)\; d\boldsymbol{w}_s\; dw_t = \iint_{\Re^{d+1}} \cos(\boldsymbol{w}_s^{\mathrm{T}}\boldsymbol{\Delta} + w_t\delta) f(\boldsymbol{w}_s, w_t)\; d\boldsymbol{w}_s\; dw_t,$$

Since f is integrable, differentiating with respect to $\boldsymbol{\Delta}$ and δ we obtain

$$\boldsymbol{\partial}_s K(\boldsymbol{\Delta}, \delta) = -\iint_{\Re^{d+1}} \boldsymbol{w}_s \sin(\boldsymbol{w}_s^{\mathrm{T}}\boldsymbol{\Delta} + w_t\delta) f(\boldsymbol{w}_s, w_t)\; d\boldsymbol{w}_s\; dw_t,$$

$$\partial_t K(\boldsymbol{\Delta}, \delta) = -\iint_{\Re^{d+1}} w_t \sin(\boldsymbol{w}_s^{\mathrm{T}}\boldsymbol{\Delta} + w_t\delta) f(\boldsymbol{w}_s, w_t)\; d\boldsymbol{w}_s\; dw_t,$$

Since, $|\boldsymbol{w}_s \sin(\boldsymbol{w}_s^{\mathrm{T}}\boldsymbol{\Delta} + w_t\delta) f(\boldsymbol{w}_s, w_t)| \le |\boldsymbol{w}_s f(\boldsymbol{w}_s, w_t)|$, $|w_t \sin(\boldsymbol{w}_s^{\mathrm{T}}\boldsymbol{\Delta} + w_t\delta) f(\boldsymbol{w}_s, w_t)| \le |w_t f(\boldsymbol{w}_s, w_t)|$,

$\iint\limits_{\Re^{d+1}} |\boldsymbol{w}_s| f(\boldsymbol{w}_s, w_t)\, d\boldsymbol{w}_s\, dw_t < \infty$ and $\iint\limits_{\Re^{d+1}} |w_t| f(\boldsymbol{w}_s, w_t)\, d\boldsymbol{w}_s\, dw_t < \infty$ differentiation under the integral sign is valid and we have

$$\partial_t^j \boldsymbol{\partial}_s^{r-j} K(\boldsymbol{\Delta}, \delta) = \iint_{\Re^{d+1}} \boldsymbol{w}_s^{\otimes(r-j)} w_t^j \cos\left(\frac{r\pi}{2} + \boldsymbol{w}_s^{\mathrm{T}} \boldsymbol{\Delta} + w_t \delta\right) f(\boldsymbol{w}_s, w_t)\, d\boldsymbol{w}_s\, dw_t,$$

where $r > 0$ and for $\boldsymbol{w}^{\otimes^{rs}}$ the integration is defined element-wise. Under isotropy, noting that the differentiation is with respect to $||\boldsymbol{\Delta}||$ and $|\delta|$ (see, for e.g, Adler 1981, Theorem 2.5.2, p. 35), the above result simplifies to

$$\partial_t^j \boldsymbol{\partial}_s^{r-j} \widetilde{K}(||\boldsymbol{\Delta}||, |\delta|) = \iint\limits_{\Re^2} \boldsymbol{w}_s^{r-j} w_t^j \cos\left(\frac{r\pi}{2} + \boldsymbol{w}_s ||\boldsymbol{\Delta}|| + w_t |\delta|\right) \tilde{f}(\boldsymbol{w}_s, w_t)\, d\boldsymbol{w}_s\, dw_t.$$

We focus on $\partial_t^4 \boldsymbol{\partial}_s^4 K(\boldsymbol{\Delta}, \delta)$ and pass to the limits. We note that the limits exist and the Dominated Convergence Theorem allows for the limit to be interchanged with the integral sign. We make the following observations,

$$\lim_{\substack{||\boldsymbol{\Delta}|| \to 0 \\ |\delta| \to 0}} \left(\partial_t^4 \boldsymbol{\partial}_s^2 \widetilde{K} - \frac{\partial_t^4 \boldsymbol{\partial}_s \widetilde{K}}{||\boldsymbol{\Delta}||}\right)$$
$$= -\lim_{||\boldsymbol{\Delta}|| \to 0} \iint\limits_{\Re^2} \left(\cos(\boldsymbol{w}_s ||\boldsymbol{\Delta}||) - \frac{\sin(\boldsymbol{w}_s ||\boldsymbol{\Delta}||)}{\boldsymbol{w}_s ||\boldsymbol{\Delta}||}\right) \boldsymbol{w}_s^2 w_t^4 \tilde{f}(\boldsymbol{w}_s, w_t)\, d\boldsymbol{w}_s dw_t = 0,$$
$$\lim_{\substack{||\boldsymbol{\Delta}|| \to 0 \\ |\delta| \to 0}} \partial_t^4 \boldsymbol{\partial}_s^3 \widetilde{K} = \lim_{||\boldsymbol{\Delta}|| \to 0} \iint\limits_{\Re^2} \boldsymbol{w}_s^3 w_t^4 \sin(\boldsymbol{w}_s ||\boldsymbol{\Delta}||) \tilde{f}(\boldsymbol{w}_s, w_t)\, d\boldsymbol{w}_s dw_t = 0,$$
$$\lim_{\substack{||\boldsymbol{\Delta}|| \to 0 \\ |\delta| \to 0}} \partial_t^4 \boldsymbol{\partial}_s^4 \widetilde{K} = \lim_{||\boldsymbol{\Delta}|| \to 0} \iint\limits_{\Re^2} \boldsymbol{w}_s^4 w_t^4 \cos(\boldsymbol{w}_s ||\boldsymbol{\Delta}||) \tilde{f}(\boldsymbol{w}_s, w_t)\, d\boldsymbol{w}_s dw_t$$
$$= \iint\limits_{\Re^2} \boldsymbol{w}_s^4 w_t^4 \tilde{f}(\boldsymbol{w}_s, w_t)\, d\boldsymbol{w}_s dw_t < \infty.$$

Referring to the expressions in Proposition 2, we observe that the factors corresponding to $\left(d_{|\delta|}^4 d_{||\boldsymbol{\Delta}||}^2 \widetilde{K} - \frac{d_{|\delta|}^4 d_{||\boldsymbol{\Delta}||} \widetilde{K}}{||\boldsymbol{\Delta}||}\right)$ and $d_{|\delta|}^4 d_{||\boldsymbol{\Delta}||}^3 \widetilde{K}$ are $\left(-3\frac{\mathbf{P}_{4,1}}{||\boldsymbol{\Delta}||^4} + \frac{\mathbf{P}_{4,2}}{||\boldsymbol{\Delta}||^2} + 15\frac{\mathbf{P}_{4,3}}{||\boldsymbol{\Delta}||^6} - 3\frac{\mathbf{P}_{4,4}}{||\boldsymbol{\Delta}||^4}\right)$ and $\left(\frac{\mathbf{P}_{4,1}}{||\boldsymbol{\Delta}||^3} - 6\frac{\mathbf{P}_{4,3}}{||\boldsymbol{\Delta}||^5} + \frac{\mathbf{P}_{4,4}}{||\boldsymbol{\Delta}||^3}\right)$ respectively, remain bounded as $||\boldsymbol{\Delta}|| \to 0$. For the spatiotemporal derivative process to be valid, the spectral density, $\tilde{f}(\boldsymbol{w}_s, w_t)$, must possess fourth moments in space and time. □

Working with non-separable spatiotemporal kernels, verifying the necessary condition

in Proposition 5 requires the existence of closed-form Fourier inversion for the spectral density associated with the kernel. In a purely spatial setting and/or a separable space-time scenario, where Fourier transform pairs exist, verifying this condition is straightforward (see, e.g., Wang et al. 2018, Halder et al. 2024). If we assume separability, then covariance kernels satisfying this condition are readily available. However, non-separable spatiotemporal covariance functions that have closed-form spectral distributions are not easily available (see, e.g., Gneiting 2002, Fuentes et al. 2008). The Gneiting class (see, e.g., Gneiting 2002) of stationary non-separable spatiotemporal covariance functions allows for a simpler verification of the required condition. They are expressible as $K(\boldsymbol{\Delta},\delta) = \widetilde{K}(\|\boldsymbol{\Delta}\|,|\delta|) = \frac{\sigma^2}{\psi(|\delta|^2)^{d/2}}\varphi\left(\frac{\|\boldsymbol{\Delta}\|^2}{\psi(|\delta|^2)}\right)$, where $\varphi(x)$ and $\psi(x)$ are complete monotone, and positive with complete monotone derivative on $[0,\infty)$ respectively. Particularly, we are interested in the Matérn kernel, $\varphi(x) = \left(2^{\nu-1}\boldsymbol{\Gamma}(\nu)\right)^{-1}(\phi_s x^{1/2})^{\nu}K_{\nu}(\phi_s x^{1/2})$, $\phi_s > 0, \nu > 0$, where $K_{\nu}(\cdot)$ is the modified Bessel function of second kind, and $\psi(x) = (\phi_t^2 x^{\alpha}+1)^{\beta}$, $a > 0$, $0 < \alpha \leq 1$, $0 \leq \beta \leq 1$. We fix $\alpha = \beta = 1$, producing the covariance kernel, $\widetilde{K}(\|\boldsymbol{\Delta}\|,|\delta|;\boldsymbol{\theta}) = \frac{\sigma^2}{2^{\nu-1}\boldsymbol{\Gamma}(\nu)A_t^{d/2}}\left(\frac{\phi_s\|\boldsymbol{\Delta}\|}{A_t^{1/2}}\right)^{\nu}K_{\nu}\left(\frac{\phi_s\|\boldsymbol{\Delta}\|}{A_t^{1/2}}\right)$, $A_t = \phi_t^2|\delta|^2+1$. Observe that for $\delta = 0$, $K(\|\boldsymbol{\Delta}\|,0;\boldsymbol{\theta})$ is the spatial Matérn and, for a fixed δ, $\widetilde{K}(\|\boldsymbol{\Delta}\|,|\delta|;\boldsymbol{\theta})$ is a re-scaled spatial Matérn, with a spectral density, $\tilde{f}(\boldsymbol{w}_s) = C_1(\boldsymbol{\theta},|\delta|)\left(C_2(\boldsymbol{\theta},|\delta|)+\|\boldsymbol{w}_s\|^2\right)^{-\nu-d/2}$ that belongs to the t-family. The fourth spectral moment exists if $\nu > 2$. If $\|\boldsymbol{\Delta}\| \to 0$, observe that $\varphi(0) = 1$, hence, $\widetilde{K}(0,|\delta|;\boldsymbol{\theta}) = \frac{\sigma^2}{(\phi_t^2|\delta|^2+1)^{d/2}}$. This has a spectral density in the Laplace family, $\tilde{f}(w_t) = \frac{\sigma^2}{\phi_t}\sqrt{\frac{\pi}{2}}\exp\left(-\frac{|w_t|}{\phi_t^2}\right)$, for which the fourth moment always exists. In case $\|\boldsymbol{\Delta}\| \neq 0$ fixed, and $|\delta|$ close to 0, $\varphi\left(\frac{\|\boldsymbol{\Delta}\|^2}{\psi(|\delta|^2)}\right)$ does not interfere with smoothness of $\psi(|\delta|^2)^{d/2}$, for our choices of $\varphi(\cdot)$ and $\psi(\cdot)$, allowing for $K(\|\boldsymbol{\Delta}\|,|\delta|)$ to inherit smoothness properties of $K(0,|\delta|)$ (see for e.g. Stein 2005, pp. 311–312). We also consider the case, $\varphi(x)$, $\phi_s > 0$ and $\nu \to \infty$ resulting in the squared exponential kernel. In such a scenario, if $|\delta| \neq 0$ is kept constant, the spectral distribution, $\tilde{f}(\boldsymbol{w}_s) = \left(\frac{\pi}{\phi_s}\right)^{d/2}\exp\left(-\pi^2\frac{\psi(|\delta|^2)}{\phi_s}\|\boldsymbol{w}_s\|^2\right)$, belongs to the Gaussian family having a finite fourth moment (see, e.g., Rasmussen & Williams 2005, Section 4.2.1, pp. 82–83). Evidently, in the Gneiting class (with $\varphi(x)$ chosen to be Matérn), spatial smoothness of

process realizations is governed by the fractal parameter, $\nu > 0$, of the Matérn kernel. We consider only half-integer values of $\nu = \nu_0 + \frac{1}{2}$, $\nu_0 = 0, 1, 2$ and $\nu \to \infty$, for which we have closed-form expressions of $K_\nu(\cdot)$. Our choice of $\alpha = 1$ allows us to study second order temporal derivatives. However, $\alpha \in (0, 1]$ can be used to control the temporal smoothness of process realizations (see, e.g., Gneiting 2002). The next discussion focuses on surfaces in $\Re^3$ that have *simple* parameterizations.

S7 Parametric Surfaces

We focus on parametric surfaces $\mathscr{C} = \left\{(s_x(\omega, v), s_y(\omega, v), t(v)) : (\omega, v) \in \mathcal{D}_\omega \times \mathcal{D}_v\right\}$. The normal to $\mathscr{C}$ at a point $(\boldsymbol{s}(\omega, v), t(v))$, $\boldsymbol{s}(\omega, v) = (s_x(\omega, v), s_y(\omega, v))^{\mathrm{T}}$ is,

$$\begin{aligned}\overline{\boldsymbol{n}}(\boldsymbol{s}(\omega,v),v) &= \left(\frac{\partial s_x(\omega,v)}{\partial\omega}, \frac{\partial s_y(\omega,v)}{\partial\omega}, 0\right)^{\mathrm{T}} \times \left(\frac{\partial s_x(\omega,v)}{\partial v}, \frac{\partial s_y(\omega,v)}{\partial v}, \frac{\partial t(v)}{\partial v}\right)^{\mathrm{T}} \\ &= \begin{vmatrix} i & j & k \\ \frac{\partial s_x(\omega,v)}{\partial\omega} & \frac{\partial s_y(\omega,v)}{\partial\omega} & 0 \\ \frac{\partial s_x(\omega,v)}{\partial v} & \frac{\partial s_y(\omega,v)}{\partial v} & \frac{\partial t(v)}{\partial v}\end{vmatrix} = \frac{\partial s_y(\omega,v)}{\partial\omega}\frac{\partial t(v)}{\partial v}\,\hat{\imath} - \frac{\partial s_x(\omega,v)}{\partial\omega}\frac{\partial t(v)}{\partial v}\,\hat{\jmath} + \left|\frac{\partial(s_x,s_y)}{\partial(\omega,v)}\right|\,\hat{k},\end{aligned} \tag{S4}$$

where $\times$ is the vector product. The equation of the tangent plane at $(\boldsymbol{s}(\omega_0, v_0), v_0)$ is, $\left\{(x, y, z) : \overline{\boldsymbol{n}}(\boldsymbol{s}(\omega_0, v_0), t(v_0))^{\mathrm{T}} \begin{pmatrix} x - s_x(\omega_0,v_0) \\ y - s_y(\omega_0,v_0) \\ z - t(v_0)\end{pmatrix} = 0\right\}$. For convenience, we assume $t(v) = v$. Note that $\overline{\boldsymbol{n}}_s(\omega, v) = \left(\frac{\partial s_y(\omega,v)}{\partial\omega}, -\frac{\partial s_x(\omega,v)}{\partial\omega}\right)^{\mathrm{T}}$ is the normal to the parametric planar curve, $C_v = \{(s_x(\omega, v), s_y(\omega, v)) : \omega \in \mathcal{D}_\omega\}$. This connects parametric curvilinear wombling (see, e.g., Banerjee & Gelfand 2006, Halder et al. 2024) to surface wombling. It is also the reason behind our choice of parameterization for $\mathscr{C}$. The usual parameterization found in the literature $\mathscr{C} = \left\{(s_x(\omega, v), s_y(\omega, v), t(\omega, v)) : (\omega, v) \in \mathcal{D}_\omega \times \mathcal{D}_v\right\}$ is not suitable; the parameterization of the time coordinate cannot depend on the parameterization of spatial coordinates. Hence, $t(\omega, v) = t(v)$. In that case, $\overline{\boldsymbol{n}}(\boldsymbol{s}(\omega, v), v) = \frac{\partial s_y(\omega,v)}{\partial\omega}\frac{\partial t(v)}{\partial v}\,\hat{\imath} - \frac{\partial s_x(\omega,v)}{\partial\omega}\frac{\partial t(v)}{\partial v}\,\hat{\jmath} + \left|\frac{\partial(s_x,s_y)}{\partial(\omega,v)}\right|\,\hat{k}$. Further assuming, $t(v) = v$, produces the desired normal. The surface $\mathscr{C}$ is regular if the bottom two rows of the determinant in eq. (S4) are linearly independent. Note that our parameterization results in regular surfaces with the exception where $\frac{\partial s_x(\omega,v)}{\partial\omega} = \frac{\partial s_y(\omega,v)}{\partial\omega} = 0$ and $\|\boldsymbol{n}_s\| = 0$.

Hence, we define $\mathscr{C}$ as regular if $||\boldsymbol{n}_{\boldsymbol{s}}|| \neq 0$. Parametric curves were defined to be regular in Banerjee & Gelfand (2006) using the same condition. Finally, turning to static wombling, $\left|\frac{\partial(s_x,s_y)}{\partial(\omega,v)}\right|$ can be zero even if the curve is evolving over time—if the vectors $\left(\frac{\partial s_x(\omega,v)}{\partial\omega}, \frac{\partial s_y(\omega,v)}{\partial\omega}\right)^{\mathrm{T}}$, $\left(\frac{\partial s_x(\omega,v)}{\partial v}, \frac{\partial s_y(\omega,v)}{\partial v}\right)^{\mathrm{T}}$ are linearly dependent. We exclude such parameterizations. Eventually, working with parametric triangular planes (see Section 4.2 of the manuscript) this is not a concern.

S8 Curves on Surfaces: Gauss's Divergence Theorem

We provide proofs for the results concerning *spatiotemporal flux* (STF) and the *rate of change in spatiotemporal flux* (RSTF) in Section 3.2 of the manuscript. We assume that $d = 2$.

Proposition 6. *Let the process $Z(\boldsymbol{s},t)$ be thrice differentiable, $\boldsymbol{F}_1(\boldsymbol{s},t) = \begin{pmatrix} F_{11} \\ F_{12} \\ F_{13} \end{pmatrix} = \nabla^{\otimes 1}Z(\boldsymbol{s},t) = \begin{pmatrix} \partial_{\boldsymbol{s}}Z(\boldsymbol{s},t) \\ \partial_t Z(\boldsymbol{s},t) \end{pmatrix}$ and $\boldsymbol{F}_2(\boldsymbol{s},t) = \begin{pmatrix} F_{21} \\ \vdots \\ F_{26} \end{pmatrix} = \nabla^{\otimes 2}Z(\boldsymbol{s},t)$. The STF and RSTF satisfy*

$$\oiint_{\mathscr{C}=\partial W} \boldsymbol{n}(\boldsymbol{s},t)^{\mathrm{T}}\boldsymbol{F}_1(\boldsymbol{s},t)\,\mathrm{d}\mathscr{A} = \iiint_W \mathrm{div}\ \boldsymbol{F}_1\ \mathrm{d}W,$$

$$\oiint_{\mathscr{C}=\partial W} \{\boldsymbol{n}(\boldsymbol{s},t)^{\otimes 2}\}^{\mathrm{T}}\boldsymbol{F}_2(\boldsymbol{s},t)\,\mathrm{d}\mathscr{A} = \iiint_W \left\{\mathrm{div}\ n_{s_x}\begin{pmatrix} F_{21} \\ F_{22} \\ F_{23} \end{pmatrix} + \mathrm{div}\ n_{s_y}\begin{pmatrix} F_{22} \\ F_{24} \\ F_{25} \end{pmatrix} + \mathrm{div}\ n_t\begin{pmatrix} F_{23} \\ F_{25} \\ F_{26} \end{pmatrix}\right\}\mathrm{d}W,$$

where W is a simple region enclosed by curves, C_v which are closed for every $v \in [v_0, v_1]$, $\oiint$ denotes the surface integral using the default counter-clockwise orientation of $\mathscr{C}$ which produces outward pointing normals, $\mathrm{d}W = ds_x\ ds_y\ dt$ is the volume element and $\mathrm{div}\ n_{s_x}\begin{pmatrix} F_{21} \\ F_{22} \\ F_{23} \end{pmatrix} + \mathrm{div}\ n_{s_y}\begin{pmatrix} F_{22} \\ F_{24} \\ F_{25} \end{pmatrix} + \mathrm{div}\ n_t\begin{pmatrix} F_{23} \\ F_{25} \\ F_{26} \end{pmatrix} = (\frac{\partial}{\partial s_x}n_{s_x}F_{21} + \frac{\partial}{\partial s_y}n_{s_x}F_{22} + \frac{\partial}{\partial t}n_{s_x}F_{23}) + (\frac{\partial}{\partial s_x}n_{s_y}F_{22} + \frac{\partial}{\partial s_y}n_{s_y}F_{24} + \frac{\partial}{\partial t}n_{s_y}F_{25}) + (\frac{\partial}{\partial s_x}n_tF_{23} + \frac{\partial}{\partial s_y}n_tF_{25} + \frac{\partial}{\partial t}n_tF_{26})$, consisting of elements from $\nabla^{\otimes 3}Z(\boldsymbol{s},t)$.

Proof. With regard to the first identity for STF, observe that

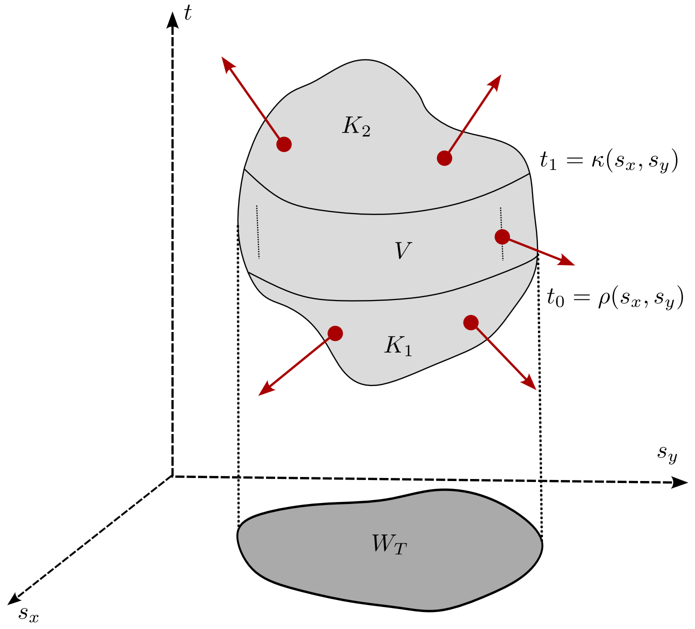

Figure S1: Figure supplementing the proof of Proposition 6. Normals to the surface are in red.

$$\oiint_{\mathscr{C}=\partial W} \boldsymbol{n}(\boldsymbol{s},t)^{\mathrm{T}}\boldsymbol{F}_1(\boldsymbol{s},t)\,\mathrm{d}\mathscr{A} = \iint_{\mathscr{C}} \frac{\overline{\boldsymbol{n}}(\boldsymbol{s}(\omega,v),v)^{\mathrm{T}}}{\|\overline{\boldsymbol{n}}(\boldsymbol{s}(\omega,v),v)\|}\boldsymbol{F}_1(\boldsymbol{s}(\omega,v),v)\,\|\overline{\boldsymbol{n}}(\boldsymbol{s}(\omega,v),v)\|\,d\omega\,dv$$
$$= \iint_{\mathscr{C}} F_{11}\frac{\partial s_y(\omega,v)}{\partial\omega} - F_{12}\frac{\partial s_x(\omega,v)}{\partial\omega} + F_{13}\left|\frac{\partial(s_x,s_y)}{\partial(\omega,v)}\right|\,d\omega\,dv$$
$$= \iint_{\mathscr{C}} F_{11}\frac{\partial s_y(\omega,v)}{\partial\omega}\,d\omega\,dv + \iint_{\mathscr{C}} F_{12}\left(-\frac{\partial s_x(\omega,v)}{\partial\omega}\right)\,d\omega\,dv + \iint_{\mathscr{C}} F_{13}\left|\frac{\partial(s_x,s_y)}{\partial(\omega,v)}\right|\,d\omega\,dv.$$

Focusing on the third component above, we prove the identity

$$\iint_{\mathscr{C}} F_{13}\left|\frac{\partial(s_x,s_y)}{\partial(\omega,v)}\right|\,d\omega\,dv = \iiint_{W}\frac{\partial F_{13}}{\partial t}\,ds_x\,ds_y\,dt = \iiint_{W}\frac{\partial F_{13}}{\partial t}\,\mathrm{d}W.$$

Since W is a simple region (s_x-s_y simple), there exists a simple region, W_T in $\Re^2$ and continuous functions $\rho : \Re^2 \to \Re$ and $\kappa : \Re^2 \to \Re$ such that $\rho(s_x,s_y) < \tau(s_x,s_y)$ for all $(s_x,s_y) \in W_T \setminus \partial W_T$ as seen in fig. S1. The boundary $\partial W = \mathscr{C}$ for W consists of a top, the graph of $\kappa(s_x,s_y)$, denoted by K_2, a bottom, the graph of $\rho(s_x,s_y)$, denoted by K_1 and possibly a vertical collar V and $\iint_{\mathscr{C}} F_{13}\left|\frac{\partial(s_x,s_y)}{\partial(\omega,v)}\right|\,d\omega\,dv = \iint_{K_1} F_{13}\left|\frac{\partial(s_x,s_y)}{\partial(\omega,v)}\right|\,d\omega\,dv + \iint_{V} F_{13}\left|\frac{\partial(s_x,s_y)}{\partial(\omega,v)}\right|\,d\omega\,dv + \iint_{K_2} F_{13}\left|\frac{\partial(s_x,s_y)}{\partial(\omega,v)}\right|\,d\omega\,dv$. Since K_j, for $j = 1,2$ is a surface, there are simple regions $S_1,\ldots,S_k$ whose interiors, denoted by $\mathrm{Int}(S_i)$, $i = 1,\ldots,k$, are disjoint, such that for injective and smooth functions $s_x : \Re^2 \to \Re$ and $s_y : \Re^2 \to \Re$ on $U = \bigcup_{i=1}^{k}\mathrm{Int}(S_i)$,

$K_j = \left\{(s_x(\omega, v)), s_y(\omega, v), v) : \omega, v \in S = \bigcup_{i=1}^{k} S_i\right\}$, for $j = 1, 2$. Since K_1 is the bottom of the surface, the third coordinate of $\overline{\boldsymbol{n}}(\boldsymbol{s}(\omega, v), v)$ is negative. Using change of variables, we get

$$\iint_{K_1} F_{13} \left|\frac{\partial(s_x, s_y)}{\partial(\omega, v)}\right| \, d\omega \, dv = -\iint_{W_T} F_{13}(s_x, s_y, \rho(s_x, s_y)) ds_x \, ds_y.$$

Since F_{13} is tangential to the vertical collar V, $\iint_V F_{13} \left|\frac{\partial(s_x, s_y)}{\partial(\omega, v)}\right| \, d\omega \, dv = 0$. Note that K_2 is the top of the surface and hence the third coordinate of $\overline{\boldsymbol{n}}$ would be positive. Using change of variables, $\iint_{K_2} F_{13} \left|\frac{\partial(s_x, s_y)}{\partial(\omega, v)}\right| \, d\omega \, dv = \iint_{W_T} F_{13}(s_x, s_y, \kappa(s_x, s_y)) \, ds_x \, ds_y$. It follows that

$$\begin{aligned}\iint_{\mathscr{C}} F_{13} \left|\frac{\partial(s_x, s_y)}{\partial(\omega, v)}\right| \, d\omega \, dv &= \iint_{W_T} F_{13}(s_x, s_y, \kappa(s_x, s_y)) \, ds_x \, ds_y - \iint_{W_T} F_{13}(s_x, s_y, \rho(s_x, s_y)) \, ds_x \, ds_y, \\ &= \iint_{W_T} ds_x \, ds_y \int_{\rho(s_x, s_y)}^{\kappa(s_x, s_y)} \frac{\partial F_{13}(s_x, s_y, t)}{\partial t} \, dt = \iiint_W \frac{\partial F_{13}}{\partial t} \mathrm{d}W.\end{aligned}$$

Similar results follow for F_{11} and F_{12} observing that $\left|\frac{\partial(s_y, t)}{\partial(\omega, v)}\right| = \frac{\partial s_y(\omega, v)}{\partial \omega}$, $\left|\frac{\partial(s_x, t)}{\partial(\omega, v)}\right| = -\frac{\partial s_x(\omega, v)}{\partial \omega}$ provided that $\frac{\partial F_{11}}{\partial s_x}$, $\frac{\partial F_{12}}{\partial s_y}$ and $\frac{\partial F_{13}}{\partial t}$ exist, i.e. $Z(\boldsymbol{s}, t)$ is twice differentiable.

The second identity concerning the RSTF is proved similarly. Observe that $F_{21} = \frac{\partial^2}{\partial s_x^2} Z(\boldsymbol{s}, t)$, $F_{22} = \frac{\partial^2}{\partial s_x \partial s_y} Z(\boldsymbol{s}, t)$, $F_{23} = \frac{\partial^2}{\partial s_x \partial t} Z(\boldsymbol{s}, t)$, $F_{24} = \frac{\partial^2}{\partial s_y^2} Z(\boldsymbol{s}, t)$, $F_{25} = \frac{\partial^2}{\partial s_y \partial t} Z(\boldsymbol{s}, t)$ and $F_{26} = \frac{\partial^2}{\partial t^2} Z(\boldsymbol{s}, t)$. Hence, $\boldsymbol{\nabla}^{\otimes 2} Z(\boldsymbol{s}, t) = (F_{21}, F_{22}, F_{23}, F_{22}, F_{24}, F_{25}, F_{23}, F_{25}, F_{26})^{\mathrm{T}}$ is the 9×1 vector of pure and mixed spatiotemporal derivatives. Let $\overline{\boldsymbol{n}}(\boldsymbol{s}(\omega, v), v) = (\overline{n}_{s_x}(\omega, v), \overline{n}_{s_y}(\omega, v), \overline{n}_t(\omega, v))^{\mathrm{T}} = \left(\frac{\partial s_y(\omega, v)}{\partial \omega}, -\frac{\partial s_x(\omega, v)}{\partial \omega}, \left|\frac{\partial(s_x, s_y)}{\partial(\omega, v)}\right|\right)^{\mathrm{T}}$ and similarly $n_{s_x}(\omega, v) = \frac{\overline{n}_{s_x}(\omega, v)}{\|\overline{\boldsymbol{n}}(\boldsymbol{s}(\omega, v), v)\|}$ etc. The left-hand side of the identity can be expressed as

$$\oiint_{\mathscr{C} = \partial W} \left(n_{s_x}^2 F_{21} + n_{s_y}^2 F_{24} + n_t^2 F_{26}\right) + 2\left(n_{s_x} n_{s_y} F_{22} + n_{s_x} n_t F_{23} + n_{s_y} n_t F_{25}\right) \, \mathrm{d}\mathscr{A}.$$

We show $\oiint_{\mathscr{C} = \partial W} n_t^2 F_{26} \, \mathrm{d}\mathscr{A} = \iiint_W \frac{\partial \, n_t F_{26}}{\partial t} \mathrm{d}W$ and $2 \oiint_{\mathscr{C} = \partial W} n_{s_y} n_t F_{25} \, \mathrm{d}\mathscr{A} = \iiint_W \frac{\partial \, n_t F_{25}}{\partial s_y} \mathrm{d}W + \iiint_W \frac{\partial \, n_{s_y} F_{25}}{\partial t} \mathrm{d}W$. Similar identities follow for the other terms. For the first identity,

$$\iint\limits_{\mathscr{C}} n_t F_{26} \left|\frac{\partial(s_x, s_y)}{\partial(\omega, v)}\right| d\omega\, dv = \iint\limits_{W_T} n_t(s_x, s_y, \kappa(s_x, s_y))\, F_{26}(s_x, s_y, \kappa(s_x, s_y))\, ds_x\, ds_y -$$

$$\iint\limits_{W_T} n_t(s_x, s_y, \rho(s_x, s_y))\, F_{26}(s_x, s_y, \rho(s_x, s_y))\, ds_x\, ds_y,$$

$$= \iint\limits_{W_T} ds_x\, ds_y \int\limits_{\rho(s_x,s_y)}^{\kappa(s_x,s_y)} \frac{\partial n_t(s_x, s_y, t) F_{26}(s_x, s_y, t)}{\partial t} dt = \iiint\limits_{W} \frac{\partial\, n_t F_{26}}{\partial t} \mathrm{d}W.$$

The second equality is proved by observing that W is also s_x-t simple and writing $2 \oiint\limits_{\mathscr{C}} n_{s_y} n_t F_{25}\ \mathrm{d}\mathscr{A} = \oiint\limits_{\mathscr{C}} n_{s_y} F_{25} \left|\frac{\partial(s_x,s_y)}{\partial(\omega,v)}\right|\ d\omega\ dv + \oiint\limits_{\mathscr{C}} n_t F_{25} \left|\frac{\partial(s_x,t)}{\partial(\omega,v)}\right|\ d\omega\ dv$ followed by similar arguments as pursued for the proof for the previous identity. Combining we have

$$\oiint\limits_{\mathscr{C}=\partial W} \{\boldsymbol{n}(\boldsymbol{s}, t)^{\otimes 2}\}^{\mathrm{T}} F_2(\boldsymbol{s}, t)\ \mathrm{d}\mathscr{A} = \iiint\limits_{W} \left\{\left(\frac{\partial}{\partial s_x} n_{s_x} F_{21} + \frac{\partial}{\partial s_y} n_{s_x} F_{22} + \frac{\partial}{\partial t} n_{s_x} F_{23}\right) + \left(\frac{\partial}{\partial s_y} n_{s_y} F_{24} + \frac{\partial}{\partial s_x} n_{s_y} F_{22} + \frac{\partial}{\partial t} n_{s_y} F_{25}\right) + \left(\frac{\partial}{\partial t} n_t F_{26} + \frac{\partial}{\partial s_x} n_t F_{23} + \frac{\partial}{\partial s_y} n_t F_{25}\right)\right\}\ \mathrm{d}W.$$

The integrand on the right-hand side is the expanded expression for div $n_{s_x} \begin{pmatrix} F_{21} \\ F_{22} \\ F_{23} \end{pmatrix} +$ div $n_{s_y} \begin{pmatrix} F_{22} \\ F_{24} \\ F_{25} \end{pmatrix} +$ div $n_t \begin{pmatrix} F_{23} \\ F_{25} \\ F_{26} \end{pmatrix}$. It is comprised of elements from $\nabla^{\otimes 3} Z(\boldsymbol{s}, t)$ which requires $Z(\boldsymbol{s}, t)$ to be thrice differentiable. The arguments above are adapted from the proof of Gauss's Divergence Theorem (see, e.g., Spivak 1979). □

S9 Asymptotic Properties of $\boldsymbol{\Gamma}(\widetilde{\mathscr{C}})$

We investigate the asymptotic properties of the wombling measure, $\boldsymbol{\Gamma}(\widetilde{\mathscr{C}})$ computed on the triangulation, $\widetilde{\mathscr{C}}$ of a regular surface, $\mathscr{C}$ using a partition, $\mathscr{P}$ as the norm of the partition, $|\mathscr{P}|_A \to 0$. This supplements the discussion in Section 4.2 of the manuscript.

Theorem 2. *Let $\mathscr{C}$ be a regular (wombling) surface that can be triangulated using a partition $\mathscr{P}$ (with norm $|\mathscr{P}|_A$) and let $\widetilde{\mathscr{C}}$ be the triangulated approximation. Then $\boldsymbol{\Gamma}_g(\widetilde{\mathscr{C}}) \overset{a.s.}{\to} \boldsymbol{\Gamma}_g(\mathscr{C})$ as $|\mathscr{P}|_A \to 0$, where $\boldsymbol{\Gamma}_g(\widetilde{\mathscr{C}})$ is the wombling measure for $\widetilde{\mathscr{C}}$ and $\boldsymbol{\Gamma}_g(\mathscr{C}) = \iint\limits_{\mathscr{C}} g\left(\boldsymbol{\mathcal{L}}^*_{n_t, \boldsymbol{n}_s} Z(\boldsymbol{s}, t)\right)\ \mathrm{d}\mathscr{A}$ with $g(\cdot)$ being any uniformly continuous (u.c.) function.*

Proof. Over the rectangle $\mathcal{D}_\omega \times \mathcal{D}_v = [\omega_0, \omega_1] \times [v_0, v_1]$, the wombling measure $\boldsymbol{\Gamma}_g(\mathscr{C}) = \int_{v_0}^{v_1}\int_{\omega_0}^{\omega_1} g\left(\boldsymbol{\mathcal{L}}^*_{n_t,\boldsymbol{n}_s} Z(\boldsymbol{s}(\omega,v),v)\right) \ \|\overline{\boldsymbol{n}}(\boldsymbol{s}(\omega,v),v)\| \ d\omega \ dv$ and we denote $g\left(\boldsymbol{\mathcal{L}}^*_{n_t,\boldsymbol{n}_s} Z(\boldsymbol{s}(\omega,v),v)\right) = g(\omega,v)$ and $\overline{\boldsymbol{n}}(\boldsymbol{s}(\omega,v),v) = \overline{\boldsymbol{n}}(\omega,v)$. Given the partition $\mathscr{P}$, $\omega_0 = \omega'_1 < \ldots < \omega'_{n_\omega} < \omega_1$ and $v_0 = v'_1 < \ldots < v'_{n_v} = v_1$ used to triangulate the surface, using the definition of a surface integral—for any $\epsilon > 0$ there exists a $\delta_0 > 0$ such that for any $|\mathscr{P}|_A < \delta_0$

$$\left|\int_{v_0}^{v_1}\int_{\omega_0}^{\omega_1} g(\omega,v) \ \|\overline{\boldsymbol{n}}(\omega,v)\| \ d\omega \ dv - \sum_{i=1}^{n_\omega-1}\sum_{j=1}^{n_v-1}\sum_{k=1}^{2}(v'_{j+1}-v'_j)(\omega'_{i+1}-\omega'_i) g\left(\omega^*_i, v^*_j\right) \|\overline{\boldsymbol{n}}_{ijk}(\omega^*_i, v^*_j)\|\right| < \frac{\epsilon}{2},$$

where $\sum_{i=1}^{n_\omega-1}\sum_{j=1}^{n_v-1}\sum_{k=1}^{2}(v'_{j+1}-v'_j)(\omega'_{i+1}-\omega'_i) \ g\left(\omega^*_i, v^*_j\right) \ \|\overline{\boldsymbol{n}}_{ijk}(\omega^*_i, v^*_j)\|$ is the *Riemann sum approximation*, $\begin{pmatrix}\omega^*_i \\ v^*_j\end{pmatrix} = \begin{pmatrix}\omega'_i \\ v'_j\end{pmatrix} + \frac{1}{2}\begin{pmatrix}\omega'_i+\omega'_{i+1} \\ v'_j+v'_{j+1}\end{pmatrix}$ are the centroids of the rectangles which partition $\bigcup_{k=1}^{2} T_{ijk}$ and $\overline{\boldsymbol{n}}_{ijk}$ is the normal to the triangle T_{ijk}, for $k = 1, 2$.

Since, $g : \Re^2 \to \Re$ is a u.c. function over $\mathcal{D}_\omega \times \mathcal{D}_v$, for $\epsilon > 0$, there exists $\delta_1 > 0$ such that for any $x, y \in \mathcal{D}_\omega \times \mathcal{D}_v$ with $\|x - y\| < \delta_1$, $x = \begin{pmatrix}x_\omega \\ x_v\end{pmatrix}$, $y = \begin{pmatrix}y_\omega \\ y_v\end{pmatrix}$ we have

$$\left|g(x_\omega, x_v) \|\overline{\boldsymbol{n}}_{ijk}(x_\omega, x_v)\| - g(y_\omega, y_v) \|\overline{\boldsymbol{n}}_{ijk}(y_\omega, y_v)\|\right| < \frac{\epsilon}{2(\omega_1-\omega_0)(v_1-v_0)}.$$

Setting $\delta = \min\{\delta_0, \delta_1\}$, $|\mathscr{P}|_A < \delta$ and using the Mean Value Theorem we obtain the following inequalities,

$$\Bigg|\sum_{i=1}^{n_\omega-1}\sum_{j=1}^{n_v-1}\sum_{k=1}^{2}\iint_{T_{ijk}} g(\omega,v) \ \|\overline{\boldsymbol{n}}_{ijk}(\omega,v)\| \ d\omega \ dv -$$
$$\sum_{i=1}^{n_\omega-1}\sum_{j=1}^{n_v-1}\sum_{k=1}^{2}(v'_{j+1}-v'_j) \ (\omega'_{i+1}-\omega'_i) \ g\left(\omega^*_i, v^*_j\right) \ \|\overline{\boldsymbol{n}}_{ijk}(\omega^*_i, v^*_j)\|\Bigg| \leq$$
$$\Bigg|\sum_{i=1}^{n_\omega-1}\sum_{j=1}^{n_v-1}\sum_{k=1}^{2}(v'_{j+1}-v'_j)(\omega'_{i+1}-\omega'_i) \sup_{\mathcal{D}_\omega\times\mathcal{D}_v} g(\omega,v) \|\overline{\boldsymbol{n}}_{ijk}(\omega,v)\| -$$
$$\sum_{i=1}^{n_\omega-1}\sum_{j=1}^{n_v-1}\sum_{k=1}^{2}(v'_{j+1}-v'_j)(\omega'_{i+1}-\omega'_i) g\left(\omega^*_i, v^*_j\right) \|\overline{\boldsymbol{n}}_{ijk}(\omega^*_i, v^*_j)\|\Bigg| \leq$$
$$\sum_{i=1}^{n_\omega-1}\sum_{j=1}^{n_v-1}\sum_{k=1}^{2}(v'_{j+1}-v'_j)(\omega'_{i+1}-\omega'_i) \sup_{\mathcal{D}_\omega\times\mathcal{D}_v}\left|g(\omega,v) \|\overline{\boldsymbol{n}}_{ijk}(\omega,v)\| \ - g\left(\omega^*_i, v^*_j\right) \|\overline{\boldsymbol{n}}_{ijk}(\omega^*_i, v^*_j)\|\right| \leq \frac{\epsilon}{2}.$$

Combining this with the first inequality in this proof—for every $\epsilon > 0$, there exists a $\delta > 0$

such that for any $|\mathscr{P}|_A < \delta$ we have

$$\left| \int_{v_0}^{v_1} \int_{\omega_0}^{\omega_1} g(\omega, v)\ \|\overline{\boldsymbol{n}}(\omega, v)\|\ d\omega\ dv - \sum_{i=1}^{n_\omega - 1} \sum_{j=1}^{n_v - 1} \sum_{k=1}^{2} \iint_{T_{ijk}} g(\omega, v)\ \|\overline{\boldsymbol{n}}_{ijk}(\omega, v)\|\ d\omega\ dv \right| < \epsilon. \tag{S5}$$

Our choices for g are all linear, for example, directional derivatives and curvatures which are by definition u.c. Next we show the almost sure convergence of $\boldsymbol{\Gamma}_g(\widetilde{\mathscr{C}})$.

Let $\boldsymbol{\Gamma}_{g,n_\omega,n_v}(\widetilde{\mathscr{C}_Z}) = \sum_{i=1}^{n_\omega-1} \sum_{j=1}^{n_v-1} \sum_{k=1}^{2} \iint_{T_{ijk}} g\left(\boldsymbol{\mathcal{L}}^*_{n_t,\boldsymbol{n}_s} Z(\boldsymbol{s}(\omega, v), v)\right)\ \|\overline{\boldsymbol{n}}(\boldsymbol{s}(\omega, v), v)\|\ d\omega\ dv$ be the wombling measure approximating $\boldsymbol{\Gamma}_g(\mathscr{C}_Z)$ using a triangulated approximation, $\widetilde{\mathscr{C}_Z}$, of surface $\mathscr{C}_Z$, where the suffix Z underscores the dependence on the parent process $Z(\boldsymbol{s}, t)$. Consider sequences $n_\omega, n_v \to \infty$ such that $|\mathscr{P}|_A = \max_{\substack{1\le i\le n_\omega-1 \\ 1\le j\le n_v-1}} (\omega'_{i+1} - \omega'_i)(v'_{j+1} - v'_j) \to 0$. Using eq. (S5) we obtain $\boldsymbol{\Gamma}_{g,n_\omega,n_v}(\widetilde{\mathscr{C}_Z}) \xrightarrow{a.s.} \boldsymbol{\Gamma}_g(\mathscr{C}_Z)$. Therefore, $\boldsymbol{\Gamma}_{g,n_\omega,n_v}(\widetilde{\mathscr{C}_Z})$ is a strongly consistent estimator for $\boldsymbol{\Gamma}_g(\mathscr{C}_Z)$. □

S10 Computational Simplifications

The choice of parameterization, be it simple parametric planes in the spatiotemporal context or, rectilinear segments in the spatial context, allows us to avoid high dimensional quadrature when computing the variance of wombling measures. Simplifications arise in the purely spatial and spatiotemporal case. Our choice of kernels provides further simplifications. They are the focus of the ensuing discussion. We start with the spatial scenario before moving to the spatiotemporal scene. Within a spatial context (see, e.g., Banerjee & Gelfand 2006, Halder et al. 2024, Section 4 and Section 3 respectively), depending on the smoothness of the process, we are interested in performing posterior inference either for wombling measures based on the spatial gradient or, curvature wombling, which seeks posterior inference for wombling measures based jointly on the spatial gradient and curvature processes. Detailing the construction of the cross-covariance matrix associated with such inferential pursuits in the next paragraph, we observe that in the spatial case,

no quadrature is required. We consider $d = 2$ for ease of presentation. We denote the standard Gaussian cumulative distribution function (cdf) using $\Phi(\cdot)$ and the cdf of a gamma distribution with shape parameter a and scale parameter, b as, $G\left(a, \frac{x}{b}\right)$, where $G(\cdot,\cdot)$ is the regularized gamma function.

S10.1 Spatial

Consider the purely spatial case found in the discussion after eq. (12), p. 1493, Banerjee & Gelfand (2006) and also eq. (10) in Halder et al. (2024). Using expressions found in the latter, observe that the curve is parameterized via rectilinear segments. The following lemma details the simplification in the cross-covariance followed by a proposition which uses Lemma 2 to derive further simplifications specific to kernels in Halder et al. (2024).

Lemma 2. *On each rectilinear segment, $C_{t^*} = \{\boldsymbol{s}_0 + t\boldsymbol{u} : t \in [0, t^*]\}$, where $\boldsymbol{u}$ is a unit vector and $\boldsymbol{u}^\perp$ is its normal, the terms in the cross-covariance matrix are*

$$\begin{aligned} k_{ij}(t^*, t^*) &= (-1)^i \int_0^{t^*}\int_0^{t^*} \boldsymbol{a}_i(t_1)^{\mathrm{T}}\, \boldsymbol{\partial}_{\boldsymbol{s}}^{i+j} K(\boldsymbol{\Delta}(t_1,t_2))\, \boldsymbol{a}_j(t_2)\, \|\boldsymbol{s}'(t_1)\|\, \|\boldsymbol{s}'(t_2)\|\, dt_1\, dt_2, \\ &= (-1)^i\, t^* \int_{-t^*}^{t^*} \boldsymbol{a}_i^{\mathrm{T}}\, \boldsymbol{\partial}_{\boldsymbol{s}}^{i+j} K(x\boldsymbol{u})\, \boldsymbol{a}_j\, dx, \quad i,j = 1,2, \end{aligned}$$

where $\boldsymbol{a}_1(t) = \boldsymbol{n}(\boldsymbol{s}(t))$ is the normal to the segment, $\boldsymbol{a}_2(t) = \mathscr{E}_2\, \boldsymbol{n}(\boldsymbol{s}(t)) \otimes \boldsymbol{n}(\boldsymbol{s}(t))$, where $\mathscr{E}_2 = \begin{pmatrix} 1 & & & \\ & 1 & 1 & \\ & & & 1 \end{pmatrix}$ is an elimination matrix and $\boldsymbol{\Delta}(t_1,t_2) = \boldsymbol{s}_2(t) - \boldsymbol{s}_1(t)$.

Proof. Considering the parametric segment, $C_{t^*} = \{\boldsymbol{s}_0 + t\boldsymbol{u} : t \in [0, t^*]\}$, where $\boldsymbol{u} = (u_1, u_2)^{\mathrm{T}}$ is a unit vector, $\|\boldsymbol{u}\| = 1$, $\boldsymbol{u}^\perp$ is its normal, $\boldsymbol{u}^{\mathrm{T}}\, \boldsymbol{u}^\perp = 0$, $\boldsymbol{a}_1(t) = \boldsymbol{a}_1 = \boldsymbol{u}^\perp$ and $\boldsymbol{a}_2(t) = \boldsymbol{a}_2 = \mathscr{E}_2(\boldsymbol{u}^\perp \otimes \boldsymbol{u}^\perp)$ are free of t, and $\boldsymbol{\Delta}(t_1,t_2) = (t_2 - t_1)\boldsymbol{u}$. The integrand in $k_{ij}(t^*,t^*) = (-1)^i \int_0^{t^*}\int_0^{t^*} \boldsymbol{a}_i^{\mathrm{T}}\, \boldsymbol{\partial}_{\boldsymbol{s}}^{i+j} K((t_2 - t_1)\boldsymbol{u})\, \boldsymbol{a}_j\, dt_1\, dt_2$, depends only on $(t_2 - t_1)$. Making a change of variable–define $x = t_2 - t_1$, $y = t_2 + t_1$, implying $\frac{x+y}{2} = t_2$ and $\frac{y-x}{2} = t_1$. The Jacobian is $\frac{1}{2}$. Hence, $0 \le y + x \le 2t^*$ and $0 \le y - x \le 2t^*$. This implies $0 \le y \le 2t^*$ and $-t^* \le x \le t^*$. Making the substitution above reduces, $k_{ij}(t^*,t^*) = \frac{(-1)^i}{2}\int_0^{2t^*}\int_{-t^*}^{t^*} \boldsymbol{a}_i^{\mathrm{T}}\, \boldsymbol{\partial}_{\boldsymbol{s}}^{i+j} K(x\boldsymbol{u})\, \boldsymbol{a}_j\, dx\, dy = (-1)^i\, t^* \int_{-t^*}^{t^*} \boldsymbol{a}_i^{\mathrm{T}}\, \boldsymbol{\partial}_{\boldsymbol{s}}^{i+j} K(x\boldsymbol{u})\, \boldsymbol{a}_j\, dx$. Setting $i = j = 1$, produces the scenario in Banerjee & Gelfand (2006). □

Proposition 7. *For the Matérn kernel, with $\nu = 3/2$, the variance of the gradient based wombling measure is $k_{11}(t^*, t^*) = 2\sqrt{3}\sigma^2\phi_s t^* G\left(1, \sqrt{3}\phi_s t^*\right)$. In case $\nu = 5/2$, the cross-covariance matrix for the wombling measures based on spatial gradient and curvature is constructed using $k_{11}(t^*, t^*) = \frac{2\sqrt{5}}{3}\sigma^2\phi_s\ t^*\left\{G\left(1, \sqrt{5}\phi_s t^*\right) + G\left(2, \sqrt{5}\phi_s t^*\right)\right\}$, $k_{22}(t^*, t^*) = 10\sqrt{5}\sigma^2\phi_s^3\ t^* G(1, \sqrt{5}\phi_s t^*)$ and $k_{21}(t^*, t^*) = -k_{12}(t^*, t^*) = 0$. For the Gaussian kernel,*

$$\mathbf{V}_{\boldsymbol{\Gamma}}(t^*) = 2\sigma^2\sqrt{\pi}\phi_s^{1/2}\ t^*\left\{2\Phi\left(\sqrt{2\phi_s}t^*\right) - 1\right\}\begin{pmatrix} 1 & 0 \\ 0 & 6\phi_s \end{pmatrix}.$$

In both cases $\mathbf{V}_{\boldsymbol{\Gamma}}(t^*)$ is a variance-covariance matrix.

Proof. Using Lemma 2, if $\nu = 3/2$, then we obtain $k_{11}(t^*, t^*) = 3\sigma^2\phi_s^2\ t^*\int_{-t^*}^{t^*} e^{-\sqrt{3}\phi_s|x|}\,dx = 2\sqrt{3}\sigma^2\phi_s t^*\ G(1, \sqrt{3}\phi_s t^*)$. If $\nu = 5/2$, then $k_{11}(t^*, t^*) = \frac{5}{3}\sigma^2\phi_s\ t^*\int_{-t^*}^{t^*}(1 + \sqrt{5}\phi_s|x|)\ e^{-\sqrt{5}\phi_s|x|}\,dx = \frac{2\sqrt{5}}{3}\sigma^2\phi_s\ t^*\left\{G\left(1, \sqrt{5}\phi_s t^*\right) + G\left(2, \sqrt{5}\phi_s t^*\right)\right\}$. For terms k_{21} and k_{12}, consider $a_1^{\mathrm{T}}\boldsymbol{\partial}_{\boldsymbol{s}}^3 K(x\boldsymbol{u})\ a_2 = \frac{25}{3}\sigma^2\phi_s e^{-\sqrt{5}\phi_s|x|}|x|\left\{\boldsymbol{a}_1^{\mathrm{T}}\mathbf{A}_1\boldsymbol{a}_2 - \sqrt{5}\phi_s|x|\ \boldsymbol{a}_1^{\mathrm{T}}\ \mathbf{A}_2\ \boldsymbol{a}_2\right\}$, where

$$\mathbf{A}_1 = \begin{pmatrix} 3u_1 & u_2 & u_1 \\ u_2 & u_1 & 3u_2 \end{pmatrix}, \quad \mathbf{A}_2 = \begin{pmatrix} u_1^3 & u_1^2\,u_2 & u_2^2\,u_1 \\ u_1^2\,u_2 & u_2^2\,u_1 & u_2^3 \end{pmatrix}.$$

The first and the second term both reduce to 0. Hence, $k_{21}(t^*, t^*) = -k_{12}(t^*, t^*) = 0$. Next, $\boldsymbol{a}_2^{\mathrm{T}}\boldsymbol{\partial}_{\boldsymbol{s}}^4 K(x\boldsymbol{u})\ \boldsymbol{a}_2 = \frac{25}{3}\sigma^2\phi_s^4\ e^{-\sqrt{5}\phi_s|x|}\left\{\boldsymbol{a}_2^{\mathrm{T}}\mathbf{A}_3\ \boldsymbol{a}_2 - \sqrt{5}\phi_s\ |x|\ \boldsymbol{a}_2^{\mathrm{T}}\mathbf{A}_4\left(\sqrt{5}\ \phi_s\ |x| + 1\right)\boldsymbol{a}_2\right\}$, where

$$\mathbf{A}_3 = \begin{pmatrix} 3 & 0 & 1 \\ 0 & 1 & 0 \\ 1 & 0 & 3 \end{pmatrix}, \quad \mathbf{A}_4(x') = \begin{pmatrix} 6\,u_1^2 - x'\,u_1^4 & (3-x'\,u_1^2)\,u_1\,u_2 & 1-x'\,u_1^2\,u_2^2 \\ (3-x'\,u_1^2)\,u_1\,u_2 & 1-x'\,u_1^2\,u_2^2 & (3-x'\,u_2^2)\,u_1\,u_2 \\ 1-x'\,u_1^2\,u_2^2 & (3-x'\,u_2^2)\,u_1\,u_2 & 6\,u_2^2 - x'\,u_2^4 \end{pmatrix}.$$

After some algebra, $\boldsymbol{a}_2^{\mathrm{T}}\ \boldsymbol{\partial}_{\boldsymbol{s}}^4 K(x\boldsymbol{u})\ \boldsymbol{a}_2 = 25\sigma^2\phi_s^4\ e^{-\sqrt{5}\phi_s|x|}\left\{1 - 2\sqrt{5}\ \phi_s\ |x|\ \boldsymbol{u}_1^{\perp}\ \boldsymbol{u}_2^{\perp}\ (\boldsymbol{u}_1\ \boldsymbol{u}_2 + \boldsymbol{u}_1^{\perp}\ \boldsymbol{u}_2^{\perp})\right\}$. Observe that $\boldsymbol{u}_1^{\perp} = \boldsymbol{u}_2$ and $\boldsymbol{u}_2^{\perp} = -\boldsymbol{u}_1$, substituting we get $k_{22}(t^*, t^*) = 10\sqrt{5}\sigma^2\phi_s^3\ t^* G(1, \sqrt{5}\phi_s t^*)$. For the Gaussian kernel, $k_{11}(t^*, t^*) = 2\sigma^2\phi_s\ t^*\int_{-t^*}^{t^*} e^{-\phi_s x^2}\,dx = 2\sigma^2\sqrt{\pi}\phi_s^{1/2}\ t^*\left\{2\Phi\left(\sqrt{2\phi_s}t^*\right) - 1\right\}$. Note that $\boldsymbol{a}_1^{\mathrm{T}}\boldsymbol{\partial}_{\boldsymbol{s}}^3 K(x\boldsymbol{u})\ \boldsymbol{a}_2 = 4\sigma^2\phi_s^2\ e^{-\phi_s x^2}x\left\{\boldsymbol{a}_1^{\mathrm{T}}\mathbf{A}_1\boldsymbol{a}_2 - 2\phi_s x^2\ \boldsymbol{a}_1^{\mathrm{T}}\ \mathbf{A}_2\ \boldsymbol{a}_2\right\}$. Again, the first and second terms equate to 0 implying $k_{21}(t^*, t^*) = -k_{12}(t^*, t^*) = 0$. Next, $\boldsymbol{a}_2^{\mathrm{T}}\boldsymbol{\partial}_{\boldsymbol{s}}^4 K(x\boldsymbol{u})\ \boldsymbol{a}_2 = 4\sigma^2\phi_s^2\ e^{-\phi_s x^2}\left\{\boldsymbol{a}_2^{\mathrm{T}}\mathbf{A}_3\ \boldsymbol{a}_2 - 2\phi_s\ x^2\ \boldsymbol{a}_2^{\mathrm{T}}\mathbf{A}_4(2\phi_s x^2)\ \boldsymbol{a}_2\right\}$. After some algebra, $\boldsymbol{a}_2^{\mathrm{T}}\ \boldsymbol{\partial}_{\boldsymbol{s}}^4 K(x\boldsymbol{u})\ \boldsymbol{a}_2 = 12\sigma^2\phi_s^2\ e^{-\phi_s x^2}\left\{1 - 4\ \phi_s\ x^2\ \boldsymbol{u}_1^{\perp}\ \boldsymbol{u}_2^{\perp}\ (\boldsymbol{u}_1\ \boldsymbol{u}_2 + \boldsymbol{u}_1^{\perp}\ \boldsymbol{u}_2^{\perp})\right\}$. Substituting, we get $k_{22}(t^*, t^*) = 12\sigma^2\sqrt{\pi}\phi_s^{3/2}t^*\left\{2\Phi\left(\sqrt{2\phi_s}t^*\right) - 1\right\}$ resulting in the required expression for $\mathbf{V}_{\boldsymbol{\Gamma}}(t^*)$. □

The covariance matrix for the Gaussian case has a simpler form than a Matérn with $\nu = 5/2$. The required expressions for $\partial_{\boldsymbol{s}}^{i+j} K(x\boldsymbol{u})$, $i, j = 1, 2$, used for the above construction can be found in sections S12.1.1 and S12.1.2 (also see Section S3, Supplement of Halder et al. (2024)). Finally, considering the covariance between $Z(\boldsymbol{s}_i)$, $i = 1, \ldots, N$ and the wombling measures—in the Gaussian case, closed-forms are available (see, e.g., Halder et al. 2024, end of Section 3). Therefore, the inferential exercise of spatial wombling does not require quadrature when using a Gaussian kernel. However, only one-dimensional quadrature is required for the same when a Matérn kernel is used.

S10.2 Spatiotemporal

In spatiotemporal wombling, the surface is partitioned using parametric triangular planes. The wombling measures are computed on each triangular plane. Their variance involves a four-dimensional integral (see eq. (8) in the manuscript). The integrand depends on $(\omega_1, \omega_2, v_1, v_2)$ through $(\omega_2 - \omega_1)$ and $(v_2 - v_1)$. The next lemma extends Lemma 2.

Lemma 3. *For a wombling surface $\mathscr{C}$ triangulated using a partition $\mathscr{P}$ as in Theorem 2, the cross-covariance for the wombling measures $\boldsymbol{\Gamma}(\mathscr{C}_{ijk})$ on the triangular plane, $T = \{(\omega, v) : 0 \le \omega + v \le 1\}$ satisfies the following identity,*

$$\begin{aligned}
\mathbf{K}_{\boldsymbol{\Gamma}}(\mathscr{C}_{ijk}, \mathscr{C}_{ijk}) &= \iiiint\limits_{T \times T} \mathbf{N}_{ijk,\boldsymbol{st}}\; \mathbf{V}_{\mathcal{L}^* Z}(\boldsymbol{\Delta}_k(\omega_1, \omega_2, v_1, v_2), \delta_k(v_1, v_2))\; \mathbf{N}_{ijk,\boldsymbol{st}}^{\mathrm{T}}\; \|\overline{\boldsymbol{n}}_{ijk}\|^2\; d\omega\; dv \\
&= \frac{\|\overline{\boldsymbol{n}}_{ijk}\|^2}{2} \int_{-1}^{1} \int_{-1-x_v}^{1-x_v} \mathbf{N}_{ijk,\boldsymbol{st}}\; \mathbf{V}_{\mathcal{L}^* Z}(\boldsymbol{\Delta}_k(x_\omega, x_v), \delta_k(x_v))\; \mathbf{N}_{ijk,\boldsymbol{st}}^{\mathrm{T}}\; dx_\omega\; dx_v,
\end{aligned}$$

where , $i = 1, \ldots, n_\omega - 1$, $j = 1, \ldots, n_v - 1$, $k = 1, 2$, $x_\omega = \omega_2 - \omega_1$ and $x_v = v_2 - v_1$ and the integral acts element-wise on matrices.

Proof. We make the following change of variables, $x_\omega = \omega_2 - \omega_1$, $y_\omega = \omega_2 + \omega_1$, $x_v = v_2 - v_1$, $y_v = v_2 + v_1$. As a result, $\frac{x_\omega + y_\omega}{2} = \omega_2$, $\frac{y_\omega - x_\omega}{2} = \omega_1$, $\frac{x_v + y_v}{2} = v_2$ and $\frac{y_v - x_v}{2} = v_1$. The Jacobian is $\frac{1}{4}$. Since $0 \le \omega_k + v_k \le 1$ for $k = 1, 2$ the above change of variables also results in

$0 \leq y_\omega + y_v \leq 2$ and $-1 \leq x_\omega + x_v \leq 1$. $\mathbf{N}_{ijk,\boldsymbol{st}}$ is free of (ω_k, v_k) for every $i = 1, \ldots, n_\omega - 1$, $j = 1, \ldots, n_v - 1$ and $k = 1, 2$ (see Section 4.2 of the manuscript). Making the substitution, $K_{\boldsymbol{\Gamma}}(\mathscr{C}_{ijk}, \mathscr{C}_{ijk}) = \frac{\|n_{ijk}\|^2}{4} \int_0^2 \int_0^{2-y_v} \int_{-1}^1 \int_{-1-x_v}^{1-x_v} \mathbf{N}_{ijk,\boldsymbol{st}}\ \mathbf{V}_{\mathcal{L}^* Z}(\boldsymbol{\Delta}_k(x_\omega, x_v), \delta_k(x_v))\ \mathbf{N}_{ijk,\boldsymbol{st}}^{\mathrm{T}}\ dx_\omega\ dx_v\ dy_\omega\ dy_v = \frac{\|\boldsymbol{n}_{ijk}\|^2}{2} \int_{-1}^1 \int_{-1-x_v}^{1-x_v} \mathbf{N}_{ijk,\boldsymbol{st}}\ \mathbf{V}_{\mathcal{L}^* Z}(\boldsymbol{\Delta}_k(x_\omega, x_v), \delta_k(x_v))\ \mathbf{N}_{ijk,\boldsymbol{st}}^{\mathrm{T}}\, dx_\omega\, dx_v$. □

The identity in the above lemma reduces an integration in four dimensions to two dimension thereby easing the computational burden associated with high dimensional quadrature. Subsequently, Lemma 3 finds use for analytic evaluation of the cross-covariance matrix when obtaining posterior samples of the wombling measures (see Algorithm 4 for details). Further simplifications for the Gaussian kernel are outlined in the next discussion.

S10.3 Non-separable Gaussian Kernel

We consider elements of the 8×1 vector, $\boldsymbol{\gamma}(\mathscr{C}_{ijk}) = \begin{pmatrix} \gamma_1 \\ \vdots \\ \gamma_8 \end{pmatrix}$ computed over the triangular region, $T = \{(\omega, v) : 0 \leq \omega + v < 1\}$ for any $i = 1, \ldots, n_\omega - 1$, $j = 1, \ldots, n_v - 1$, $k = 1, 2$ and an arbitrary point $(\boldsymbol{s}_{i'}, t_{i'})$, $i' = 1, \ldots, N$. We first recap the notation. Let $\boldsymbol{\Delta}_{i'1}(\omega, v) = \boldsymbol{\Delta}_{i'ij} - \omega \boldsymbol{u}_{ij} - v \boldsymbol{v}_{ij+1}$, $\boldsymbol{\Delta}_{i'2}(\omega, v) = \boldsymbol{\Delta}_{i'i+1j+1} - \omega \boldsymbol{u}_{ij+1} - v \boldsymbol{v}_{ij}$, $\delta_{i'1}(v) = \delta_{i'j} - v\ \delta_{j+1j}$ and $\delta_{i'2}(v) = \delta_{i'j+1} + v\ \delta_{j+1j}$ for $i' = 1, \ldots, N$, where $\boldsymbol{\Delta}_{i'ij} = \boldsymbol{s}_{i'} - \boldsymbol{s}(\omega_i', t_j)$, $\boldsymbol{\Delta}_{i'i+1j+1} = \boldsymbol{s}_{i'} - \boldsymbol{s}(\omega_{i+1}', t_{j+1})$, $\delta_{i'j} = t_{i'} - t_j$, $\delta_{i'j+1} = t_{i'} - t_{j+1}$ and $\delta_{jj+1} = t_{j+1} - t_j$.

Proposition 8. *Let $Z(\boldsymbol{s}, t) \sim GP(0, K(\cdot, \cdot; \boldsymbol{\theta}))$ where K is the Gaussian kernel. For a wombling surface, $\mathscr{C}$, upon triangulation (see Section 4.2), analytic evaluation of $\mathbf{G}(\mathscr{C})$ and $\mathbf{K}_{\boldsymbol{\Gamma}}(\mathscr{C}, \mathscr{C})$ (see eq. (10)) requires only 1-dimensional quadrature.*

Proof. First we note that for a Gaussian kernel $\mathcal{L}^* Z(\boldsymbol{s}, t)$ is well-defined. We focus on one triangular plane. The calculation for the other plane is similar and hence mentioned in brief. We refer to Section S12.2.3 for required expressions of $\partial_t^j \boldsymbol{\partial}_{\boldsymbol{s}}^{r-j} \widetilde{K}$, $j = 0, \ldots, r$, $r = 1, 2$.

Triangular plane 1: Here $k = 1$. The normal to the plane is given by $\overline{\boldsymbol{n}}_{ij1} = \binom{\boldsymbol{v}_{ij+1}}{1} \times \binom{\boldsymbol{u}_{ij}}{0} = \begin{pmatrix} -u_{ij2} \\ u_{ij1} \\ v_{ij+11}u_{ij2} - v_{ij+12}u_{ij1} \end{pmatrix} = \begin{pmatrix} \boldsymbol{u}_{ij}^{\perp} \\ -\boldsymbol{v}_{ij+1}^{\mathrm{T}}\boldsymbol{u}_{ij}^{\perp} \end{pmatrix} = \binom{\overline{\boldsymbol{n}}_{\boldsymbol{s}}}{\overline{n}_t}$, where $\boldsymbol{u}_{ij}^{\perp}$ is the normal to $\boldsymbol{u}_{ij}$, $\overline{\boldsymbol{n}}_{\boldsymbol{s}} = \boldsymbol{u}_{ij}^{\perp}$ and $\overline{n}_t = -\boldsymbol{v}_{ij+1}^{\mathrm{T}}\boldsymbol{u}_{ij}^{\perp}$. The unit normal is $\boldsymbol{n}_{ij1} = \frac{\overline{\boldsymbol{n}}_{ij1}}{\|\overline{\boldsymbol{n}}_{ij1}\|}$, where $\|\overline{\boldsymbol{n}}_{ij1}\| = \boldsymbol{u}_{ij}^{\perp\mathrm{T}}\boldsymbol{u}_{ij}^{\perp} + \left(\boldsymbol{v}_{ij+1}^{\mathrm{T}}\boldsymbol{u}_{ij}^{\perp}\right)^{\mathrm{T}}\left(\boldsymbol{v}_{ij+1}^{\mathrm{T}}\boldsymbol{u}_{ij}^{\perp}\right) = \boldsymbol{u}_{ij}^{\perp\mathrm{T}}\left(\mathbf{I}_2 + \boldsymbol{v}_{ij+1}\boldsymbol{v}_{ij+1}^{\mathrm{T}}\right)\boldsymbol{u}_{ij}^{\perp}$. The following identities are required for subsequent simplifications

$$\boldsymbol{n}_s^{\mathrm{T}}\boldsymbol{\Delta}_{i'1}(\omega, v) = \frac{\boldsymbol{u}_{ij}^{\perp\mathrm{T}}}{\|\boldsymbol{n}_{ij1}\|}\left(\boldsymbol{\Delta}_{i'ij} - \omega\boldsymbol{u}_{ij} - v\boldsymbol{v}_{ij+1}\right) = \frac{\boldsymbol{u}_{ij}^{\perp\mathrm{T}}}{\|\boldsymbol{n}_{ij1}\|}\left(\boldsymbol{\Delta}_{i'ij} - v\boldsymbol{v}_{ij+1}\right) = \boldsymbol{n}_s^{\mathrm{T}}\boldsymbol{\Delta}(v), \tag{S6}$$

$$\begin{aligned}\|\boldsymbol{\Delta}_{i'1}(\omega, v)\|^2 &= \boldsymbol{\Delta}_{i'1}(\omega, v)^{\mathrm{T}}\boldsymbol{\Delta}_{i'1}(\omega, v) = \|\boldsymbol{u}_{ij}\|^2\left(\omega - \frac{\boldsymbol{u}_{ij}^{\mathrm{T}}\boldsymbol{\Delta}(v)}{\|\boldsymbol{u}_{ij}\|^2}\right)^2 + \boldsymbol{\Delta}(v)^{\mathrm{T}}\left(\mathbf{I}_2 - \frac{\boldsymbol{u}_{ij}\boldsymbol{u}_{ij}^{\mathrm{T}}}{\|\boldsymbol{u}_{ij}\|^2}\right)\boldsymbol{\Delta}(v) \\ &= \|\boldsymbol{u}_{ij}\|^2\left(\omega - \frac{\boldsymbol{u}_{ij}^{\mathrm{T}}\boldsymbol{\Delta}(v)}{\|\boldsymbol{u}_{ij}\|^2}\right)^2 + \boldsymbol{\Delta}(v)^{\mathrm{T}}\frac{\boldsymbol{u}_{ij}^{\perp}\boldsymbol{u}_{ij}^{\perp\mathrm{T}}}{\|\boldsymbol{u}_{ij}\|^2}\boldsymbol{\Delta}(v) = \|\boldsymbol{u}_{ij}\|^2\left(\omega - \frac{\boldsymbol{u}_{ij}^{\mathrm{T}}\boldsymbol{\Delta}(v)}{\|\boldsymbol{u}_{ij}\|^2}\right)^2 + \left(\frac{\boldsymbol{u}_{ij}^{\perp\mathrm{T}}\boldsymbol{\Delta}(v)}{\|\boldsymbol{u}_{ij}\|}\right)^2,\end{aligned} \tag{S7}$$

$$\begin{aligned}\|\boldsymbol{\Delta}_{i'1}(\omega, v)\|^4 &= \{\boldsymbol{\Delta}_{i'1}(\omega, v)^{\mathrm{T}}\boldsymbol{\Delta}_{i'1}(\omega, v)\}^2 \\ &= \left\{\|\boldsymbol{u}_{ij}\|^2\left(\omega - \frac{\boldsymbol{u}_{ij}^{\mathrm{T}}\boldsymbol{\Delta}(v)}{\|\boldsymbol{u}_{ij}\|^2}\right)^2 + \boldsymbol{\Delta}(v)^{\mathrm{T}}\left(\mathbf{I}_2 - \frac{\boldsymbol{u}_{ij}\boldsymbol{u}_{ij}^{\mathrm{T}}}{\|\boldsymbol{u}_{ij}\|^2}\right)\boldsymbol{\Delta}(v)\right\}^2 \\ &= \|\boldsymbol{u}_{ij}\|^4\left(\omega - \frac{\boldsymbol{u}_{ij}^{\mathrm{T}}\boldsymbol{\Delta}(v)}{\|\boldsymbol{u}_{ij}\|^2}\right)^4 + 2\left(\omega - \frac{\boldsymbol{u}_{ij}^{\mathrm{T}}\boldsymbol{\Delta}(v)}{\|\boldsymbol{u}_{ij}\|^2}\right)^2\boldsymbol{\Delta}(v)^{\mathrm{T}}\boldsymbol{u}_{ij}^{\perp}\boldsymbol{u}_{ij}^{\perp\mathrm{T}}\boldsymbol{\Delta}(v) + \left(\boldsymbol{\Delta}(v)^{\mathrm{T}}\frac{\boldsymbol{u}_{ij}^{\perp}\boldsymbol{u}_{ij}^{\perp\mathrm{T}}}{\|\boldsymbol{u}_{ij}\|^2}\boldsymbol{\Delta}(v)\right)^2 \\ &= \|\boldsymbol{u}_{ij}\|^4\left(\omega - \frac{\boldsymbol{u}_{ij}^{\mathrm{T}}\boldsymbol{\Delta}(v)}{\|\boldsymbol{u}_{ij}\|^2}\right)^4 + 2\left(\omega - \frac{\boldsymbol{u}_{ij}^{\mathrm{T}}\boldsymbol{\Delta}(v)}{\|\boldsymbol{u}_{ij}\|^2}\right)^2\left(\boldsymbol{u}_{ij}^{\perp\mathrm{T}}\boldsymbol{\Delta}(v)\right)^2 + \left(\frac{\boldsymbol{u}_{ij}^{\perp\mathrm{T}}\boldsymbol{\Delta}(v)}{\|\boldsymbol{u}_{ij}\|}\right)^4.\end{aligned} \tag{S8}$$

Using eqs. (S6) and (S7), the entry corresponding to the spatial gradient satisfies

$$\begin{aligned}\gamma_1 &= \iint_T -2\,\sigma^2\frac{\phi_s^2}{A_t^2}\exp\left(-\frac{\phi_s^2}{A_t}\|\boldsymbol{\Delta}_{i'1}(\omega, v)\|^2\right)\,\boldsymbol{n}_s^{\mathrm{T}}\,\boldsymbol{\Delta}_{i'1}(\omega, v)\,\|\overline{\boldsymbol{n}}_{ij1}\|\,d\omega\,dv \\ &= \int_0^1 -2\sigma^2\frac{\phi_s^2}{A_t^2}\exp\left\{-\frac{\phi_s^2}{A_t}\left(\frac{\boldsymbol{u}_{ij}^{\perp\mathrm{T}}\boldsymbol{\Delta}(v)}{\|\boldsymbol{u}_{ij}\|}\right)^2\right\}\boldsymbol{n}_s^{\mathrm{T}}\boldsymbol{\Delta}(v)\,\|\overline{\boldsymbol{n}}_{ij1}\| \\ &\qquad\qquad \int_0^{1-v}\exp\left\{-\frac{\phi_s^2}{A_t}\|\boldsymbol{u}_{ij}\|^2\left(\omega - \frac{\boldsymbol{u}_{ij}^{\mathrm{T}}\boldsymbol{\Delta}(v)}{\|\boldsymbol{u}_{ij}\|^2}\right)^2\right\}\,d\omega\,dv \\ &= \int_0^1 a(v)\,F_1(v)\,\boldsymbol{n}_s^{\mathrm{T}}\boldsymbol{\Delta}(v)\,dv,\end{aligned} \tag{S9}$$

where $A_t = 1 + \phi_t^2\ |\delta_{i'1}(v)|^2$, $a(v) = a(v;\boldsymbol{\theta},\boldsymbol{n}_s) = -2\sigma^2 \frac{\sqrt{\pi}\phi_s}{A_t^{3/2}}\ \exp\left\{-\frac{\phi_s^2}{A_t}\left(\frac{\boldsymbol{u}_{ij}^{\perp\mathrm{T}}\boldsymbol{\Delta}(v)}{\|\boldsymbol{u}_{ij}\|}\right)^2\right\}\frac{\|\overline{\boldsymbol{n}}_{ij1}\|}{\|\boldsymbol{u}_{ij}\|}$ and $F_1(v) = F_1(v;\phi_s,\phi_t) = \Phi(a_2(v))+\Phi(-a_1(v))-1$, where $a_2(v) = a_2(v;\phi_s,\phi_t) = \frac{\sqrt{2}\phi_s}{A_t^{1/2}}\|\boldsymbol{u}_{ij}\|\left(1 - v - \frac{\boldsymbol{u}_{ij}^{\mathrm{T}}\boldsymbol{\Delta}(v)}{\|\boldsymbol{u}_{ij}\|^2}\right)$ and $a_1(v) = a_1(v;\phi_s,\phi_t) = -\frac{\sqrt{2}\phi_s}{A_t^{1/2}}\frac{\boldsymbol{u}_{ij}^{\mathrm{T}}\boldsymbol{\Delta}(v)}{\|\boldsymbol{u}_{ij}\|}$. Similarly, for spatial curvature, we have

$$\begin{aligned}\gamma_2 &= \iint_T -2\,\sigma^2\,\frac{\phi_s^2}{A_t^2}\ \exp\left(-\frac{\phi_s^2}{A_t}\|\boldsymbol{\Delta}_{i'1}(\omega,v)\|^2\right)\left(\|\boldsymbol{n}_s\|^2 - 2\frac{\phi_s^2}{A_t}\boldsymbol{n}_s^{\mathrm{T}}\boldsymbol{\Delta}(v)\boldsymbol{\Delta}(v)^{\mathrm{T}}\boldsymbol{n}_s\right)\ \|\overline{\boldsymbol{n}}_{ij1}\|\ d\omega\ dv\\ &= \int_0^1 a(v)\ F_1(v)\ \left(\|\boldsymbol{n}_s\|^2 - 2\frac{\phi_s^2}{A_t}\boldsymbol{n}_s^{\mathrm{T}}\boldsymbol{\Delta}(v)\boldsymbol{\Delta}(v)^{\mathrm{T}}\boldsymbol{n}_s\right)\ dv. \qquad \text{(S10)}\end{aligned}$$

Identities eqs. (S9) and (S10) serve as spatiotemporal extensions for the results found at the end of Section 3 in Halder et al. (2024). For the temporal derivative, we can write $\partial_t\widetilde{K} = -2\sigma^2\frac{\phi_t^2}{A_t^2}\exp\left(-\frac{\phi_s^2}{A_t}\|\boldsymbol{\Delta}_{i'1}(\omega,v)\|^2\right)\ \delta_{i'1}(v)+2\sigma^2\frac{\phi_t^2}{A_t^2}\exp\left(-\frac{\phi_s^2}{A_t}\|\boldsymbol{\Delta}_{i'1}(\omega,v)\|^2\right)\frac{\phi_s^2}{A_t}\|\boldsymbol{\Delta}_{i'1}(\omega,v)\|^2\delta_{i'1}(v)$. The cross-covariance has the following expression

$$\begin{aligned}\gamma_3 = &\iint_T -2\,\sigma^2\,\frac{\phi_t^2}{A_t^2}\ \exp\left(-\frac{\phi_s^2}{A_t}\|\boldsymbol{\Delta}_{i'1}(\omega,v)\|^2\right)\ n_t\ \delta_{i'1}(v)\ \|\boldsymbol{n}_{ij1}\|\ d\omega\ dv\ +\\ &\iint_T 2\,\sigma^2\,\frac{\phi_t^2}{A_t^2}\ \exp\left(-\frac{\phi_s^2}{A_t}\|\boldsymbol{\Delta}_{i'1}(\omega,v)\|^2\right)\ \frac{\phi_s^2}{A_t}\ \|\boldsymbol{\Delta}_{i'1}(\omega,v)\|^2\ n_t\ \delta_{i'1}(v)\ \|\boldsymbol{n}_{ij1}\|\ d\omega\ dv.\end{aligned}$$

The first integral when evaluated using similar algebra as the preceding calculation produces $\int_0^1 a(v)\ \frac{\phi_t^2}{\phi_s^2}\ F_1(v)\ n_t\ \delta_{i'1}(v)\ dv$. We evaluate the second integral,

$$\begin{aligned}&\iint_T 2\,\sigma^2\,\frac{\phi_t^2}{A_t^2}\ \exp\left(-\frac{\phi_s^2}{A_t}\|\boldsymbol{\Delta}_{i'1}(\omega,v)\|^2\right)\ \frac{\phi_s^2}{A_t}\ \|\boldsymbol{\Delta}_{i'1}(\omega,v)\|^2\ n_t\ \delta_{i'1}(v)\ \|\boldsymbol{n}_{ij1}\|\ d\omega\ dv\\ &= \int_0^1 2\sigma^2\frac{\phi_t^2}{A_t^2}\frac{\phi_s^2}{A_t}\left(\frac{\boldsymbol{u}_{ij}^{\perp\mathrm{T}}\boldsymbol{\Delta}(v)}{\|\boldsymbol{u}_{ij}\|}\right)^2\exp\left\{-\frac{\phi_s^2}{A_t}\left(\frac{\boldsymbol{u}_{ij}^{\perp\mathrm{T}}\boldsymbol{\Delta}(v)}{\|\boldsymbol{u}_{ij}\|}\right)^2\right\}\ n_t\ \delta_{i'1}(v)\ \|\overline{\boldsymbol{n}}_{ij1}\|\\ &\qquad\int_0^{1-v}\exp\left\{-\frac{\phi_s^2}{A_t}\|\boldsymbol{u}_{ij}\|^2\left(\omega - \frac{\boldsymbol{u}_{ij}^{\mathrm{T}}\boldsymbol{\Delta}(v)}{\|\boldsymbol{u}_{ij}\|^2}\right)^2\right\}d\omega\ dv\ +\\ &\quad\int_0^1 2\sigma^2\frac{\phi_t^2}{A_t^2}\exp\left\{-\frac{\phi_s^2}{A_t}\left(\frac{\boldsymbol{u}_{ij}^{\perp\mathrm{T}}\boldsymbol{\Delta}(v)}{\|\boldsymbol{u}_{ij}\|}\right)^2\right\}\ n_t\ \delta_{i'1}(v)\ \|\overline{\boldsymbol{n}}_{ij1}\|\\ &\qquad\int_0^{1-v}\frac{\phi_s^2}{A_t}\|\boldsymbol{u}_{ij}\|^2\left(\omega - \frac{\boldsymbol{u}_{ij}^{\mathrm{T}}\boldsymbol{\Delta}(v)}{\|\boldsymbol{u}_{ij}\|^2}\right)^2\ \exp\left\{-\frac{\phi_s^2}{A_t}\|\boldsymbol{u}_{ij}\|^2\left(\omega - \frac{\boldsymbol{u}_{ij}^{\mathrm{T}}\boldsymbol{\Delta}(v)}{\|\boldsymbol{u}_{ij}\|^2}\right)^2\right\}d\omega\ dv\end{aligned}$$

$$= -\int_0^1 a(v)\frac{\phi_t^2}{A_t}\left(\frac{\boldsymbol{u}_{ij}^{\perp\mathrm{T}}\boldsymbol{\Delta}(v)}{\|\boldsymbol{u}_{ij}\|}\right)^2 F_1(v)\; n_t\; \delta_{i'1}(v)\; dv \; -$$

$$\int_0^1 a(v)\frac{\phi_t^2}{\phi_s^2}\,\frac{1}{4}\left\{G\left(\frac{3}{2},\frac{a_2(v)^2}{2}\right) - G\left(\frac{3}{2},\frac{a_1(v)^2}{2}\right)\right\}\; n_t\; \delta_{i'1}(v)\; dv$$

$$= -\int_0^1 a(v)\;\frac{\phi_t^2}{\phi_s^2}\left\{\frac{\phi_s^2}{A_t}\left(\frac{\boldsymbol{u}_{ij}^{\perp\mathrm{T}}\boldsymbol{\Delta}(v)}{\|\boldsymbol{u}_{ij}\|}\right)^2 F_1(v) + \frac{1}{4}F_2(v)\right\}\; n_t\; \delta_{i'1}(v)\; dv,$$

where $F_2(v) = F_2(v;\phi_s,\phi_t) = G\left(\frac{3}{2},\frac{a_2(v)^2}{2}\right) - G\left(\frac{3}{2},\frac{a_1(v)^2}{2}\right)$. Combining the two terms yield

$$\gamma_3 = \int_0^1 a(v)\;\frac{\phi_t^2}{\phi_s^2}\;\left[\left\{1 - \frac{\phi_s^2}{A_t}\left(\frac{\boldsymbol{u}_{ij}^{\perp\mathrm{T}}\boldsymbol{\Delta}(v)}{\|\boldsymbol{u}_{ij}\|}\right)^2\right\}F_1(v) - \frac{1}{4}F_2(v)\right]\; n_t\; \delta_{i'1}(v)\; dv. \qquad \text{(S11)}$$

For the term corresponding to the spatial-temporal derivative the covariance is given by $\partial_t\boldsymbol{\partial_s}\widetilde{K} = 4\sigma^2\frac{\phi_s^2\phi_t^2}{A_t^3}\exp\left(-\frac{\phi_s^2\|\boldsymbol{\Delta}\|^2}{A_t}\right)\left(2 - \frac{\phi_s^2\|\boldsymbol{\Delta}\|^2}{A_t}\right)\boldsymbol{\Delta}\delta$. Hence,

$$\gamma_4 = \iint_T 8\sigma^2\frac{\phi_s^2\phi_t^2}{A_t^3}\exp\left(-\frac{\phi_s^2}{A_t}\|\boldsymbol{\Delta}_{i'1}(\omega,v)\|^2\right)\boldsymbol{n}_s^{\mathrm{T}}\boldsymbol{\Delta}(v)\; n_t\delta_{i'1}(v)\; \|\overline{\boldsymbol{n}}_{ij1}\|\; d\omega\; dv \; -$$

$$\iint_T 4\sigma^2\frac{\phi_s^4\phi_t^2}{A_t^4}\|\boldsymbol{\Delta}_{i'1}(\omega,v)\|^2\exp\left(-\frac{\phi_s^2}{A_t}\|\boldsymbol{\Delta}_{i'1}(\omega,v)\|^2\right)\boldsymbol{n}_s^{\mathrm{T}}\boldsymbol{\Delta}(v)\; n_t\delta_{i'1}(v)\; \|\overline{\boldsymbol{n}}_{ij1}\|\; d\omega\; dv.$$

The first term reduces to $-\int_0^1 4\; a(v)\;\frac{\phi_t^2}{A_t^2}\; F_1(v)\; \boldsymbol{n}_s^{\mathrm{T}}\boldsymbol{\Delta}(v)\; n_t\; \delta_{i'1}(v)\; dv$. The second term is $\int_0^1 2\;\frac{\phi_t^2}{A_t}\; a(v)\left\{-\frac{\phi_s^2}{A_t}\left(\frac{\boldsymbol{u}_{ij}^{\perp\mathrm{T}}\boldsymbol{\Delta}(v)}{\|\boldsymbol{u}_{ij}\|}\right)^2 F_1(v) - \frac{1}{4}F_2(v)\right\}\; \boldsymbol{n}_s^{\mathrm{T}}\boldsymbol{\Delta}(v)\; n_t\delta_{i'1}(v)\; dv$ following the previous calculation for γ_3. Combining the terms, we have

$$\gamma_4 = \int_0^1 2\; a(v)\;\frac{\phi_t^2}{A_t}\left[\left\{\frac{\phi_s^2}{A_t}\left(\frac{\boldsymbol{u}_{ij}^{\perp\mathrm{T}}\boldsymbol{\Delta}(v)}{\|\boldsymbol{u}_{ij}\|}\right)^2 - \frac{2}{A_t}\right\}F_1(v)\; + \frac{1}{4}F_2(v)\right]\; \boldsymbol{n}_s^{\mathrm{T}}\boldsymbol{\Delta}(v)\; n_t\delta_{i'1}(v)\; dv. \qquad \text{(S12)}$$

Next, for γ_5 associated with the temporal derivative of spatial curvature, we require $\partial_t\boldsymbol{\partial}_{\boldsymbol{s}}^2\widetilde{K}(\boldsymbol{\Delta},\delta) = 4\sigma^2\frac{\phi_s^2\phi_t^2}{A_t^3}\exp\left(-\frac{\phi_s^2}{A_t}\|\boldsymbol{\Delta}\|^2\right)\left\{\left(2 - \frac{\phi_s^2\|\boldsymbol{\Delta}\|^2}{A_t}\right)\mathbf{I}_2 - 2\frac{\phi_s^2}{A_t}\left(3 - \frac{\phi_s^2\|\boldsymbol{\Delta}\|^2}{A_t}\right)\boldsymbol{\Delta}\boldsymbol{\Delta}^{\mathrm{T}}\right\}\delta$. Hence,

$$\gamma_5 = \iint\limits_T n_t n_s^{\mathrm{T}} \partial_t \boldsymbol{\partial}_{\boldsymbol{s}}^2 \widetilde{K}(\boldsymbol{\Delta}, \delta) \boldsymbol{n}_s \, \|\overline{\boldsymbol{n}}_{ij1}\| \, d\omega \, dv$$

$$= \int_0^1 2\, a(v) \, \frac{\phi_t^2}{A_t} \left[\left\{ \left(\frac{\phi_s^2}{A_t} \left(\frac{\boldsymbol{u}_{ij}^{\perp\mathrm{T}} \boldsymbol{\Delta}(v)}{\|\boldsymbol{u}_{ij}\|} \right)^2 - \frac{2}{A_t} \right) F_1(v) + \frac{1}{4} F_2(v) \right\} \|\boldsymbol{n}_s\|^2 - \right.$$

$$\left. \frac{2\phi_s^2}{A_t} \left\{ \left(\frac{\phi_s^2}{A_t} \left(\frac{\boldsymbol{u}_{ij}^{\perp\mathrm{T}} \boldsymbol{\Delta}(v)}{\|\boldsymbol{u}_{ij}\|} \right)^2 - \frac{3}{A_t} \right) F_1(v) + \frac{1}{4} F_2(v) \right\} \boldsymbol{n}_s^{\mathrm{T}} \boldsymbol{\Delta}(v) \boldsymbol{\Delta}(v)^{\mathrm{T}} \boldsymbol{n}_s \right] n_t \delta_{i'1}(v) \, dv. \quad \text{(S13)}$$

Next, for γ_6 associated with the temporal curvature, $\partial_t^2 \widetilde{K} = -2\sigma^2 \frac{\phi_t^2}{A_t^2} \exp\left(-\frac{\phi_s^2 \|\boldsymbol{\Delta}\|^2}{A_t}\right)\left(1 - \frac{\phi_s^2 \|\boldsymbol{\Delta}\|^2}{A_t}\right) + 4\,\sigma^2 \frac{\phi_t^4 \delta^2}{A_t^3} \exp\left(-\frac{\phi_s^2 \|\boldsymbol{\Delta}\|^2}{A_t}\right)\left(2 - 4\frac{\phi_s^2 \|\boldsymbol{\Delta}\|^2}{A_t} + \frac{\phi_s^4 \|\boldsymbol{\Delta}\|^4}{A_t^2}\right)$ is required with $\gamma_6 = \iint\limits_T n_t^2 \partial_t^2 \widetilde{K}(\boldsymbol{\Delta}, \delta) \, \|\overline{\boldsymbol{n}}_{ij1}\| \, d\omega \, dv$. Evaluation of the terms in γ_6 follows a similar algebra as seen in the previous calculations with the last term being an exception. We use eqs. (S6) to (S8) to simplify the last term,

$$\iint\limits_T 4\sigma^2 \frac{\phi_t^4}{A_t^3} \, \exp\left(-\frac{\phi_s^2}{A_t} \|\boldsymbol{\Delta}_{i'1}(\omega, v)\|^2 \right) \, \frac{\phi_s^4}{A_t^2} \, \|\boldsymbol{\Delta}_{i'1}(\omega, v)\|^4 \, n_t^2 \, \delta_{i'1}^2(v) \, \|\boldsymbol{n}_{ij1}\| \, d\omega \, dv$$

$$= 2 \int_0^1 2\sigma^2 \frac{\phi_t^4}{A_t^3} \frac{\phi_s^4}{A_t^2} \left(\frac{\boldsymbol{u}_{ij}^{\perp\mathrm{T}} \boldsymbol{\Delta}(v)}{\|\boldsymbol{u}_{ij}\|} \right)^4 \exp\left\{ -\frac{\phi_s^2}{A_t} \left(\frac{\boldsymbol{u}_{ij}^{\perp\mathrm{T}} \boldsymbol{\Delta}(v)}{\|\boldsymbol{u}_{ij}\|} \right)^2 \right\} \, n_t^2 \, \delta_{i'1}(v)^2 \, \|\overline{\boldsymbol{n}}_{ij1}\|$$

$$\int_0^{1-v} \exp\left\{ -\frac{\phi_s^2}{A_t} \|\boldsymbol{u}_{ij}\|^2 \left(\omega - \frac{\boldsymbol{u}_{ij}^{\mathrm{T}} \boldsymbol{\Delta}(v)}{\|\boldsymbol{u}_{ij}\|^2} \right)^2 \right\} d\omega \, dv +$$

$$4 \int_0^1 2\sigma^2 \frac{\phi_t^4}{A_t^3} \exp\left\{ -\frac{\phi_s^2}{A_t} \left(\frac{\boldsymbol{u}_{ij}^{\perp\mathrm{T}} \boldsymbol{\Delta}(v)}{\|\boldsymbol{u}_{ij}\|} \right)^2 \right\} \, n_t^2 \, \delta_{i'1}(v)^2 \, \|\overline{\boldsymbol{n}}_{ij1}\|$$

$$\int_0^{1-v} \frac{\phi_s^4}{A_t^2} \left(\frac{\boldsymbol{u}_{ij}^{\perp\mathrm{T}} \boldsymbol{\Delta}(v)}{\|\boldsymbol{u}_{ij}\|} \right)^2 \|\boldsymbol{u}_{ij}\|^2 \left(\omega - \frac{\boldsymbol{u}_{ij}^{\mathrm{T}} \boldsymbol{\Delta}(v)}{\|\boldsymbol{u}_{ij}\|^2} \right)^2 \exp\left\{ -\frac{\phi_s^2}{A_t} \|\boldsymbol{u}_{ij}\|^2 \left(\omega - \frac{\boldsymbol{u}_{ij}^{\mathrm{T}} \boldsymbol{\Delta}(v)}{\|\boldsymbol{u}_{ij}\|^2} \right)^2 \right\} d\omega \, dv +$$

$$2 \int_0^1 2\sigma^2 \frac{\phi_t^4}{A_t^3} \exp\left\{ -\frac{\phi_s^2}{A_t} \left(\frac{\boldsymbol{u}_{ij}^{\perp\mathrm{T}} \boldsymbol{\Delta}(v)}{\|\boldsymbol{u}_{ij}\|} \right)^2 \right\} \, n_t^2 \, \delta_{i'1}(v)^2 \, \|\overline{\boldsymbol{n}}_{ij1}\|$$

$$\int_0^{1-v} \frac{\phi_s^4}{A_t^2} \|\boldsymbol{u}_{ij}\|^4 \left(\omega - \frac{\boldsymbol{u}_{ij}^{\mathrm{T}} \boldsymbol{\Delta}(v)}{\|\boldsymbol{u}_{ij}\|^2} \right)^4 \exp\left\{ -\frac{\phi_s^2}{A_t} \|\boldsymbol{u}_{ij}\|^2 \left(\omega - \frac{\boldsymbol{u}_{ij}^{\mathrm{T}} \boldsymbol{\Delta}(v)}{\|\boldsymbol{u}_{ij}\|^2} \right)^2 \right\} d\omega \, dv$$

$$= -2 \int_0^1 a(v) \frac{\phi_t^4 \phi_s^2}{A_t^3} \left(\frac{\boldsymbol{u}_{ij}^{\perp\mathrm{T}} \boldsymbol{\Delta}(v)}{\|\boldsymbol{u}_{ij}\|} \right)^4 F_1(v) \, n_t^2 \, \delta_{i'1}(v)^2 \, dv -$$

$$\int_0^1 a(v) \frac{\phi_t^4}{A_t^2} \left(\frac{\boldsymbol{u}_{ij}^{\perp\mathrm{T}} \boldsymbol{\Delta}(v)}{\|\boldsymbol{u}_{ij}\|} \right)^2 F_2(v) \, n_t^2 \, \delta_{i'1}(v)^2 \, dv -$$

$$3\int_0^1 a(v)\frac{\phi_t^4}{\phi_s^2 A_t}\left\{G\left(\frac{5}{2},\frac{a_2(v)^2}{2}\right)-G\left(\frac{5}{2},\frac{a_1(v)^2}{2}\right)\right\} n_t^2\,\delta_{i'1}(v)^2\,dv$$

$$=-\int_0^1 a(v)\frac{\phi_t^4}{\phi_s^2 A_t}\left\{\frac{2\phi_s^4}{A_t^2}\left(\frac{\boldsymbol{u}_{ij}^{\perp\top}\boldsymbol{\Delta}(v)}{\|\boldsymbol{u}_{ij}\|}\right)^4 F_1(v)+\frac{\phi_s^2}{A_t}\left(\frac{\boldsymbol{u}_{ij}^{\perp\top}\boldsymbol{\Delta}(v)}{\|\boldsymbol{u}_{ij}\|}\right)^2 F_2(v)+3F_3(v)\right\} n_t^2\delta_{i'1}(v)^2\,dv,$$

where $F_3(v)=F_3(v;\phi_s,\phi_t)=G\left(\frac{5}{2},\frac{a_2(v)^2}{2}\right)-G\left(\frac{5}{2},\frac{a_1(v)^2}{2}\right)$. Collecting terms, the final expression for γ_6 is obtained as follows:

$$\gamma_6=\int_0^1 a(v)\frac{\phi_t^2}{\phi_s^2}\Bigg[\left\{\left(1-\frac{\phi_s^2}{A_t}\left(\frac{\boldsymbol{u}_{ij}^{\perp\top}\boldsymbol{\Delta}(v)}{\|\boldsymbol{u}_{ij}\|}\right)^2\right)F_1(v)-\frac{1}{4}F_2(v)\right\}n_t^2+$$
$$\frac{\phi_t^2}{A_t}\left\{\left(-4+8\frac{\phi_s^2}{A_t}\left(\frac{\boldsymbol{u}_{ij}^{\perp\top}\boldsymbol{\Delta}(v)}{\|\boldsymbol{u}_{ij}\|}\right)^2-2\frac{\phi_s^4}{A_t^2}\left(\frac{\boldsymbol{u}_{ij}^{\perp\top}\boldsymbol{\Delta}(v)}{\|\boldsymbol{u}_{ij}\|}\right)^4\right)F_1(v)+\right.$$
$$\left.\left(2-\frac{\phi_s^2}{A_t}\left(\frac{\boldsymbol{u}_{ij}^{\perp\top}\boldsymbol{\Delta}(v)}{\|\boldsymbol{u}_{ij}\|}\right)^2\right)F_2(v)-3F_3(v)\right\}n_t^2\delta_{i'1}(v)^2\Bigg]\,dv. \quad \text{(S14)}$$

Proceeding similarly for the temporal curvature in the spatial gradient, we have

$$\gamma_7=\int_0^1 a(v)\frac{\phi_t^2}{A_t}\Bigg[\left\{\left(-4+2\frac{\phi_s^2}{A_t}\left(\frac{\boldsymbol{u}_{ij}^{\perp\top}\boldsymbol{\Delta}(v)}{\|\boldsymbol{u}_{ij}\|}\right)^2\right)F_1(v)+\frac{1}{4}F_2(v)\right\}n_t^2+$$
$$\frac{\phi_t^2}{A_t}\left\{\left(24-24\frac{\phi_s^2}{A_t}\left(\frac{\boldsymbol{u}_{ij}^{\perp\top}\boldsymbol{\Delta}(v)}{\|\boldsymbol{u}_{ij}\|}\right)^2+4\frac{\phi_s^4}{A_t^2}\left(\frac{\boldsymbol{u}_{ij}^{\perp\top}\boldsymbol{\Delta}(v)}{\|\boldsymbol{u}_{ij}\|}\right)^4\right)F_1(v)+\right.$$
$$\left.\left(6-2\frac{\phi_s^2}{A_t}\right)F_2(v)-6F_3(v)\right\}n_t^2\delta_{i'1}(v)^2\Bigg]\,\boldsymbol{n}_s^\top\boldsymbol{\Delta}(v)\,dv. \quad \text{(S15)}$$

Finally, for the spatial-temporal curvature, we note that

$$\partial_t^2\boldsymbol{\partial}_{\boldsymbol{s}}^2\widetilde{K}=4\sigma^2\frac{\phi_s^2\phi_t^2}{A_t^3}\exp\left(-\frac{\phi_s^2\|\boldsymbol{\Delta}\|^2}{A_t}\right)\Bigg[\left\{\left(2-\frac{\phi_s^2\|\boldsymbol{\Delta}\|^2}{A_t}\right)-2\left(6-6\frac{\phi_s^2\|\boldsymbol{\Delta}\|^2}{A_t}+\frac{\phi_s^4\|\boldsymbol{\Delta}\|^4}{A_t^2}\right)\frac{\phi_t^2\delta^2}{A_t}\right\}\mathbf{I}_2-$$
$$2\frac{\phi_s^2}{A_t}\left\{\left(3-\frac{\phi_s^2\|\boldsymbol{\Delta}\|^2}{A_t}\right)-2\left(12-8\frac{\phi_s^2\|\boldsymbol{\Delta}\|^2}{A_t}+\frac{\phi_s^4\|\boldsymbol{\Delta}\|^4}{A_t^2}\right)\frac{\phi_t^2\delta^2}{A_t}\right\}\boldsymbol{\Delta}\boldsymbol{\Delta}^\top\Bigg].$$

We compute the terms separately using algebra similar to that required for obtaining γ_6. The first term evaluates to

$$\int_0^1 a(v)\frac{\phi_t^2}{A_t}\left[\left\{\left(-4+2\frac{\phi_s^2}{A_t}\left(\frac{\boldsymbol{u}_{ij}^{\perp\mathrm{T}}\boldsymbol{\Delta}(v)}{\|\boldsymbol{u}_{ij}\|}\right)^2\right)F_1(v)+\frac{1}{4}F_2(v)\right\}n_t^2+\right.$$

$$\frac{\phi_t^2}{A_t}\left\{\left(24-24\frac{\phi_s^2}{A_t}\left(\frac{\boldsymbol{u}_{ij}^{\perp\mathrm{T}}\boldsymbol{\Delta}(v)}{\|\boldsymbol{u}_{ij}\|}\right)^2+4\frac{\phi_s^4}{A_t^2}\left(\frac{\boldsymbol{u}_{ij}^{\perp\mathrm{T}}\boldsymbol{\Delta}(v)}{\|\boldsymbol{u}_{ij}\|}\right)^4\right)F_1(v)+\right.$$

$$\left.\left.\left(6-2\frac{\phi_s^2}{A_t}\right)F_2(v)-6F_3(v)\right\}n_t^2\delta_{i'1}(v)^2\right]\|\boldsymbol{n}_s\|^2\,dv, \tag{S16a}$$

while for the second term again following similar algebra produces

$$2\int_0^1 a(v)\,\frac{\phi_t^2\phi_s^2}{A_t^2}\left[\left\{\left(6-2\frac{\phi_s^2}{A_t}\left(\frac{\boldsymbol{u}_{ij}^{\perp\mathrm{T}}\boldsymbol{\Delta}(v)}{\|\boldsymbol{u}_{ij}\|}\right)^2\right)F_1(v)-\frac{1}{4}F_2(v)\right\}n_t^2+\right.$$

$$\frac{\phi_t^2}{A_t}\left\{\left(-48+32\frac{\phi_s^2}{A_t}\left(\frac{\boldsymbol{u}_{ij}^{\perp\mathrm{T}}\boldsymbol{\Delta}(v)}{\|\boldsymbol{u}_{ij}\|}\right)^2-4\frac{\phi_s^4}{A_t^2}\left(\frac{\boldsymbol{u}_{ij}^{\perp\mathrm{T}}\boldsymbol{\Delta}(v)}{\|\boldsymbol{u}_{ij}\|}\right)^4\right)F_1(v)+\right.$$

$$\left.\left.\left(-8+2\frac{\phi_s^4}{A_t^4}\right)F_2(v;\phi_s,\phi_t)+6F_3(v)\right\}n_t^2\delta_{i'1}(v)^2\right]\boldsymbol{n}_s^{\mathrm{T}}\boldsymbol{\Delta}(v)\boldsymbol{\Delta}(v)^{\mathrm{T}}\boldsymbol{n}_s\,dv. \tag{S16b}$$

Adding eqs. (S16a) and (S16b) produces γ_8. Referring to eqs. (S9) to (S15), (S16a) and (S16b), we note that the evaluation of $\gamma(\mathscr{C}_{ij1})$ requires a 1-dimensional quadrature over $[0,1]$.

Triangular plane 2: Here $k=2$. The normal to the plane is given by $\overline{\boldsymbol{n}}_{ij2}=\begin{pmatrix}-\boldsymbol{u}_{ij+1}\\0\end{pmatrix}\times\begin{pmatrix}-\boldsymbol{v}_{i+1j}\\1\end{pmatrix}=\begin{pmatrix}-u_{ij+12}\\u_{ij+11}\\-v_{i+1j1}u_{ij+12}+v_{i+1j2}u_{ij+11}\end{pmatrix}=\begin{pmatrix}\boldsymbol{u}_{ij+1}^{\perp}\\\boldsymbol{v}_{i+1j}^{\mathrm{T}}\boldsymbol{u}_{ij+1}^{\perp}\end{pmatrix}=\begin{pmatrix}\overline{\boldsymbol{n}}_{\boldsymbol{s}}\\\overline{n}_t\end{pmatrix}$, where $\boldsymbol{u}_{ij+1}^{\perp}$ is the normal to $\boldsymbol{u}_{ij+1}$, $\overline{\boldsymbol{n}}_{\boldsymbol{s}}=\boldsymbol{u}_{ij+1}^{\perp}$ and $\overline{n}_t=\boldsymbol{v}_{i+1j}^{\mathrm{T}}\boldsymbol{u}_{ij+1}^{\perp}$. The unit normal is $\boldsymbol{n}_{ij2}=\frac{\overline{\boldsymbol{n}}_{ij2}}{\|\overline{\boldsymbol{n}}_{ij2}\|}$, where $\|\overline{\boldsymbol{n}}_{ij2}\|=\boldsymbol{u}_{ij+1}^{\perp\;\mathrm{T}}\boldsymbol{u}_{ij+1}^{\perp}+\left(\boldsymbol{v}_{i+1j}^{\mathrm{T}}\boldsymbol{u}_{ij+1}^{\perp}\right)^{\mathrm{T}}\left(\boldsymbol{v}_{i+1j}^{\mathrm{T}}\boldsymbol{u}_{ij+1}^{\perp}\right)=\boldsymbol{u}_{ij+1}^{\perp\;\mathrm{T}}\left(\mathbf{I}_2+\boldsymbol{v}_{i+1j}\boldsymbol{v}_{i+1j}^{\mathrm{T}}\right)\boldsymbol{u}_{ij+1}^{\perp}$. The identities required for simplifications are as follows:

$$\boldsymbol{n}_s^{\mathrm{T}}\boldsymbol{\Delta}_{i'2}(\omega,v)=\frac{\boldsymbol{u}_{ij+1}^{\perp\;\mathrm{T}}}{\|n_{ij2}\|}\left(\boldsymbol{\Delta}_{i'i+1j+1}-\omega\boldsymbol{u}_{ij+1}-v v_{ij}\right)=\frac{\boldsymbol{u}_{ij+1}^{\perp\;\mathrm{T}}}{\|n_{ij2}\|}\left(\boldsymbol{\Delta}_{i'i+1j+1}-v v_{ij}\right)$$

$$=\boldsymbol{n}_s^{\mathrm{T}}\left(\boldsymbol{\Delta}_{i'i+1j+1}-v v_{ij}\right)=\boldsymbol{n}_s^{\mathrm{T}}\boldsymbol{\Delta}(v), \tag{S17}$$

$$\|\boldsymbol{\Delta}_{i'2}(\omega,v)\|^2=\boldsymbol{\Delta}_{i'2}(\omega,v)^{\mathrm{T}}\boldsymbol{\Delta}_{i'2}(\omega,v)=\|\boldsymbol{u}_{ij+1}\|^2\left(\omega-\frac{\boldsymbol{u}_{ij+1}^{\mathrm{T}}\boldsymbol{\Delta}(v)}{\|\boldsymbol{u}_{ij+1}\|^2}\right)^2+\boldsymbol{\Delta}(v)^{\mathrm{T}}\left(\mathbf{I}_2-\frac{\boldsymbol{u}_{ij+1}\boldsymbol{u}_{ij+1}^{\mathrm{T}}}{\|\boldsymbol{u}_{ij+1}\|^2}\right)\boldsymbol{\Delta}(v)$$

$$=\|\boldsymbol{u}_{ij+1}\|^2\left(\omega-\frac{\boldsymbol{u}_{ij+1}^{\mathrm{T}}\boldsymbol{\Delta}(v)}{\|\boldsymbol{u}_{ij+1}\|^2}\right)^2+\boldsymbol{\Delta}(v)^{\mathrm{T}}\frac{\boldsymbol{u}_{ij+1}^{\perp}\boldsymbol{u}_{ij+1}^{\perp\;\mathrm{T}}}{\|\boldsymbol{u}_{ij+1}\|^2}\boldsymbol{\Delta}(v)$$

$$= \|\boldsymbol{u}_{ij+1}\|^2 \left(\omega - \frac{\boldsymbol{u}_{ij+1}^{\mathrm{T}}\boldsymbol{\Delta}(v)}{\|\boldsymbol{u}_{ij+1}\|^2}\right)^2 + \left(\frac{\boldsymbol{u}_{ij+1}^{\perp}{}^{\mathrm{T}}\boldsymbol{\Delta}(v)}{\|\boldsymbol{u}_{ij+1}\|}\right)^2, \tag{S18}$$

$$\|\boldsymbol{\Delta}_{i'2}(\omega, v)\|^4 = \left\{\|\boldsymbol{u}_{ij+1}\|^2 \left(\omega - \frac{\boldsymbol{u}_{ij+1}^{\mathrm{T}}\boldsymbol{\Delta}(v)}{\|\boldsymbol{u}_{ij+1}\|^2}\right)^2 + \boldsymbol{\Delta}(v)^{\mathrm{T}}\left(\mathbf{I}_2 - \frac{\boldsymbol{u}_{ij+1}\boldsymbol{u}_{ij+1}^{\mathrm{T}}}{\|\boldsymbol{u}_{ij+1}\|^2}\right)\boldsymbol{\Delta}(v)\right\}^2$$

$$= \|\boldsymbol{u}_{ij+1}\|^4 \left(\omega - \frac{\boldsymbol{u}_{ij+1}^{\mathrm{T}}\boldsymbol{\Delta}(v)}{\|\boldsymbol{u}_{ij+1}\|^2}\right)^4 + 2\left(\omega - \frac{\boldsymbol{u}_{ij+1}^{\mathrm{T}}\boldsymbol{\Delta}(v)}{\|\boldsymbol{u}_{ij+1}\|^2}\right)^2 \boldsymbol{\Delta}(v)^{\mathrm{T}}\boldsymbol{u}_{ij+1}^{\perp}\boldsymbol{u}_{ij+1}^{\perp}{}^{\mathrm{T}}\boldsymbol{\Delta}(v) + \left(\boldsymbol{\Delta}(v)^{\mathrm{T}}\frac{\boldsymbol{u}_{ij+1}^{\perp}\boldsymbol{u}_{ij+1}^{\perp}{}^{\mathrm{T}}}{\|\boldsymbol{u}_{ij+1}\|^2}\boldsymbol{\Delta}(v)\right)^2$$

$$= \|\boldsymbol{u}_{ij+1}\|^4 \left(\omega - \frac{\boldsymbol{u}_{ij+1}^{\mathrm{T}}\boldsymbol{\Delta}(v)}{\|\boldsymbol{u}_{ij+1}\|^2}\right)^4 + 2\left(\omega - \frac{\boldsymbol{u}_{ij+1}^{\mathrm{T}}\boldsymbol{\Delta}(v)}{\|\boldsymbol{u}_{ij+1}\|^2}\right)^2 \left(\boldsymbol{u}_{ij+1}^{\perp}{}^{\mathrm{T}}\boldsymbol{\Delta}(v)\right)^2 + \left(\frac{\boldsymbol{u}_{ij+1}^{\perp}{}^{\mathrm{T}}\boldsymbol{\Delta}(v)}{\|\boldsymbol{u}_{ij+1}\|}\right)^4. \tag{S19}$$

The remainder of the calculation follows triangular plane 1 using eqs. (S17) to (S19). Overall, the evaluation of $\gamma(\mathscr{C}_{ijk})$ requires 1-dimensional quadrature. □

Finally, we conclude by noting that simplifications for the terms in $\mathbf{K}_{\boldsymbol{\Gamma}}(\mathscr{C}, \mathscr{C})$ follow similar algebraic steps and make use of Lemma 3. They can be expressed using cdfs for χ^2-distributions with higher degrees of freedom. The next discussion outlines algorithms used for triangulating surfaces, posterior sampling of gradients and wombling measures.

S11 Algorithms

We outline algorithms for obtaining posterior samples of the spatiotemporal differential processes and the wombling measures. We use a grid G over which the differential processes are sampled. The choice of G can be regularly spaced locations spanning the spatial domain at every time point or space-time locations between time points, or extrapolated space-time locations. These algorithms are implemented in the R-statistical environment (R Core Team 2021) and available for download at https://github.com/arh926/sptwombling.

S11.1 Spatiotemporal Gradients

Algorithm 1: Posterior sampling of spatiotemporal differential processes

Input: (i) Observed process $(\boldsymbol{s}_i, t_i)$, $i = 1, \ldots, N$;
(ii) Posterior samples $\boldsymbol{\theta}_1, \ldots, \boldsymbol{\theta}_{n_{mcmc}}$ from fitting model in eq. (11);
(iii) A kernel, $K(\cdot, \boldsymbol{\theta})$ of choice (Matérn with $\nu = 3/2, 5/2$ or ∞);
(iv) A grid $G = \{(\boldsymbol{s}_{i_g}, t_{i_g}), i_g = 1, \ldots, n_G\}$;
Output: Posterior samples of spatiotemporal differential processes over G;

```
for i = 1,...,N do
    for i_g = 1,...,n_G do
        /* Compute Δ and δ for observed locations, (s_i, t_i) and grid G   */
        (Δ[i,i_g], δ[i,i_g]) = (s_{i_g} − s_i, t_{i_g} − t_i);
for i_θ = 1,...,n_mcmc do
    K = K(·;θ_{i_θ}), K^{-1} = (K(·;θ_{i_θ}))^{-1} ;       // O(N^3) step--compute once
    M = K^{-1} + τ^{-2} I_N, m = τ^{-2} M^{-1} 𝒴, 𝒵 ~ N(m, M^{-1}); // Posterior sample of Z
    V_0 = V_{ℒ*}(0_2, 0; θ_{i_θ});                          // see Section S12
    for i_g = 1,...,n_G do
        𝒦_0 = ℒ* K(Δ[, i_g], δ[, i_g]; θ_{i_θ});
        /* Posterior mean and cross-covariance matrix                        */
        μ_{ℒ*} = −𝒦_0^T K^{-1} 𝒵, Σ_{ℒ*} = V_0 − 𝒦_0^T K^{-1} 𝒦_0;
        ℒ* Z[i_θ, i_g] ~ 𝒩_18(μ_{ℒ*}, Σ_{ℒ*})
return ℒ* Z
```

S11.2 Spatiotemporal Wombling Measures

We focus on obtaining posterior samples for $\Gamma(\mathscr{C})$. It relies on Algorithm 2, which triangulates $\mathscr{C}$. Alternatively, posterior samples of the spatiotemporal differential processes can be used to generate posterior samples of $\Gamma(\mathscr{C})$. Algorithm 3 utilizes posterior samples of $\mathcal{L}^* Z$ and the Riemann sum approximation from Theorem 2 to generate posterior samples of $\Gamma(\mathscr{C})$ and the induced GP over parametric triangular planes. Finally, Algorithm 4 uses the posterior in eq. (10). If derivative estimation and wombling are performed simultaneously, Algorithms 1 and 4 are combined to avoid repeated inversion of $K(\cdot, \cdot; \boldsymbol{\theta})$.

Algorithm 2: Triangulation of a wombling surface

Input: Wombling surface $\mathscr{C}$ and partitioned set of planar curves $\widetilde{C}_{t_j}$, $j = 1, \ldots, N$ (see Section 4.2) on $\mathscr{C}$ with the length of partition (n_ω, n_v);
Output: Set of points constituting parametric triangles with normals
for $i = 1, \ldots, n_\omega - 1$ **do**
  for $j = 1, \ldots, n_v - 1$ **do**
    $T = \{(\omega, v) : 0 \le \omega + v < 1\}$; `// Triangle in` $\Re^2$
    `/* Parametric Plane 1 */`
    $\mathscr{C}_{ij1} = \left\{ \begin{pmatrix} \boldsymbol{s}(\omega_i', t_j) \\ t_j \end{pmatrix} + \omega \begin{pmatrix} \boldsymbol{u}_{ij} \\ 0 \end{pmatrix} + v \begin{pmatrix} \boldsymbol{v}_{ij+1} \\ t_{j+1} - t_j \end{pmatrix} : (\omega, v) \in T \right\}$;
    `/* Parametric Plane 2 */`
    $\mathscr{C}_{ij2} = \left\{ \begin{pmatrix} \boldsymbol{s}(\omega_{i+1}', t_{j+1}) \\ t_{j+1} \end{pmatrix} + \omega \begin{pmatrix} \boldsymbol{u}_{ij+1} \\ 0 \end{pmatrix} + v \begin{pmatrix} \boldsymbol{v}_{i+1j} \\ t_j - t_{j+1} \end{pmatrix} : (\omega, v) \in T \right\}$;
    $\overline{\boldsymbol{n}}_{ij1} = \begin{pmatrix} \boldsymbol{v}_{ij+1} \\ t_{j+1} - t_j \end{pmatrix} \times \begin{pmatrix} \boldsymbol{u}_{ij} \\ 0 \end{pmatrix}$; `// Normal to Plane 1`
    $\overline{\boldsymbol{n}}_{ij2} = \begin{pmatrix} -\boldsymbol{u}_{ij+1} \\ 0 \end{pmatrix} \times \begin{pmatrix} -\boldsymbol{v}_{i+1j} \\ t_{j+1} - t_j \end{pmatrix}$; `// Normal to Plane 2`

return $\widetilde{\mathscr{C}} = \bigcup_{i=1}^{n_\omega - 1} \bigcup_{j=1}^{n_v - 1} \bigcup_{k=1}^{2} \mathscr{C}_{ijk}$ and $\left\{ \overline{\boldsymbol{n}}_{ijk} : i = 1, \ldots, n_\omega - 1, j = 1, \ldots, n_v - 1, k = 1, 2 \right\}$

Algorithm 3: Posterior samples of wombling measures using Riemann sums

Input: Triangulated Surface $\widetilde{\mathscr{C}}$ and Normals (using Algorithm 2);
Output: Posterior samples of $\Gamma(\widetilde{\mathscr{C}})$;
Step 1: Use Algorithm 1 to obtain posterior samples of $\mathcal{L}^* Z$ for $\widetilde{\mathscr{C}}$;
for $i_{\boldsymbol{\theta}} = 1, \ldots, n_{mcmc}$ **do**
  `/* Riemann sum approximation */`
  $\Gamma(\widetilde{\mathscr{C}})[i_{\boldsymbol{\theta}}] = \sum_{i=1}^{n_\omega - 1} \sum_{j=1}^{n_v - 1} \sum_{k=1}^{2} (v_{j+1}' - v_j') \, (\omega_{i+1}' - \omega_i') \, g\left(\omega_i^*, v_j^*\right) \, \|\overline{\boldsymbol{n}}_{ijk}(\omega_i^*, v_j^*)\|$;

return $\Gamma(\widetilde{\mathscr{C}})$

In Algorithm 3, gs are linear functions in $\mathcal{L}^*_{n_t,\boldsymbol{n}_s}$—spatiotemporal directional derivatives and curvatures and ω^*s and v^*s are mid-points of rectangles (see Theorem 2 p. 27).

Algorithm 4: Posterior samples of wombling measures using eq. (10)

Input: (i) Observed process $(\boldsymbol{s}_i, t_i)$, $i = 1, \ldots, N$;
(ii) Posterior samples $\boldsymbol{\theta}_1, \ldots, \boldsymbol{\theta}_{n_{mcmc}}$ from fitting model in eq. (11);
(iii) A kernel, $K(\cdot, \boldsymbol{\theta})$ of choice (Matérn with $\nu = 3/2, 5/2$ or ∞);
(iv) Triangulated Surface $\widetilde{\mathscr{C}}$ and Normals (using Algorithm 2);
Output: Posterior samples of $\boldsymbol{\Gamma}(\widetilde{\mathscr{C}})$;
for $i_{\boldsymbol{\theta}} = 1, \ldots, n_{mcmc}$ **do**

$\mathbf{K} = \mathbf{K}(\cdot; \boldsymbol{\theta}_{i_{\boldsymbol{\theta}}})$, $\mathbf{K}^{-1} = (\mathbf{K}(\cdot; \boldsymbol{\theta}_{i_{\boldsymbol{\theta}}}))^{-1}$; // $O(N^3)$ step--compute once

$\mathbf{M} = \mathbf{K}^{-1} + \tau^{-2}\mathbf{I}_N$, $\boldsymbol{m} = \tau^{-2}\mathbf{M}^{-1}\boldsymbol{\mathcal{Y}}$, $\boldsymbol{\mathcal{Z}} \sim N(\boldsymbol{m}, \mathbf{M}^{-1})$; // Posterior sample of Z

/* computed using 2-D quadrature */

$\mathbf{G}(\widetilde{\mathscr{C}}) = \iint_{\widetilde{\mathscr{C}}} \mathbf{N}_{st}\boldsymbol{\mathcal{K}}_0(\boldsymbol{\Delta}(\omega, v), \delta(v))\|\overline{\boldsymbol{n}}\|\, d\omega\, dv$;

/* computed using Lemma 3 and 2-D quadrature */

$\mathbf{K}_{\boldsymbol{\Gamma}}(\widetilde{\mathscr{C}}, \widetilde{\mathscr{C}}) = \|\overline{\boldsymbol{n}}\|^2 \int \cdots \int_{\widetilde{\mathscr{C}} \times \widetilde{\mathscr{C}}} \mathbf{N}_{st}\mathbf{V}_{\mathcal{L}^*Z}(\boldsymbol{\Delta}(\omega_1, \omega_2, v_1, v_2), \delta(v_1, v_2))\, \mathbf{N}_{st}^{\mathrm{T}}\, d\omega_1\, d\omega_2\, dv_1\, dv_2$;

/* Posterior mean and cross-covariance matrix */

$\boldsymbol{\mu}_{\boldsymbol{\Gamma}} = -\mathbf{G}(\widetilde{\mathscr{C}})^{\mathrm{T}}\mathbf{K}^{-1}\boldsymbol{\mathcal{Z}}$, $\Sigma_{\boldsymbol{\Gamma}} = \mathbf{K}_{\boldsymbol{\Gamma}}(\widetilde{\mathscr{C}}, \widetilde{\mathscr{C}}) - \mathbf{G}(\widetilde{\mathscr{C}})^{\mathrm{T}}\mathbf{K}^{-1}\mathbf{G}(\widetilde{\mathscr{C}})$;

$\boldsymbol{\Gamma}(\widetilde{\mathscr{C}})[i_{\boldsymbol{\theta}}] \sim \mathcal{N}_8(\boldsymbol{\mu}_{\boldsymbol{\Gamma}}, \Sigma_{\boldsymbol{\Gamma}})$

return $\boldsymbol{\Gamma}(\widetilde{\mathscr{C}})$

S12 Spatiotemporal Covariance Functions

S12.1 Separable:

The required derivatives for the separable space-time kernels are computed using $\partial_t^j \boldsymbol{\partial}_{\boldsymbol{s}}^{r-j} K(\boldsymbol{\Delta}, \delta) = \partial_t^j \boldsymbol{\partial}_{\boldsymbol{s}}^{r-j} \{K_t(\delta) \otimes K_s(\boldsymbol{\Delta})\} = \partial_t^j K_t(\delta) \otimes \boldsymbol{\partial}_{\boldsymbol{s}}^{r-j} K_s(\boldsymbol{\Delta})$. The derivatives are listed using x which can either be δ or, $\boldsymbol{\Delta}$. In case $x = \delta$, $\|x\| = |\delta|$, then $\phi = \phi_t$ and $\partial = \partial_t$, otherwise, $\partial = \boldsymbol{\partial}_{\boldsymbol{s}}$, $\|x\| = \|\boldsymbol{\Delta}\|$, and $\phi = \phi_s$, where $\|\cdot\|$ stands for the Euclidean norm. For a Matérn with $\nu = 3/2$ we have $\partial\widetilde{K}(x) = -3\sigma^2\phi^2 e^{-\sqrt{3}\phi\|x\|}x$ and $\partial^2\widetilde{K}(x) = -3\sigma^2\phi^2 e^{-\sqrt{3}\phi\|x\|}\left(1 - \sqrt{3}\phi\frac{xx^{\mathrm{T}}}{\|x\|}\right)$.

S12.1.1 Matérn Covariance ($\nu = 5/2$)

We have the first derivative, $\partial\widetilde{K}(x) = -\frac{5}{3}\sigma^2\phi^2 e^{-\sqrt{5}\phi\|x\|}\left(1+\sqrt{5}\phi\|x\|\right)x$ and the second, $\partial^2\widetilde{K}(x) = -\frac{5}{3}\sigma^2\phi^2 e^{-\sqrt{5}\phi\|x\|}\left(1+\sqrt{5}\phi\|x\| - 5\phi^2 xx^{\mathrm{T}}\right)$. The third and fourth derivatives are

$$\partial^3\widetilde{K}(x) = \begin{cases} \frac{25}{3}\sigma^2\phi^4 e^{-\sqrt{5}\phi\|x\|}\left(3-\sqrt{5}\phi\frac{x_i^2}{\|x\|}\right)x_i & iii \\ \frac{25}{3}\sigma^2\phi^4 e^{-\sqrt{5}\phi\|x\|}\left(1-\sqrt{5}\phi\frac{x_i^2}{\|x\|}\right)x_j & iij \\ \frac{25\sqrt{5}}{3}\sigma^2\phi^5 e^{-\sqrt{5}\phi\|x\|}\frac{x_i x_j x_k}{\|x\|} & ijk \end{cases}$$

$$\partial^4\widetilde{K}(x) = \begin{cases} \frac{25}{3}\sigma^2\phi^4 e^{-\sqrt{5}\phi\|x\|}\left\{3-6\sqrt{5}\phi\frac{x_i^2}{\|x\|}+\sqrt{5}\phi\left(\sqrt{5}\phi+\frac{1}{\|x\|}\right)\frac{x_i^4}{\|x\|^2}\right\} & iiii \\ -\frac{25\sqrt{5}}{3}\sigma^2\phi^5 e^{-\sqrt{5}\phi\|x\|}\left\{3-\sqrt{5}\phi\frac{x_i^2}{\|x\|}-\frac{x_i}{\|x\|^2}\right\}\frac{x_i x_j}{\|x\|} & iiij \\ \frac{25}{3}\sigma^2\phi^4 e^{-\sqrt{5}\phi\|x\|}\left\{\left(1-\sqrt{5}\phi\frac{x_i^2}{\|x\|}\right)\left(1-\sqrt{5}\phi\frac{x_j^2}{\|x\|}\right)+\sqrt{5}\phi\frac{x_i^2 x_j^2}{\|x\|^3}\right\} & iijj \\ -\frac{25\sqrt{5}}{3}\sigma^2\phi^5 e^{-\sqrt{5}\phi\|x\|}\left\{1-\frac{x_i}{\|x\|}\left(\sqrt{5}\phi+\frac{1}{\|x\|}\right)\right\}\frac{x_j x_k}{\|x\|} & iijk \\ \frac{25\sqrt{5}}{3}\sigma^2\phi^5 e^{-\sqrt{5}\phi\|x\|}\left(\sqrt{5}\phi+\frac{1}{\|x\|}\right)\frac{x_i x_j x_k x_l}{\|x\|^2} & ijkl \end{cases}$$

S12.1.2 Gaussian

We have $\partial\widetilde{K}(x) = -2\sigma^2\phi^2 e^{-\phi^2\|x\|^2}x$ and $\partial^2\widetilde{K}(x) = -2\sigma^2\phi^2 e^{-\phi^2\|x\|^2}(1-2\phi^2 xx^{\mathrm{T}})$. Next,

$$\partial^3\widetilde{K}(x) = \begin{cases} 4\sigma^2\phi^4 e^{-\phi^2\|x\|^2}(3-2\phi^2 x_i^2)x_i, & iii \\ 4\sigma^2\phi^4 e^{-\phi^2\|x\|^2}(1-2\phi^2 x_i^2)x_j, & iij \\ -8\sigma^2\phi^6 e^{-\phi^2\|x\|^2}x_i x_j x_k, & ijk \end{cases}, \partial^4\widetilde{K}(x) = \begin{cases} 4\sigma^2\phi^4 e^{-\phi^2\|x\|^2}(3-12\phi^2 x_i^2+4\phi^4 x_i^4), & iiii \\ -8\sigma^2\phi^6 e^{-\phi^2\|x\|^2}(3-2\phi^2 x_i^2)x_i x_j, & iiij \\ 4\sigma^2\phi^4 e^{-\phi^2\|x\|^2}(1-2\phi^2 x_i^2)(1-2\phi^2 x_j^2), & iijj \\ 8\sigma^2\phi^6 e^{-\phi^2\|x\|^2}(1-2\phi^2 x_i^2)x_j x_k, & iijk \\ 16\sigma^2\phi^8 e^{-\phi^2\|x\|^2}x_i x_j x_k x_l, & ijkl \end{cases}$$

S12.1.3 Inverse

Particularly, for comparing results with non-separable kernels, presented in the next discussion, we let $\widetilde{K}_t(|\delta|) = \sigma^2(\phi_t^2|\delta|^2 + 1)^{-1} = \sigma^2 A_t^{-1}$. Note that $\frac{\partial}{\partial\delta}A_t = 2\phi_t^2\delta$. The required derivatives are, $\partial_t\widetilde{K}(\delta) = -\frac{2\phi_t^2\delta}{A_t^2}$, $\partial_t^2\widetilde{K}(\delta) = -\frac{2\phi_t^2}{A_t^3}(1 - 3\phi_t^2\delta^2)$, $\partial_t^3\widetilde{K}(\delta) = \frac{24\phi_t^4\delta}{A_t^4}(1 - \phi_t^2\delta^2)$ and $\partial_t^4\widetilde{K}(\delta) = \frac{24\phi_t^4}{A_t^5}(1 - 12\phi_t^2\delta^2 + 5\phi_t^4\delta^4)$.

For comparison with the non-separable case, the resulting cross-covariance using Matérn with $\nu = 3/2$, $\nu = 5/2$ and the Gaussian case for the spatial covariance and the inverse for the temporal covariance is shown below. For a Matérn with $\nu = 3/2$, $\mathcal{L}^*Z(\boldsymbol{s}_0, t_0) = (Z(s_\mathcal{S}, t_\mathcal{T}), \boldsymbol{\partial}_{\boldsymbol{s}}Z(\boldsymbol{s}_0, t_0)^{\mathrm{T}}, \partial_t Z(\boldsymbol{s}_0, t_0), \partial_t\boldsymbol{\partial}_{\boldsymbol{s}}Z(\boldsymbol{s}_0, t_0)^{\mathrm{T}})^{\mathrm{T}}$, at an arbitrary space-time location, $(\boldsymbol{s}_0, t_0)$

$$\mathbf{V}_{\mathcal{L}^*} = \sigma^2 \begin{pmatrix} \Sigma_Z & \boldsymbol{\partial}_{\boldsymbol{s}}\mathbf{K}_0^{\mathrm{T}} & \partial_t\mathbf{K}_0 & \partial_t\boldsymbol{\partial}_{\boldsymbol{s}}\mathbf{K}_0^{\mathrm{T}} \\ -\boldsymbol{\partial}_{\boldsymbol{s}}\mathbf{K}_0 & 3\phi_s^2\mathbf{I}_d & 0 & 0 \\ -\partial_t\mathbf{K}_0 & 0 & 2\phi_t^2 & 0 \\ \partial_t\boldsymbol{\partial}_{\boldsymbol{s}}\mathbf{K}_0 & 0 & 0 & 6\phi_s^2\phi_t^2\mathbf{I}_d \end{pmatrix}, \tag{S20}$$

In case of a Matérn with $\nu = 5/2$ and Gaussian, the full cross-covariance matrix is obtained as $(\|\boldsymbol{\Delta}\|, |\delta|) \to 0$, excepting the terms $\boldsymbol{\partial}_{\boldsymbol{s}}K, \partial_t K, \widetilde{\boldsymbol{\partial}_{\boldsymbol{s}}}^2 K, \partial_t\boldsymbol{\partial}_{\boldsymbol{s}}K, \partial_t^2 K, \partial_t\widetilde{\boldsymbol{\partial}_{\boldsymbol{s}}}^2 K, \partial_t^2\boldsymbol{\partial}_{\boldsymbol{s}}K$ and $\partial_t^2\widetilde{\boldsymbol{\partial}_{\boldsymbol{s}}}^2 K$. The resulting cross-covariance matrices are

$$\mathbf{V}_{\mathcal{L}^*} = \sigma^2 \left[\begin{array}{c:cc:ccc:ccc} \Sigma_Z & \boldsymbol{\partial}_{\boldsymbol{s}}\mathbf{K}_0^{\mathrm{T}} & \widetilde{\boldsymbol{\partial}_{\boldsymbol{s}}}^2\mathbf{K}_0^{\mathrm{T}} & \partial_t\mathbf{K}_0 & \partial_t\boldsymbol{\partial}_{\boldsymbol{s}}\mathbf{K}_0^{\mathrm{T}} & \partial_t\widetilde{\boldsymbol{\partial}_{\boldsymbol{s}}}^2\mathbf{K}_0^{\mathrm{T}} & \partial_t^2\mathbf{K}_0 & \partial_t^2\boldsymbol{\partial}_{\boldsymbol{s}}\mathbf{K}_0^{\mathrm{T}} & \partial_t^2\widetilde{\boldsymbol{\partial}_{\boldsymbol{s}}}^2\mathbf{K}_0^{\mathrm{T}} \\ \hdashline -\boldsymbol{\partial}_{\boldsymbol{s}}\mathbf{K}_0 & \frac{5}{3}\phi_s^2 I_d & 0 & 0 & 0 & 0 & 0 & -\frac{10}{3}\phi_s^2\phi_t^2\mathbf{I}_d & 0 \\ \widetilde{\boldsymbol{\partial}_{\boldsymbol{s}}}^2\mathbf{K}_0 & 0 & \frac{25}{3}\phi_s^4\mathbf{A} & 0 & 0 & 0 & \frac{10}{3}\phi_s^2\phi_t^2\tilde{I}_d & 0 & -\frac{50}{3}\phi_s^4\phi_t^2\mathbf{A} \\ \hdashline -\partial_t\mathbf{K}_0 & 0 & 0 & 2\phi_t^2 & 0 & -\frac{10}{3}\phi_s^2\phi_t^2\tilde{I}_d^{\mathrm{T}} & 0 & 0 & 0 \\ \partial_t\boldsymbol{\partial}_{\boldsymbol{s}}\mathbf{K}_0 & 0 & 0 & 0 & \frac{10}{3}\phi_s^2\phi_t^2 I_d & 0 & 0 & 0 & 0 \\ -\partial_t\widetilde{\boldsymbol{\partial}_{\boldsymbol{s}}}^2\mathbf{K}_0 & 0 & 0 & -\frac{10}{3}\phi_s^2\phi_t^2\tilde{I}_d & 0 & \frac{50}{3}\phi_s^4\phi_t^2\mathbf{A} & 0 & 0 & 0 \\ \hdashline \partial_t^2\mathbf{K}_0 & 0 & \frac{10}{3}\phi_s^2\phi_t^2\tilde{I}_d^{\mathrm{T}} & 0 & 0 & 0 & 24\phi_t^4 & 0 & -40\phi_s^2\phi_t^4\tilde{I}_d^{\mathrm{T}} \\ -\partial_t^2\boldsymbol{\partial}_{\boldsymbol{s}}\mathbf{K}_0 & -\frac{10}{3}\phi_s^2\phi_t^2\mathbf{I}_d & 0 & 0 & 0 & 0 & 0 & 40\phi_s^2\phi_t^4\mathbf{I}_d & 0 \\ \partial_t^2\widetilde{\boldsymbol{\partial}_{\boldsymbol{s}}}^2\mathbf{K}_0 & 0 & -\frac{50}{3}\phi_s^4\phi_t^2\mathbf{A} & 0 & 0 & 0 & -40\phi_s^2\phi_t^4\tilde{I}_d & 0 & 200\phi_s^4\phi_t^4\mathbf{A} \end{array}\right],$$

and,

$$
\mathbf{V}_{\mathcal{L}^*} = \sigma^2 \left(\begin{array}{ccc:ccc:ccc}
\Sigma_Z & \boldsymbol{\partial}_{\boldsymbol{s}}\mathbf{K}_0^{\mathrm{T}} & \widetilde{\boldsymbol{\partial}_{\boldsymbol{s}}}^2\mathbf{K}_0^{\mathrm{T}} & \partial_t\mathbf{K}_0 & \partial_t\boldsymbol{\partial}_{\boldsymbol{s}}\mathbf{K}_0^{\mathrm{T}} & \partial_t\widetilde{\boldsymbol{\partial}_{\boldsymbol{s}}}^2\mathbf{K}_0^{\mathrm{T}} & \partial_t^2\mathbf{K}_0 & \partial_t^2\boldsymbol{\partial}_{\boldsymbol{s}}\mathbf{K}_0^{\mathrm{T}} & \partial_t^2\widetilde{\boldsymbol{\partial}_{\boldsymbol{s}}}^2\mathbf{K}_0^{\mathrm{T}} \\
-\boldsymbol{\partial}_{\boldsymbol{s}}\mathbf{K}_0 & 2\phi_s^2\mathbf{I}_d & 0 & 0 & 0 & 0 & 0 & -4\phi_s^2\phi_t^2\mathbf{I}_d & 0 \\
\widetilde{\boldsymbol{\partial}_{\boldsymbol{s}}}^2\mathbf{K}_0 & 0 & 4\phi_s^4\mathbf{A} & 0 & 0 & 0 & 4\phi_s^2\phi_t^2\tilde{I}_d & 0 & -8\phi_s^4\phi_t^2\mathbf{A} \\
\hdashline
-\partial_t\mathbf{K}_0 & 0 & 0 & 2\phi_t^2 & 0 & -4\phi_s^2\phi_t^2\tilde{I}_d^{\mathrm{T}} & 0 & 0 & 0 \\
\partial_t\boldsymbol{\partial}_{\boldsymbol{s}}\mathbf{K}_0 & 0 & 0 & 0 & 4\phi_s^2\phi_t^2\mathbf{I}_d & 0 & 0 & 0 & 0 \\
-\partial_t\widetilde{\boldsymbol{\partial}_{\boldsymbol{s}}}^2\mathbf{K}_0 & 0 & 0 & -4\phi_s^2\phi_t^2\tilde{I}_d & 0 & 8\phi_s^4\phi_t^2\mathbf{A} & 0 & 0 & 0 \\
\hdashline
\partial_t^2\mathbf{K}_0 & 0 & 4\phi_s^2\phi_t^2\tilde{I}_d^{\mathrm{T}} & 0 & 0 & 0 & 24\phi_t^4 & 0 & -48\phi_s^2\phi_t^4\tilde{I}_d^{\mathrm{T}} \\
-\partial_t^2\boldsymbol{\partial}_{\boldsymbol{s}}\mathbf{K}_0 & -4\phi_s^2\phi_t^2\mathbf{I}_d & 0 & 0 & 0 & 0 & 0 & 48\phi_s^2\phi_t^4\mathbf{I}_d & 0 \\
\partial_t^2\widetilde{\boldsymbol{\partial}_{\boldsymbol{s}}}^2\mathbf{K}_0 & 0 & -8\phi_s^4\phi_t^2\mathbf{A} & 0 & 0 & 0 & -48\phi_s^2\phi_t^4\tilde{I}_d & 0 & 96\phi_s^4\phi_t^4\mathbf{A}
\end{array}\right),
$$

respectively, where $\mathbf{A} = \mathbf{A}_{d(d+1)/2} = \begin{pmatrix} 3\ 0\ 0 & \cdots & 1\ 0 & \cdots & 1\ 0\ 1 \\ 0\ 1\ 0 & \cdots & 0\ 0 & \cdots & 0\ 0\ 0 \\ \vdots\ \vdots\ \vdots & \ddots & \vdots\ \vdots & \ddots & \vdots\ \vdots\ \vdots \\ 1\ 0\ 0 & \cdots & 3\ 0 & \cdots & 1\ 0\ 1 \\ \vdots\ \vdots\ \vdots & \ddots & \vdots\ \vdots & \ddots & \vdots\ \vdots\ \vdots \\ 1\ 0\ 0 & \cdots & 1\ 0 & \cdots & 1\ 0\ 3 \end{pmatrix}$. For $d = 2$, $\mathbf{A} = \mathbf{A}_3 = \begin{pmatrix} 3 & 0 & 1 \\ 0 & 1 & 0 \\ 1 & 0 & 3 \end{pmatrix}$. Observe that the *variance for spatiotemporal derivatives is lower* compared to the cross-covariance matrices for the non-separable kernels (for, e.g., compare eqs. (S20) and (S21)).

S12.2 Non-separable

We provide examples of non-separable stationary space-time covariance. We focus on the Matérn family of covariance functions (see, for e.g, Gneiting 2002, Quick et al. 2015). Let $K = \widetilde{K}(\|\boldsymbol{\Delta}\|, |\delta|) = \widetilde{K}(\|\boldsymbol{\Delta}\|, |\delta|; \boldsymbol{\theta})$, where $\boldsymbol{\theta} = \{\phi_s, \phi_t, \sigma^2\}$ denote the covariance kernel. The Matérn family has an additional fractal parameter, ν determining smoothness of process realizations. We consider three kernels, $\nu = 3/2, 5/2$ and ∞ (Gaussian or, squared exponential kernel). Let $A_t = \phi_t^2|\delta|^2 + 1$, then $\dfrac{\partial A_t}{\partial \delta} = 2\phi_t^2\delta$. Substituting $\delta = 0$ in the expressions derived below produces spatial derivatives (see Section S12.1).

S12.2.1 Matérn Covariance ($\nu = 3/2$)

The covariance kernel is

$$
\widetilde{K}(\|\boldsymbol{\Delta}\|, |\delta|) = \widetilde{K} = \frac{\sigma^2}{A_t}\left(1 + \sqrt{3}\frac{\phi_s\|\boldsymbol{\Delta}\|}{A_t^{1/2}}\right)\exp\left(-\sqrt{3}\frac{\phi_s\|\boldsymbol{\Delta}\|}{A_t^{1/2}}\right).
$$

It admits two derivatives in space-time respectively, i.e. $\partial_t^2\boldsymbol{\partial}_{\boldsymbol{s}}^2\widetilde{K} < \infty$. Let $c(\boldsymbol{\theta}) = c(\|\boldsymbol{\Delta}\|,|\delta|;\boldsymbol{\theta}) = \frac{\sigma^2}{A_t^2}e^{-\sqrt{3}\frac{\phi_s\|\boldsymbol{\Delta}\|}{A_t^{1/2}}}$. Partial derivatives with respect to δ and $\boldsymbol{\Delta}$ are, $\frac{\partial}{\partial\delta}c(\boldsymbol{\theta}) = \frac{c(\boldsymbol{\theta})}{A_t}\left(\sqrt{3}\frac{\phi_s\|\boldsymbol{\Delta}\|}{A_t^{1/2}} - 4\right)\phi_t^2\delta$, and $\frac{\partial}{\partial\boldsymbol{\Delta}}c(\boldsymbol{\theta}) = -c(\boldsymbol{\theta})\left(\sqrt{3}\frac{\phi_s}{A_t^{1/2}}\frac{\boldsymbol{\Delta}}{\|\boldsymbol{\Delta}\|}\right)$. We compute the required derivatives. $\boldsymbol{\partial}_{\boldsymbol{s}}\widetilde{K} = -3\phi_s^2c(\boldsymbol{\theta})\boldsymbol{\Delta}$, $\partial_t\widetilde{K} = -2\phi_t^2c(\boldsymbol{\theta})\ f_{10}\ \delta$, where $f_{10} = 1 + \sqrt{3}\frac{\phi_s\|\boldsymbol{\Delta}\|}{A_t^{1/2}} - \frac{3}{2}\frac{\phi_s^2\|\boldsymbol{\Delta}\|^2}{A_t}$, $\boldsymbol{\partial}_{\boldsymbol{s}}^2\widetilde{K} = -3\phi_s^2c(\boldsymbol{\theta})\left(\mathbf{I}_d - \sqrt{3}\frac{\phi_s}{A_t^{1/2}}\frac{\boldsymbol{\Delta}\boldsymbol{\Delta}^{\mathrm{T}}}{\|\boldsymbol{\Delta}\|}\right)$, $\partial_t\boldsymbol{\partial}_{\boldsymbol{s}}\widetilde{K} = 6\phi_s^2\phi_t^2\frac{c(\boldsymbol{\theta})}{A_t}\ f_{11}\ \boldsymbol{\Delta}\delta$, where $f_{11} = 2 - \frac{\sqrt{3}}{2}\frac{\phi_s\|\boldsymbol{\Delta}\|}{A_t^{1/2}}$, $\partial_t^2\widetilde{K} = -2\phi_t^2c(\boldsymbol{\theta})\left\{f_{10} - f_{20}\frac{\phi_t^2\delta^2}{A_t}\right\}$, where $f_{20} = -\left\{\left(\sqrt{3}\frac{\phi_s\|\boldsymbol{\Delta}\|}{A_t^{1/2}} - 4\right)f_{10} + \frac{A_t}{\phi_t^2\delta}\frac{\partial}{\partial\delta}f_{10}\right\}$, $\partial_t\boldsymbol{\partial}_{\boldsymbol{s}}^2\widetilde{K} = 3\phi_s^2\phi_t^2\frac{c(\boldsymbol{\theta})}{A_t}\ f_{12}\ \delta$, where $f_{12} = 4\mathbf{I}_d - 5\sqrt{3}\frac{\phi_s}{A_t^{1/2}}\frac{\boldsymbol{\Delta}\boldsymbol{\Delta}^{\mathrm{T}}}{\|\boldsymbol{\Delta}\|} - \sqrt{3}\frac{\phi_s\|\boldsymbol{\Delta}\|}{A_t^{1/2}}\mathbf{I}_d + 3\frac{\phi_s^2}{A_t}\boldsymbol{\Delta}\boldsymbol{\Delta}^{\mathrm{T}}$, $\partial_t^2\boldsymbol{\partial}_{\boldsymbol{s}}\widetilde{K} = 6\phi_s^2\phi_t^2\frac{c(\boldsymbol{\theta})}{A_t}\left\{f_{11} - f_{21}\frac{\phi_t^2\delta^2}{A_t}\right\}\boldsymbol{\Delta}$, where $f_{21} = 12 - \frac{11\sqrt{3}}{2}\frac{\phi_s\|\boldsymbol{\Delta}\|}{A_t^{1/2}} + \frac{3}{2}\frac{\phi_s^2\|\boldsymbol{\Delta}\|^2}{A_t} = -\left\{\left(\sqrt{3}\frac{\phi_s\|\boldsymbol{\Delta}\|}{A_t^{1/2}} - 6\right)f_{11} + \frac{A_t}{\phi_t^2\delta}\frac{\partial}{\partial\delta}f_{11}\right\}$ and finally $\partial_t^2\boldsymbol{\partial}_{\boldsymbol{s}}^2\widetilde{K} = 3\phi_s^2\phi_t^2\frac{c(\boldsymbol{\theta})}{A_t}\left\{f_{12} - f_{22}\frac{\phi_t^2\delta^2}{A_t}\right\}$, where $f_{22} = 24\mathbf{I}_d - 35\sqrt{3}\frac{\phi_s}{A_t^{1/2}}\frac{\boldsymbol{\Delta}\boldsymbol{\Delta}^{\mathrm{T}}}{\|\boldsymbol{\Delta}\|} - 11\sqrt{3}\frac{\phi_s\|\boldsymbol{\Delta}\|}{A_t^{1/2}}\mathbf{I}_d + 39\frac{\phi_s^2}{A_t}\boldsymbol{\Delta}\boldsymbol{\Delta}^{\mathrm{T}} + 3\frac{\phi_s^2\|\boldsymbol{\Delta}\|^2}{A_t}\mathbf{I}_d - 3\sqrt{3}\frac{\phi_s^3\|\boldsymbol{\Delta}\|}{A_t^{3/2}}\boldsymbol{\Delta}\boldsymbol{\Delta}^{\mathrm{T}} = -\left\{\left(\sqrt{3}\frac{\phi_s\|\boldsymbol{\Delta}\|}{A_t^{1/2}} - 6\right)f_{12} + \frac{A_t}{\phi_t^2\delta}\frac{\partial}{\partial\delta}f_{12}\right\}$. These recurrence relations for f above are increasingly useful when computing higher order derivatives as is seen in the following subsections. Statistical inference is sought on the spatiotemporal differential process, $\boldsymbol{\mathcal{L}}^*Z(\boldsymbol{s}_0,t_0) = (Z(s_{\mathcal{S}},t_{\mathcal{T}}),\boldsymbol{\partial}_{\boldsymbol{s}}Z(\boldsymbol{s}_0,t_0)^{\mathrm{T}},\partial_tZ(\boldsymbol{s}_0,t_0),\partial_t\boldsymbol{\partial}_{\boldsymbol{s}}Z(\boldsymbol{s}_0,t_0)^{\mathrm{T}})^{\mathrm{T}}$, at an arbitrary space-time location, $(\boldsymbol{s}_0,t_0)$. This requires evaluation of the derivatives pertaining to the variance-covariance sub-matrix as $(\|\boldsymbol{\Delta}\|,|\delta|)\to 0$. They combine to produce

$$\mathbf{V}_{Z,\boldsymbol{\mathcal{L}}^*Z} = \sigma^2\begin{pmatrix} K & \boldsymbol{\partial}_{\boldsymbol{s}}K^{\mathrm{T}} & \partial_tK & \partial_t\boldsymbol{\partial}_{\boldsymbol{s}}K^{\mathrm{T}} \\ -\boldsymbol{\partial}_{\boldsymbol{s}}K & 3\phi_s^2\mathbf{I}_d & 0 & 0 \\ -\partial_tK & 0 & 2\phi_t^2 & 0 \\ \partial_t\boldsymbol{\partial}_{\boldsymbol{s}}K & 0 & 0 & 12\phi_s^2\phi_t^2\mathbf{I}_d \end{pmatrix}, \tag{S21}$$

which is the required cross-covariance matrix for obtaining posterior samples of $\boldsymbol{\mathcal{L}}^*Z(\boldsymbol{s}_0,t_0)$. The variance-covariance matrix pertaining to the differential processes is a *diagonal matrix*.

S12.2.2 Matérn Covariance ($\nu = 5/2$)

The covariance kernel is $\widetilde{K} = \frac{\sigma^2}{A_t}\left(1 + \sqrt{5}\frac{\phi_s\|\boldsymbol{\Delta}\|}{A_t^{1/2}} + \frac{5}{3}\frac{\phi_s^2\|\boldsymbol{\Delta}\|^2}{A_t}\right)\exp\left(-\sqrt{5}\frac{\phi_s\|\boldsymbol{\Delta}\|}{A_t^{1/2}}\right)$. The required derivatives are obtained via successive differentiation. We require them to compute $\mathbf{V}_{Z,\boldsymbol{\mathcal{L}}^*Z}$ in eq. (S3) at an arbitrary space-time location $(\boldsymbol{s}_0,t_0)$ for the spatiotemporal differential

process,

$$\mathcal{L}^{*}Z(\boldsymbol{s},t) = \Big(Z(s_{\mathcal{S}},t_{\mathcal{T}}), \boldsymbol{\partial}_{\boldsymbol{s}}Z(\boldsymbol{s}_0,t_0)^{\mathrm{T}}, \partial_t Z(\boldsymbol{s}_0,t_0), \widetilde{\boldsymbol{\partial}_{\boldsymbol{s}}}^{2} Z(\boldsymbol{s}_0,t_0)^{\mathrm{T}}, \boldsymbol{\partial}_{\boldsymbol{s}}\partial_t Z(\boldsymbol{s}_0,t_0)^{\mathrm{T}},$$
$$\partial_t^2 Z(\boldsymbol{s}_0,t_0), \widetilde{\boldsymbol{\partial}_{\boldsymbol{s}}}^{2}\partial_t Z(\boldsymbol{s}_0,t_0)^{\mathrm{T}}, \boldsymbol{\partial}_{\boldsymbol{s}}\partial_t^2 Z(\boldsymbol{s}_0,t_0)^{\mathrm{T}}, \widetilde{\boldsymbol{\partial}_{\boldsymbol{s}}}^{2}\partial_t^2 Z(\boldsymbol{s}_0,t_0)^{\mathrm{T}}\Big)^{\mathrm{T}}.$$

Considering the cross-covariance matrix, excepting the terms $\boldsymbol{\partial}_{\boldsymbol{s}}K, \partial_t K, \widetilde{\boldsymbol{\partial}_{\boldsymbol{s}}}^{2} K, \partial_t\boldsymbol{\partial}_{\boldsymbol{s}}K, \partial_t^2 K, \partial_t\widetilde{\boldsymbol{\partial}_{\boldsymbol{s}}}^{2} K$, $\partial_t^2\boldsymbol{\partial}_{\boldsymbol{s}}K$ and $\partial_t^2\widetilde{\boldsymbol{\partial}_{\boldsymbol{s}}}^{2} K$, all other terms are evaluated as $(\|\boldsymbol{\Delta}\|, |\delta|) \to 0$ resulting in the following cross-covariance matrix. Note that compared to Matérn($\nu = 3/2$) it is not a diagonal matrix.

$$\mathbf{V}_{Z,\mathcal{L}^{*}Z} = \sigma^2 \left[\begin{array}{c|cc|ccc|ccc}
\Sigma_Z & \boldsymbol{\partial}_{\boldsymbol{s}}\widetilde{K}^{\mathrm{T}} & \widetilde{\boldsymbol{\partial}_{\boldsymbol{s}}}^{2}\widetilde{K}^{\mathrm{T}} & \partial_t\widetilde{K} & \partial_t\boldsymbol{\partial}_{\boldsymbol{s}}\widetilde{K}^{\mathrm{T}} & \partial_t\widetilde{\boldsymbol{\partial}_{\boldsymbol{s}}}^{2}\widetilde{K}^{\mathrm{T}} & \partial_t^2\widetilde{K} & \partial_t^2\boldsymbol{\partial}_{\boldsymbol{s}}\widetilde{K}^{\mathrm{T}} & \partial_t^2\widetilde{\boldsymbol{\partial}_{\boldsymbol{s}}}^{2}\widetilde{K}^{\mathrm{T}} \\ \hline
-\boldsymbol{\partial}_{\boldsymbol{s}}\widetilde{K} & \frac{5}{3}\phi_s^2\mathbf{I}_d & 0 & 0 & 0 & 0 & 0 & -\frac{20}{3}\phi_s^2\phi_t^2\mathbf{I}_d & 0 \\
\widetilde{\boldsymbol{\partial}_{\boldsymbol{s}}}^{2}\widetilde{K} & 0 & \frac{25}{3}\phi_s^4\mathbf{A} & 0 & 0 & 0 & \frac{20}{3}\phi_s^2\phi_t^2\tilde{I}_d & 0 & -50\phi_s^4\phi_t^2\mathbf{A} \\ \hline
-\partial_t\widetilde{K} & 0 & 0 & 2\phi_t^2 & 0 & -\frac{20}{3}\phi_s^2\phi_t^2\tilde{I}_d^{\mathrm{T}} & 0 & 0 & 0 \\
\partial_t\boldsymbol{\partial}_{\boldsymbol{s}}\widetilde{K} & 0 & 0 & 0 & \frac{20}{3}\phi_s^2\phi_t^2\mathbf{I}_d & 0 & 0 & 0 & 0 \\
-\partial_t\widetilde{\boldsymbol{\partial}_{\boldsymbol{s}}}^{2}\widetilde{K} & 0 & 0 & -\frac{20}{3}\phi_s^2\phi_t^2\tilde{I}_d & 0 & 50\phi_s^4\phi_t^2\mathbf{A} & 0 & 0 & 0 \\ \hline
\partial_t^2\widetilde{K} & 0 & \frac{20}{3}\phi_s^2\phi_t^2\tilde{I}_d^{\mathrm{T}} & 0 & 0 & 0 & 24\phi_t^4 & 0 & -120\phi_s^2\phi_t^4\tilde{I}_d^{\mathrm{T}} \\
-\partial_t^2\boldsymbol{\partial}_{\boldsymbol{s}}\widetilde{K} & -\frac{20}{3}\phi_s^2\phi_t^2\mathbf{I}_d & 0 & 0 & 0 & 0 & 0 & 120\phi_s^2\phi_t^4\mathbf{I}_d & 0 \\
\partial_t^2\widetilde{\boldsymbol{\partial}_{\boldsymbol{s}}}^{2}\widetilde{K} & 0 & -50\phi_s^4\phi_t^2\mathbf{A} & 0 & 0 & 0 & -120\phi_s^2\phi_t^4\tilde{I}_d & 0 & 1200\phi_s^4\phi_t^4\mathbf{A}
\end{array}\right].$$

S12.2.3 Gaussian Covariance

In case $\nu \to \infty$, $\widetilde{K} = \dfrac{\sigma^2}{A_t}\exp\left(-\dfrac{\phi_s^2\|\boldsymbol{\Delta}\|^2}{A_t}\right)$. The required derivatives are obtained via successive differentiation. We require to compute $\mathbf{V}_{Z,\mathcal{L}^{*}Z}$ at an arbitrary space-time location, $(\boldsymbol{s}_0, t_0)$ for the spatiotemporal differential process,

$$\mathcal{L}^{*}Z(\boldsymbol{s},t) = \Big(Z(s_{\mathcal{S}},t_{\mathcal{T}}), \boldsymbol{\partial}_{\boldsymbol{s}}Z(\boldsymbol{s}_0,t_0)^{\mathrm{T}}, \partial_t Z(\boldsymbol{s}_0,t_0), \widetilde{\boldsymbol{\partial}_{\boldsymbol{s}}}^{2} Z(\boldsymbol{s}_0,t_0)^{\mathrm{T}}, \partial_t\boldsymbol{\partial}_{\boldsymbol{s}} Z(\boldsymbol{s}_0,t_0)^{\mathrm{T}},$$
$$\partial_t^2 Z(\boldsymbol{s}_0,t_0), \partial_t\widetilde{\boldsymbol{\partial}_{\boldsymbol{s}}}^{2} Z(\boldsymbol{s}_0,t_0)^{\mathrm{T}}, \partial_t^2\boldsymbol{\partial}_{\boldsymbol{s}} Z(\boldsymbol{s}_0,t_0)^{\mathrm{T}}, \partial_t^2\widetilde{\boldsymbol{\partial}_{\boldsymbol{s}}}^{2} Z(\boldsymbol{s}_0,t_0)^{\mathrm{T}}\Big)^{\mathrm{T}}.$$

Referring to the full covariance matrix, excepting the terms $\boldsymbol{\partial}_{\boldsymbol{s}}K^{\mathrm{T}}, \partial_t K, \widetilde{\boldsymbol{\partial}_{\boldsymbol{s}}}^{2} K, \boldsymbol{\partial}_{\boldsymbol{s}}\partial_t K^{\mathrm{T}}, \partial_t^2 K$, $\widetilde{\boldsymbol{\partial}_{\boldsymbol{s}}}^{2}\partial_t K^{\mathrm{T}}, \boldsymbol{\partial}_{\boldsymbol{s}}\partial_t^2 K^{\mathrm{T}}$ and $\widetilde{\boldsymbol{\partial}_{\boldsymbol{s}}}^{2}\partial_t^2 K^{\mathrm{T}}$, the other terms are evaluated as $(\|\boldsymbol{\Delta}\|, |\delta|) \to 0$ resulting in

$$
\mathbf{V}_{Z,\mathcal{L}^*Z} = \sigma^2 \left(\begin{array}{ccc:ccc:ccc}
\Sigma_Z & \boldsymbol{\partial}_s\widetilde{K}^{\top} & \widetilde{\boldsymbol{\partial}_s}^{2}\widetilde{K}^{\top} & \partial_t\widetilde{K} & \partial_t\boldsymbol{\partial}_s\widetilde{K}^{\top} & \partial_t\widetilde{\boldsymbol{\partial}_s}^{2}\widetilde{K}^{\top} & \partial_t^2\widetilde{K} & \partial_t^2\boldsymbol{\partial}_s\widetilde{K}^{\top} & \partial_t^2\widetilde{\boldsymbol{\partial}_s}^{2}\widetilde{K}^{\top} \\
-\boldsymbol{\partial}_s\widetilde{K} & 2\phi_s^2\mathbf{I}_d & 0 & 0 & 0 & 0 & 0 & -8\phi_s^2\phi_t^2\mathbf{I}_d & 0 \\
\widetilde{\boldsymbol{\partial}_s}^{2}\widetilde{K} & 0 & 4\phi_s^4\mathbf{A} & 0 & 0 & 0 & 8\phi_s^2\phi_t^2\tilde{I}_d & 0 & -24\phi_s^4\phi_t^2\mathbf{A} \\
\hdashline
-\partial_t\widetilde{K} & 0 & 0 & 2\phi_t^2 & 0 & -8\phi_s^2\phi_t^2\tilde{I}_d^{\top} & 0 & 0 & 0 \\
\partial_t\boldsymbol{\partial}_s\widetilde{K} & 0 & 0 & 0 & 8\phi_s^2\phi_t^2\mathbf{I}_d & 0 & 0 & 0 & 0 \\
-\partial_t\widetilde{\boldsymbol{\partial}_s}^{2}\widetilde{K} & 0 & 0 & -8\phi_s^2\phi_t^2\tilde{I}_d & 0 & 24\phi_s^4\phi_t^2\mathbf{A} & 0 & 0 & 0 \\
\hdashline
\partial_t^2\widetilde{K} & 0 & 8\phi_s^2\phi_t^2\tilde{I}_d^{\top} & 0 & 0 & 0 & 24\phi_t^4 & 0 & -144\phi_s^2\phi_t^4\tilde{I}_d^{\top} \\
-\partial_t^2\boldsymbol{\partial}_s\widetilde{K} & -8\phi_s^2\phi_t^2\mathbf{I}_d & 0 & 0 & 0 & 0 & 0 & 144\phi_s^2\phi_t^4\mathbf{I}_d & 0 \\
\partial_t^2\widetilde{\boldsymbol{\partial}_s}^{2}\widetilde{K} & 0 & -24\phi_s^4\phi_t^2\mathbf{A} & 0 & 0 & 0 & -144\phi_s^2\phi_t^4\tilde{I}_d & 0 & 576\phi_s^4\phi_t^4\mathbf{A}
\end{array}\right).
$$

S13 Comparison with clustering, image segmentation, hotspot detection techniques and level set modeling

Wombling can be contrastingly compared to spatiotemporal clustering. While clustering aims to find homogeneous regions and provide predictive probabilities for cluster membership for a new point, wombling seeks predictive process-based inference on rates of change over curvilinear or surface boundaries. Substantive interest focuses on the boundary itself and what distinguishes the regions on either side, rather than on any particular region.

Although several approaches exist for spatiotemporal clustering for example, the spatiotemporal density based scan statistics (ST-DBSCAN) (see, e.g., Birant & Kut 2007) or, the spatiotemporal k-means (see, e.g., Dorabiala et al. 2024), we were unable to find any dependable open-source software for the R-statistical environment. The situation is similar for spatiotemporal DP approaches (see, e.g., Gelfand et al. 2007, Duan et al. 2007, Kottas et al. 2008). In the broader AI/ML scope of research, image segmentation is also related to boundary analysis and can be used as a precursor to provide boundaries that are of possible interest to the user. We select U-Net (see, e.g., Ronneberger et al. 2015), a supervised learning framework, to compare and demonstrate our ideas. It uses a convolutional neural network (CNN). There also exist other approaches that use residual learning (see, e.g., He

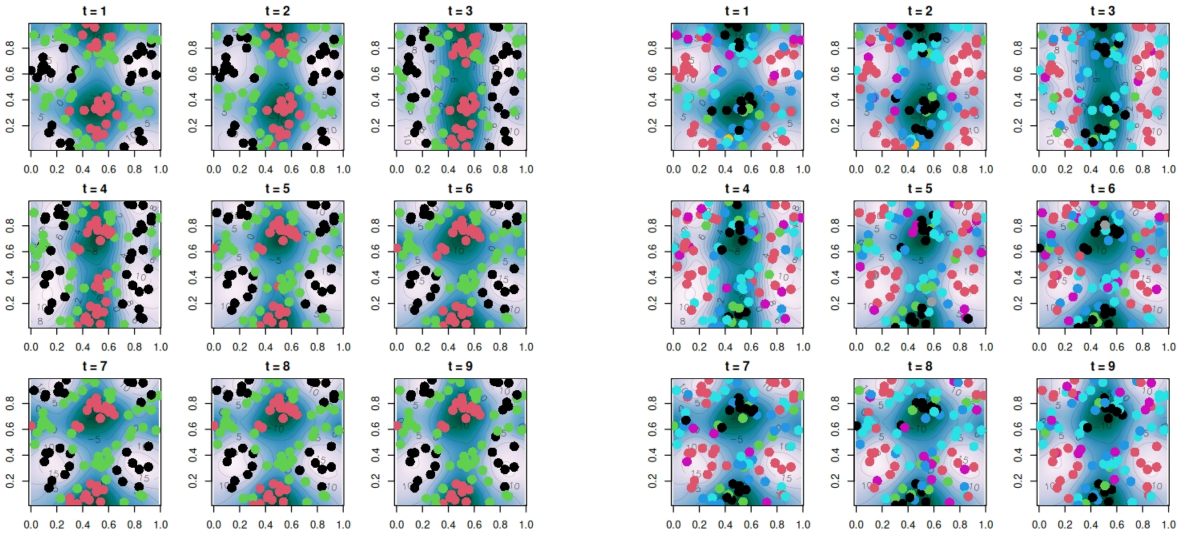

(a) k-Means with 3 clusters

(b) Dirichlet Process Mixture Model fit

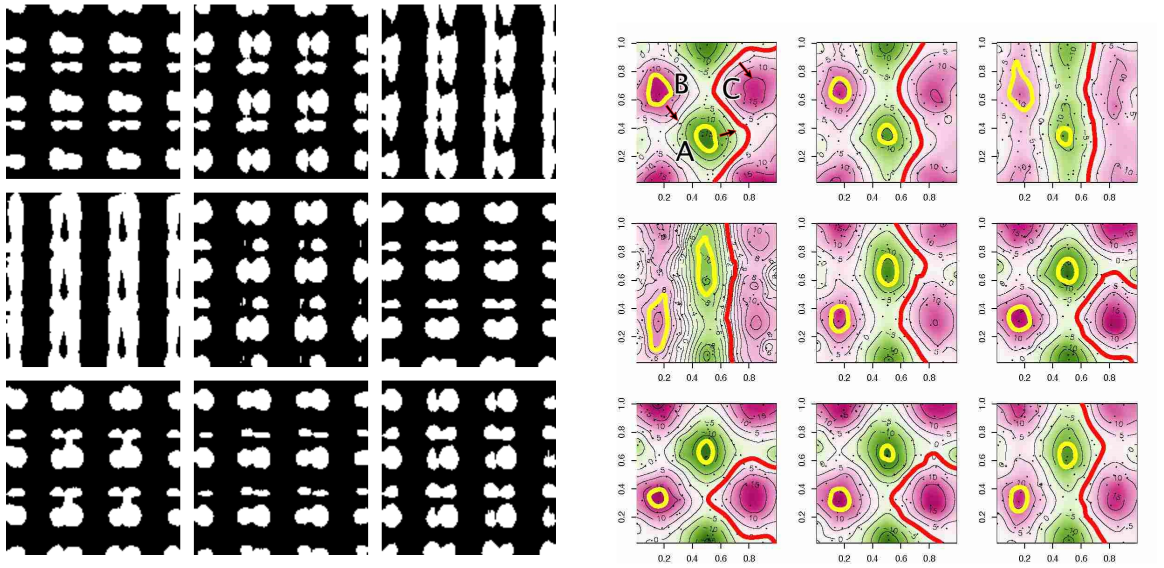

(c) Segmentation masks generated by U-Net.

(d) Curves for wombling surfaces

Figure S2: Comparison between (a) k-means clusters, (b) DPMM clusters (c) segmentation produced by U-Net and (d) the curves chosen for spatiotemporal wombling on simulated data from Pattern 1.

et al. 2016).

Furthering the contrastive comparison featuring the approaches discussed, we demonstrate results on the simulated dataset referred to as Pattern 1 in the manuscript using (a) a standard k-means algorithm with Euclidean distance for the triplet $(\mathbf{s}_i, t_i, y(\mathbf{s}_i, , t_i))^{\mathrm{T}}$, $\mathbf{s} = (s_x, s_y)$ using three clusters that identify peaks, flats and troughs. We use the `kmeans` subroutine in base `R` (b) a DP mixture model (DPMM) using a Gaussian-Inverse-Gamma base measure featured in the `dirichletprocess` package in `R` (Ross & Markwick 2018) to cluster $y(\mathbf{s}_i, t_i)$ and (c) we trained the CNN for 500 epochs using the binary accuracy and

dice metric (see, e.g., Zou et al. 2004) on binary segmentation masks isolating peaks and troughs within interpolated surface plot images. We achieved a validation accuracy of 84%. The images for each time point in Pattern 1 were then supplied to the trained algorithm for boundary detection.

The clusters detected using k-means are shown in fig. S2a using colored points (s_i, t_i) over an interpolated plot for $y(s_i, t_i)$. The DPMM detected 6 clusters which are shown in fig. S2b. This is the generic end result of a clustering procedure. The segmented images from U-Net are shown in fig. S2c where *white* regions indicate zones of rapid change. For the sake of comparison we show the curves used for constructing wombling surfaces in fig. S2d. The boundaries we pick in our simulation overlap with those located by U-Net. Comparing the outputs from clustering, the *region between two clusters* can house possible *wombling boundaries*. Uncertainty quantification along such boundaries is essential. Although quick and easy to implement, clustering is unable to provide any uncertainty quantification on curves (or surfaces) that separate clusters. Image segmentation methods like U-Net require careful training and are unable to provide uncertainty quantification for boundaries. Spatiotemporal wombling provides model-based uncertainty quantification on surface boundaries generated by curves A, B and C. The curves A and B are *contours* within clusters detected by both clustering procedures since they enclose peaks and troughs. However, the curve C is arbitrary. In real world scenarios, as seen in our data examples, when studying precipitation in Northern California, the curve of interest is the central Sierra Nevada Mountain range; in the neuroimaging example, curves delineate regions of the human skull, e.g. the frontal lobe. Lastly, there is no way to characterize clusters, for example, in fig. S2a, the difference between the cluster in red from the cluster in black. Spatiotemporal gradients enable inference on the geometry of the surface indicating that the red points constitute troughs while the black points are peaks.

We also did a contrastive comparison with local hot/cold spot detection approaches. The

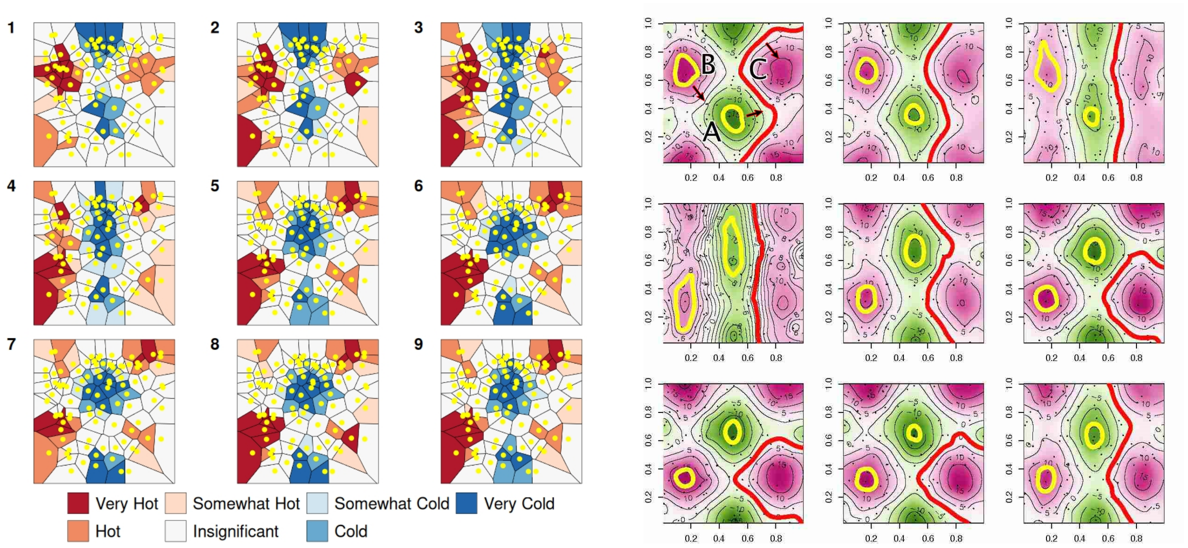

(a) Hot & Cold spots

(b) Curves for wombling surfaces

Figure S3: Comparison between (a) hot & cold spot detection using local Getis-Ord statistic and (b) the curves chosen for spatiotemporal wombling on simulated data from Pattern 1.

results are shown in fig. S3a. We show the selected curves for spatiotemporal wombling on the side in figure fig. S3b for reference purposes. For the detection of hot & cold spots we use the local Getis-Ord statistic (see, e.g., Getis & Ord 1992, Ord & Getis 1995, Bivand & Wong 2018). We were able to find literature on spatiotemporal hot-spot detection (see, e.g., Fanaee-T & Gama 2015, Di Martino et al. 2018, Butt et al. 2020) but no stable implementations for the R-statistical environment. Hot-spot analysis is typically done on areal data. We use Voronoi triangulation (see any plot in fig. S3a) on the random co-ordinates generated for the simulated data in Pattern 1 to obtain polygons corresponding to an areal representation of the data. We used the `sfdep` package in R to obtain corresponding p-values for the Getis-Ord statistic. Breaks of $(0.05, 0.1]$, $(0.01, 0.05]$ and $(0, 0.01]$ were used on the p-values to construct the levels “Somewhat Hot”, “Hot” and “Very Hot” (similarly for cold). The resulting plots are shown in fig. S3a. Comparing the hot and cold spots we see that surfaces produced by curves A and B in our spatiotemporal wombling experiment track peaks and troughs respectively and fall in the hot/cold-spot regions.

The convex hull obtained from the hot/cold-spot polygons can be used to construct a crude contour approximation for use in developing wombling surfaces. Wombling

estimates spatiotemporal gradients along the surface generated using edges of these polygons to determine whether it forms a spatiotemporal boundary. This is not possible using a hotspot detection approach. The surface generated using curve C is arbitrary and does not correspond to any particular spot but rather lies in the "Insignificant" zone separating regions of rapid change.

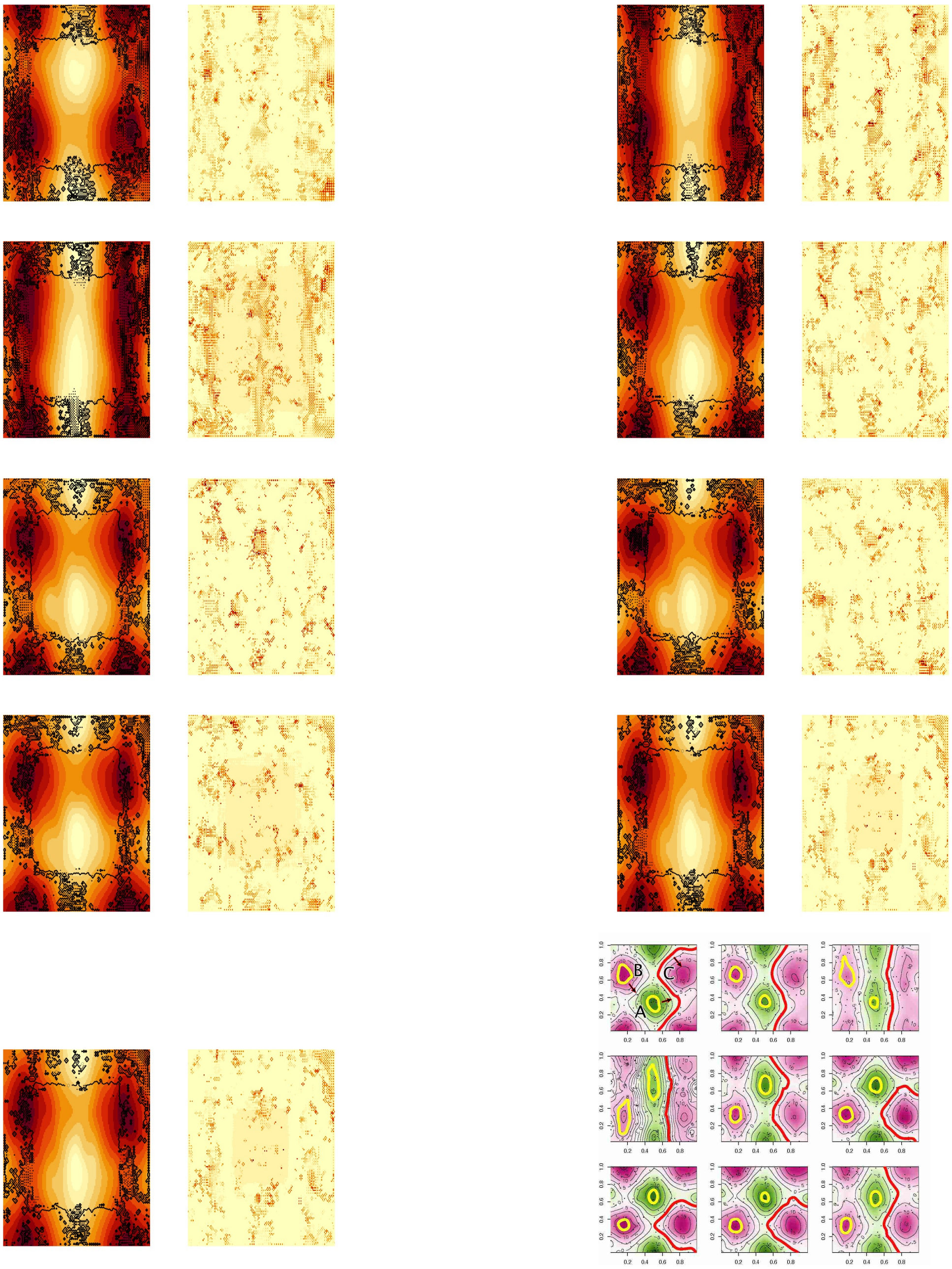

Figure S4: Level set modeling for Pattern 1. Each row (with the exception of the last) consists of the output generated for two time points. For each time point the first picture shows the level set overlay and the second picture shows the values of the level set function, ϕ. The last plot shows curves chosen for wombling.

Level Set Modeling: Level set modeling has a rich history (see, e.g., Osher & Sethian 1988, Malladi et al. 1995, Osher & Fedkiw 2001, Lie et al. 2006, Biswas & Hazra 2022, Saad-Falcon et al. 2024). We provide a brief outline before making remarks. The level set method requires a Lipschitz continuous function often chosen to be the signed distance function (also called level set function) ϕ defined for a boundary $\mathscr{C}$ for $\Omega \subseteq \Re^d$ as $\phi(\boldsymbol{s}) = -\|\boldsymbol{s} - \mathscr{C}\|_2 \, I(\boldsymbol{s} \in \Omega^-) + \|\boldsymbol{s} - \mathscr{C}\|_2 \, I(\boldsymbol{s} \in \Omega^+) + 0 \, I(\boldsymbol{s} \in \mathscr{C})$, where Ω^- and Ω^+ indicate regions inside and outside the boundary respectively and $\|\cdot\|_2$ is the Euclidean norm. The normal to $\mathscr{C}$ and the mean curvature are $\boldsymbol{n}(\boldsymbol{s}) = \nabla\phi(\boldsymbol{s})/|\nabla\phi(\boldsymbol{s})|$ and $\kappa(\boldsymbol{s}) = \nabla \cdot \boldsymbol{n}(\boldsymbol{s})$ respectively. The curve $\mathscr{C}$ evolves under a velocity field, $\boldsymbol{v} = (u, v, w)^{\mathrm{T}}$ according to the equation $\frac{\partial\phi}{\partial t} + \boldsymbol{v} \cdot \boldsymbol{n} \, |\nabla\phi| = 0$. Solutions to this equation (for details see, e.g., Sussman et al. 1994, 1998) provides us with the required level sets.

The level set method is used primarily for shape modeling, computer experiments and image classification. We would like to make some remarks and some possible directions for future work in the following point-wise discussion.

1. The end result of level set modeling comprises the detected level sets and the value of the level set function. There is no uncertainty quantification performed on the level sets detected. Alternatively, Bayesian wombling assumes that the investigator has specific curves that are of scientific interest and performs model-based uncertainty quantification on them. There is precedent for automatic construction and detection of boundaries using significant gradients in the response (see, e.g., Sec. 5, Banerjee & Gelfand 2005). Future work can also explore a joint criteria involving both gradient and curvature in the response. Albeit a time-consuming approach, another alternative is to generate the boundaries using the level set method followed by uncertainty quantification on boundary segments using Bayesian wombling.

2. Computationally, the level set method is implemented using finite differences. Finite differences produce unstable inference for smaller step-sizes (for more discussion,

see, Banerjee et al. 2003). A model based approach like Bayesian wombling offers computationally stable and exact closed form posterior inference on rates of change.

3. Specifically for a spatiotemporal response, the level set method requires a space-time level set function $\phi(\boldsymbol{s}, t)$. As expected, the ensuing computation is sufficiently complex (see, e.g., Boledi et al. 2022). For the sake of comparison we perform a brief simulation that employs the spatial version for every time point independently. We used the simulated data in Pattern 1 from Sec. 5 of the manuscript. The results are shown in Figure S4. For comparison, we also show the curves used for our simulation. Evidently, there is a high overlap between the level sets detected and our choice of curves.

We would like to make some comments that might possibly help with clarification of the proposed developments. Our manuscript (and Banerjee & Gelfand (2006), Halder et al. (2024)) deals with statistical methods for discerning whether segments of a *predetermined* curve (can be a level set) forms a boundary and *not* methods for explicit construction of the curve which are the focus of methods like U-Net and level set modeling.

S14 Static Wombling

In certain applications, spatiotemporal wombling can be regarded as a fairly straightforward extension of purely spatial wombling. For example, investigators may be interested in evaluating whether a geographic feature (e.g., a mountain range or a river) delineates a region of rapid rates of change. Here, one seeks to estimate the wombling measures over a curve that stays fixed over time. This special case is derived by setting $n_t = 0$. Hence, the directional spatiotemporal differential process $\boldsymbol{\mathcal{L}}^*_{0,\boldsymbol{n}_s} Z(\boldsymbol{s}, t) = \widetilde{\boldsymbol{n}}_{\boldsymbol{s},-1} \boldsymbol{\mathcal{L}}_{\boldsymbol{s}} Z(\boldsymbol{s}, t)$ is 2×1 consisting of the directional spatial derivative and curvature of $Z(\boldsymbol{s}, t)$. Consequently, the wombling measures only capture the total and average spatial derivative and curvature.

Flux in the spatiotemporal derivative and curvature processes are muted. Using $\boldsymbol{\mathcal{L}}^*_{1,\boldsymbol{n_s}}Z(\boldsymbol{s},t)$ is an alternative to produce spatiotemporal derivative and curvature wombling measures. However, this alters the normal, $\overline{\boldsymbol{n}}(\boldsymbol{s},t)$, thereby removing the geometric interpretation associated with the wombling measures. We provide some examples.

The parametric line segment $C = \{\boldsymbol{s}_0 + \omega\boldsymbol{u} : \|\boldsymbol{u}\| = 1, \omega \in [0,1]\}$ ranges over $\{t_0 + v : v \in [0,1]\}$ to form $\mathscr{C}$ containing the co-planar points, $\left\{\binom{\boldsymbol{s}_0}{t_0}, \binom{\boldsymbol{s}_0+\boldsymbol{u}}{t_0}, \binom{\boldsymbol{s}_0}{t_0+1}, \binom{\boldsymbol{s}_0+\boldsymbol{u}}{t_0+1}\right\}$. The normal to $\mathscr{C}$ is $\overline{\boldsymbol{n}} = \binom{\overline{\boldsymbol{n}}_{\boldsymbol{s}}}{0}$, where $\overline{\boldsymbol{n}}_{\boldsymbol{s}} = (u_y, -u_x)^{\mathrm{T}} = \boldsymbol{u}^{\perp}$. It is independent of (ω, v) and $\|\overline{\boldsymbol{n}}\| = 1$. Hence, $\mathscr{C} = \left\{(x,y,z) : \overline{\boldsymbol{n}}^{\mathrm{T}}\begin{pmatrix} x\\ y\\ z\end{pmatrix} = \overline{\boldsymbol{n}}^{\mathrm{T}}\begin{pmatrix} s_{0x}\\ s_{0y}\\ t_0\end{pmatrix}\right\}$. The vector of directional differential processes is $\boldsymbol{\mathcal{L}}^*_{0,\boldsymbol{u}^{\perp}}Z(\boldsymbol{s},t)$. Then, we have $\boldsymbol{\Gamma}(\mathscr{C}) = \int_{t_0}^{t_0+1}\int_0^1 \boldsymbol{\mathcal{L}}^*_{0,\boldsymbol{u}^{\perp}}Z(\boldsymbol{s}(\omega),v)\,d\omega\,dv = \left(\int_{t_0}^{t_0+1}\int_0^1 \boldsymbol{u}^{\perp\mathrm{T}}\boldsymbol{\partial_s}Z(\boldsymbol{s}(\omega),v)\,d\omega\,dv, \int_{t_0}^{t_0+1}\int_0^1 \{\widetilde{\boldsymbol{u}^{\perp}}^{\otimes 2}\}^{\mathrm{T}}\widetilde{\boldsymbol{\partial}}_{\boldsymbol{s}}^{2}Z(\boldsymbol{s}(\omega),v)\,d\omega\,dv\right)^{\mathrm{T}}$. Next, consider a half-quadrant of radius r, $C = \{(r\cos\omega, r\sin\omega) : \omega \in [0,\pi/2]\}$ ranging over $\{t_0 + v : v \in [0,1]\}$, generating the quarter-circular sheet $\mathscr{C} = \{(r\cos\omega, r\sin\omega, t_0+v) : \omega \in [0,\pi/2], v \in [0,1]\}$. The normal to $\mathscr{C}$ is $\overline{\boldsymbol{n}}(\boldsymbol{s}(\omega),v) = \binom{\overline{\boldsymbol{n}}_{\boldsymbol{s}}(\omega)}{0}$, where $\overline{\boldsymbol{n}}_{\boldsymbol{s}}(\omega) = (r\cos\omega, -r\sin\omega)^{\mathrm{T}}$ with, $\|\overline{\boldsymbol{n}}(\boldsymbol{s}(\omega),v)\| = r$. The area, $\mathscr{A}(\mathscr{C}) = \int_{t_0}^{t_0+1}\int_0^{\pi/2} r\,d\omega\,dv = \frac{r\pi}{2}$ and $\overline{\boldsymbol{\Gamma}}(\mathscr{C}) = \frac{2}{\pi}\int_{t_0}^{t_0+1}\int_0^{\pi/2} \boldsymbol{\mathcal{L}}^*_{0,\boldsymbol{n_s}}Z(\boldsymbol{s}(\omega),v)\,d\omega\,dv = \left(\frac{2}{\pi}\int_{t_0}^{t_0+1}\int_0^{\pi/2} \boldsymbol{n_s}(\omega)^{\mathrm{T}}\boldsymbol{\partial_s}Z(\boldsymbol{s}(\omega),v)\,d\omega\,dv, \frac{2}{\pi}\int_{t_0}^{t_0+1}\int_0^{\pi/2} \{\widetilde{\boldsymbol{n_s}}(\omega)^{\otimes 2}\}^{\mathrm{T}}\widetilde{\boldsymbol{\partial}}_{\boldsymbol{s}}^{2}Z(\boldsymbol{s}(\omega),v)\,d\omega\,dv\right)^{\mathrm{T}}$. In the foregoing examples, using $\boldsymbol{\mathcal{L}}^*_{1,\boldsymbol{n_s}}Z(\boldsymbol{s},t)$ produces the complete vector of eight wombling measures. Lastly, although the curve evolves over time, $n_t = n_t(\omega, v)$ can still be zero—if the vectors $\begin{pmatrix}\frac{\partial s_x(\omega,v)}{\partial v}\\ \frac{\partial s_y(\omega,v)}{\partial v}\end{pmatrix}$ and $\begin{pmatrix}\frac{\partial s_x(\omega,v)}{\partial \omega}\\ \frac{\partial s_y(\omega,v)}{\partial \omega}\end{pmatrix}$ are linearly dependent. We exclude such pathological cases.

S15 Additional Applications

S15.1 Precipitation in Northern California

The state of California receives 90% of its rainfall during the months of October to April. The Sierra Nevada mountain range, part of the American Cordillera, is a prominent topographic feature of the state. The presence of a mountain range affects precipitation in

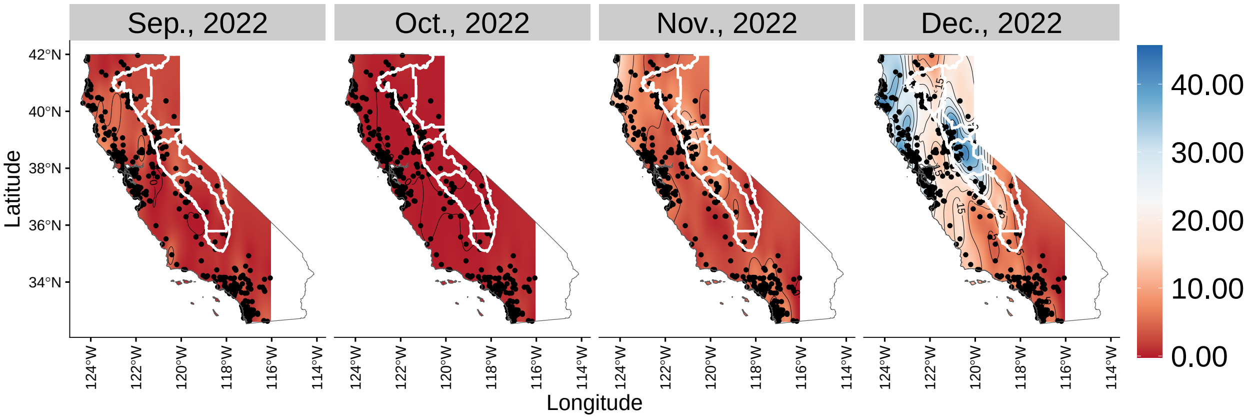

Figure S5: Monthly precipitation (in cms.) for the state of California from September to December, 2022. The Sierra Nevada Conservancy sub-regions are marked in `white`.

Table S1: Posterior estimates accompanied by HPD intervals for process parameters from analyzing precipitation in California during September–December, 2022.

Parameter	Estimate	Parameter	Estimate
σ^2 (Space-time Var.)	74.40 (58.77, 89.76)	ϕ_t (Temporal)	4.74 (2.38, 9.06)
τ^2 (Error Var.)	11.15 (10.28, 12.18)	β_0 (Intercept)	6.82 (6.64, 7.01)
ϕ_s (Spatial)	1.32 (1.15, 1.44)	β_1 (Elevation)	3.44×10^{-3} $(-0.05, 7.08)\times 10^{-3}$

the surrounding areas, causing clouds to form over the windward side and rain-shadow regions on the leeward side, producing the *orographic effect* (see, e.g., Marra et al. 2021). Topographic complexity of the feature effects geophysical manifestation of the orographic effect. Figure S5 captures this in the northern California region during the year 2022. We explore further statistical quantification of the orographic effect using spatiotemporal rates of change and wombling.

Precipitation data is obtained from the National Ocean and Atmospheric Administration (NOAA) for 411 stations strewn across the state recording daily measurements (in tenths of mms.). They are aggregated to produce monthly totals in cms. Figure S5 shows the interpolated surfaces obtained. Elevation (in meters) of the station is used as a covariate. Table S1 shows posterior estimates of $\boldsymbol{\theta}$ resulting from hierarchical modeling of precipitation. Strength of the spatiotemporal story is measured using $\frac{\widehat{\sigma^2}}{\widehat{\sigma^2+\tau^2}} = \frac{74.40}{74.40+11.15} \times 100 \approx 87\%$. Comparing spatial and temporal ranges—the spatial variation is stronger compared to temporal variation. Figure S6 shows the spatial gradient along the latitude and longitude.

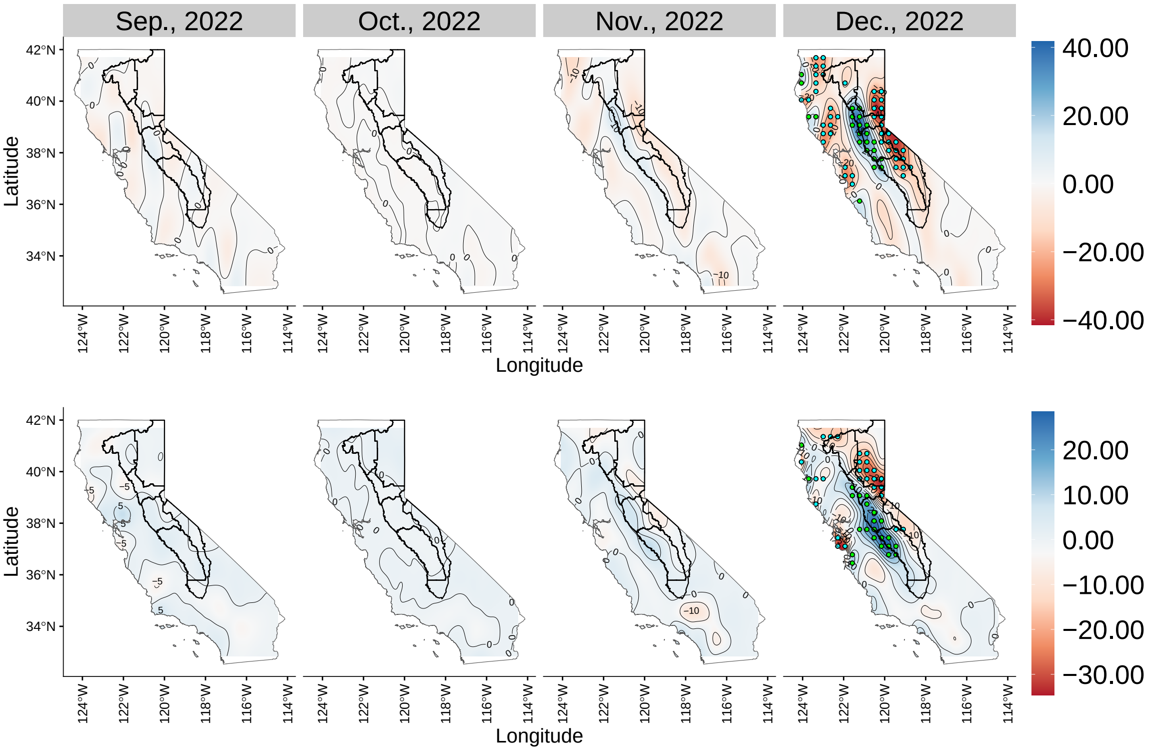

Figure S6: Showing ∂_{s_x} and ∂_{s_y} in precipitation with color-coded grid locations for significant estimates: positive/negative (`green`/`cyan`).

Owing to high precipitation in Dec. we see significant gradients towards the western (windward) side of Sierra Nevada followed by negative gradients on the eastern (leeward) side—a consequence of the orographic effect.

Static wombling is performed on the residual surfaces, $Z(\boldsymbol{s}, t)$ using the north and south central Sierra as a static boundary as seen in the left plot for fig. S7. Performing static wombling only yields measures for the spatial derivatives and curvature (see Section 3.3 of the manuscript). The north and south central Sierra shows a significant overall spatial wombling measure (HPD interval) of 1.00 (0.37, 1.68) but no significant curvature estimated (HPD interval) at 1.18 (-1.16, 3.52). A time-interval level analysis is shown in table S2. The intervals Oct.–Nov. and Nov.–Dec. show significant spatial wombling measures as can also be seen in the significance plots in fig. S7.

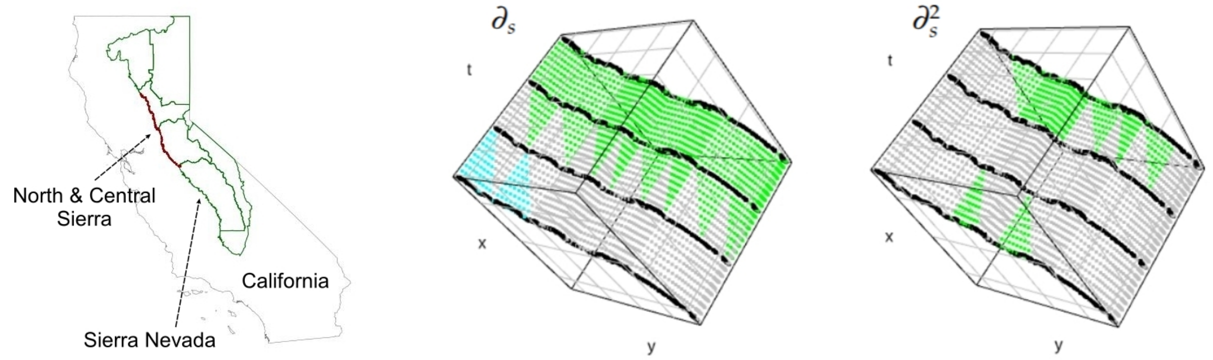

Figure S7: Wombling and significance analysis on the North and Centra Sierra. Plots (left) showing the north and central Sierra to be tested for a wombling boundary (center and right) significance at the triangular region level for the *static wombling* surface.

Table S2: Estimated average wombling measure split by time intervals for the precipitation analysis. They are accompanied by HPD intervals. Estimates that are significant are marked in bold.

$\overline{\Gamma}(\mathscr{C}^*)$	Time	Estimate
∂_s	Sep.–Oct.	-0.24 (-1.65, 1.06)
	Oct.–Nov.	**1.81 (0.34, 3.41)**
	Nov.–Dec.	**7.36 (4.59, 10.55)**
∂_s^2	Sep.–Oct.	2.56 (-3.78, 9.30)
	Oct.–Nov.	1.33 (-5.62, 7.83)
	Nov.–Dec.	6.67 (-0.97, 14.27)

S15.2 Ambient particle concentration ($PM_{2.5}$) and the Canada Wildfires

Record setting wildfires in the year 2023 burned 5% of the entire forest area of Canada. Waves of smoke descended into the US increasing $PM_{2.5}$ in the air, resulting in increased hospitalizations and evacuations. During June 6–8, New York City saw record rises in $PM_{2.5}$ (117μgm^{-3}) from a smoke wave resulting in massive respiratory hospitalizations (see, e.g., Chen et al. 2023). The city awoke to a smog-filled overcast sky on Oct. 1 as another smoke wave passed through. Interpolated spatiotemporal $PM_{2.5}$ surface plots shown in figs. S8a and S8b during these time periods serve as supporting evidence. We

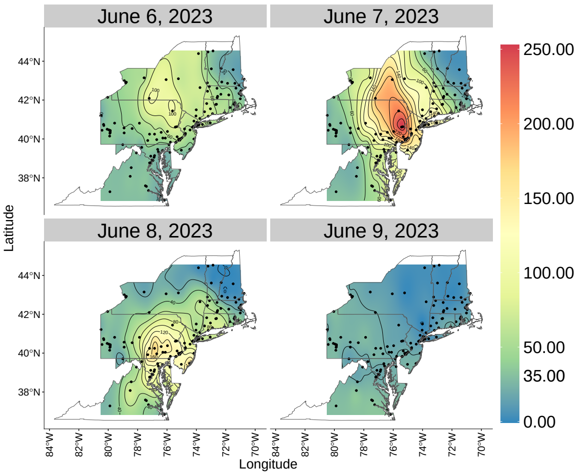

(a) June 6 – 9

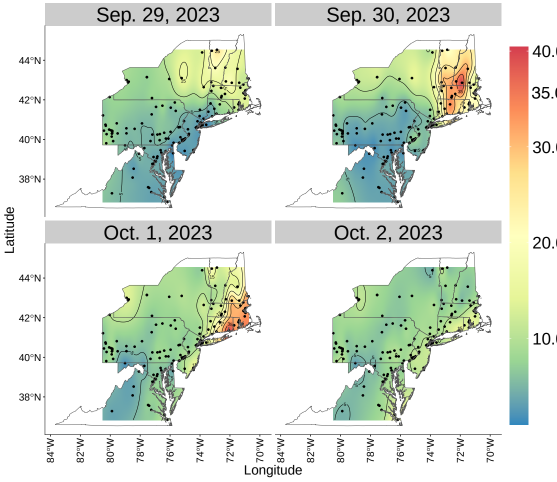

(b) Sep. 29 – Oct. 2

Figure S8: Spatiotemporal $PM_{2.5}$ (in μgm^{-3}) surfaces during the Canada wildfires, 2023. The national daily standard for $PM_{2.5}$ is set at $35\mu gm^{-3}$ by the US Environmental Protection Agency (EPA).

Table S3: Posterior estimates accompanied by HPD intervals for $\boldsymbol{\theta}$ from the $PM_{2.5}$ analyses.

Parameter	Estimate		Parameter	Estimate	
	Jun. 6–9	Sep. 29 – Oct. 2		Jun. 6–9	Sep. 29 – Oct. 2
σ^2	2057.18 (1316.47, 3224.98)	55.59 (42.36, 85.54)	ϕ_t	0.90 (0.66, 1.29)	0.73 (0.60, 0.90)
τ^2	128.78 (109.93, 143.82)	2.99 (2.40, 3.40)	β_0	59.27 (58.22, 60.26)	10.36 (10.20, 10.53)
ϕ_s	0.51 (0.45, 0.61)	0.60 (0.50, 0.73)	$\beta_1 \times 10^{-2}$	-3.31 (-3.81, -2.88)	-0.42 (-0.48, -0.35)

Table S4: Average wombling measures for wombling surfaces selected for the two waves.

Waves	$\overline{\Gamma}(\mathscr{C})$							
	∂_s	∂_s^2	∂_t	$\partial_t\partial_s$	$\partial_t\partial_s^2$	∂_t^2	$\partial_t^2\partial_s$	$\partial_t^2\partial_s^2$
June	**-26.33 (-29.50, -23.03)**	**-28.08 (-32.21, -23.59)**	**-36.84 (-41.57, -32.59)**	**-13.19 (-15.69, -10.27)**	**12.12 (8.12, 16.14)**	**-100.18 (-107.07, -91.79)**	**20.41 (14.38, 27.97)**	**19.19 (11.51, 26.56)**
Sep.–Oct.	**-4.57 (-5.13, -4.04)**	**-1.34 (-2.10,-0.52)**	**-6.35 (-6.87, -5.80)**	**-0.50 (-0.86, -0.13)**	**1.89 (1.19, 2.63)**	**-4.15 (-4.96, -3.37)**	**4.44 (3.72, 5.18)**	0.30 (-0.91, 1.57)

use spatiotemporal wombling to detect boundaries delineating zones of high $PM_{2.5}$ levels in the neighboring regions.

Daily $PM_{2.5}$ data from 144 and 154 monitoring stations is obtained from the EPA for June and Sep.-Oct. respectively. We use elevation of the station as a covariate. Posterior results from modeling $PM_{2.5}$ are shown in table S3. Strength of the spatiotemporal story in both waves is evident, given $\frac{\widehat{\sigma^2}}{\widehat{\sigma^2+\tau^2}} = \frac{2057.18}{2057.18+128.78} \times 100 = 94.11\%$ and $\frac{\widehat{\sigma^2}}{\widehat{\sigma^2+\tau^2}} = \frac{55.59}{55.59+2.99} \times 100 = 94.90\%$ of the variation being spatiotemporal in nature, respectively. Figure S9 shows the mixed spatial-temporal derivative along the longitude.

The residual spatiotemporal surface, $Z(s,t)$ is used for wombling. The wombling surface for June 6–9 is constructed using closed level curves while for Sep.–Oct. we use

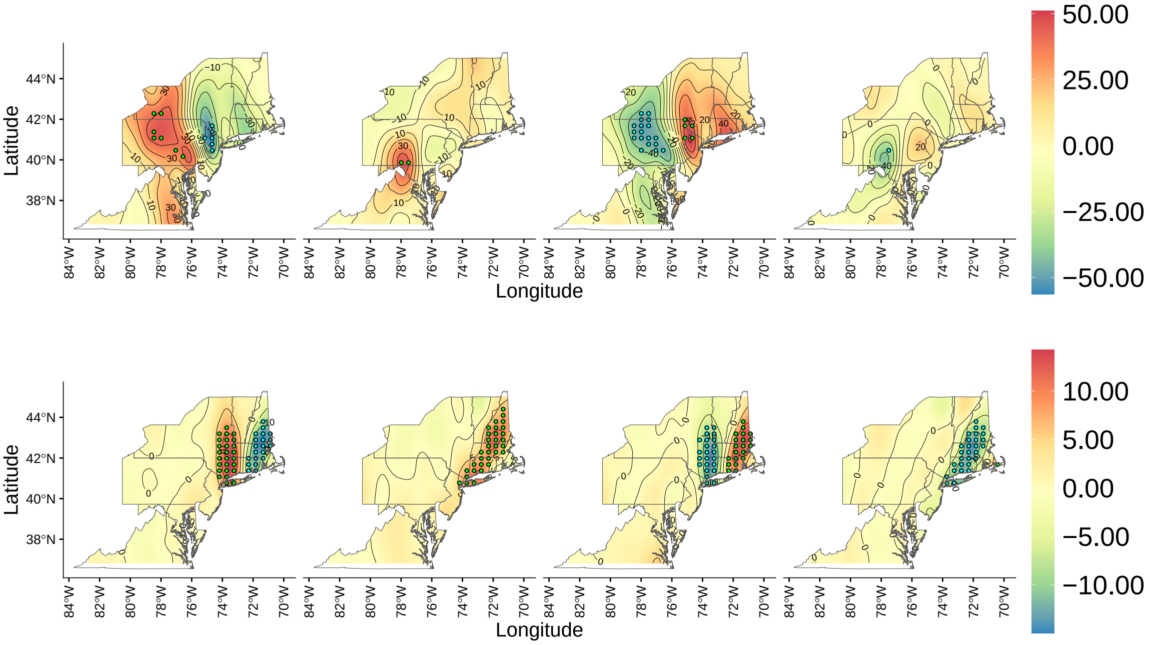

Figure S9: Comparing $\partial_t \partial_{s_x}$ for Jun 6–9 (row 1) versus Sep. 29 – Oct. 2 (row 2). Significant grid locations are color-coded: positive/negative (`green`/`cyan`).

open curves (see fig. S10). Wombling measures for the two surfaces are shown in table S4. Comparing intensities for the two waves, the June 6–9 wave was severe, as is evident from the differences in magnitudes and significance of the overall average wombling measures for the two waves. The rapidity of spatial-temporal changes in $PM_{2.5}$ levels is captured by the differences between mixed derivatives. These changes translated to rapidly deteriorating air-quality resulting in massive respiratory hospitalizations on June 7, 2023 in New York City. The total wombling measures summarized by time-intervals, as seen in Table S11 of the Supplement, serve as further evidence.

S16 Spatiotemporal Surface Wombling: time interval and triangular region level analysis

We supplement the discussion in Section 5 with time-interval and triangular-region level analysis. For the time-interval level analysis we refer to tables S9 and S10 to verify and draw conclusions. The triangular level analysis is carried out through assessing significance

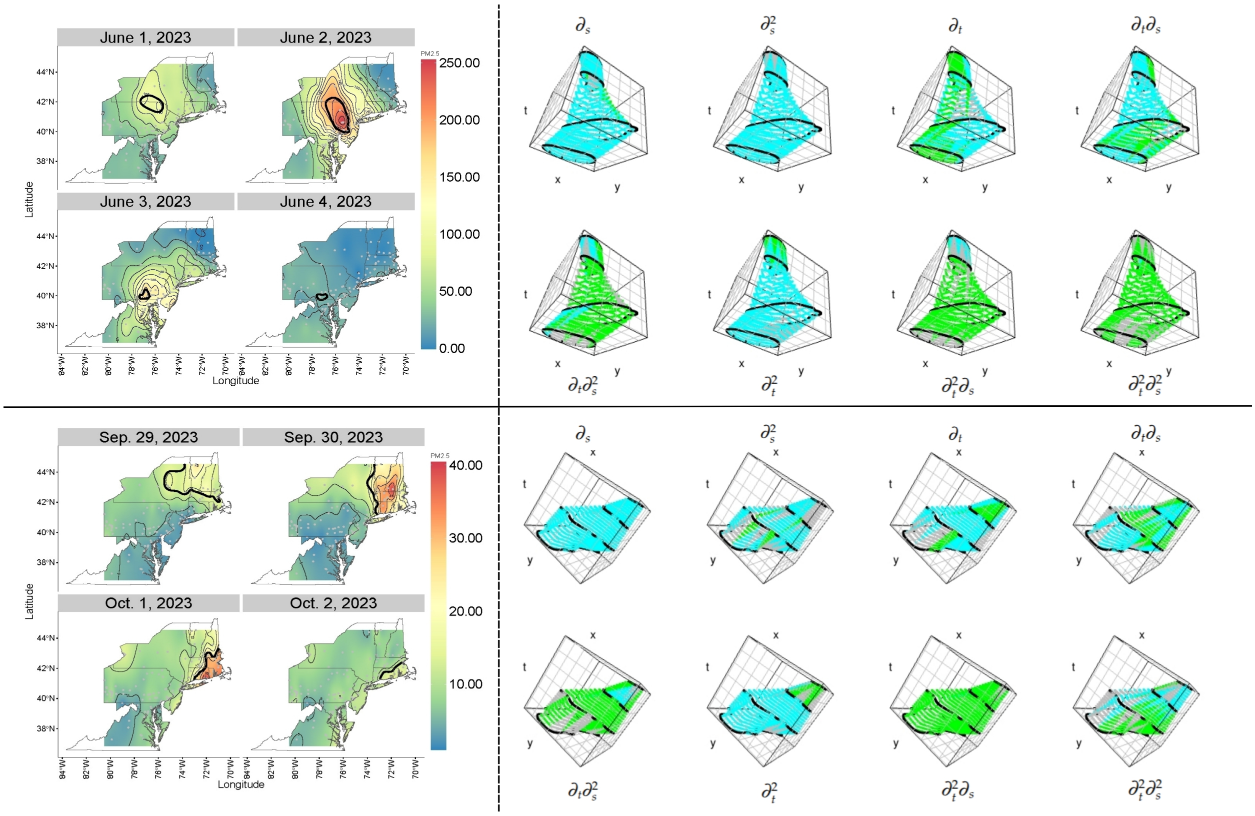

Figure S10: Wombling and significance analysis for Jun 6–9 (row 1) versus Sep. 29 – Oct. 2 (row 2).

for surfaces A, B and C as shown in figs. S15a and S15b. We interpret the wombling measures (WMs) associated with temporal gradient. The others follow a similar pattern. During time intervals (1–2) and (3–4) troughs (peaks) enclosed by surface A (surface B) flatten yielding positive (negative) WMs, while surface C does not experience much change yielding insignificant WMs. Interval (3–4) is one of change where planar curves generating A and B shift spatially and the surface flattens overall. During intervals (4–5), (5–6) and (6–7) the troughs (peaks) re-appear for surface A (surface B), while surface C experiences decreasing temporal gradients before returning to insignificant WMs. Significance at the triangular region level is available in figs. S15a and S15b third plot from the left in row 1. In the following subsections we show tables and figures associated with simulation experiments and applications in the manuscript.

S17 Tables

S17.1 Non-separable Kernels

Table S5: Goodness-of-fit for the spatiotemporal differential processes for simulation experiments performed using Pattern 1.

N_s	N_t	τ^2 (Truth = 1.00)	∂_{s_x}	∂_{s_y}	$\partial^2_{s_{xx}}$	$\partial^2_{s_{xy}}$	$\partial^2_{s_{yy}}$	∂_t	$\partial_t\partial_{s_x}$	$\partial_t\partial_{s_y}$	$\partial_t\partial^2_{s_{xx}}$	$\partial_t\partial^2_{s_{xy}}$	$\partial_t\partial^2_{s_{yy}}$	∂^2_t	$\partial^2_t\partial_{s_x}$	$\partial^2_t\partial_{s_y}$	$\partial^2_t\partial^2_{s_{xx}}$	$\partial^2_t\partial^2_{s_{xy}}$	$\partial^2_t\partial^2_{s_{yy}}$
			RMSE																
30	3	0.74	38.09	21.07	308.59	117.41	238.24	1.34	6.56	10.32	70.33	43.70	124.38	0.79	4.07	6.11	45.61	19.76	67.83
		(0.25, 1.37)	(5.17), (0.8)	(2.54), (0.9)	(45.30), (0.9)	(11.71), (1.0)	(22.21), (1.0)	(0.17), (0.9)	(0.94), (0.9)	(1.03), (0.8)	(15.25), (1.0)	(7.44), (1.0)	(9.90), (1.0)	(0.10), (0.8)	(1.31), (1.0)	(0.66), (1.0)	(16.30), (1.0)	(9.00), (1.0)	(8.11), (1.0)
	6	0.87	34.84	19.94	263.03	119.92	240.95	1.15	5.59	7.72	55.30	38.07	103.23	0.56	2.13	4.36	22.70	13.98	49.86
		(0.61, 1.17)	(4.06), (0.8)	(1.86), (0.9)	(37.91), (0.9)	(8.87), (1.0)	(21.46), (0.9)	(0.13), (0.9)	(0.71), (0.8)	(0.86), (0.8)	(10.19), (1.0)	(3.01), (1.0)	(10.86), (0.9)	(0.05), (0.6)	(0.41), (1.0)	(0.53), (0.8)	(6.10), (1.0)	(2.75), (1.0)	(5.09), (0.9)
	9	0.88	33.48	20.81	277.93	128.89	235.40	1.05	5.18	7.75	51.83	35.52	98.28	0.57	2.76	4.55	28.54	17.71	53.36
		(0.67, 1.15)	(5.26), (0.8)	(1.94), (0.9)	(42.67), (0.9)	(8.26), (0.9)	(19.05), (0.9)	(0.17), (0.9)	(0.37), (0.8)	(0.68), (0.7)	(5.65), (1.0)	(2.99), (1.0)	(8.33), (0.9)	(0.05), (0.7)	(0.30), (1.0)	(0.20), (0.9)	(4.32), (1.0)	(2.25), (1.0)	(2.40), (0.9)
50	3	0.86	25.33	16.52	209.54	112.17	207.27	1.08	6.40	7.86	62.90	39.11	104.08	0.81	4.60	5.38	44.39	21.22	60.49
		(0.54 1.29)	(2.79), (0.9)	(0.85), (1.0)	(28.11), (1.0)	(15.26), (1.0)	(12.48), (0.9)	(0.14), (1.0)	(1.11), (0.8)	(1.27), (0.8)	(18.48), (1.0)	(7.77), (1.0)	(11.58), (0.9)	(0.09), (0.7)	(1.11), (1.0)	(0.58), (1.0)	(13.35), (1.0)	(6.12), (1.0)	(6.30), (1.0)
	6	**0.88**	**24.03**	**15.12**	**190.88**	**105.09**	**185.69**	**0.68**	**4.92**	**6.02**	**46.68**	**33.10**	**84.06**	**0.45**	**2.27**	**3.29**	**22.89**	**12.91**	**41.20**
		(0.70, 1.10)	(2.72), (0.9)	(1.84), (1.0)	(14.76), (1.0)	(6.66), (1.0)	(11.59), (1.0)	(0.15), (1.0)	(0.51), (0.8)	(1.07), (0.7)	(5.21), (1.0)	(3.15), (1.0)	(7.37), (0.9)	(0.05), (0.7)	(0.34), (1.0)	(0.34), (0.9)	(3.47), (1.0)	(2.23), (1.0)	(3.02), (0.9)
	9	0.96	23.84	15.31	194.11	110.39	183.62	0.69	4.42	5.58	41.45	30.15	75.19	0.44	2.39	3.43	23.57	15.29	43.10
		(0.78, 1.16)	(4.78), (0.9)	(1.97), (1.0)	(29.37), (0.9)	(7.58), (1.0)	(16.72), (0.9)	(0.11), (1.0)	(0.41), (0.8)	(0.44), (0.8)	(3.38), (1.0)	(2.07), (1.0)	(5.69), (0.9)	(0.05), (0.7)	(0.33), (1.0)	(0.17), (0.9)	(2.47), (1.0)	(1.28), (1.0)	(1.95), (0.9)
100	3	**0.90**	**14.20**	**12.32**	**140.74**	**96.92**	**166.74**	**0.78**	**5.57**	**6.21**	**53.58**	**32.80**	**85.01**	**0.72**	**4.64**	**4.59**	**43.58**	**16.59**	**50.75**
		(0.70, 1.15)	(2.16), (1.0)	(1.23), (1.0)	(17.63), (1.0)	(7.94), (0.9)	(16.94), (0.9)	(0.07), (1.0)	(0.68), (0.8)	(0.44), (0.7)	(7.65), (1.0)	(4.03), (1.0)	(5.54), (0.9)	(0.09), (0.7)	(0.72), (1.0)	(0.38), (1.0)	(6.78), (1.0)	(2.52), (1.0)	(4.23), (1.0)
	6	0.97	13.37	12.77	132.80	94.34	176.47	0.50	3.80	5.20	40.19	26.07	74.91	0.39	2.05	2.58	19.27	10.88	35.63
		(0.82, 1.17)	(2.03), (1.0)	(0.73), (1.0)	(9.29), (1.0)	(6.95), (1.0)	(8.41), (0.9)	(0.06), (1.0)	(0.43), (0.8)	(0.52), (0.7)	(3.23), (1.0)	(2.56), (1.0)	(2.51), (0.9)	(0.04), (0.7)	(0.13), (1.0)	(0.36), (1.0)	(1.27), (1.0)	(1.16), (1.0)	(3.49), (0.9)
	9	0.96	10.76	11.34	118.83	81.59	166.53	0.46	3.48	4.61	34.53	24.01	68.69	0.36	2.04	3.00	19.51	11.55	38.77
		(0.84, 1.08)	(1.54), (1.0)	(1.15), (1.0)	(8.17), (1.0)	(4.61), (1.0)	(5.24), (0.9)	(0.04), (1.0)	(0.32), (0.8)	(0.48), (0.7)	(2.52), (1.0)	(1.98), (1.0)	(2.89), (0.9)	(0.02), (0.7)	(0.15), (1.0)	(0.15), (1.0)	(1.78), (1.0)	(0.85), (1.0)	(1.49), (0.8)

Table S6: Goodness-of-fit for the spatiotemporal differential processes for simulation experiments performed using Pattern 2.

N_s	N_t	τ^2 (Truth = 1.00)	∂_{s_x}	∂_{s_y}	$\partial^2_{s_{xx}}$	$\partial^2_{s_{xy}}$	$\partial^2_{s_{yy}}$	∂_t	$\partial_t\partial_{s_x}$	$\partial_t\partial_{s_y}$	$\partial_t\partial^2_{s_{xx}}$	$\partial_t\partial^2_{s_{xy}}$	$\partial_t\partial^2_{s_{yy}}$	∂^2_t	$\partial^2_t\partial_{s_x}$	$\partial^2_t\partial_{s_y}$	$\partial^2_t\partial^2_{s_{xx}}$	$\partial^2_t\partial^2_{s_{xy}}$	$\partial^2_t\partial^2_{s_{yy}}$
			RMSE																
30	3	0.44	26.71	20.19	265.40	216.34	257.71	1.43	15.57	12.58	166.88	133.97	172.29	0.92	12.42	11.71	211.41	112.00	215.27
		(0.01, 1.12)	(2.44), (0.8)	(2.40), (0.9)	(60.50), (1.0)	(31.36), (1.0)	(63.81), (1.0)	(0.10), (0.8)	(1.39), (0.9)	(1.73), (1.0)	(58.17), (1.0)	(29.09), (1.0)	(64.63), (1.0)	(0.24), (1.0)	(4.67), (1.0)	(6.95), (1.0)	(195.87), (1.0)	(119.65), (1.0)	(228.83), (1.0)
	6	0.92	24.65	18.13	233.04	200.04	224.00	1.18	13.14	9.81	127.17	108.50	123.88	0.45	7.14	4.22	57.40	42.74	58.77
		(0.61, 1.29)	(2.44), (0.8)	(2.11), (0.9)	(21.50), (1.0)	(19.05), (0.9)	(21.28), (1.0)	(0.18), (0.7)	(1.37), (0.7)	(1.23), (0.8)	(14.07), (1.0)	(11.16), (0.9)	(14.09), (1.0)	(0.05), (0.9)	(0.58), (0.9)	(0.66), (1.0)	(14.10), (1.0)	(7.00), (1.0)	(14.81), (1.0)
	9	0.89	25.43	18.86	255.28	206.19	241.77	0.99	11.37	8.56	115.37	95.07	108.49	0.51	7.64	4.62	61.88	50.69	60.57
		(0.67, 1.18)	(2.87), (0.8)	(2.95), (0.9)	(28.42), (1.0)	(28.46), (0.9)	(29.95), (1.0)	(0.13), (0.8)	(1.14), (0.7)	(1.25), (0.8)	(10.32), (1.0)	(11.67), (0.9)	(12.52), (1.0)	(0.05), (0.8)	(0.38), (0.8)	(0.46), (0.9)	(6.28), (1.0)	(4.67), (1.0)	(8.59), (1.0)
50	3	0.73	20.43	14.42	222.15	173.34	205.63	1.10	12.72	9.79	145.37	109.78	144.18	0.81	12.00	9.37	147.28	96.60	155.16
		(0.36, 1.17)	(1.23), (0.9)	(1.24), (1.0)	(22.46), (1.0)	(9.88), (1.0)	(21.28), (1.0)	(0.07), (0.9)	(0.64), (0.9)	(0.81), (0.9)	(11.49), (1.0)	(7.39), (1.0)	(13.75), (1.0)	(0.13), (0.9)	(1.14), (1.0)	(1.20), (1.0)	(18.93), (1.0)	(11.98), (1.0)	(20.97), (1.0)
	6	**0.89**	**18.25**	**12.91**	**198.32**	**155.60**	**189.54**	**0.80**	**9.79**	**6.65**	**103.26**	**82.24**	**95.38**	**0.33**	**7.78**	**3.14**	**53.41**	**35.49**	**50.46**
		(0.66, 1.18)	(1.66), (0.9)	(1.53), (1.0)	(22.96), (1.0)	(12.90), (0.9)	(20.68), (1.0)	(0.10), (0.9)	(0.82), (0.7)	(0.79), (0.9)	(11.88), (1.0)	(6.86), (0.9)	(11.30), (1.0)	(0.03), (0.9)	(0.61), (0.8)	(0.42), (1.0)	(11.25), (1.0)	(4.51), (1.0)	(11.06), (1.0)
	9	1.01	20.98	13.93	217.75	168.32	210.82	0.79	9.53	6.73	99.50	78.72	99.39	0.44	8.16	3.87	59.44	43.55	56.19
		(0.79, 1.24)	(1.82), (0.9)	(1.27), (0.9)	(22.24), (1.0)	(16.71), (0.9)	(18.56), (1.0)	(0.07), (0.9)	(0.72), (0.7)	(0.57), (0.8)	(8.58), (1.0)	(6.95), (0.9)	(9.20), (1.0)	(0.03), (0.8)	(0.30), (0.8)	(0.30), (1.0)	(6.48), (1.0)	(3.60), (1.0)	(6.44), (1.0)
100	3	**0.87**	**15.34**	**10.45**	**182.46**	**134.34**	**165.86**	**0.86**	**10.42**	**7.63**	**116.20**	**89.68**	**107.22**	**0.83**	**12.21**	**8.26**	**113.92**	**74.32**	**115.24**
		(0.66, 1.12)	(0.99), (0.9)	(0.81), (1.0)	(16.50), (1.0)	(7.59), (0.9)	(12.39), (1.0)	(0.07), (1.0)	(0.54), (0.8)	(0.55), (0.9)	(10.78), (1.0)	(3.70), (1.0)	(6.55), (1.0)	(0.11), (0.8)	(1.17), (0.9)	(0.98), (1.0)	(17.24), (1.0)	(8.76), (1.0)	(13.30), (1.0)
	6	0.93	14.53	9.65	171.10	129.04	153.40	0.57	7.65	5.07	86.70	66.41	76.32	0.28	8.43	2.77	47.06	29.86	44.09
		(0.79, 1.10)	(0.87), (0.9)	(0.58), (1.0)	(9.32), (1.0)	(6.37), (0.9)	(8.41), (1.0)	(0.04), (1.0)	(0.52), (0.7)	(0.26), (0.8)	(5.10), (1.0)	(4.42), (0.9)	(4.71), (1.0)	(0.02), (0.9)	(0.38), (0.8)	(0.17), (1.0)	(4.83), (1.0)	(1.98), (1.0)	(2.85), (1.0)
	9	0.95	15.25	9.87	172.23	127.89	161.00	0.56	7.22	4.73	77.06	59.96	69.60	0.35	8.64	3.07	46.50	33.66	43.01
		(0.82, 1.09)	(0.79), (0.9)	(0.59), (1.0)	(9.55), (1.0)	(5.36), (0.9)	(8.40), (1.0)	(0.04), (1.0)	(0.33), (0.7)	(0.30), (0.8)	(4.70), (1.0)	(2.02), (0.9)	(4.11), (1.0)	(0.03), (0.8)	(0.20), (0.7)	(0.25), (1.0)	(3.56), (1.0)	(1.69), (1.0)	(3.87), (1.0)

S17.2 Separable Kernels

Table S7: Goodness-of-fit for the spatiotemporal differential processes for simulation experiments performed using Pattern 1.

N_s	N_t	τ^2 (Truth = 1.00)	RMSE ∂_{s_x}	∂_{s_y}	$\partial^2_{s_{xx}}$	$\partial^2_{s_{xy}}$	$\partial^2_{s_{yy}}$	∂_t	$\partial_t\partial_{s_x}$	$\partial_t\partial_{s_y}$	$\partial_t\partial^2_{s_{xx}}$	$\partial_t\partial^2_{s_{xy}}$	$\partial_t\partial^2_{s_{yy}}$	∂^2_t	$\partial^2_t\partial_{s_x}$	$\partial^2_t\partial_{s_y}$	$\partial^2_t\partial^2_{s_{xx}}$	$\partial^2_t\partial^2_{s_{xy}}$	$\partial^2_t\partial^2_{s_{yy}}$
30	3	1.33<br>(0.73, 2.10)	37.56<br>(3.56), (0.8)	21.29<br>(2.71), (1.0)	305.51<br>(29.05), (1.0)	129.57<br>(11.66), (1.0)	248.14<br>(32.94), (1.0)	1.44<br>(0.09), (0.9)	6.36<br>(0.56), (0.9)	10.34<br>(0.82), (0.7)	67.82<br>(6.18), (1.0)	35.76<br>(4.61), (1.0)	168.31<br>(5.69), (0.9)	0.87<br>(0.11), (0.9)	3.84<br>(0.59), (1.0)	5.98<br>(0.60), (1.0)	31.62<br>(5.82), (1.0)	16.37<br>(2.46), (1.0)	71.05<br>(3.18), (1.0)
	6	1.08<br>(0.76, 1.48)	35.56<br>(2.00), (0.9)	21.00<br>(1.30), (1.0)	291.35<br>(23.50), (1.0)	124.83<br>(13.89), (1.0)	245.35<br>(19.94), (1.0)	1.16<br>(0.20), (0.9)	5.79<br>(0.37), (0.8)	8.11<br>(0.81), (0.7)	71.05<br>(7.92), (1.0)	36.66<br>(2.65), (1.0)	159.98<br>(6.75), (0.7)	0.57<br>(0.05), (0.8)	2.25<br>(0.35), (1.0)	4.04<br>(0.46), (0.9)	22.90<br>(3.61), (1.0)	12.32<br>(1.44), (1.0)	66.26<br>(2.73), (0.8)
	9	1.03<br>(0.77, 1.30)	33.48<br>(3.94), (0.8)	20.81<br>(2.09), (1.0)	277.93<br>(31.63), (1.0)	128.89<br>(11.03), (1.0)	235.40<br>(17.01), (1.0)	1.05<br>(0.16), (0.9)	5.18<br>(0.33), (0.9)	7.75<br>(1.07), (0.7)	51.83<br>(7.98), (1.0)	35.52<br>(1.48), (1.0)	98.28<br>(8.84), (0.7)	0.57<br>(0.07), (0.7)	2.76<br>(0.26), (1.0)	4.55<br>(0.39), (0.8)	28.54<br>(4.27), (1.0)	17.71<br>(1.41), (1.0)	53.36<br>(3.59), (0.8)
50	3	1.12<br>(0.75 1.58)	26.25<br>(2.88), (0.9)	17.13<br>(2.23), (1.0)	214.23<br>(15.61), (1.0)	116.46<br>(8.72), (1.0)	214.19<br>(25.15), (1.0)	1.15<br>(0.20), (1.0)	5.60<br>(0.77), (0.8)	8.64<br>(0.76), (0.7)	56.49<br>(2.57), (1.0)	32.60<br>(3.22), (1.0)	173.11<br>(3.11), (0.6)	0.82<br>(0.11), (0.9)	3.88<br>(0.64), (1.0)	5.57<br>(0.36), (0.9)	25.61<br>(2.55), (1.0)	15.06<br>(1.88), (1.0)	71.03<br>(1.60), (1.0)
	6	**1.01**<br>(0.79, 1.31)	**24.18**<br>(3.51), (0.9)	**16.98**<br>(1.62), (1.0)	**203.51**<br>(25.25), (1.0)	**114.78**<br>(11.23), (1.0)	**207.63**<br>(14.40), (1.0)	**0.77**<br>(0.19), (1.0)	**4.69**<br>(0.43), (0.8)	**6.47**<br>(0.98), (0.7)	**58.38**<br>(4.38), (1.0)	**30.86**<br>(1.66), (1.0)	**167.77**<br>(5.85), (0.5)	**0.49**<br>(0.05), (0.8)	**2.10**<br>(0.21), (1.0)	**3.24**<br>(0.38), (1.0)	**19.36**<br>(2.00), (1.0)	**11.00**<br>(0.77), (1.0)	**68.03**<br>(2.11), (0.6)
	9	0.99<br>(0.80, 1.22)	23.21<br>(3.05), (0.9)	16.33<br>(1.69), (1.0)	189.47<br>(15.57), (1.0)	109.55<br>(8.48), (1.0)	208.69<br>(13.58), (1.0)	0.71<br>(0.07), (1.0)	4.24<br>(0.31), (0.8)	6.33<br>(0.39), (0.7)	52.98<br>(2.64), (1.0)	28.38<br>(2.39), (1.0)	151.37<br>(2.77), (0.6)	0.54<br>(0.04), (0.7)	2.40<br>(0.19), (1.0)	4.03<br>(0.22), (0.9)	23.38<br>(1.31), (1.0)	13.06<br>(1.15), (1.0)	74.76<br>(1.23), (0.5)
100	3	**0.95**<br>(0.73, 1.21)	**13.97**<br>(2.41), (1.0)	**14.17**<br>(1.31), (1.0)	**142.00**<br>(17.80), (1.0)	**95.28**<br>(7.81), (1.0)	**190.14**<br>(7.43), (0.9)	**0.84**<br>(0.10), (1.0)	**5.28**<br>(0.60), (0.8)	**7.69**<br>(1.00), (0.6)	**50.50**<br>(3.34), (1.0)	**28.71**<br>(2.41), (1.0)	**178.57**<br>(3.99), (0.5)	**0.81**<br>(0.07), (0.9)	**4.18**<br>(0.50), (1.0)	**5.76**<br>(0.45), (0.9)	**23.38**<br>(2.63), (1.0)	**14.44**<br>(1.33), (1.0)	**71.50**<br>(2.52), (0.8)
	6	0.96<br>(0.81, 1.12)	15.34<br>(2.48), (1.0)	14.00<br>(1.63), (1.0)	146.19<br>(12.21), (1.0)	95.03<br>(8.36), (1.0)	192.18<br>(12.63), (0.9)	0.59<br>(0.17), (1.0)	4.10<br>(0.49), (0.8)	5.99<br>(0.66), (0.7)	53.76<br>(1.63), (1.0)	27.00<br>(2.64), (1.0)	174.14<br>(2.94), (0.4)	0.43<br>(0.09), (0.8)	2.07<br>(0.34), (1.0)	2.96<br>(0.23), (1.0)	17.92<br>(1.14), (1.0)	10.31<br>(0.86), (1.0)	69.52<br>(1.20), (0.5)
	9	0.97<br>(0.86, 1.10)	13.23<br>(3.13), (1.0)	13.31<br>(0.77), (1.0)	129.31<br>(19.66), (1.0)	88.65<br>(6.42), (1.0)	194.43<br>(3.43), (0.9)	0.51<br>(0.03), (1.0)	3.45<br>(0.37), (0.8)	5.61<br>(0.39), (0.6)	47.13<br>(1.38), (1.0)	22.84<br>(2.91), (1.0)	158.94<br>(1.83), (0.4)	0.45<br>(0.03), (0.8)	2.02<br>(0.18), (1.0)	3.58<br>(0.20), (0.9)	20.77<br>(0.85), (1.0)	10.94<br>(1.15), (1.0)	77.17<br>(0.82), (0.4)

Table S8: Goodness-of-fit for the spatiotemporal differential processes for simulation experiments performed using Pattern 2.

N_s	N_t	τ^2 (Truth = 1.00)	RMSE ∂_{s_x}	∂_{s_y}	$\partial^2_{s_{xx}}$	$\partial^2_{s_{xy}}$	$\partial^2_{s_{yy}}$	∂_t	$\partial_t\partial_{s_x}$	$\partial_t\partial_{s_y}$	$\partial_t\partial^2_{s_{xx}}$	$\partial_t\partial^2_{s_{xy}}$	$\partial_t\partial^2_{s_{yy}}$	∂^2_t	$\partial^2_t\partial_{s_x}$	$\partial^2_t\partial_{s_y}$	$\partial^2_t\partial^2_{s_{xx}}$	$\partial^2_t\partial^2_{s_{xy}}$	$\partial^2_t\partial^2_{s_{yy}}$
30	3	1.44<br>(0.81, 2.33)	25.37<br>(1.83), (0.8)	18.80<br>(2.26), (0.9)	254.07<br>(23.06), (1.0)	209.71<br>(21.55), (1.0)	235.54<br>(25.56), (1.0)	1.40<br>(0.09), (0.8)	14.72<br>(0.87), (0.7)	11.27<br>(0.98), (0.8)	121.13<br>(8.05), (1.0)	122.14<br>(9.03), (1.0)	121.24<br>(7.98), (1.0)	0.70<br>(0.07), (1.0)	7.58<br>(0.57), (1.0)	5.36<br>(0.38), (1.0)	67.89<br>(7.70), (1.0)	51.24<br>(3.36), (1.0)	68.25<br>(7.50), (1.0)
	6	1.03<br>(0.58, 1.71)	25.66<br>(2.44), (0.9)	19.58<br>(2.46), (1.0)	253.65<br>(22.31), (1.0)	215.64<br>(26.27), (1.0)	242.26<br>(25.41), (1.0)	1.29<br>(0.10), (0.8)	13.55<br>(1.11), (0.7)	10.32<br>(1.03), (0.8)	114.59<br>(14.22), (1.0)	113.97<br>(13.85), (1.0)	114.21<br>(14.30), (1.0)	0.53<br>(0.03), (0.9)	6.82<br>(0.48), (0.9)	4.31<br>(0.52), (1.0)	50.17<br>(6.94), (1.0)	44.69<br>(5.22), (1.0)	49.70<br>(6.86), (1.0)
	9	1.00<br>(0.71, 1.34)	28.38<br>(1.40), (0.8)	21.12<br>(1.32), (0.9)	289.47<br>(20.16), (1.0)	234.66<br>(10.57), (1.0)	278.79<br>(19.08), (1.0)	1.18<br>(0.07), (0.8)	12.49<br>(0.55), (0.7)	9.42<br>(0.50), (0.8)	104.09<br>(4.77), (1.0)	103.96<br>(4.20), (1.0)	104.04<br>(5.07), (1.0)	0.61<br>(0.03), (0.8)	7.25<br>(0.29), (0.8)	4.86<br>(0.16), (0.9)	55.92<br>(2.89), (1.0)	51.81<br>(1.61), (1.0)	55.61<br>(2.69), (1.0)
50	3	1.21<br>(0.79, 1.77)	20.29<br>(1.88), (0.9)	14.44<br>(1.35), (1.0)	211.83<br>(20.24), (1.0)	169.11<br>(14.61), (1.0)	201.95<br>(20.83), (1.0)	1.21<br>(0.12), (0.9)	12.81<br>(1.05), (0.7)	9.59<br>(0.77), (0.8)	103.66<br>(9.43), (1.0)	106.34<br>(7.56), (1.0)	103.38<br>(8.91), (1.0)	0.88<br>(0.20), (0.9)	9.40<br>(1.42), (1.0)	6.86<br>(1.55), (1.0)	70.72<br>(14.18), (1.0)	56.68<br>(9.40), (1.0)	71.60<br>(14.41), (1.0)
	6	**0.93**<br>(0.73, 1.18)	**20.99**<br>(2.30), (0.9)	**14.78**<br>(1.69), (1.0)	**226.43**<br>(22.22), (1.0)	**177.90**<br>(14.31), (1.0)	**209.76**<br>(24.48), (1.0)	**0.99**<br>(0.16), (0.9)	**11.25**<br>(1.27), (0.7)	**7.90**<br>(1.03), (0.8)	**86.48**<br>(13.22), (1.0)	**93.25**<br>(8.46), (0.9)	**86.49**<br>(12.95), (1.0)	**0.47**<br>(0.05), (0.9)	**7.70**<br>(0.33), (0.9)	**3.65**<br>(0.52), (1.0)	**40.47**<br>(6.21), (1.0)	**37.52**<br>(3.42), (1.0)	**40.64**<br>(6.42), (1.0)
	9	0.97<br>(0.77, 1.19)	23.59<br>(1.40), (0.9)	16.58<br>(1.53), (1.0)	246.29<br>(18.00), (1.0)	199.07<br>(14.80), (1.0)	240.90<br>(22.47), (1.0)	0.95<br>(0.07), (0.9)	10.63<br>(0.56), (0.7)	7.58<br>(0.63), (0.8)	83.52<br>(5.50), (1.0)	88.77<br>(5.96), (0.9)	83.32<br>(5.64), (1.0)	0.55<br>(0.04), (0.8)	7.86<br>(0.32), (0.8)	4.33<br>(0.32), (1.0)	47.88<br>(3.86), (1.0)	46.01<br>(2.80), (1.0)	47.87<br>(3.78), (1.0)
100	3	**0.99**<br>(0.72, 1.26)	**15.72**<br>(1.09), (0.9)	**9.97**<br>(0.74), (1.0)	**188.76**<br>(14.06), (1.0)	**136.03**<br>(6.61), (1.0)	**162.48**<br>(12.92), (1.0)	**0.93**<br>(0.07), (1.0)	**10.64**<br>(0.65), (0.7)	**7.62**<br>(0.39), (0.8)	**85.59**<br>(5.08), (1.0)	**89.24**<br>(4.65), (1.0)	**85.39**<br>(4.94), (1.0)	**1.02**<br>(0.18), (0.9)	**11.62**<br>(1.60), (0.9)	**8.55**<br>(1.69), (1.0)	**71.17**<br>(11.70), (1.0)	**69.37**<br>(12.32), (1.0)	**71.97**<br>(11.63), (1.0)
	6	0.98<br>(0.81, 1.18)	15.48<br>(0.96), (1.0)	10.49<br>(0.68), (1.0)	187.59<br>(17.55), (1.0)	136.70<br>(7.98), (1.0)	167.15<br>(23.27), (1.0)	0.67<br>(0.05), (1.0)	8.14<br>(0.47), (0.7)	5.36<br>(0.28), (0.8)	67.31<br>(3.28), (1.0)	67.87<br>(3.93), (0.9)	67.22<br>(3.34), (1.0)	0.44<br>(0.07), (0.9)	8.46<br>(0.38), (0.8)	3.05<br>(0.23), (1.0)	28.61<br>(1.62), (1.0)	28.52<br>(1.94), (1.0)	28.42<br>(1.77), (1.0)
	9	0.96<br>(0.82, 1.11)	17.23<br>(1.95), (0.9)	11.12<br>(0.91), (1.0)	201.00<br>(12.29), (1.0)	145.78<br>(11.79), (1.0)	176.16<br>(12.47), (1.0)	0.68<br>(0.06), (1.0)	8.04<br>(0.57), (0.7)	5.27<br>(0.30), (0.8)	67.68<br>(7.36), (1.0)	66.18<br>(4.73), (0.9)	67.53<br>(7.26), (1.0)	0.50<br>(0.03), (0.8)	8.48<br>(0.24), (0.7)	3.62<br>(0.19), (1.0)	38.68<br>(1.85), (1.0)	37.19<br>(1.51), (0.9)	38.73<br>(1.75), (1.0)

Table S9: True and estimated *total wombling measure* split by time-intervals. They are accompanied by HPD intervals. Estimates that are not significant are marked in bold.

$\Gamma(\mathscr{C}^*)$	Time	A		B		C	
		Estimate	True	Estimate	True	Estimate	True
∂_s	1–2	1.02<br>(0.78, 1.26)	1.03	-1.31<br>(-1.60, -1.04)	-1.30	0.88<br>(0.21, 1.55)	0.99
	2–3	0.51<br>(0.36, 0.67)	0.49	-1.14<br>(-1.41, -0.90)	-1.09	**0.25**<br>(-0.21, 0.69)	0.23
	3–4	1.68<br>(1.17, 2.21)	1.75	-1.91<br>(-2.52, -1.32)	-1.93	**0.02**<br>(-0.67, 0.76)	0.03
	4–5	1.90<br>(1.29, 2.52)	1.98	-1.86<br>(-2.44, -1.27)	-1.89	**-0.05**<br>(-0.93, 0.83)	-0.01
	5–6	1.52<br>(1.21, 1.82)	1.55	-1.63<br>(-1.92, -1.34)	-1.55	1.02<br>(0.37, 1.71)	1.16
	6–7	1.17<br>(0.95, 1.40)	1.17	-1.30<br>(-1.52, -1.06)	-1.23	3.97<br>(3.35, 4.56)	4.16
	7–8	0.58<br>(0.43, 0.73)	0.57	-0.86<br>(-1.03, -0.69)	-0.82	2.79<br>(2.29, 3.27)	2.89
	8–9	0.72<br>(0.53, 0.89)	0.68	-1.03<br>(-1.25, -0.82)	-1.00	0.85<br>(0.35, 1.35)	0.87
∂_s^2	1–2	15.24<br>(12.48, 18.07)	16.41	-19.02<br>(-22.32, -15.66)	-18.50	**1.20**<br>(-4.77, 7.24)	1.49
	2–3	8.57<br>(6.94, 10.33)	9.11	-14.27<br>(-17.26, -11.28)	-14.11	**2.43**<br>(-1.51, 6.31)	2.37
	3–4	36.40<br>(32.34, 40.3)	36.84	-40.26<br>(-45.45, -35.24)	-39.24	**1.13**<br>(-5.12, 7.31)	-0.52
	4–5	43.87<br>(38.98, 48.86)	44.34	-42.07<br>(-47.98, -35.68)	-41.26	**-2.65**<br>(-9.67, 5.01)	-5.46
	5–6	20.53<br>(17.56, 23.55)	20.47	-20.04<br>(-23.43, -16.58)	-19.99	-7.01<br>(-11.6, -2.2)	-10.18
	6–7	17.04<br>(14.66, 19.39)	16.92	-17.11<br>(-19.87, -14.33)	-17.02	**3.87**<br>(-1.82, 9.57)	2.37
	7–8	11.84<br>(10.31, 13.34)	11.69	-13.07<br>(-15.12, -10.95)	-13.11	**2.44**<br>(-2.34, 7.34)	0.59
	8–9	12.31<br>(10.48, 14.15)	12.10	-13.35<br>(-15.99, -10.71)	-14.00	**-2.80**<br>(-7.46, 1.77)	-4.79
∂_t	1–2	0.11<br>(0.07, 0.14)	0.09	-0.09<br>(-0.13, -0.05)	-0.10	**-0.06**<br>(-0.16, 0.04)	-0.07
	2–3	0.04<br>(0.03, 0.05)	0.04	-0.06<br>(-0.08, -0.03)	-0.06	**0.01**<br>(-0.03, 0.05)	0.01
	3–4	1.05<br>(0.78, 1.32)	1.00	1.25<br>(0.88, 1.60)	1.22	-1.51<br>(-1.95, -1.02)	-1.55
	4–5	-0.42<br>(-0.59, -0.26)	-0.38	0.38<br>(0.22, 0.56)	0.36	-0.32<br>(-0.58, -0.05)	-0.26
	5–6	-0.04<br>(-0.05, -0.03)	-0.04	0.05<br>(0.03, 0.06)	0.04	0.04<br>(0.00, 0.07)	0.03
	6–7	**-0.01**<br>(-0.03, 0.00)	-0.02	0.02<br>(0.01, 0.03)	0.02	**0.03**<br>(-0.01, 0.06)	0.02
	7–8	0.02<br>(0.01, 0.03)	0.01	**-0.02**<br>(-0.03, 0.00)	-0.02	**0.02**<br>(-0.02, 0.06)	0.02
	8–9	-0.07<br>(-0.09, -0.06)	-0.08	0.09<br>(0.07, 0.11)	0.10	**-0.06**<br>(-0.12, 0.00)	-0.11
$\partial_t\partial_s$	1–2	-0.20<br>(-0.37, -0.02)	-0.27	0.23<br>(0.02, 0.42)	0.34	-0.80<br>(-1.26, -0.31)	-0.95
	2–3	-0.13<br>(-0.20, -0.07)	-0.11	0.27<br>(0.18, 0.37)	0.20	0.23<br>(0.07, 0.40)	0.16
	3–4	3.56<br>(2.76, 4.37)	3.62	3.95<br>(2.87, 4.95)	4.15	-9.42<br>(-11.35, -7.72)	-9.54
	4–5	1.98<br>(1.26, 2.67)	2.03	-0.95<br>(-1.61, -0.32)	-1.14	4.13<br>(3.11, 5.13)	3.98
	5–6	0.34<br>(0.28, 0.39)	0.28	-0.26<br>(-0.31, -0.21)	-0.26	**0.07**<br>(-0.03, 0.18)	0.08
	6–7	0.11<br>(0.05, 0.17)	0.05	**-0.03**<br>(-0.08, 0.03)	-0.05	0.24<br>(0.09, 0.38)	0.19
	7–8	**-0.01**<br>(-0.08, 0.05)	-0.03	0.06<br>(0.00, 0.13)	0.05	-0.32<br>(-0.52, -0.12)	-0.23
	8–9	0.26<br>(0.15, 0.36)	0.29	-0.40<br>(-0.52, -0.28)	-0.37	**0.01**<br>(-0.27, 0.28)	-0.00

Table S10: True and estimated total wombling measure split by time intervals. They are accompanied by HPD intervals. Estimates that are not significant are marked in bold. Estimates not containing true values are marked in `teal`.

$\Gamma(\mathscr{C}^*)$	Time	A		B		C	
		Estimate	True	Estimate	True	Estimate	True
$\partial_t\partial_s^2$	1–2	-5.08<br>(-8.33, -2.2)	-4.33	4.15<br>(0.45, 7.53)	4.64	**6.58**<br>(-0.11, 13.49)	6.32
	2–3	**-0.82**<br>(-2.08, 0.47)	-0.71	**-0.69**<br>(-2.92, 1.34)	0.51	**-2.68**<br>(-5.68, 0.51)	-4.40
	3–4	-27.29<br>(-42.43, -12.22)	-27.77	-51.48<br>(-73.22, -29.60)	-49.29	80.35<br>(48.53, 114.35)	80.17
	4–5	**-2.97**<br>(-14.25, 8.92)	-0.82	**-13.32**<br>(-27.19, 0.38)	-8.96	21.83<br>(3.10, 40.14)	18.22
	5–6	1.73<br>(0.61, 2.90)	2.28	-2.35<br>(-3.61, -1.06)	-2.37	**-1.97**<br>(-4.07, 0.03)	-0.96
	6–7	**0.02**<br>(-1.05, 1.13)	0.45	**-0.29**<br>(-1.55, 0.95)	-0.52	**1.05**<br>(-1.99, 4.00)	-0.12
	7–8	**-0.56**<br>(-1.69, 0.56)	-0.59	**1.14**<br>(-0.36, 2.62)	0.68	**1.04**<br>(-2.64, 4.54)	-0.46
	8–9	2.61<br>(0.85, 4.33)	2.88	-3.44<br>(-5.87, -1.12)	-3.70	**3.33**<br>(-1.27, 7.73)	5.63
∂_t^2	1–2	0.21<br>(0.13, 0.30)	0.30	-0.35<br>(-0.46, -0.26)	-0.33	-0.53<br>(-0.77, -0.28)	-0.20
	2–3	0.03<br>(0.02, 0.05)	0.05	-0.07<br>(-0.09, -0.04)	-0.07	-0.05<br>(-0.09, -0.01)	-0.00
	3–4	**0.91**<br>(-0.62, 2.50)	0.89	**0.88**<br>(-1.22, 3.08)	-1.39	**1.87**<br>(-1.86, 5.65)	0.79
	4–5	1.74<br>(1.30, 2.21)	1.43	-1.52<br>(-1.97, -1.05)	-1.27	1.92<br>(1.10, 2.70)	0.67
	5–6	0.05<br>(0.04, 0.05)	0.04	-0.04<br>(-0.05, -0.04)	-0.04	0.01<br>(0.00, 0.02)	-0.01
	6–7	0.08<br>(0.08, 0.09)	0.08	-0.08<br>(-0.09, -0.08)	-0.08	-0.08<br>(-0.10, -0.06)	-0.08
	7–8	0.11<br>(0.10, 0.13)	0.11	-0.11<br>(-0.12, -0.09)	-0.11	0.13<br>(0.08, 0.18)	0.16
	8–9	0.10<br>(0.06, 0.13)	0.11	-0.12<br>(-0.17, -0.08)	-0.14	**0.08**<br>(-0.03, 0.18)	0.27
$\partial_t^2\partial_s$	1–2	-1.01<br>(-1.61, -0.43)	-1.22	1.28<br>(0.64, 1.98)	1.40	-3.54<br>(-5.15, -1.99)	-3.73
	2–3	**-0.11**<br>(-0.24, 0.02)	-0.09	0.16<br>(-0.06, 0.39)	0.21	-0.38<br>(-0.73, -0.02)	-0.20
	3–4	**-7.08**<br>(-22.16, 8.22)	-8.89	**9.30**<br>(-12.68, 30.49)	13.25	**1.92**<br>(-32.64, 37.94)	-3.03
	4–5	-6.69<br>(-11.7, -1.75)	-5.87	5.57<br>(0.79, 10.51)	5.84	**2.27**<br>(-5.12, 9.58)	1.29
	5–6	-0.24<br>(-0.3, -0.17)	-0.23	0.27<br>(0.21, 0.33)	0.22	-0.20<br>(-0.34, -0.07)	-0.21
	6–7	-0.24<br>(-0.33, -0.15)	-0.17	0.18<br>(0.10, 0.28)	0.16	-0.76<br>(-0.99, -0.51)	-0.74
	7–8	-0.21<br>(-0.35, -0.08)	-0.27	0.33<br>(0.19, 0.47)	0.31	-1.68<br>(-2.15, -1.24)	-1.62
	8–9	-0.35<br>(-0.58, -0.13)	-0.54	0.61<br>(0.29, 0.94)	0.73	**0.17**<br>(-0.49, 0.89)	0.08
$\partial_t^2\partial_s^2$	1–2	-11.17<br>(-18.47, -4.07)	-16.36	17.66<br>(9.25, 26.29)	18.03	18.73<br>(2.98, 33.62)	14.11
	2–3	**-0.70**<br>(-2.72, 1.15)	-1.18	**0.19**<br>(-3.09, 3.44)	1.19	**-3.83**<br>(-8.88, 1.03)	-2.15
	3–4	**-38.05**<br>(-186.63, 122.86)	-30.46	**-68.52**<br>(-317.43, 177.02)	53.02	**-296.99**<br>(-743.29, 129.76)	-48.13
	4–5	**-23.93**<br>(-82.71, 35.55)	-34.80	**54.47**<br>(-15.23, 126.69)	38.03	**-76.32**<br>(-172.6, 20.09)	-11.36
	5–6	-2.10<br>(-2.99, -1.27)	-1.92	2.39<br>(1.49, 3.36)	2.03	**0.42**<br>(-1.14, 1.98)	0.95
	6–7	-1.54<br>(-2.71, -0.37)	-1.77	2.69<br>(1.37, 4.01)	1.98	**0.34**<br>(-2.96, 3.58)	0.08
	7–8	-4.79<br>(-6.57, -3.1)	-4.06	4.39<br>(2.2, 6.45)	4.44	**-4.30**<br>(-9.61, 0.93)	-4.51
	8–9	-6.03<br>(-8.95, -3.22)	-5.23	4.33<br>(0.59, 8.23)	7.14	**-6.75**<br>(-14.24, 0.86)	-15.45

Table S11: Estimated total wombling measure split by time intervals for the $PM_{2.5}$ analysis. They are accompanied by HPD intervals. Estimates that are not significant are marked in bold.

$\Gamma(\mathscr{C}^*)$	Time	June 6 – 9 Estimate	Sep. 29 – Oct. 2 Estimate
$\boldsymbol{\partial}_s$	1–2	-291.63<br>(-326.07, -256.89)	-49.28<br>(-56.41, -42.40)
	2–3	-253.85<br>(-282.87, -222.63)	-35.56<br>(-39.02, -32.30)
	3–4	-9.34<br>(-12.76, -5.80)	-14.41<br>(-16.02, -12.97)
$\boldsymbol{\partial}_s^2$	1–2	-283.86<br>(-328.55, -232.16)	-16.50<br>(-25.38, -6.52)
	2–3	-282.52<br>(-316.84, -248.03)	-8.37<br>(-12.64, -3.96)
	3–4	-25.28<br>(-33.33, -17.03)	-4.31<br>(-7.65, -0.85)
∂_t	1–2	-265.87<br>(-289.47, -239.92)	-57.01<br>(-63.03, -50.45)
	2–3	-506.81<br>(-582.19, -444.05)	-73.18<br>(-77.87, -68.36)
	3–4	-3.65<br>(-4.41, -2.87)	-7.70<br>(-8.28, -7.15)
$\partial_t\boldsymbol{\partial}_s$	1–2	8.21<br>(-9.09, 27.37)	-8.98<br>(-13.46, -4.26)
	2–3	-284.20<br>(-318.73, -242.77)	-2.83<br>(-5.60, -0.14)
	3–4	-1.93<br>(-2.87, -0.97)	0.95<br>(0.33, 1.60)
$\partial_t\boldsymbol{\partial}_s^2$	1–2	107.04<br>(74.42, 139.73)	10.24<br>(1.81, 18.95)
	2–3	145.63<br>(96.49, 195.38)	27.88<br>(22.76, 33.35)
	3–4	2.77<br>(0.28, 5.10)	2.99<br>(1.17, 4.81)
∂_t^2	1–2	-182.88<br>(-217.80, -144.68)	-30.78<br>(-39.36, -22.05)
	2–3	-1928.07<br>(-2037.92, -1790.01)	-58.78<br>(-67.17, -51.10)
	3–4	-0.14<br>(-0.55, 0.34)	-0.58<br>(-1.11, -0.05)
$\partial_t^2\boldsymbol{\partial}_s$	1–2	126.95<br>(95.55, 153.08)	28.75<br>(21.03, 36.31)
	2–3	302.13<br>(206.92, 434.72)	63.95<br>(56.78, 71.52)
	3–4	1.11<br>(0.56, 1.60)	3.84<br>(3.01, 4.70)
$\partial_t^2\boldsymbol{\partial}_s^2$	1–2	86.33<br>(36.98, 133.26)	10.67<br>(-4.09, 26.08)
	2–3	316.79<br>(205.67, 424.09)	-5.90<br>(-15.84, 4.17)
	3–4	1.29<br>(-0.07, 2.44)	1.80<br>(0.14, 3.87)

Table S12: Estimated total wombling measures ($\times 10^2$) split by time intervals for the EEG analysis. They are accompanied by HPD intervals. Estimates that are not significant are in bold.

$\Gamma(\mathscr{C}^*)$	Time	Alcoholic Estimate	Control Estimate
$\boldsymbol{\partial}_s$	1–2	180.29 (160.08, 201.04)	8.35 (-19.24, 34.95)
	2–3	30.97 (13.46, 51.05)	309.90 (282.57, 338.02)
	3–4	-116.14 (-133.00, -98.24)	369.72 (342.11, 399.08)
	4–5	-86.06 (-103.93, -68.21)	307.01 (282.08, 331.66)
	5–6	-79.70 (-97.02, -62.00)	258.40 (234.22, 281.08)
$\boldsymbol{\partial}_s^2$	1–2	-617.55 (-750.63, -477.67)	-42.90 (-228.8, 155.70)
	2–3	-287.54 (-415.89, -172.75)	-565.06 (-753.63, -374.55)
	3–4	-104.61 (-214.16, 21.59)	-755.23 (-943.36, -567.42)
	4–5	34.67 (-62.01, 124.43)	-471.44 (-650.69, -279.69)
	5–6	27.33 (-84.93, 145.26)	-707.47 (-894.38, -530.20)
∂_t	1–2	1.26 (0.71, 1.81)	-0.31 (-1.76, 1.27)
	2–3	2.29 (0.84, 3.64)	-7.64 (-8.45, -6.83)
	3–4	-1.40 (-1.87, -0.94)	1.11 (0.46, 1.74)
	4–5	-1.18 (-2.47, 0.10)	0.05 (-0.02, 0.12)
	5–6	-0.14 (-0.36, 0.07)	-0.31 (-0.44, -0.18)
$\partial_t\boldsymbol{\partial}_s$	1–2	-5.26 (-6.99, -3.71)	-32.27 (-36.45, -27.97)
	2–3	1.20 (-2.83, 5.16)	-21.63 (-23.80, -19.38)
	3–4	-1.83 (-3.31, -0.49)	-6.54 (-8.47, -4.58)
	4–5	-7.66 (-10.74, -4.39)	0.54 (0.35, 0.75)
	5–6	-0.05 (-0.64, 0.61)	-1.60 (-2.04, -1.19)

Table S13: Estimated total wombling measures (×10^2) split by time intervals for the EEG analysis. They are accompanied by HPD intervals. Estimates that are not significant are in bold.

$\boldsymbol{\Gamma}(\mathscr{C}^*)$	Time	Alcoholic Estimate	Control Estimate
$\partial_t \boldsymbol{\partial}_s^2$	1–2	2.88<br>(-10.51, 17.30)	-65.39<br>(-105.14, -29.25)
	2–3	-1.56<br>(-29.08, 27.45)	8.12<br>(-19.80, 33.41)
	3–4	14.39<br>(2.64, 26.79)	-11.82<br>(-34.85, 9.23)
	4–5	-0.68<br>(-23.07, 22.79)	2.52<br>(0.18, 4.76)
	5–6	-0.03<br>(-5.26, 4.66)	-4.34<br>(-8.61, 0.35)
∂_t^2	1–2	0.61<br>(0.51, 0.73)	5.75<br>(4.88, 6.72)
	2–3	1.02<br>(0.56, 1.48)	-1.54<br>(-1.67, -1.40)
	3–4	-0.57<br>(-0.63, -0.49)	-0.95<br>(-1.02, -0.87)
	4–5	-1.14<br>(-1.71, -0.53)	0.00<br>(-0.00, 0.00)
	5–6	-0.03<br>(-0.06, -0.01)	-0.04<br>(-0.06, -0.02)
$\partial_t^2 \boldsymbol{\partial}_s$	1–2	-2.46<br>(-2.79, -2.14)	1.65<br>(-0.70, 4.11)
	2–3	3.16<br>(1.05, 5.21)	-7.54<br>(-8.67, -6.61)
	3–4	1.39<br>(1.05, 1.73)	-0.92<br>(-1.46, -0.31)
	4–5	-3.56<br>(-6.20, -1.11)	0.01<br>(0.00, 0.01)
	5–6	-0.01<br>(-0.09, 0.05)	-0.04<br>(-0.06, -0.02)
$\partial_t^2 \boldsymbol{\partial}_s^2$	1–2	1.52<br>(-0.73, 4.14)	-23.49<br>(-39.70, -6.29)
	2–3	-19.23<br>(-32.45, -5.83)	4.23<br>(-3.34, 12.63)
	3–4	1.97<br>(-0.16, 4.29)	6.25<br>(1.67, 10.94)
	4–5	3.44<br>(-9.33, 18.73)	-0.02<br>(-0.09, 0.04)
	5–6	0.11<br>(-0.37, 0.60)	0.13<br>(-0.02, 0.31)

S18 Figures

S18.1 Spatiotemporal Curvature

S18.1.1 Pattern 2

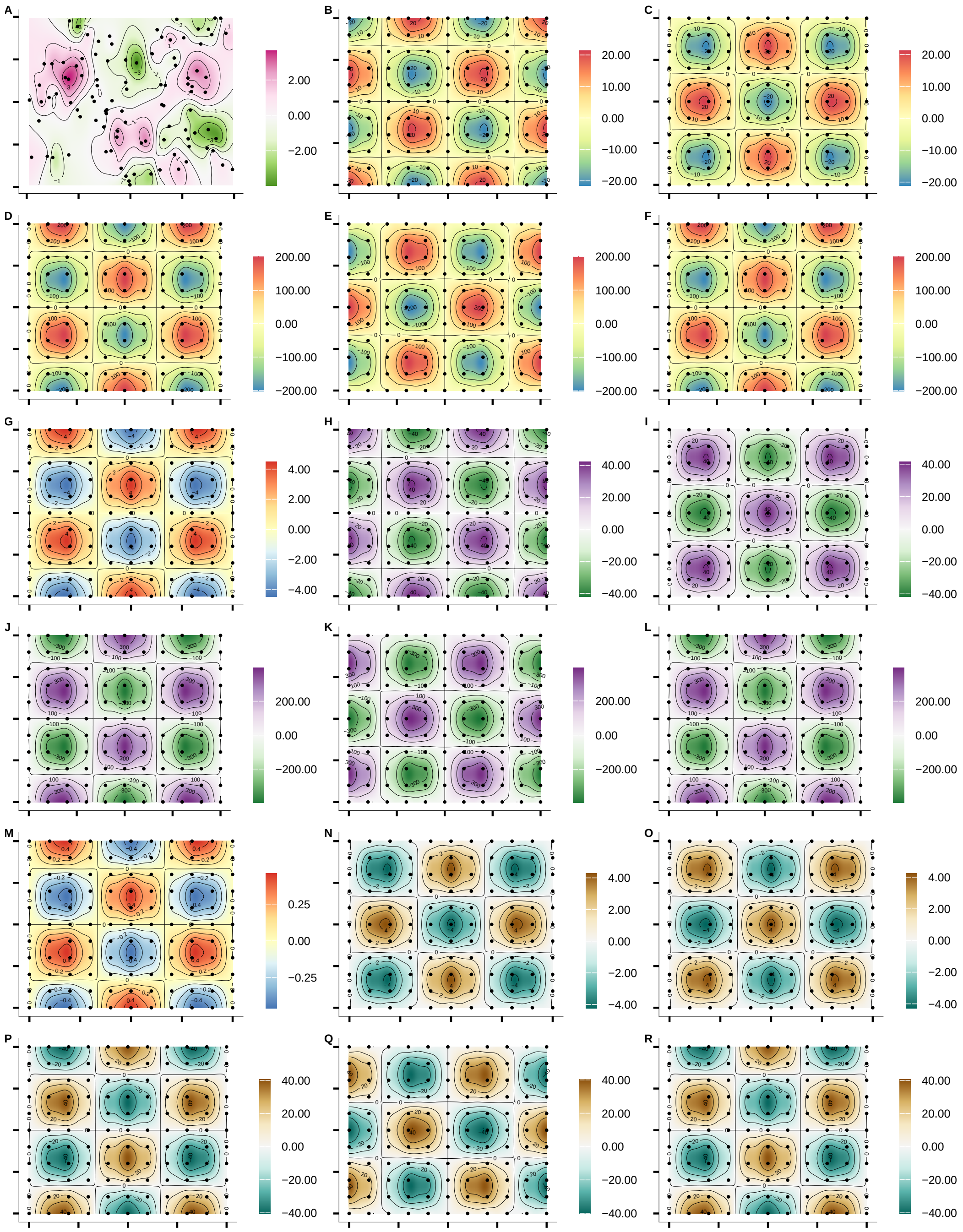

Figure S11: True spatiotemporal process, derivatives and curvature for $t = 3$. (A) response from Pattern 2, (B) spatial gradient along x, (C) along y, (D) spatial curvature along x, (E) along x and y and (F) along y, (G–L) temporal gradients for processes in (A–F), (M–R) temporal curvature for processes in (A–F).

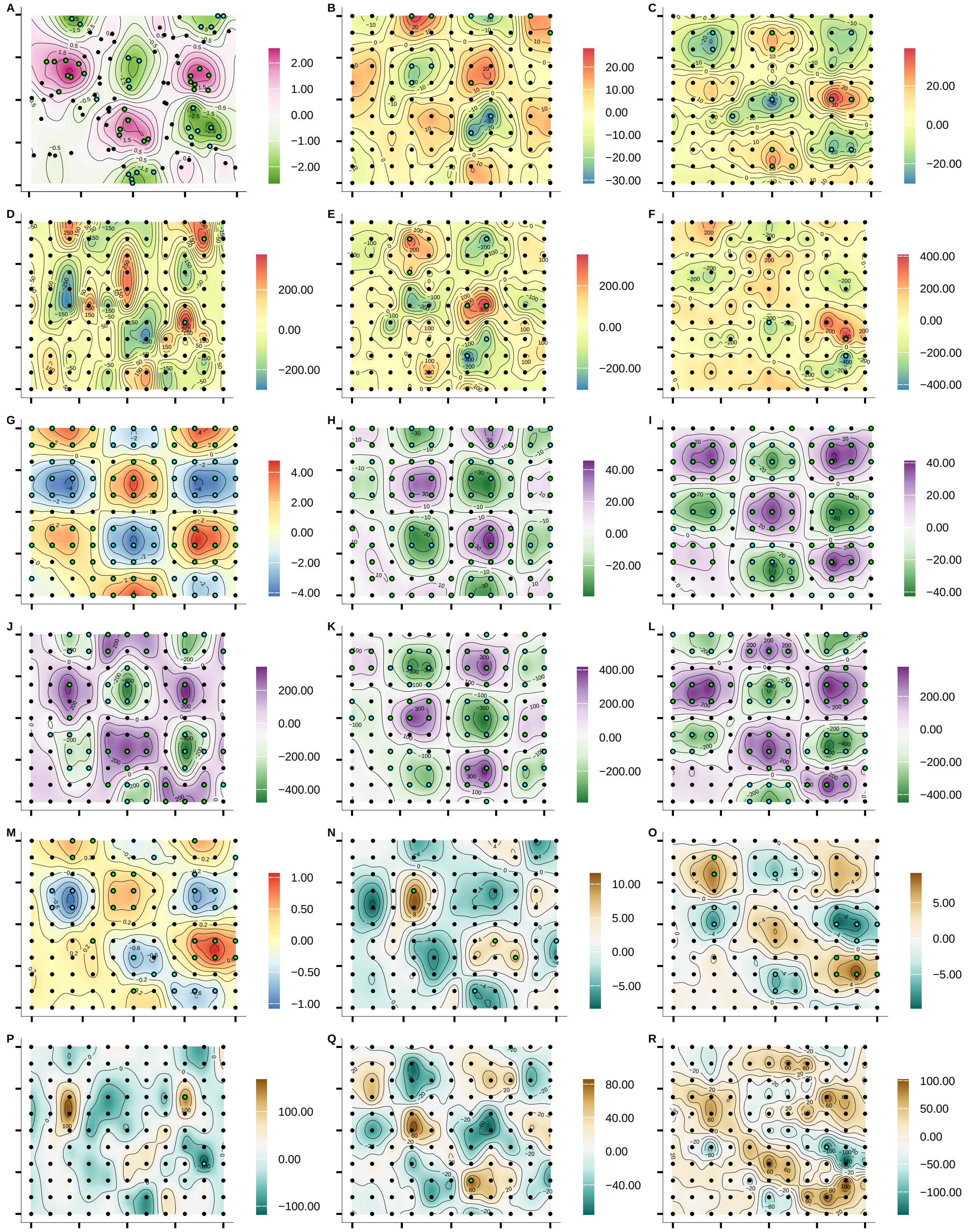

Figure S12: Estimated spatiotemporal process, derivatives and curvature for $t = 3$. (A) response from Pattern 2, (B) spatial gradient along x, (C) along y, (D) spatial curvature along x, (E) along x and y and (F) along y, (G–L) temporal gradients for processes in (A–F), (M–R) temporal curvature for processes in (A–F).

S18.2 Differential Geometric Operators

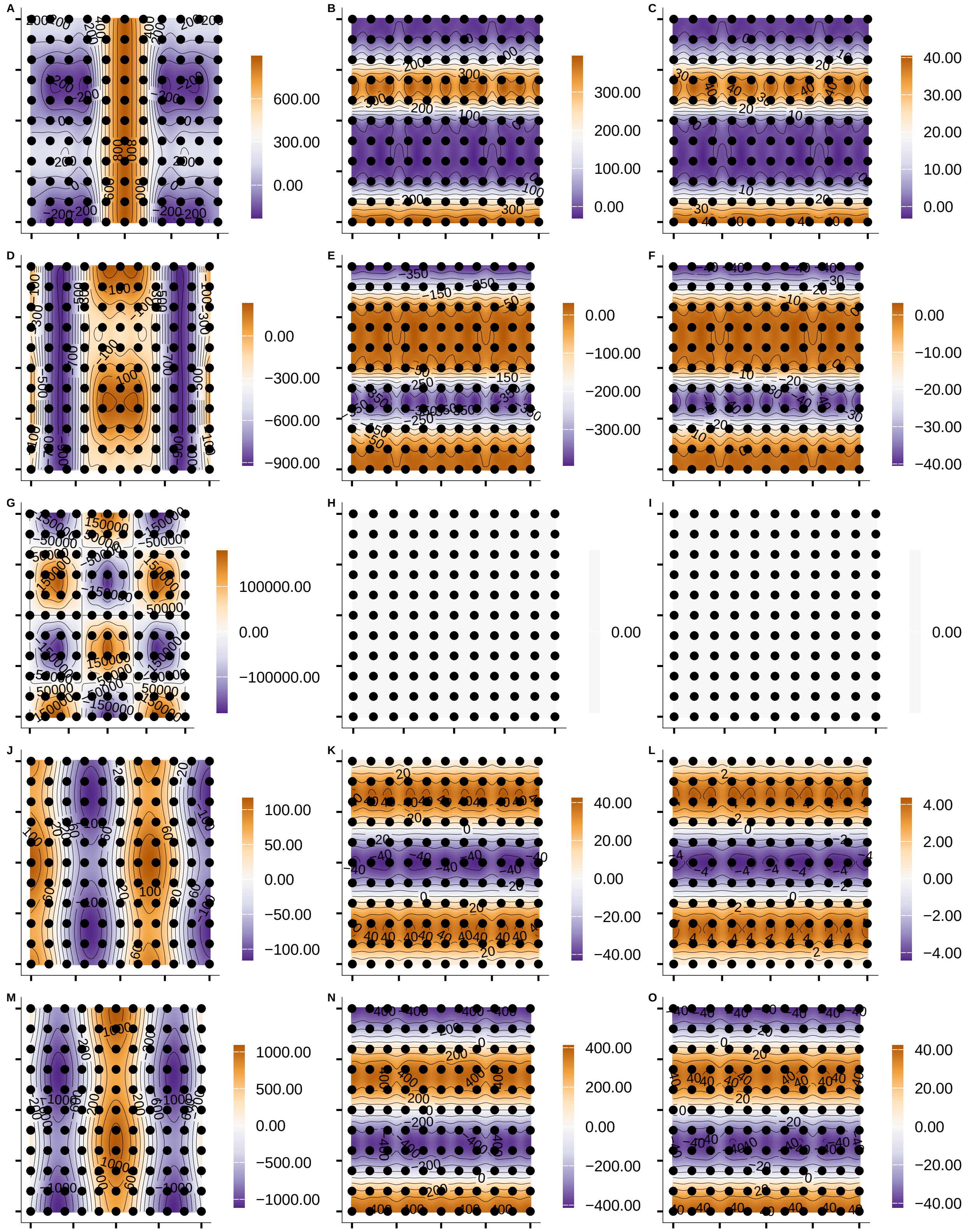

Figure S13: True spatiotemporal differential geometric processes for $t = 3$. (A–C) Eigen-value 1, its temporal derivative and curvature (D–F) Eigen-value 2 its temporal derivative and curvature (G–I) Gaussian curvature, its temporal derivative and curvature (J–L) Divergence (see pp. 10–11) (M–O) Laplacian (see pp. 10–11).

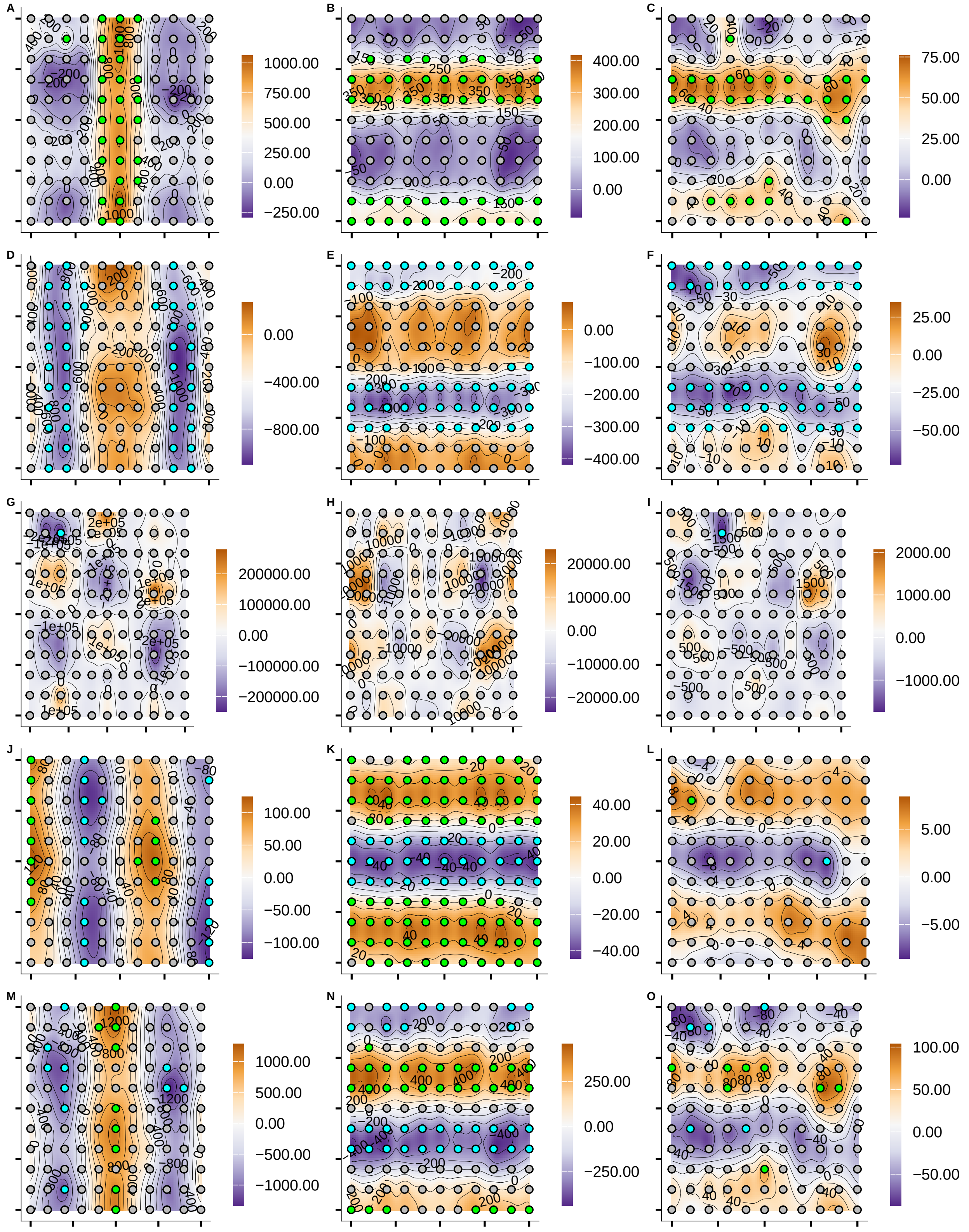

Figure S14: Estimated spatiotemporal differential geometric processes for $t = 3$. (A–C) Eigen-value 1, its temporal derivative and curvature (D–F) Eigen-value 2 its temporal derivative and curvature (G–I) Gaussian curvature, its temporal derivative and curvature (J–L) Divergence (see pp. 10–11) (M–O) Laplacian (see pp. 10–11). Significant grid locations are in color—*green* indicates significant positive and *cyan* indicates significant negative values.

S18.3 Spatiotemporal Surface Wombling: Significance

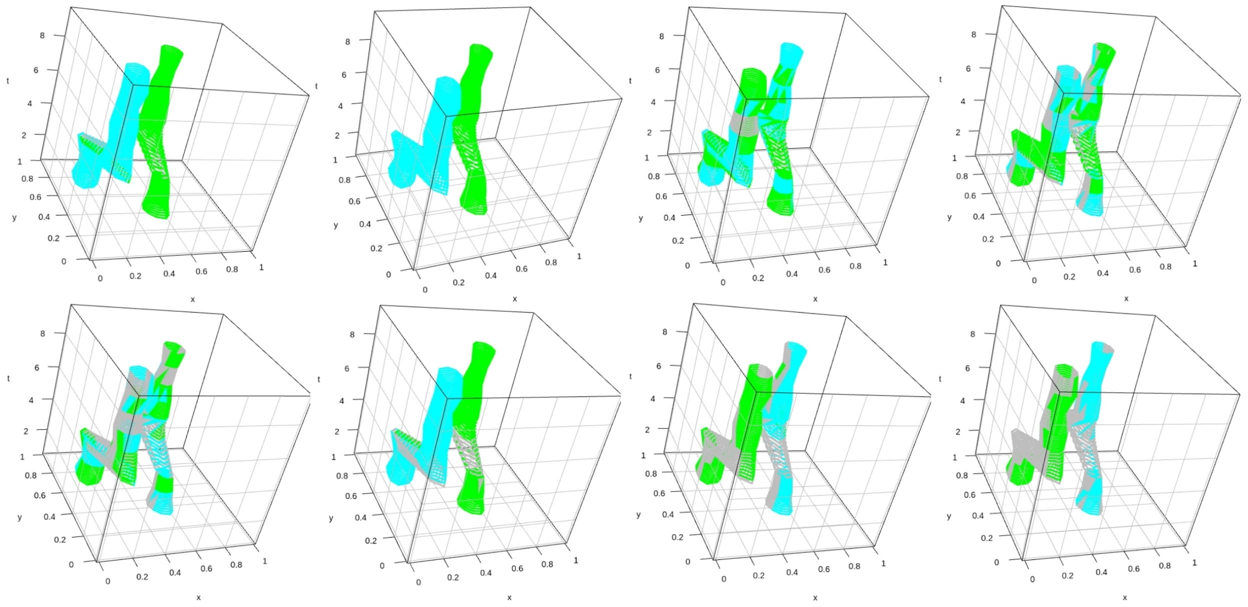

(a) Wombling Surfaces A and B

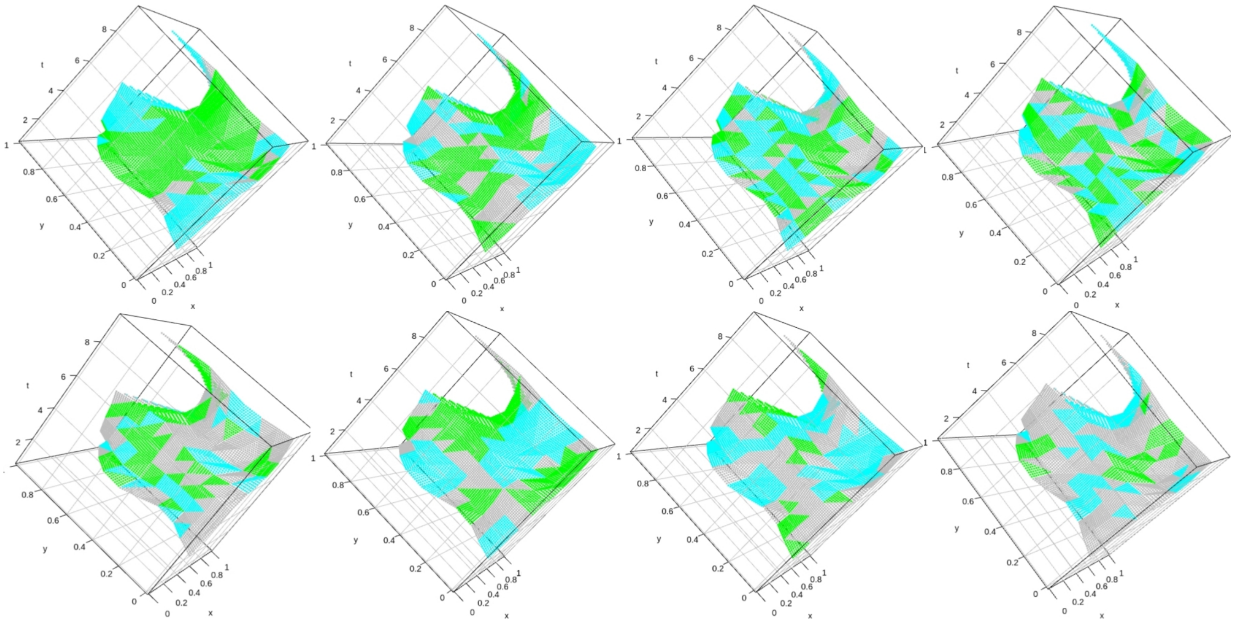

(b) Wombling Surface C

Figure S15: Significance of spatiotemporal wombling measures for wombling surfaces (a) A, B and (b) C (see Section 5.3, fig. 4). (Row 1: left to right) spatial gradient, spatial curvature, temporal gradient, spatial-temporal gradient, (Row 2: left to right) temporal gradient in spatial curvature, temporal curvature, temporal curvature in spatial gradient, spatial-temporal curvature. Regions marked `green` (`cyan`) indicate positive (negative) significance while `grey` indicates no significance.

S18.4 Applications: Neuroimaging

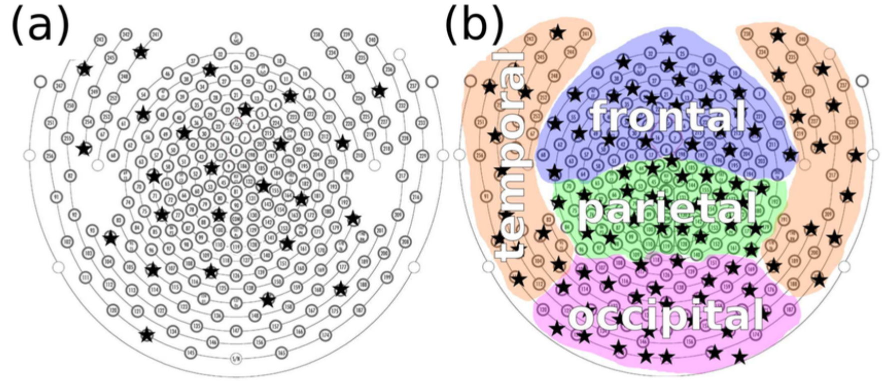

Figure S16: Scalp regions overlaid over a 256 electrode EEG cap for recording measurements. Figure (a) shows the electrode markings/locations and (b) shows the regions.

References

Adler, R. J. (1981), *The Geometry of Random Fields*, SIAM.

Banerjee, S. & Gelfand, A. E. (2005), 'Boundary Analysis: Significance and Construction of Curvilinear Boundaries', *Journal of the American Statistical Association. To appear* .

Banerjee, S. & Gelfand, A. E. (2006), 'Bayesian Wombling: Curvilinear Gradient Assessment Under Spatial Process Models', *Journal of the American Statistical Association* **101**(476), 1487–1501.

Banerjee, S., Gelfand, A. E. & Sirmans, C. (2003), 'Directional Rates of Change Under Spatial Process Models', *Journal of the American Statistical Association* **98**(464), 946–954.

Birant, D. & Kut, A. (2007), 'ST-DBSCAN: An algorithm for clustering spatial–temporal data', *Data & Knowledge Engineering* **60**(1), 208–221.

Biswas, S. & Hazra, R. (2022), 'State-of-the-Art Level Set Models and Their Performances in Image Segmentation: A Decade Review', *Archives of Computational Methods in Engineering* **29**(4), 2019–2042.

Bivand, R. S. & Wong, D. W. S. (2018), 'Comparing implementations of global and local indicators of spatial association', *TEST* **27**(3), 716–748.

Bochner, S. (2005), *Harmonic Analysis and the Theory of Probability*, Courier Corporation.

Boledi, L., Terschanski, B., Elgeti, S. & Kowalski, J. (2022), 'A level-set based space-time finite element approach to the modelling of solidification and melting processes', *Journal of Computational Physics* **457**, 111047.

Butt, U. M., Letchmunan, S., Hassan, F. H., Ali, M., Baqir, A. & Sherazi, H. H. R. (2020), 'Spatio-temporal crime hotspot detection and prediction: a systematic literature review', *IEEE access* **8**, 166553–166574.

Chen, K., Ma, Y., Bell, M. L. & Yang, W. (2023), 'Canadian Wildfire Smoke and Asthma Syndrome Emergency Department Visits in New York City', *JAMA* **330**(14), 1385–1387.

Di Martino, F., Pedrycz, W. & Sessa, S. (2018), 'Spatiotemporal extended fuzzy c-means clustering algorithm for hotspots detection and prediction', *Fuzzy sets and systems* **340**, 109–126.

Dorabiala, O., Dabke, D. V., Webster, J., Kutz, N. & Aravkin, A. (2024), 'Spatiotemporal k-means'. arXiv:2211.05337 [cs].

Duan, J. A., Guindani, M. & Gelfand, A. E. (2007), 'Generalized Spatial Dirichlet Process Models', *Biometrika* **94**(4), 809–825.

Fanaee-T, H. & Gama, J. (2015), 'Eigenspace method for spatiotemporal hotspot detection', *Expert systems* **32**(3), 454–464.

Fuentes, M., Chen, L. & Davis, J. M. (2008), 'A Class of Nonseparable and Nonstationary Spatial Temporal Covariance Functions', *Environmetrics: The official journal of the International Environmetrics Society* **19**(5), 487–507.

Gelfand, A., Guindani, M., Petrone, S. et al. (2007), Bayesian Nonparametric Modelling for Spatial Data Using Dirichlet Processes, *in* 'Bayesian Statistics 8', Oxford University Press, pp. 175–200.

Getis, A. & Ord, J. K. (1992), 'The Analysis of Spatial Association by Use of Distance Statistics', *Geographical Analysis* **24**(3), 189–206. _eprint: https://onlinelibrary.wiley.com/doi/pdf/10.1111/j.1538-4632.1992.tb00261.x.

Gneiting, T. (2002), 'Nonseparable, Stationary Covariance Functions for Space–Time Data', *Journal of the American Statistical Association* **97**(458), 590–600.

Graham, A. (2018), *Kronecker Products and Matrix Calculus with Applications*, Courier Dover Publications. Google-Books-ID: XMJeDwAAQBAJ.

Halder, A., Banerjee, S. & Dey, D. K. (2024), 'Bayesian Modeling with Spatial Curvature Processes', *Journal of the American Statistical Association* **119**(546), 1155–1167.

He, K., Zhang, X., Ren, S. & Sun, J. (2016), Deep Residual Learning for Image Recognition, *in* 'Proceedings of the IEEE Conference on Computer Vision and Pattern Recognition (CVPR)', pp. 770–778.

Kottas, A., Duan, J. A. & Gelfand, A. E. (2008), 'Modeling Disease Incidence Data with Spatial and Spatio Temporal Dirichlet Process Mixtures', *Biometrical Journal* **50**(1), 29–42.

Lie, J., Lysaker, M. & Tai, X.-C. (2006), 'A binary level set model and some applications to mumford-shah image segmentation', *IEEE transactions on image processing* **15**(5), 1171–1181.

Malladi, R., Sethian, J. & Vemuri, B. (1995), 'Shape modeling with front propagation: a level set approach', *IEEE Transactions on Pattern Analysis and Machine Intelligence* **17**(2), 158–175.

Marra, F., Armon, M., Borga, M. & Morin, E. (2021), 'Orographic Effect on Extreme Precipitation Statistics Peaks at Hourly Time Scales', *Geophysical Research Letters* **48**(5), e2020GL091498.

Ord, J. K. & Getis, A. (1995), 'Local Spatial Autocorrelation Statistics: Distributional Issues and an Application', *Geographical Analysis* **27**(4), 286–306. _eprint: https://onlinelibrary.wiley.com/doi/pdf/10.1111/j.1538-4632.1995.tb00912.x.

Osher, S. & Fedkiw, R. P. (2001), 'Level Set Methods: An Overview and Some Recent Results', *Journal of Computational Physics* **169**(2), 463–502.

Osher, S. & Sethian, J. A. (1988), 'Fronts propagating with curvature-dependent speed: Algorithms based on Hamilton-Jacobi formulations', *Journal of Computational Physics* **79**(1), 12–49.

Quick, H., Banerjee, S. & Carlin, B. P. (2015), 'Bayesian Modeling and Analysis for Gradients in Spatiotemporal Processes', *Biometrics* **71**(3), 575–584.

R Core Team (2021), *R: A Language and Environment for Statistical Computing*, R Foundation for Statistical Computing, Vienna, Austria.

Rasmussen, C. E. & Williams, C. K. I. (2005), *Gaussian Processes for Machine Learning*, The MIT Press.

Ronneberger, O., Fischer, P. & Brox, T. (2015), U-Net: Convolutional Networks for Biomedical Image Segmentation, *in* N. Navab, J. Hornegger, W. M. Wells & A. F. Frangi, eds, 'Medical Image Computing and Computer-Assisted Intervention – MICCAI 2015', Springer International Publishing, Cham, pp. 234–241.

Ross, G. J. & Markwick, D. (2018), 'dirichletprocess: An R Package for Fitting Complex Bayesian Nonparametric Models'.

Saad-Falcon, A., Howard, C., Romberg, J. & Allen, K. (2024), 'Level set methods for gradient-free optimization of metasurface arrays', *Scientific Reports* **14**(1), 16674. Publisher: Nature Publishing Group.

Spivak, M. (1979), *A Comprehensive Introduction To Differential Geometry*, number v. 1–5 *in* 'A Comprehensive Introduction to Differential Geometry', Publish or Perish Inc.

Stein, M. L. (2005), 'Space–Time Covariance Functions', *Journal of the American Statistical Association* **100**(469), 310–321.

Sussman, M., Fatemi, E., Smereka, P. & Osher, S. (1998), 'An improved level set method for incompressible two-phase flows', *Computers & Fluids* **27**(5-6), 663–680.

Sussman, M., Smereka, P. & Osher, S. (1994), 'A level set approach for computing solutions to incompressible two-phase flow', *Journal of Computational physics* **114**(1), 146–159.

Vetter, W. (1970), 'Derivative Operations on Matrices', *IEEE Transactions on Automatic Control* **15**(2), 241–244. Conference Name: IEEE Transactions on Automatic Control.

Wang, F., Bhattacharya, A. & Gelfand, A. E. (2018), 'Process Modeling for Slope and Aspect with Application to Elevation Data Maps', *TEST* **27**(4), 749–772.

Zou, K. H., Warfield, S. K., Bharatha, A., Tempany, C. M., Kaus, M. R., Haker, S. J., Wells, W. M., Jolesz, F. A. & Kikinis, R. (2004), 'Statistical Validation of Image Segmentation Quality Based on a Spatial Overlap Index', *Academic radiology* **11**(2), 178–189.
