# Supplementary material for: Bayesian Spatiotemporal Wombling
Source: J Am Stat Assoc. Author manuscript; Available in PMC 2026 Oct 15. (PMC13664449; doi:10.1080/01621459.2026.2700798)
Supplement: Supplementary file 1 [file NIHMS2211750-supplement-Supp_1.zip › uasa_a_2700798_sm0137.pdf]

This form documents the artifacts associated with the article (i.e., the data and code supporting the computational findings) and describes how to reproduce the findings.

Part 1: Data

☐ This paper does not involve analysis of external data (i.e., no data are used or the only data are generated by the authors via simulation in their code).

☒ I certify that the author(s) of the manuscript have legitimate access to and permission to use the data used in this manuscript.

Abstract

We use three datasets in the manuscript: (i) precipitation data for northern California obtained from National Ocean https://www.ncei.noaa.gov/cdo-web/ (ii) ambient particle concentration ($PM_{2.5}$) data for the north mid-Atlantic and New England region, United States and (iii) Event-related potentials arising from electroencephalography sessions conducted with the purpose of detecting predisposition to alcoholism. Each datset contains spatiotemporal coordinates used for referencing. They are available for free access at GitHub repository:`REDACTED` (available in unblinded version)

Availability

☒ Data **are** publicly available.
☐ Data **cannot be made** publicly available.

If the data are publicly available, see the *Publicly available data* section. Otherwise, see the *Non-publicly available data* section, below.

Publicly available data

☒ Data are available online at: GitHub repository:`REDACTED` (available in unblinded version)

☒ Data are available as part of the paper's supplementary material.

☐ Data are publicly available by request, following the process described here:

☐ Data are or will be made available through some other mechanism, described here:

Non-publicly available data

Description

File format(s)

☐ CSV or other plain text.
☒ Software-specific binary format (.Rda, Python pickle, etc.): pkcle
☐ Standardized binary format (e.g., netCDF, HDF5, etc.):
☐ Other (please specify):

Data dictionary

☐ Provided by authors in the following file(s):
☒ Data file(s) is(are) self-describing (e.g., netCDF files)
☐ Available at the following URL:

Additional Information (optional)

Part 2: Code

Abstract

The code includes a collapsed Markov Chain Monte Carlo sampler that (a) fits a hierarchical Bayesian spatiotemporal models (b) posterior prediction for rates of change/spatiotemporal differential processes and (c) performs surface wombling. It also contains a function for generating simulated data used in the manuscript, a plethora of plotting functions for diagnosing convergence and plotting surfaces.

Description

Code format(s)

- ☒ Script files
  - ☒ R
  - ☐ Python
  - ☐ Matlab
  - ☐ Other:
- ☒ Package
  - ☒ R
  - ☐ Python
  - ☐ MATLAB toolbox
  - ☐ Other:
- ☒ Reproducible report
  - ☐ R Markdown
  - ☐ Jupyter notebook
  - ☒ Other: JASA Reproducibility GitHub Repository
- ☐ Shell script
- ☐ Other (please specify):

Supporting software requirements

all supporting packages are part of the primary GitHub repository.

Version of primary software used R (>= 3.5.0)

Libraries and dependencies used by the code

Supporting system/hardware requirements (optional)

Parallelization used

- ☐ No parallel code used
- ☒ Multi-core parallelization on a single machine/node
  - Number of cores used: 24
- ☐ Multi-machine/multi-node parallelization
  - Number of nodes and cores used:

License

- ☒ MIT License (default)
- ☐ BSD
- ☐ GPL v3.0
- ☐ Creative Commons

☐ Other: (please specify)

Additional information (optional)

Part 3: Reproducibility workflow

Scope

The provided workflow reproduces:

☒ Any numbers provided in text in the paper
☒ The computational method(s) presented in the paper (i.e., code is provided that implements the method(s))
☒ All tables and figures in the paper
☐ Selected tables and figures in the paper, as explained and justified below:

Workflow

Location

The workflow is available:

☐ As part of the paper's supplementary material.
☒ In this Git repository: https://github.com/arh926/Bayesian-Spatiotemporal-Wombling
☐ Other (please specify):

Format(s)

☐ Single master code file
☐ Wrapper (shell) script(s)
☐ Self-contained R Markdown file, Jupyter notebook, or other literate programming approach
☒ Text file (e.g., a readme-style file) that documents workflow
☐ Makefile
☐ Other (more detail in *Instructions* below)

Instructions

Expected run-time

Approximate time needed to reproduce the analyses on a standard desktop machine:

☐ < 1 minute
☐ 1-10 minutes
☐ 10-60 minutes
☐ 1-8 hours
☒ > 8 hours
☒ Not feasible to run on a desktop machine, as described here: Run on a Linux machine powered by a 12th Gen. Intel Core-i9 12.9K with 24 cores and 64GB of RAM.

Additional information (optional)

Notes (optional)
